# Geminal homologative fluorination of carbonyl derivatives *en route* to 1-fluoro-2-haloethyl skeletons†

**DOI:** 10.1039/d5cc01542a

**Published:** 2025-06-06

**Authors:** Margherita Miele, Davide Castiglione, Alexander Prado-Roller, Laura Castoldi, Vittorio Pace

**Affiliations:** a Department of Chemistry, University of Turin Via Giuria 7 10125 Turin Italy margherita.miele@unito.it vittorio.pace@unito.it; b Institute of Inorganic Chemistry, University of Vienna Waehringerstrasse 42 1090 Vienna Austria; c Department of Pharmaceutical Sciences, General and Organic Chemisty Section “A. Marchesini” – Via Venezian 21, University of Milan 20133 Milan Italy laura.castoldi@unimi.it; d Department of Pharmaceutical Sciences, Division of Pharmaceutical Chemistry, University of Vienna Josef-Holaubek-Platz 2 1090 Vienna Austria vittorio.pace@univie.ac.at

## Abstract

Carbonyl groups undergo the sequential installation of two nucleophilic elements, halomethyl and fluoride moieties. This formal *gem*-difunctionalization enables the preparation–under full chemocontrol – of vic-fluorohaloethanes by simply defining the C1 nucleophile, thus enabling access to all combinations of the four halogens.

## Abstract 2

Carbonyl groups undergo the sequential installation of two nucleophilic elements, halomethyl and fluoride moieties.

## Graphical abstract

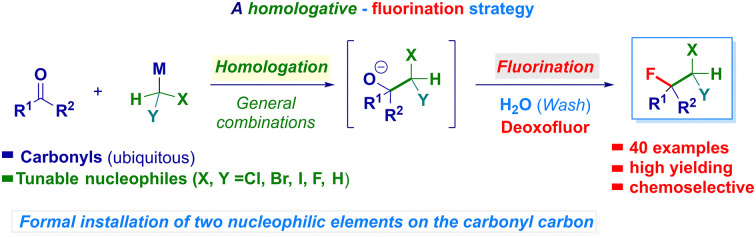

The unique ability of the fluorine atom to modulate the structural editing of organic skeletons constitutes a robust tool for the fine-tuning of pivotal physical–chemical parameters.^1^ This is mainly due to the following constitutive aspects: (a) local polarity inversion at the competent connective carbon; (b) minimal steric variation as a consequence of comparable van der Waals radii, *inter alia*.^2,3^ Positioning an (additional) distinct halogen on the vicinal carbon further amplifies the modular control of the stereoelectronic and conformational features of the resulting backbone exhibiting two adjacent sites amenable for regioselective diversification (Scheme 1, upper panel).^4^ Indeed, the selective insertion of one or two halogen atoms enables the precise design of bioisosters, and thus the generation of new entities suitable for undergoing pharmacokinetic and dynamic analysis, as well as for uncovering novel materials or agrochemicals.^5^ Historically, olefins have been used to forge 1,2-dihaloethyl units using different strategies based – *inter alia* – on a formal electrophilic addition with an X–Y type reagent (Scheme 1, path a).^6^ While documenting outstanding levels of stereocontrol in the case of preparing chloro- and bromo-analogues,^5,7^ as also shown in the preparation of halogenated natural products,^6*c*,8^ the access to 1-halo-2-fluoroethyl clusters still remains critical. The origin of this shortcoming is attributed to regioselective aspects arising from the inherent difficulty of controlling the attack of two halogens with opposite polarity and sensitively different *radii* (*e.g.* iodine *vs.* bromine or chlorine compared to fluorine).^4*a*,9^ Moreover, it is instructive to mention some substrate dependence that was noticed by O’Hagan when synthesizing 1-bromo-2-fluoro systems.^10^ Notably, the adoption of catalytic alkene-activation strategies^9*b*^ pioneered in fluorinative chemistry by Jacobsen,^11^ Gilmour^12^ and Lennox^13^ enabled the productive attack of two F^−^ anions, thus realizing the hitherto elusive 1,2-difluorination of olefins (Scheme 1, path b).^12*c*,14^ The concept was elegantly translated to a diastereodivergent assembly of chloro–fluoro–ethanes by Lennox in 2024 (Scheme 1, path c):^15^ indeed, the tunable electrochemical oxidative formation of distinct *λ*^3^-iodanes for activating the olefin allows the controlled installation of the inherently reactive (*i.e.* nucleophilic) fluoride and chloride anions by modulating their relative concentrations. Accordingly, both *anti* and *syn* addition products can be directly prepared. Unfortunately, this benchmark tactic is not expansive. Thus, designing a route enabling access to all the possible distinct fluorohaloethanes (chloro, bromo, iodo) persists as an unmet goal in current synthesis.^16^ Inspired by our interest in forging functionalized C–C bonds *via* electrophilic–nucleophilic reactivity,^17^ we questioned whether the release of halomethyl synthons (–CH_2_–Hal) to a competent recipient electrophilic acceptor (*i.e.* carbonyl moiety)–followed by direct deoxyfluorination^18^ of the addition intermediate–could supply a reliable preparative protocol for 1-halo-2-fluoroethyl chains (Scheme 1, path d). Indeed, the proposed approach relies on the documented flexibility of delivering the methylene unit featuring the exact degree of functionalization through the use of a proper (nucleophilic) lithium halocarbenoid (*i.e.* LiCHXY).^19^ Thus, after selecting the desired (tunable) CH_2_–Hal element to attack the carbonyl group, the subsequent fluorinative event conducted on the addition intermediate produces the targeted vic-fluorohaloethyl chain. Collectively, the transformation can be conceptualized as a carbonyl-geminal difunctionalization,^20^ with the initial carbonyl linchpin being the site of attack of the tunable CH_2_–Hal element (Cl, Br, F, I) and the subsequently introduced fluorine. Whenever productive, the strategy would *de facto* overcome the risk of low regiocontrol affecting dihalofunctionalizations of olefins.

**Scheme 1:**
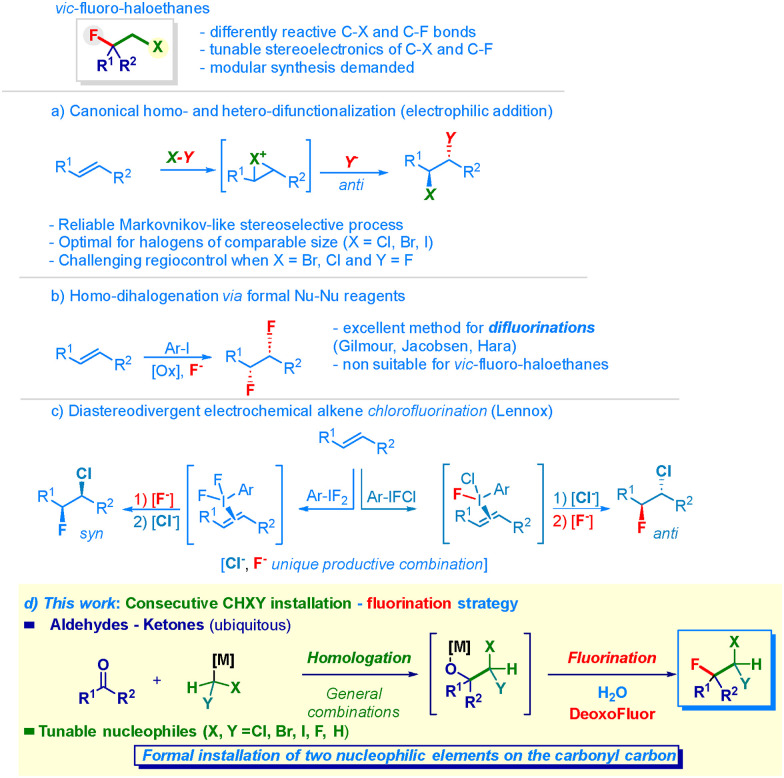
General context of the presented work.

ω-Chlorobutyrophenone 1 was selected as the model substrate featuring two reactive electrophilic sites that are potentially amenable to modification under the employed nucleophilic conditions (Table 1). The success of the ketone homologation was deeply influenced by the correct genesis of the carbenoid through I/Li exchange conducted on chloroiodomethane and MeLi–LiBr.^21^ In particular, the rate of addition of the latter played a critical role in maximizing the process (Table 1, green box). The controlled delivery (0.20 mL min^−1^) *via* a syringe pump enabled the precise generation of LiCH_2_Cl (1.4 equiv.) in THF at −78 °C, thus furnishing the lithiated tetrahedral intermediate adduct 1a – almost quantitatively within 30 min – as judged by the conversion into chlorohydrin 2a after acidic quenching (NH_4_Cl). Having established the feasibility of the initial event of the transformation occurring with promising chemoselective control (no change at the ω-chloro pendant functionality), we then focused on the direct nucleophilic fluorination of 1a. Accordingly, by adding DAST^22^ (1.5 equiv.) at −78 °C and leaving the reaction to slowly reach rt, the desired chloro–fluoro compound 2 was obtained in 21% yield together with halohydrin 2a (65%) and an appreciable amount of (unidentified) decomposition material (entry 1). Presumably, the structural characteristics of the tertiary alkoxide render the system primed for suffering competing side reactions such as eliminations and rearrangements.^23^ Considering that the carbenoid generation event might produce collateral entities altering the expected outcome,^24^ we were pleased to note that–upon washing with water, followed by re-solubilizing in DCM–the fluorination occurred with a significantly higher yield (46%) without detectable side products (entry 2). The effect of the temperature was remarkable: keeping it at −78 °C was detrimental and only traces of 2 were recovered after 24 h (entry 3), whereas increasing to 0 °C gave a modest 13% yield (entry 4), suggesting a beneficial thermal activation during the C–O breaking step. Additional implementation – augmenting the yield up to 55% – was secured by employing 2.2 equiv. of DAST (entry 5). The screening of distinct S–F-type deoxyfluorinating agents known to be applicable to carbinols (entries 6–9), such as DeoxoFluor,^25^ XtalFluor,^26^ Pyfluor,^27^ and SulfoxFluor,^28^ evidenced the optimal performance of the former (entry 6), furnishing chloro–fluoro derivative 2 in an excellent 92% yield.

**Table 1:** Reaction optimization

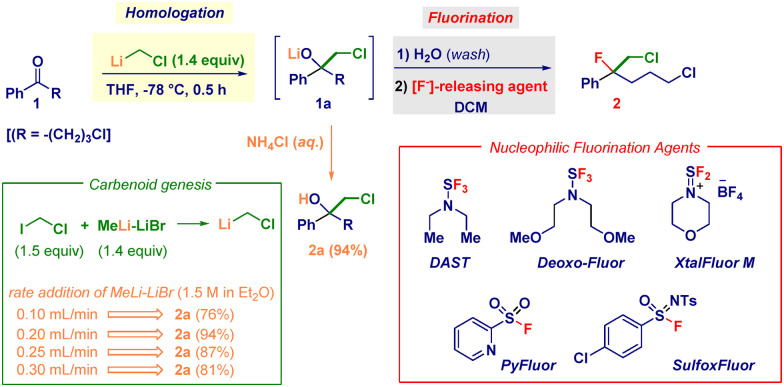				
Entry	Fluorinating agent (equiv.)	Temperature [°C]	Yield of 2^b^ (%)	Yield of 2a^a^ (%)
1^b^^c^	DAST (1.5)	−78 to rt	21	65
2	DAST (1.5)	−78 to rt	46	39
3^d^	DAST (1.5)	−78	—	90
4^d^	DAST (1.5)	0	13	74
5^*e*^	DAST (2.2)	−78 to rt	55	31
6	DeoxoFluor (2.2)	−78 to rt	92	—
7	XtalFluor M (2.2)	−78 to rt	82	13
8	PyFluor (2.2)	−78 to rt	77	17
9	SulfoxFluor (2.2)	−78 to rt	71	21

a Isolated yield after the homologation/fluorination sequence (o/n). Unless otherwise stated, after completing the addition of MeLi–LiBr, the reaction mixture was washed with purified water, evaporated and redissolved in DCM to reach a concentration of 0.5 M.

b DAST was added directly to the mixture without water washing.

c Unidentified products (10% NMR).

d The treatment with DAST was prolonged for 24 h.

With the optimal conditions in hand, we undertook a study of the scope of the sequential homologation–nucleophilic fluorinative transformation (Scheme 2). Four- and three-carbon phenones gave chloro–fluoro ethyl-units 2–4 in excellent yields. Acetophenones featuring a variety of different substituents on the aromatic ring [fluoro-(5, 6), trifluoromethyl (7), nitro (8), bromine (9), hydrogen (*i.e.* unsubstituted, 10)] provided the targeted manifolds in comparable high efficiency. Notably, α,α,α-trifluoromethyl-acetophenone could be used for assembling rare chloro-tetrafluoro-analogue 11 through a conceptually simple approach. Benzophenones (12–15) were equally compatible with the methodology: again, no significant difference was found when installing functionalities such as a second fluorine atom (13), an ether (14) or a thioether (15). To our great delight, the delivery of diverse C1 units exhibiting one, two or three fluorine atoms enabled the smooth preparation of extremely challenging poly-fluoro alkyl chains 16–18.^29^ In particular, for the 1,2-difluoroethyl analogue 16, LiCH_2_F^30^ was used, whereas the CHF_2_ residue – for constructing 17 – was delivered upon the activation of the commercially available TMSCHF_2_ (in the presence of a Lewis base such as KO-*t*-Am).^31^ This latter reaction could be run at 20 mmol scale without affecting the efficiency. The release of the formal CF_3_ carbanion from the Ruppert–Prakash reagent (TMSCF_3_)^32^ guaranteed access to compound 18. Aliphatic ketones also underwent the consecutive transformation, as documented both in the case of cyclic analogues, such as the highly sterically hindered^33^ adamantyl (19) or cycloheptyl (20) derivatives, and the acyclic analogue 21. Moreover, the synthesis of bromo–chloro–fluoro analogue 22 was accomplished by using LiCHBrCl^34^ as the competent (first) nucleophile. It should be noted that no modification occurred at the (electrophilic) nitrile functionality. The protocol could be advantageously applied for converting aldehydes into the corresponding fluorohaloethyl motifs. Thus, a series of benzaldehydes furnished products characterized by the presence of substituents of different chemical behavior, ranging from alkyl (23) to halogens (24–27) and trifluoromethyl (28), as well as nitrile (29), nitro (30) and ether (31). It is important to note that substitution at positions 2 and 6 of the phenyl ring (25) does not affect the effectiveness of the transformation. Analogously, by reacting a heteroaromatic aldehyde (3-thienyl) under the usual conditions, structure 32 was formed. The overall reaction exhibited a truly chemoselective profile: in fact, not only could the aforementioned nitrile groups be conveniently placed on the aromatic rings (22, 29) but also when an ester (33) or a piperidyl-amide (34) were present, the unique reactive site was the aldehyde carbonyl. The latter case is significant since nucleophilic acyl substitutions on these substrates are known.^35^ Furthermore, aliphatic and α,β-unsaturated aldehydes produced the chloro–fluoro-homologated adducts (35–37) under identical reaction conditions. As illustrated above, the selection of the first nucleophilic element to be added enables diversification of the substitution pattern of the resulting fluorohaloethyl chain. Therefore, LiCH_2_Br^21*c*^ or LiCHBr_2_^34^ generated platforms 38 and 39, whereas by employing the formal CCl_3_^−^ anion (see ESI†), the trichloro–fluoro system 40 was prepared. Finally, the addition of LiCH_2_I^21*a*^ provided a convenient route to the fluoro–iodo backbone 41 in which the styryl moiety maintained its chemical integrity.

**Scheme 2:**
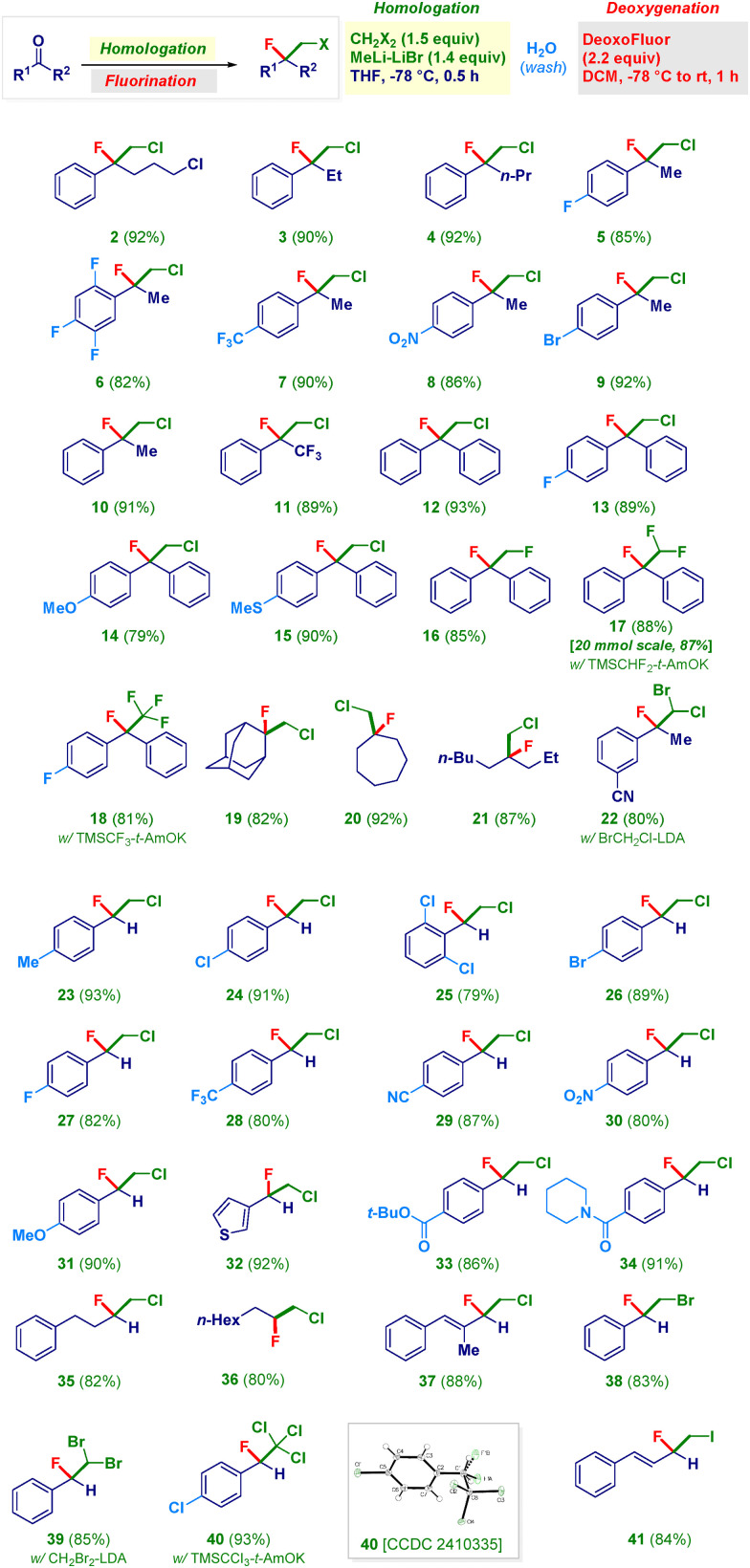
Scope of the sequential homologative fluorination procedure.

To rationalize the process, we conducted the *gem*-functionalization of an aldehyde in the absence of a halomethyl-releasing agent. To this end, upon the addition of simple MeLi to *p*-chlorobenzaldehyde followed by the usual treatment with DeoxoFluor, we noticed a dramatic increase in the reaction time (24 h) required to furnish the expected fluorinated compound 42 (Scheme 3, path a). Presumably, the constitutive lack of a halogen does not offer the possibility of creating a five-membered halogen-bond linchpin^36^ for activating the putative alkoxide towards the reaction with DeoxoFluor. This outcome is evidently not observed with LiCH_2_Cl (Scheme 3, path b) which guarantees the productive triggering of the subsequent fluorinative event, as also documented by the different isolated yields (54% for 42*vs.* 91% for 24).

**Scheme 3:**
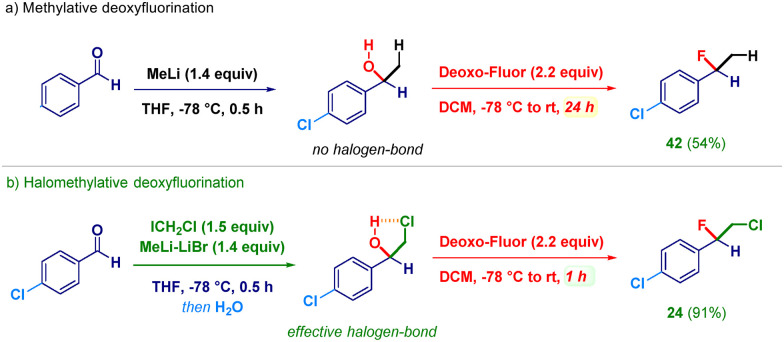
Plausible halogen-bond-triggered deoxyfluorination.

In summary, we have documented the formal geminal difunctionalization of carbonyl linchpins (aldehydes and ketones) with a (poly)-halomethyl fragment and a fluoride anion *en route* to 1-fluoro-2-haloethyl skeletons. The transformation is based on the chemoselective nucleophilic attack of the halogenated C1-synthon followed by the straightforward deoxyfluorination of the putative alcohol with DeoxoFluor. Through the judicious selection of the first nucleophile (M-CXYZ), a high degree of flexibility can be imparted to the protocol.

We thank the University of Vienna, the University of Turin, the University of Milan and All4Labels Group (Hamburg, Germany) for generous funding. Financial support from PRIN projects no. 20228W9TBL (L. C.) and no. 2022JLSZMY (V. P.), and FWF-Austria Project no. P 37068-B (V. P.) is gratefully acknowledged. The authors thank Prof. W. Holzer (University of Vienna) for NMR elucidations.

## Conflicts of interest

There are no conflicts to declare.

## Supplementary Material

CC-061-D5CC01542A-s001

CC-061-D5CC01542A-s002

## Data Availability

The data supporting this article have been included as part of the ESI.†
