# Supplementary material for: Geminal homologative fluorination of carbonyl derivatives en route to 1-fluoro-2-haloethyl skeletons
Source: Chem Commun (Camb). 2025 Jun 6;61(58):10792–5. doi: 10.1039/d5cc01542a (PMC12179841; doi:10.1039/d5cc01542a)
Supplement: CC-061-D5CC01542A-s001 [file CC-061-D5CC01542A-s001.pdf]

Supplementary Information (SI) for ChemComm.
This journal is © The Royal Society of Chemistry 2025

ELECTRONIC SUPPORTING INFORMATION

For

Geminal Homologative Fluorination of Carbonyl Derivatives *en route* to 1-Fluoro-2-Haloethyl- Skeletons

Margherita Miele, [a*] Davide Castiglione,[a] Alexander Prado-Roller,[b] Laura Castoldi,[c*] and Vittorio Pace[a,d*]

[a] University of Turin - Department of Chemistry – Via P. Giuria 7, 10125, Turin, Italy.

[b] University of Vienna – Institute of Inoganic Chemistry - Waehringerstrasse 42, 1090, Vienna, Austria.

[c] University of Milan - Department of Pharmaceutical Sciences, General and Organic Chemisty Section "A. Marchesini" – Via Venezian 21, 20133 Milan, Italy.

[b] University of Vienna - Department of Pharmaceutical Sciences, Division of Pharmaceutical Chemistry - Josef-Holaubek-Platz 2, 1090, Vienna, Austria.

e-mails: margherita.miele@unito.it; laura.castoldi@unimi.it; vittorio.pace@univie.ac.at; vittorio.pace@unito.it

TABLE OF CONTENTS

**INSTRUMENTATION AND GENERAL ANALYTICAL METHODS**	S2
**GENERAL PROCEDURE**	S3
**CHARACTERIZATION AND SPECTRAL DATA OF COMPOUNDS**	S4
**REFERENCES**	S24
**COPIES OF NMR SPECTRA (^{1}*H-*, ^{13}*C-*, *HETERONUCLEI*)**	S25
**X-RAY ANALYSIS**	S129

1. Materials and methods

Melting Points were determined on a Reichert-Kofler hot-stage microscope and are uncorrected. Mass spectra were obtained on a Shimadzu QP 1000 instrument (EI, 70 eV) and on a Bruker maXis 4G instrument (ESI-TOF, HRMS). ^{1}H, ^{13}C and ^{19} F NMR spectra were recorded at 297 K on a Bruker Avance III 400 spectrometer (400 MHz for ^{1}H, 100 MHz for ^{13}C, 40 MHz for ^{15}N, 376 MHz for ^{19}F) equipped with a directly detecting broadband observe (BBFO) probe, with a Bruker Avance III 500 spectrometer (500 MHz for ^{1}H, 125 MHz for ^{13}C) using a Prodigy cryoprobe, and with a Bruker DRX 200 spectrometer (200 MHz for ^{1}H, 50 MHz for ^{13}C) with a ^{1}H/^{13}C dual probe.
The centre of the solvent signal was used as an internal standard which was related to TMS with δ 7.26 ppm (^{1}H in $CDCl_3$), δ 7.16 ppm (^{1}H in C_6D_6), δ 77.00 ppm (^{13}C in $CDCl_3$) and δ 128.06 ppm (^{13}C in C_6D_6). Absolute referencing via *Ξ* ratio was usd for the ^{19}F NMR spectra. Spin-spin coupling constants (*J*) are given in Hz.
In nearly all cases, full and unambiguous assignment of all resonances was performed by combined application of standard NMR techniques, such as APT, HSQC, HMBC, HSQC-TOCSY, COSY and NOESY experiments.
All the reactions were carried out under inert atmosphere of argon. THF was distilled over Na/benzophenone. Chemicals were purchased from Sigma-Aldrich, Acros, Alfa Aesar and TCI Europe. Solutions were evaporated under reduced pressure with a rotary evaporator.
TLC was carried out on aluminium sheets precoated with silica gel 60F254 (Merchery-Nagel, Merk); the spots were visualised under UV light (λ = 254 nm).

2. General procedures

General Procedure 1

To a solution of carbonyl compound (aldehyde or ketone, 1.0 equiv) in dry THF (3 mL) cooled at -78 °C, the dihalomethane carbenoid precursor was added (1.5 equiv) under an Argon atmosphere. After 10 min, MeLi-LiBr 2.2 M solution in Et_2O (1.4 equiv) was added with a syringe pump 0.20 mL/min and the stirring was continued for additional 0.5 h. Subsequently, distilled water was added to the mixture and the cooling bath was removed; the organic phase was extracted with dichloromethane (3 x 3 mL) and, dried over anhydrous Na_2SO_4. The filtered solution (1.5 mL) was flushed under argon and Deoxo-Fluor 2.7 M solution in Toluene (2.2 equiv) was incorporated to it at room temperature and, the reaction was stirred overnight. Finally, the mixture was quenched with water (3 mL) and extracted with dichloromethane (3 mL). The organic layer was washed with saturated (*aq.*) NaCl (5 mL), dried over anhydrous Na_2SO_4, filtered and concentrated under reduced pressure (bath: rt) to give the crude compound eventually purified as indicated below.

General Procedure 2

To a solution of dihalomethane carbenoids precursor (1.5 equiv) in dry THF (3 mL), cooled at -78 °C, was added LDA (1.4 equiv) with a syringe pump 0.20 mL/min under n Argon atmosphere. After 30 min, the carbonyl compound (aldehyde or ketone, 1 equiv) was added dropwise during a period of 15 min and, then the stirring was continued for additional 0.5 h. Subsequently, distilled water was added to the mixture and the cooling bath was removed; the organic phase was extracted with dichloromethane (3 x 3 mL) and, dried over anhydrous Na_2SO_4. The filtered solution (1.5 mL) was flushed under argon and Deoxo-Fluor 2.7 M solution in Toluene (2.2 equiv) was incorporated to it at room temperature and, the reaction was stirred overnight. Finally, the mixture was quenched with water (3 mL) and extracted with dichloromethane (3 mL). The organic layer was washed with saturated (*aq.*) NaCl (5 mL), dried over anhydrous Na_2SO_4, filtered and concentrated under reduced pressure (bath: rt) to give the crude compound eventually purified as indicated below.

General Procedure 3

To a solution of carbonyl compound (aldehyde or ketone, 1.0 equiv) in dry THF (3 mL) cooled at 0 °C, $TMSCHF_2$ or $TMSCF_3$ or $TMSCCl_3$ was added (2.0 equiv) under an Argon atmosphere. After 5 min, potassium *tert*-pentoxide (in toluene 0.9 M, 1.8 equiv) was added dropwise and, then the stirring was continued for additional 0.5 h. Subsequently, distilled water was added to the mixture and the cooling bath was removed; the organic phase was extracted with dichloromethane (3 x 3 mL) and, dried over anhydrous Na_2SO_4. The filtered solution (1.5 mL) was flushed under argon and Deoxo-Fluor 2.7 M solution in Toluene (2.2 equiv) was incorporated to it at room temperature and, the reaction was stirred overnight. Finally, the mixture was quenched with water (3 mL) and extracted with dichloromethane (3 mL). The organic layer was washed with saturated (*aq.*) NaCl (5 mL), dried over anhydrous Na_2SO_4, filtered and concentrated under reduced pressure (bath: rt) to give the crude compound eventually purified as indicated below.

3. Spectral and Characterization Data

Compound 2
(1,5-dichloro-2-fluoro-2-pentanyl) benzene

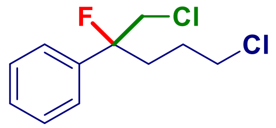

By following the **General procedure 1**, starting from 4-chloro-1-phenylbutan-1-one (200 mg, 1.1 mmol, 1.0 equiv) in dry THF (3 mL), chloroiodomethane (0.12 mL, 1.64 mmol, 1.5 equiv), MeLi-LiBr 2.2 M solution in Et_2O (0.7 mL, 1.54 mmol, 1.4 equiv), Deoxo-Fluor 2.7 M solution in Toluene (0.9 mL, 2.4 mmol, 2.2 equiv), **compound 2** was obtained in 92% yield (237 mg) as colorless oil without any further purification.

^{1}H NMR (400 MHz, $CDCl_3$) δ: 7.43-7.38 (m, 2H, Ph H-3,5), 7.37-7.32 (m, 3H, Ph H-2,4,6), 3.84-3.75 (m, 2H, CH_2Cl), 3.54-3.47 (m, 2H, CH_2CH_2Cl), 2.44-2.07 (m, 2H, CH_2CH_3), 1.91-1.56 (m, 2H, CH_2CH_2).
^{13}C NMR (100 MHz, $CDCl_3$) δ: 139.3 (d, 1C, $^2J_{C,F}$ = 21.8 Hz, Ph C-1), 128.6 (d, 2C, $^4J_{C,F}$ = 1.9 Hz, Ph C-3,5), 128.2 (d, 1C, $^5J_{C,F}$ = 1.0 Hz, Ph C-4), 124.7 (d, 2C, $^3J_{C,F}$ = 10.0 Hz, Ph C-2,6), 97.4 (d, 1C, $^1J_{C,F}$ = 182.4 Hz, CHF), 51.0 (d, 1C, $^2J_{C,F}$ = 27.6 Hz, CH_2Cl), 44.8 (1C, CH_2Cl), 34.3 (d, 1C, $^2J_{C,F}$ = 22.5 Hz, CH_2CH_2), 26.3 (d, 1C, $^3J_{C,F}$ = 3.1 Hz, $CH_2CH_2CH_2$).
^{19}F NMR (470 MHz, $CDCl_3$) δ: -162.6 (m, 1F, F-1).
HRMS (ESI), *m/z*: calcd. for $C_{11}H_{13}Cl_2FNa^+$: 257.0276 $[M+Na]^+$; found:257.0280.

Compound 2a
1,5-dichloro-2-phenylpentan-2-ol

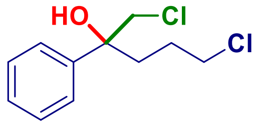

By following the **General procedure 1**, starting from 4-chloro-1-phenylbutan-1-one (200 mg, 1.1 mmol, 1.0 equiv) in dry THF (3 mL), chloroiodomethane (0.12 mL, 1.64 mmol, 1.5 equiv), MeLi-LiBr 2.2 M solution in Et_2O (0.7 mL, 1.54 mmol, 1.4 equiv), after quenching with saturated (*aq.*) NH_4Cl (3 mL), **compound 2a** was obtained in 94% yield (240 mg) as colorless oil without any further purification.
^{1}H NMR (400 MHz, $CDCl_3$) δ: 7.40-7.34 (m, 4H, Ph H-2,3,5,6), 7.29-7.26 (m, 1H, Ph H-4), 3.85-3.78 (m, 2H, CH_2Cl), 3.46-3.43 (m, 2H, CH_2CH_2Cl), 2.68 (bs, OH), 2.07-1.99 (m, 2H, CH_2CH_3), 1.83-1.50 (m, 2H, CH_2CH_2).
^{13}C NMR (100 MHz, $CDCl_3$) δ: 142.1 (Ph C-1), 128.6 (2C, Ph C-3,5), 127.6 (Ph C-4), 124.3 (2C, Ph C-2,6), 75.9 (COH), 55.3 (CH_2Cl), 45.3 (CH_2Cl), 36.9 (CH_2CH_2), 26.9 ($CH_2CH_2CH_2$).
HRMS (ESI), *m/z*: calcd. for $C_{11}H_{14}Cl_2ONa^+$: 255.0319 $[M+Na]^+$; found:255.0322.

Compound 3
(1-chloro-2-fluoro-2-butanyl) benzene

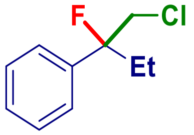

By following the **General procedure 1**, starting from propiophenone (200 mg, 1.5 mmol, 1.0 equiv) in dry THF (3 mL), chloroiodomethane (0.16 mL, 2.25 mmol, 1.5 equiv), MeLi-LiBr 2.2 M solution in Et_2O (1.0 mL, 2.1 mmol, 1.4 equiv), Deoxo-Fluor 2.7 M solution in Toluene (1.2 mL, 3.3 mmol, 2.2 equiv), **compound 3** was obtained in 90% yield (251 mg) as colorless oil without any further purification.

^{1}H NMR (400 MHz, C_6D_6) δ: 7.10-7.08 (m, 4H, Ph H-2,3,5,6), 7.06-7.02 (m, 1H, Ph H-4), 3.45-3.33 (m, 2H, CH_2Cl), 2.00-1.45 (m, 2H, CH_2CH_3), 0.66 (t, 3H, $^3J_{H,H}$ = 7.4 Hz, CH_3).
^{13}C NMR (100 MHz, C_6D_6) δ: 140.2 (d, 1C, $^2J_{C,F}$ = 21.8 Hz, Ph C-1), 128.6 (d, 2C, $^4J_{C,F}$ = 1.9 Hz, Ph C-3,5), 128.0 (d, 1C, $^5J_{C,F}$ = 1.1 Hz, Ph C-4), 125.2 (d, 2C, $^3J_{C,F}$ = 10.0 Hz, Ph C-2,6), 98.0 (d, 1C, $^1J_{C,F}$ = 182.4 Hz, CHF), 50.8 (d, 1C, $^2J_{C,F}$ = 27.5 Hz, CH_2Cl), 30.3 (d, 1C, $^2J_{C,F}$ = 23.2 Hz, CH_2CH_3), 7.4 (d, 1C, $^3J_{C,F}$ = 4.5 Hz, CH_2CH_3).
^{19}F NMR (470 MHz, C_6D_6) δ: -164.4 (m, 1F, F-1).
HRMS (ESI), *m/z*: calcd. for $C_{10}H_{12}ClFNa^+$: 209.0509 $[M+Na]^+$; found:209.0511.

Compound 4
(1-chloro-2-fluoro-2-pentanyl) benzene

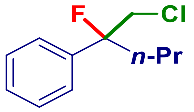

By following the **General procedure 1**, starting from 1-phenylbutan-1-one (200 mg, 1.35 mmol, 1.0 equiv) in dry THF (3 mL), chloroiodomethane (0.15 mL, 2.03 mmol, 1.5 equiv), MeLi-LiBr 2.2 M solution in Et_2O (0.86 mL, 1.89 mmol, 1.4 equiv), 2.7 M solution in Toluene (1.1 mL, 2.97 mmol, 2.2 equiv), **compound 4** was obtained in 92 %yield (249 mg) as colorless oil without any further purification.

^{1}H NMR (400 MHz, $CDCl_3$) δ: 7.43-7.38 (m, 2H, Ph H-3,5), 7.37-7.32 (m, 3H, Ph H-2,4,6), 3.83 (d, 2H, $^3J_{H,H}$ = 19.7 Hz, CH_2Cl), 2.26-1.84 (m, 2H, CH_2CH_3), 1.48-1.09 (m, 2H, CH_2CH_3), 0.91 (t, 3H, $^3J_{H,H}$ = 7.4 Hz, CH_3).
^{13}C NMR (100 MHz, $CDCl_3$) δ: 140.1 (d, 1C, $^2J_{C,F}$ = 21.9 Hz, Ph C-1), 128.4 (d, 2C, $^4J_{C,F}$ = 1.8 Hz, Ph C-3,5), 127.9 (d, 1C, $^5J_{C,F}$ = 1.1 Hz, Ph C-4), 124.7 (d, 2C, $^3J_{C,F}$ = 10.0 Hz, Ph C-2,6), 97.7 (d, 1C, $^1J_{C,F}$ = 181.5 Hz, CHF), 50.9 (d, 1C, $^2J_{C,F}$ = 27.5 Hz, CH_2Cl), 39.3 (d, 1C, $^2J_{C,F}$ = 22.7 Hz, CH_2CH_3), 16.4 (d, 1C, $^3J_{C,F}$ = 3.6 Hz, CH_2CH_3), 14.1 (1C, CH_3).
^{19}F NMR (470 MHz, $CDCl_3$) δ: -162.0 (m, 1F, F-1).
HRMS (ESI), *m/z*: calcd. for $C_{11}H_{14}ClFNa^+$: 223.0666 $[M+Na]^+$; found:223.0669.

Compound 5
1-(1-chloro-2-fluoro-2-propanyl)-4-fluoro benzene

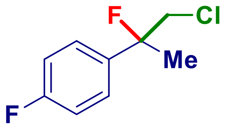

By following the **General procedure 1**, starting from 4-fluoroacetophenone (200 mg, 1.45 mmol, 1.0 equiv) in dry THF (3 mL), chloroiodomethane (0.16 mL, 2.2 mmol, 1.5 equiv), MeLi-LiBr 2.2 M solution in Et_2O (0.92 mL, 2.03 mmol, 1.4 equiv), 2.7 M solution in Toluene (1.18 mL, 3.19 mmol, 2.2 equiv), **compound 5** was obtained in 85% yield (235 mg) as colorless oil without any further purification.

^{1}H NMR (400 MHz, $CDCl_3$) δ: 7.38-7.35 (m, 2H, Ph H-2,6), 7.10-7.06 (m, 2H, Ph H-3,5), 3.82-3.68 (m, 2H, CH_2Cl), 1.80 (d, 3H, $^3J_{H,F}$ = 22.1 Hz, CH_3).
^{13}C NMR (100 MHz, $CDCl_3$) δ: 162.5 (dd, 1C, $^1J_{C,F}$ = 247.2 Hz, $^5J_{C,F}$ = 1.7 Hz, Ph C-4), 137.2 (dd, 1C, $^2J_{C,F}$ = 22.2 Hz, $^4J_{C,F}$ = 3.2 Hz, Ph C-1), 126.4 (t, 2C, $^3J_{C,F}$ = 8.6 Hz, Ph C-2,6), 115.4 (dd, 2C, $^2J_{C,F}$ = 22.5 Hz, $^4J_{C,F}$ = 1.1 Hz, Ph C-3,5), 95.3 (d, 1C, $^1J_{C,F}$ = 178.4 Hz, CF), 51.4 (d, 1C, $^2J_{C,F}$ = 29.0 Hz, CH_2Cl), 24.4 (d, 1C, $^2J_{C,F}$ = 24.2 Hz, CH_3).
^{19}F NMR (470 MHz, $CDCl_3$) δ: -148.7 (m, 1F, F-1), -113.9 (m, 1F, Ph F-4).
HRMS (ESI), *m/z*: calcd. for $C_9H_9ClF_2Na^+$: 213.0253 $[M+Na]^+$; found:213.0257.

Compound 6
1-(1-chloro-2-fluoro-2-propanyl) -2,4,5-trifluorobenzene

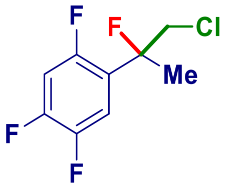

By following the **General procedure 1**, starting from 2,4,5-trifluoroacetophenone (200 mg, 1.15 mmol, 1.0 equiv) in dry THF (3 mL), chloroiodomethane (0.12 mL, 1,72 mmol, 1.5 equiv), MeLi-LiBr 2.2 M solution in Et_2O (0.73 mL, 1.6 mmol, 1.4 equiv), Deoxo-Fluor 2.7 M solution in Toluene (0.9 mL, 2.5 mmol, 2.2 equiv), **compound 6** was obtained in 82% yield (214 mg) as colorless oil without any further purification.

^{1}H NMR (400 MHz, C_6D_6) δ: 7.18-7.12 (m, 1H, Ph H-6), 6.29-6.22 (m, 1H, Ph H-3), 3.52-3.36 (m, 2H, CH_2Cl), 1.35 (dd, 3H, $^3J_{H,F}$ = 22.5 Hz, $^4J_{H,H}$ = 1.4 Hz, CH_3).
^{13}C NMR (100 MHz, C_6D_6) δ: 153.2, 150.0, 147.2 (dddd, 3C, Ph C-2,4,5), 125.4 (m, 1C, Ph C-1), 115.8 (dddd, 1C, $^2J_{C,F}$ = 21.4 Hz, $^3J_{C,F}$ = 16.6 Hz, $^3J_{C,F}$ = 5.5 Hz, $^3J_{C,F}$ = 1.4 Hz, Ph C-6), 106.2 (m, 1C, Ph C-3), 93.8 (ddt, 1C, $^1J_{C,F}$ = 183.1 Hz, $^3J_{C,F}$ = 4.9 Hz, $^3J_{C,F}$ = 0.6 Hz, CF), 49.2 (ddd, 1C, $^2J_{C,F}$ = 25.1 Hz, $^4J_{C,F}$ = 5.0 Hz, $^4J_{C,F}$ = 1.0 Hz, CH_2Cl), 23.6 (ddd, 1C, $^2J_{C,F}$ = 24.2 Hz, $^4J_{C,F}$ = 3.8 Hz, $^3J_{C,F}$ = 0.8 Hz, CH_3).
^{19}F NMR (470 MHz, C_6D_6) δ: -147.9, -141.6, -133,7, -117.2 (m, 4F, F-1, Ph F-2,4,5).
HRMS (ESI), *m/z*: calcd. for $C_9H_7ClF_4Na^+$: 249.0065 $[M+Na]^+$; found:249.0071.

Compound 7
1-(1-chloro-2-fluoro-2-propanyl)-4-(trifluoromethyl) benzene

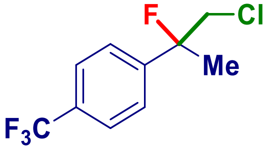

By following the **General procedure 1**, starting from 4-(trifluoromethyl)acetophenone (200 mg, 1.06 mmol, 1.0 equiv) in dry THF (3 mL), chloroiodomethane (0.12 mL, 1.59 mmol, 1.5 equiv),

MeLi-LiBr 2.2 M solution in Et_2O (0.67 mL, 1.5 mmol, 1.4 equiv), Deoxo-Fluor 2.7 M solution in Toluene (0.87 mL, 2.3 mmol, 2.2 equiv), **compound 7** was obtained in 90% yield (230 mg) as colorless oil without any further purification.

^{1}H NMR (400 MHz, $CDCl_3$) δ: 7.68-7.66 (m, 2H, Ph H-3,5), 7.65-7.50 (m, 2H, Ph H-2,6), 3.85-3.74 (m, 2H, CH_2Cl), 1.81 (d, 3H, $^3J_{H,F}$ = 24.3 Hz, CH_3).
^{13}C NMR (100 MHz, $CDCl_3$) δ: 145.2 (dq, 1C, $^2J_{C,F}$ = 22.1 Hz, $^5J_{C,F}$ = 1.3 Hz, Ph C-1), 130.5 (dq, 1C, $^2J_{C,F}$ = 32.7 Hz, $^5J_{C,F}$ = 1.2 Hz, Ph C-4), 125.5 (dq, 2C, $^3J_{C,F}$ = 3.8 Hz, $^4J_{C,F}$ = 1.2 Hz, Ph C-3,5), 125.0 (d, 2C, $^3J_{C,F}$ = 9.5 Hz, Ph C-2,6), 123.9 (q, 1C, $^1J_{C,F}$ = 272.2 Hz, CF_3), 95.3 (d, 1C, $^1J_{C,F}$ = 180.0 Hz, CF), 51.0 (dq, 1C, $^2J_{C,F}$ = 28.3 Hz, $^7J_{C,F}$ = 0.5 Hz, CH_2Cl), 24.6 (d, 1C, $^2J_{C,F}$ = 24.3 Hz, CH_3).
^{19}F NMR (470 MHz, $CDCl_3$) δ: -150.7 (m, 1F, F-1), -62.7 (m, 1F, CF_3).
HRMS (ESI), *m/z*: calcd. for $C_{10}H_9ClF_4Na^+$: 263.0221 [M+Na]$^+$; found:263.0225.

Compound 8
1-(1-chloro-2-fluoro-2-propanyl)-4-nitrobenzene

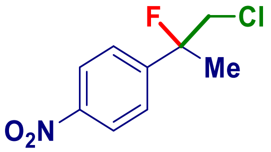

By following the **General procedure 1**, starting from 4-nitroacetophenone (200 mg, 1.2 mmol, 1.0 equiv) in dry THF (3 mL), chloroiodomethane (0.13 mL, 1.8 mmol, 1.5 equiv), MeLi-LiBr 2.2 M solution in Et_2O (0.76 mL, 1.7 mmol, 1.4 equiv), Deoxo-Fluor 2.7 M solution in Toluene (0.98 mL, 2.64 mmol, 2.2 equiv), **compound 8** was obtained in 86% yield (225 mg) as colorless oil without any further purification.

^{1}H NMR (400 MHz, C_6D_6) δ: 7.75-7.72 (m, 2H, Ph H-3,5), 6.76-6.74 (m, 2H, Ph H-2,6), 3.17-3.06 (m, 2H, CH_2Cl), 1.20 (d, 3H, $^3J_{H,F}$ = 21.9 Hz, CH_3).
^{13}C NMR (100 MHz, C_6D_6) δ: 147.9 (1C, Ph C-4), 147.7 (d, 1C, $^2J_{C,F}$ = 22.0 Hz, Ph C-1), 125.5 (d, 2C, $^3J_{C,F}$ = 9.5 Hz, Ph C-2,6), 123.6 (d, 2C, $^4J_{C,F}$ = 1.6 Hz, Ph C-3,5), 95.1 (d, 1C, $^1J_{C,F}$ = 181.5 Hz, CF), 50.5 (d, 1C, $^2J_{C,F}$ = 27.6 Hz, CH_2Cl), 24.3 (d, 1C, $^2J_{C,F}$ = 24.2 Hz, CH_3).
^{19}F NMR (470 MHz, C_6D_6) δ: -151.2 (m, 1F, F-1).
HRMS (ESI), *m/z*: calcd. for $C_9H_9ClFNO_2Na^+$: 240.0204 [M+Na]$^+$; found:240.0208.

Compound 9
1-bromo-4-(1-chloro-2-fluoro-2-propanyl) benzene

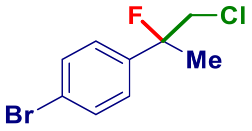

By following the **General procedure 1**, starting from 4-bromoacetophenone (200 mg, 1.0 mmol, 1.0 equiv) in dry THF (3 mL), chloroiodomethane (0.11 mL, 1.5 mmol, 1.5 equiv), MeLi-LiBr 2.2 M solution in Et_2O (0.64 mL, 1.4 mmol, 1.4 equiv), Deoxo-Fluor 2.7 M solution in Toluene (0.8 mL, 2.2 mmol, 2.2 equiv), **compound 9** was obtained in 92% yield (231 mg) as colorless oil without any further purification.

^{1}**H NMR** (400 MHz, $CDCl_3$) δ: 7.54-7.51 (m, 2H, Ph H-2,6), 7.28-7.25 (m, 2H, Ph H-3,5), 3.78 (dd, 1H, $^{3}J_{H,F}$ = 17.0 Hz, $^{2}J_{H,F}$ = 12.1 Hz, CH_2Cl), 3.73 (dd, 1H, $^{3}J_{H,F}$ = 20.5 Hz, $^{2}J_{H,F}$ = 12.1 Hz, CH_2Cl), 1.78 (d, 3H, $^{3}J_{H,F}$ = 22.1 Hz, CH_3).
^{13}**C NMR** (100 MHz, $CDCl_3$) δ: 140.4 (d, 1C, $^{2}J_{C,F}$ = 22.2 Hz, Ph C-4), 131.6 (d, 2C, $^{4}J_{C,F}$ = 1.2 Hz, Ph C-2,6), 126.3 (d, 2C, $^{3}J_{C,F}$ = 9.2 Hz, Ph C-3,5), 122.4 (d, 1C, $J_{C,F}$ = 1.7 Hz, Ph C-1), 95.3 (d, 1C, $^{1}J_{C,F}$ = 179.1 Hz, CF), 51.1 (d, 1C, $^{2}J_{C,F}$ = 28.7 Hz, CH_2Cl), 24.3 (d, 1C, $^{2}J_{C,F}$ = 24.3 Hz, CH_3).
^{19}**F NMR** (470 MHz, $CDCl_3$) δ: -150.0 (ddq, 1F, $^{2}J_{H,F}$ = 22.1 Hz, $^{3}J_{H,F}$ = 20.5 Hz, $^{3}J_{H,F}$ = 17.0 Hz, F-1).
HRMS (ESI), *m/z*: calcd. for $C_9H_9BrClFNa^+$: 272.9458 $[M+Na]^+$; found:272.9461.

Compound 10
(1-chloro-2-fluoro-2-propanyl) benzene[1]

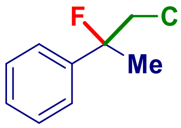

By following the **General procedure 1**, starting from acetophenone (200 mg, 1.7 mmol, 1.0 equiv) in dry THF (3 mL), chloroiodomethane (0.18 mL, 2.5mmol, 1.5 equiv), MeLi-LiBr 2.2 M solution in Et_2O (1.1 mL, 2.38 mmol, 1.4 equiv), Deoxo-Fluor 2.7 M solution in Toluene (1.4 mL, 3.7 mmol, 2.2 equiv), **compound 10** was obtained in 91% yield (266 mg) as colorless oil without any further purification.

^{1}**H NMR** (400 MHz, $CDCl_3$) δ: 7.43-7.37 (m, 4H, Ph H-2,3,5,6), 7.36-7.33 (m, 1H, Ph H-4), 3.86-3.71 (m, 2H, CH_2Cl), 1.81 (d, 3H, $^{3}J_{H,F}$ = 22.2 Hz, CH_3).
^{13}**C NMR** (100 MHz, $CDCl_3$) δ: 141.4 (d, 1C, $^{2}J_{C,F}$ = 21.7 Hz, Ph C-1), 128.5 (d, 2C, $^{4}J_{C,F}$ = 1.4 Hz, Ph C-3,5), 128.2 (d, 1C, $^{5}J_{C,F}$ = 1.3 Hz, Ph C-4), 124.4 (d, 2C, $^{3}J_{C,F}$ = 9.1 Hz, Ph C-2,6), 95.5 (d, 1C, $^{1}J_{C,F}$ = 178.5 Hz, CF), 51.6 (d, 1C, $^{2}J_{C,F}$ = 28.4 Hz, CH_2Cl), 24.3 (d, 1C, $^{2}J_{C,F}$ = 24.4 Hz, CH_3).
^{19}**F NMR** (470 MHz, $CDCl_3$) δ: -150.4 (m, 1F, F-1).
HRMS (ESI), *m/z*: calcd. for $C_9H_{10}ClFNa^+$: 195.0347 $[M+Na]^+$; found:195.0350.

Compound 11
(3-chloro-1,1,1,2-tetrafluoro-2-propanyl) benzene

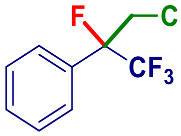

By following the **General procedure 1**, starting from 2,2,2-trifluoroacetophenone (200 mg, 1.15 mmol, 1.0 equiv) in dry THF (3 mL), chloroiodomethane (0.13 mL, 1.7 mmol, 1.5 equiv, MeLi-LiBr 2.2 M solution in Et_2O (0.73mL, 1.6 mmol, 1.4 equiv), Deoxo-Fluor 2.7 M solution in Toluene (0.94 mL, 2.5 mmol, 2.2 equiv), **compound 11** was obtained in 89% yield (232 mg) as colorless oil without any further purification.

^{1}**H NMR** (400 MHz, C_6D_6) δ: 7.20-7.15 (m, 2H, Ph H-2,6), 7.02-6.98 (m, 3H, Ph H-3,4,5), 3.69-3.50 (m, 1H, CH_2Cl), 3.47-3.39 (ddq, 1H, $^{3}J_{H,F}$ = 30.9 Hz, $^{2}J_{H,H}$ = 12.9 Hz, $^{4}J_{H,F}$ = 0.9 Hz, CH_2Cl).
^{13}**C NMR** (100 MHz, C_6D_6) δ: 131.6 (d, 1C, $^{2}J_{C,F}$ = 21.4 Hz, Ph C-1), 129.9(d, 1C, $^{5}J_{C,F}$ = 1.1 Hz, Ph C-4), 128.8 (d, 2C, $^{4}J_{C,F}$ = 2.0 Hz, Ph C-3,5), 126.0 (dq, 2C, $^{3}J_{C,F}$ = 9.8 Hz, $^{4}J_{C,F}$ = 1.1 Hz, Ph C-2,6), 123.0

(dq, 1C, $^1J_{C,F}$ = 286.1 Hz, $^2J_{C,F}$ = 29.9 Hz, CF_3), 94.8 (dq, 1C, $^1J_{C,F}$ = 193.6.0 Hz, $^2J_{C,F}$ = 30.6 Hz, CF), 43.5 (dq, 1C, $^2J_{C,F}$ = 22.5 Hz, $^3J_{C,F}$ = 1.3 Hz, CH_2Cl).
^{19}F NMR (470 MHz, C_6D_6) δ: -175.0 (m, 1F, F-1), -78.2 (d, 1F, $^3J_{F,F}$ = 7.3 Hz, CF_3).
HRMS (ESI), *m/z*: calcd. for $C_9H_7ClF_4Na^+$: 249.0065 [M+Na]$^+$; found:249.0067.

Compound 12
1,1'-(2-chloro-1-fluoro-1,1-ethanediyl) dibenzene

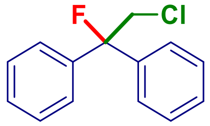

By following the **General procedure 1**, starting from benzophenone (200 mg, 1.1 mmol, 1.0 equiv) in dry THF (3 mL), chloroiodomethane (0.12 mL, 1.65 mmol, 1.5 equiv), MeLi-LiBr 2.2 M solution in Et_2O (0.73 mL, 1.6 mmol, 1.4 equiv), Deoxo-Fluor 2.7 M solution in Toluene (0.9 mL, 2.4 mmol, 2.2 equiv), **compound 12** was obtained in 93% yield (240 mg) as colorless oil after column chromatography on neutral alumina grade IV (*n*-hexane/ diethyl ether 9:1 as eluent).

^{1}H NMR (400 MHz, $CDCl_3$) δ: 7.41-7.37 (m, 4H, Ph H-2,2,6,6), 7.36-7.33 (m, 4H, Ph H-3,3,5,5), 7.36-7.33 (m, 2H, Ph H-4,4), 4.24 (d, 2H, $^3J_{H,F}$ = 21.0 Hz, CH_2Cl).
^{13}C NMR (100 MHz, $CDCl_3$) δ: 140.4 (d, 2C, $^2J_{C,F}$ = 23.2 Hz, ph C-1,1), 128.5 (m, 2C, Ph C-4,4), 128.4 (d, 4C, $^4J_{C,F}$ = 0.8 Hz, ph C-3,3,5,5), 125.8 (d, 4C, $^3J_{C,F}$ = 7.8 Hz, ph C-2,2,6,6), 97.7 (d, 1C, $^1J_{C,F}$ = 182.4 Hz, CHF), 49.4 (d, 1C, $^2J_{C,F}$ = 25.6 Hz, CH_2Cl).
^{19}F NMR (470 MHz, $CDCl_3$) δ: -150.0 (t, $^3J_{H,F}$ = 21.0 Hz, 1F, F-1).
HRMS (ESI), *m/z*: calcd. for $C_{14}H_{12}ClFNa^+$: 257.0509 [M+Na]$^+$; found:257.0511.

Compound 13
(1,2-chloro-1-fluoro-1-phenylethyl) fluorobenzene

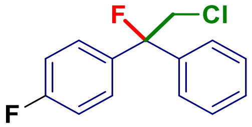

By following the **General procedure 1**, starting from 4-fluorobenzophenone (200 mg, 1.0 mmol, 1.0 equiv) in dry THF (3 mL), chloroiodomethane (0.11 mL, 1.5 mmol, 1.5 equiv), MeLi-LiBr 2.2 M solution in Et_2O (0.64 mL, 1.4 mmol, 1.4 equiv), Deoxo-Fluor 2.7 M solution in Toluene (0.8 mL, 2.2 mmol, 2.2 equiv), **compound 13** was obtained in 89% yield (225 mg) as colorless oil after column chromatography on neutral alumina grade IV (*n*-hexane/ diethyl ether 95:5 as eluent).

^{1}H NMR (400 MHz, $CDCl_3$) δ: 7.43-7.35 (m, 5H, Ph1 H-2,3,4,5,6), 7.40-7.35 (m, 2H, Ph2 H-2,6), 7.10-7.05 (m, 2H, Ph2 H-3,5), 4.21 (d, 2H, $^3J_{H,F}$ = 20.7 Hz, CH_2F).
^{13}C NMR (100 MHz, $CDCl_3$) δ: 162.6 (dd, 1C, $^1J_{C,F}$ = 248.1 Hz, $^5J_{C,F}$ = 2.0 Hz, ph2 C-4), 140.0 (d, 1C, $^3J_{C,F}$ = 23.2 Hz, Ph1 C-1), 136.0 (dd, 1C, $^2J_{C,F}$ = 23.7 Hz, $^4J_{C,F}$ = 3.3 Hz, ph2 C-1), 128.6 (d, 1C, $^5J_{C,F}$ = 1.6 Hz, ph1 C-4), 128.5 (2C, Ph1 C-3,5), 128.0 (t, 2C, $^3J_{C,F}$ = 8.0 Hz, Ph2 C-2,6), 125.8 (d, 2C, $^3J_{C,F}$ = 7.6 Hz, Ph1 C-2,6), 115.4 (d, 2C, $^2J_{C,F}$ = 21.7 Hz, Ph2 C-3,5), 97.5 (d, 1C, $^1J_{C,F}$ = 182.4 Hz, CHF), 49.3 (d, 1C, $^2J_{C,F}$ = 27.0 Hz, CH_2F).
^{19}F NMR (470 MHz, $CDCl_3$) δ: -147.9 (t, $^3J_{H,F}$ = 20.7 Hz, 1F, F-1), -113.3 (m, 1F, F-2).
HRMS (ESI), *m/z*: calcd. for $C_{14}H_{11}ClFNa^+$: 275.0415 [M+Na]$^+$; found:275.0418.

Compound 14
1-(2-chloro-1-fluoro-1-phenylethyl)-4-methoxybenzene

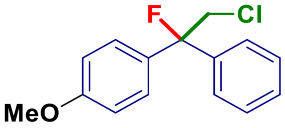

By following the **General procedure 1**, starting from 4-methoxybenzophenone (200 mg, 0.94 mmol, 1.0 equiv) in dry THF (3 mL), chloroiodomethane (0.1 mL, 1.4 mmol, 1.5 equiv), MeLi-LiBr 2.2 M solution in Et_2O (0.6 mL, 1.3 mmol, 1.4 equiv), Deoxo-Fluor 2.7 M solution in Toluene (0.77 mL, 2.07 mmol, 2.2 equiv), **compound 14** was obtained in 79% yield (197 mg) as colorless oil after column chromatography on neutral alumina grade IV (*n*-hexane/diethyl ether 8:2 as eluent).

^{1}H NMR (400 MHz, $CDCl_3$) δ: 7.40-7.38 (m, 4H, Ph1 H-2,3,5,6), 7.37-7.34 (m, 1H, Ph1 H-4), 7.33-7.30 (m, 2H, Ph2 H-2,6), 6.92-6.90 (m, 2H, Ph2 H-3,5), 4.25-4.19 (m, 2H, CH_2Cl), 3.81 (s, 3H, OCH_3).
^{13}C NMR (100 MHz, $CDCl_3$) δ: 159.6 (d, 1C, $^5J_{C,F}$ = 1.8 Hz, Ph2 C-4), 140.4 (d, 1C, $^2J_{C,F}$ = 23.3 Hz, Ph1 C-1), 132.3 (d, 1C, $^3J_{C,F}$ = 23.7 Hz, Ph2 C-1), 128.4 (d, 2C, $^4J_{C,F}$ = 0.6 Hz, Ph1 C-3,5), 128.3 (1C, Ph1 C-4), 127.4 (d, 4C, $^3J_{C,F}$ = 7.2 Hz, Ph2 C-2,6, $^3J_{C,F}$ = 7.1 Hz, Ph1 C-2,6), 113.7 (2C, Ph2 C-3,5), 97.7 (d, 1C, $^1J_{C,F}$ = 181.5 Hz, CHF), 55.2 (1C, OCH_3), 49.5 (d, 1C, $^2J_{C,F}$ = 27.2 Hz, CH_2Cl).
^{19}F NMR (470 MHz, $CDCl_3$) δ: -147.3 (t, $^3J_{H,F}$= 20.7 Hz, 1F, F-1).
HRMS (ESI), *m/z*: calcd. for $C_{15}H_{14}ClFONa^+$: 287.0615 $[M+Na]^+$; found:287.0619.

Compound 15
1-(2-chloro-1-fluoro-1-phenylethyl)-4-(methylsulfanyl) benzene

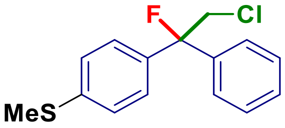

By following the **General procedure 1**, starting from 4-(methylsulfanyl) benzophenone (200 mg, 0.9 mmol, 1.0 equiv) in dry THF (3 mL), chloroiodomethane (0.1 mL, 1.4 mmol, 1.5 equiv), MeLi-LiBr 2.2 M solution in Et_2O (0.6 mL, 1.3 mmol, 1.4 equiv), Deoxo-Fluor 2.7 M solution in Toluene (0.7 mL, 1.98 mmol, 2.2 equiv), **compound 15** was obtained in 90% yield (227 mg) as colorless oil after column chromatography on neutral alumina grade IV (*n*-hexane/ diethyl ether 8:2 as eluent).

^{1}H NMR (400 MHz, $CDCl_3$) δ: 7.39-7.33 (m, 5H, Ph1 H-2,3,4,5,6), 7.30-7.29 (m, 2H, Ph2 H-2,6), 7.25-7.23 (m, 2H, Ph2 H-3,5), 4.21 (d, 2H, $^3J_{H,H}$ = 20.8 Hz, CH_2F), 2.48 (s, 3H, SCH_3).
^{13}C NMR (100 MHz, $CDCl_3$) δ: 140.1 (d, 1C, $^2J_{C,F}$ = 23.2 Hz, Ph1 C-1), 139.3 (d, 1C, $^5J_{C,F}$ = 1.8 Hz, Ph2 C-4), 136.8 (d, 1C, $^2J_{C,F}$ = 23.6 Hz, $^3J_{C,F}$ = 2.7 Hz, Ph2 C-1), 128.5 (d, 1C, $^5J_{C,F}$ = 1.7 Hz, Ph1 C-4), 128.5 (2C, Ph1 C-3,5), 126.4 (d, 2C, $^3J_{C,F}$ = 7.5 Hz, Ph2 C-2,6), 126.1 (2C, Ph2 C-3,5), 125.8 (d, 2C, $^3J_{C,F}$ = 7.6 Hz, Ph1 C-2,6), 97.6 (d, 1C, $^1J_{C,F}$ = 182.2 Hz, CHF), 49.3 (d, 1C, $^2J_{C,F}$ = 27.0 Hz, CH_2Cl), 15.4 (1C, SCH_3).
^{19}F NMR (470 MHz, $CDCl_3$) δ: -149.1 (t, $^3J_{H,F}$ = 20.8 Hz, 1F, F-1).
HRMS (ESI), *m/z*: calcd. for $C_{15}H_{14}ClFSNa^+$: 303.0381 $[M+Na]^+$; found:303.0385.

Compound 16
1,1- (1,2-difluoro-1,1-ethanediyl)dibenzene[2]

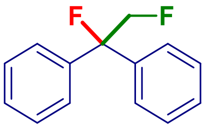

By following the **General procedure 1**, starting from benzophenone (200 mg, 1.1 mmol, 1.0 equiv) in dry THF (3 mL), chloroiodomethane (0.12 mL, 1.65 mmol, 1.5 equiv), MeLi-LiBr 2.2 M solution in Et_2O (0.73 mL, 1.6 mmol, 1.4 equiv), Deoxo-Fluor 2.7 M solution in Toluene (0.9 mL, 2.42 mmol, 2.2 equiv), **compound 16** was obtained in 85% yield (204 mg) as colorless oil after column chromatography on neutral alumina grade IV (*n*-hexane as eluent).

^{1}H NMR (400 MHz, $CDCl_3$) δ: 7.42-7.38 (m, 4H, Ph H-2,2,6,6), 7.37-7.34 (m, 4H, Ph H-3,3,5,5), 7.38-7.36 (m, 2H, Ph H-4,4), 4.98 (dd, 2H, $^2J_{H,F}$ = 47.5 Hz, $^2J_{H,H}$ = 21.6 Hz, CH_2F).
^{13}C NMR (100 MHz, $CDCl_3$) δ: 139.0 (dd, 2C, $^2J_{C,F}$ = 22.9 Hz, $^3J_{C,F}$ = 2.7 Hz, ph C-1,1), 128.6 (d, 2C, $^5J_{C,F}$ = 1.8 Hz, Ph C-4,4), 128.4 (4C, Ph C-3,3,5,5), 126.2 (dd, 4C, $^3J_{C,F}$ = 7.3 Hz, $^4J_{C,F}$ = 1.0 Hz, Ph C-2,2,6,6), 97.7 (dd, 1C, $^1J_{C,F}$ = 179.0 Hz, $^2J_{C,F}$ = 18.5 Hz, CHF), 85.1 (dd, 1C, $^1J_{C,F}$ = 185.5 Hz, $^2J_{C,F}$ = 26.6 Hz, CH_2F).
^{19}F NMR (470 MHz, $CDCl_3$) δ: -221.2 (dt, $^2J_{H,F}$ = 47.5 Hz, $^1J_{C,F}$ = 18.0 Hz, 1F, F-2), -153.0 (m, 1F, F-1).
HRMS (ESI), *m/z*: calcd. for $C_{14}H_{12}F_2Na^+$: 241.0805 [M+Na]$^+$; found:241.0809.

Compound 17
1,1'- (1,2,2-trifluoro-1,1-ethanediyl)dibenzene[3]

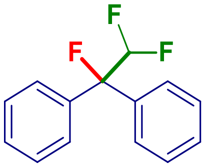

By following the **General procedure 3**, starting from benzophenone (200 mg, 1.1 mmol, 1.0 equiv) in dry THF (3 mL), $TMSCHF_2$ (0.27 mL, 2.2 mmol, 2 equiv), potassium *tert*-pentoxide 0.9 M (2.2 ml, 2.0 mmol, 1.8 equiv), Deoxo-Fluor 2.7 M solution in Toluene (0.9 mL, 2.42 mmol, 2.2 equiv), **compound 17** was obtained in 88% yield (229 mg) as colorless oil without any further purification.

^{1}H NMR (400 MHz, $CDCl_3$) δ: 7.35-7.26 (m, 10H, Ph H-2,2,3,3,4,4,5,5,6,6), 6.16 (dt, 1H, $^2J_{H,F}$ = 54.4 Hz, $^3J_{H,F}$ = 5.5 Hz, CHF_2).
^{13}C NMR (100 MHz, $CDCl_3$) δ: 136.7 (dt, 2C, $^2J_{C,F}$ = 22.2 Hz, $^3J_{C,F}$ = 1.0 Hz, ph C-1,1), 129.1 (d, 2C, $^5J_{C,F}$ = 1.9 Hz, Ph C-4,4), 128.4 (4C, Ph C-3,3,5,5), 126.8 (dt, 4C, $^3J_{C,F}$ = 7.4 Hz, $^4J_{C,F}$ = 1.5 Hz, Ph C-2,2,6,6), 114.4 (dt, 1C, $^1J_{C,F}$ = 250.7 Hz, $^2J_{C,F}$ = 36.0 Hz, CHF_2), 95.8 (dt, 1C, $^1J_{C,F}$ = 180.5 Hz, $^2J_{C,F}$ = 23.2 Hz, CHF).
^{19}F NMR (470 MHz, C_6D_6) δ: -156.0 (dt, $^3J_{F,F}$ = 11.0 Hz, $^3J_{H,F}$ = 5.8 Hz, 1F, F-1), -127.9 (dd, 1F, $^2J_{H,F}$ = 54.4 Hz, $^3J_{F,F}$ = 11.0 Hz, F-1).
HRMS (ESI), *m/z*: calcd. for $C_{14}H_{11}F_3Na^+$: 259.0712 [M+Na]$^+$; found:259.0715.

Scale up of the reaction using 20 mmol of starting material

By following the **General procedure 3**, starting from benzophenone (3645 mg, 20 mmol, 1.0 equiv) in dry THF (30 mL), $TMSCHF_2$ (5.5 mL, 40 mmol, 2 equiv), potassium *tert*-pentoxide 0.9 M (40 ml, 36 mmol, 1.8 equiv), Deoxo-Fluor 2.7 M solution in Toluene (16.3 mL, 44 mmol, 2.2 equiv), **compound 17** was obtained in 87% yield (4110 mg) as colorless oil without any further

purification. *Spectroscopic and spectrometric data match with those ones reported for the running reaction at 1.1 mmol scale.*

Compound 18

1-fluoto-4- (1,2,2,2-tetrafluoro-1-phenylethyl) benzene

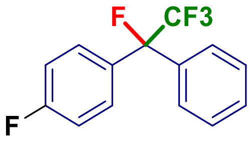

By following the **General procedure 3**, starting from 4-fluorobenzophenone (200 mg, 1.0 mmol, 1.0 equiv) in dry THF (3 mL), TMSCF$_3$ (0.3 mL, 2.0 mmol, 2.0 equiv), potassium *tert*-pentoxide 0.9 M (2.0 ml, 1.8 mmol, 1.8 equiv), Deoxo-Fluor 2.7 M solution in Toluene (0.8 mL, 2.2 mmol, 2.2 equiv), **compound 18** was obtained in 81% yield (221 mg) as colorless oil without any further purification.

^{1}H NMR (400 MHz, CDCl$_3$) δ: 7.51-7.46 (m, 2H, Ph1 H-2,6), 7.46-7.42 (m, 2H, Ph2 H-3,5), 7.47-7.43 (m, 1H, Ph1 H-4), 7.43-7.41 (m, 2H, Ph1 H-3,5), 7.13-7.07 (m, 2H, Ph2 H-2,6).
^{13}C NMR (100 MHz, CDCl$_3$) δ: 163.2 (dd, 1C, $^1J_{C,F}$ = 249.7 Hz, $^5J_{C,F}$ = 2.2 Hz, Ph2 C-1), 135.3 (d, 1C, $^2J_{C,F}$ = 22.1 Hz, Ph1 C-1), 131.5 (dd, 1C, $^2J_{C,F}$ = 22.9 Hz, $^4J_{C,F}$ = 3.4 Hz, Ph2 C-4), 129.6 (d, 1C, $^4J_{C,F}$ = 1.8 Hz, Ph1 C-4), 129.1 (m, 2C, Ph2 C-3,5), 128.4 (2C, Ph1 C-3,5), 126.7 (dq, 2C, $^3J_{C,F}$ = 7.5 Hz, $^4J_{C,F}$ = 1.6 Hz, Ph1 C-2,6), 123.1 (dq, 1C, $^1J_{C,F}$ = 285.3 Hz, $^3J_{C,F}$ = 31.0 Hz, CF$_3$), 115.4 (d, 2C, $^2J_{C,F}$ = 21.8 Hz, Ph2 C-2,5), 95.8 (dq, 1C, $^1J_{C,F}$ = 187.1 Hz, $^2J_{C,F}$ = 31.6 Hz, CHF).
^{19}F NMR (470 MHz, CDCl$_3$) δ: -153.7 (q, $^3J_{F,F}$ = 8.4 Hz, 1F, F-1), -111.6 (m, 1F, Ph F-2), -75.2 (d, $^2J_{F,F}$ = 8.4 Hz, 1F, F-3).
HRMS (ESI), *m/z*: calcd. for $C_{14}H_9F_5Na^+$: 295.0522 [M+Na]$^+$; found:295.0525.

Compound 19

2-(chloromethyl)-2-fluoroadamantane

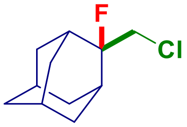

By following the **General procedure 1**, starting from adamantan-2-one (200 mg, 1.3 mmol, 1.0 equiv) in dry THF (3 mL), chloroiodomethane (0.15 mL, 2.0 mmol, 1.5 equiv), MeLi-LiBr 2.2 M solution in Et$_2$O (0.8 mL, 1.8 mmol, 1.4 equiv), Deoxo-Fluor 2.7 M solution in Toluene (1.1 mL, 2.9 mmol, 2.2 equiv), **compound 19** was obtained in 82% yield (216 mg) as colorless oil after column chromatography on neutral alumina grade IV (*n*-hexane/ ethyl acetate 8:2 as eluent).

^{1}H NMR (400 MHz, C$_6$D$_6$) δ: 4.30 (dd, 1H, $^2J_{H,H}$ = 11.9 Hz, $^3J_{H,H}$ = 27.7 Hz, CH$_2$Cl), 4.06 (dd, 1H, $^2J_{H,H}$ = 11.9 Hz, $^3J_{H,H}$ = 22.2 Hz, CH$_2$Cl), 2.23-1.35 (14H, Ad).
^{13}C NMR (100 MHz, C$_6$D$_6$) δ: 97.7 (d, 1C, $^1J_{C,F}$ = 181.8 Hz, CF), 63.9 (d, 1C, $^2J_{C,F}$ = 24.5 Hz, CH$_2$Cl), 37.6-27.2 (9C, Ad).
^{19}F NMR (470 MHz, C$_6$D$_6$) δ: -151.1 (m, 1F, F-1).
HRMS (ESI), *m/z*: calcd. for $C_{11}H_{16}ClFNa^+$: 225.0822 [M+Na]$^+$; found:225.0826.

Compound 20
1-(chloromethyl)-1-fluorocycloheptane

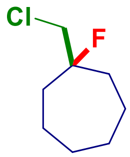

By following the **General procedure 1**, starting from cycloheptanone (200 mg, 1.8 mmol, 1.0 equiv) in dry THF (3 mL), chloroiodomethane (0.19 mL, 2.7 mmol, 1.5 equiv), MeLi-LiBr 2.2 M solution in Et_2O (1.14 mL, 2.5 mmol, 1.4 equiv), Deoxo-Fluor 2.7 M solution in Toluene (1.45 mL, 3.9 mmol, 2.2 equiv), **compound 20** was obtained in 92% yield (273 mg) as colorless oil after column chromatography on neutral alumina grade IV (*n*-hexane as eluent).

^{1}H NMR (400 MHz, $CDCl_3$) δ: 3.56 (d, 2H, $^2J_{H,H}$ = 17.9 Hz, CH_2Cl), 2.06-1.95 (m, 2H, H-2), 1.89-1.83 (m, 2H, H-7), 1.79-1.60 (m, 4H, H-3,6), 1.57-1.41 (m, 4H, H-4,5).
^{13}C NMR (100 MHz, $CDCl_3$) δ: 98.2 (d, 1C, $^1J_{C,F}$ = 175.5 Hz, CF), 51.6 (d, 1C, $^2J_{C,F}$ = 28.3 Hz, CH_2Cl), 36.7 (d, 2C, $^2J_{C,F}$ = 23.6 Hz, C-2,7), 29.7 (2C, C-4,5), 22.4 (d, 2C, $^2J_{C,F}$ = 5.4 Hz, C-2,6).
^{19}F NMR (470 MHz, $CDCl_3$) δ: -142.7 (m, 1F, F-1).
HRMS (ESI), *m/z*: calcd. for $C_8H_{14}ClFNa^+$: 187.0666 $[M+Na]^+$; found:187.0670.

Compound 21
4-(chloromethyl)-4-fluorononane

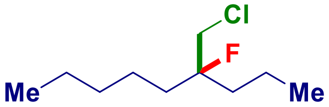

By following the **General procedure 1**, starting from nonan-4-one (200 mg, 2.1 mmol, 1.0 equiv) in dry THF (3 mL), chloroiodomethane (0.15 mL, 2.1 mmol, 1.5 equiv), MeLi-LiBr 2.2 M solution in Et_2O (0.9 mL, 1.97 mmol, 1.4 equiv), Deoxo-Fluor 2.7 M solution in Toluene (1.15 mL, 3.1 mmol, 2.2 equiv), **compound 21** was obtained in 87% yield (239 mg) as colorless oil after column chromatography on neutral alumina grade IV (*n*-hexane as eluent).

^{1}H NMR (400 MHz, $CDCl_3$) δ: 3.57 (d, 2H, $^2J_{H,H}$ = 15.6 Hz, CH_2Cl), 1.72 (dt, 4H, $^2J_{H,H}$ = 19.5 Hz, $^3J_{H,H}$ = 7.9 Hz, H-3,5), 1.35-1.32 (m, 8H, H-2,6,7,8), 0.93 (t, 6H, $^3J_{H,H}$ = 6.4 Hz, CH_3).
^{13}C NMR (100 MHz, $CDCl_3$) δ: 97.7 (d, 1C, $^1J_{C,F}$ = 175.3 Hz, CF), 47.7 (d, 1C, $^2J_{C,F}$ = 30.9 Hz, CH_2Cl), 35.0 (d, 2C, $^2J_{C,F}$ = 30.9 Hz, C-3,5), 25.3 (d, 2C, $^3J_{C,F}$ = 5.4 Hz, C-2,6), 23.1 (2C, C-7,8), 14.1 (2C, CH_3).
^{19}F NMR (470 MHz, $CDCl_3$) δ: -154.4 (m, 1F, F-1).
HRMS (ESI), *m/z*: calcd. for $C_{10}H_{20}ClFNa^+$: 217.1135 $[M+Na]^+$; found:217.1139.

Compound 22
3-(1-bromo-1-chloro-2-fluoro-2-propanyl) benzonitrile

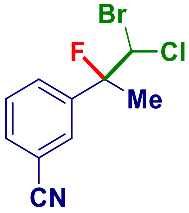

By following the **General procedure 2**, starting from 3-acetylbenzonitrile (200 mg, 1.4 mmol, 1.0 equiv) in dry THF (3 mL), bromochloromethane (0.14 mL, 2.1 mmol, 1.5 equiv), LDA 2.0 M in THF (1.0 mL, 2.0 mmol, 1.4 equiv), Deoxo-Fluor 2.7 M solution in Toluene (1.1 mL, 3.1 mmol, 2.2 equiv), **compound 22** was obtained in 80% yield (310 mg) as colorless oil without any further purification.

^{1}H NMR (400 MHz, $CDCl_3$) δ: 7.79-7.72 (m, 1H, Ph H-2), 7.73-7.71 (m, 1H, Ph H-4), 7.71-7.68 (m, 1H, Ph H-6), 7.56-7.54 (m, 1H, Ph H-5), 5.86 (d, 1H, $^3J_{H,F}$ = 10.0 Hz, CHClBr), 1.99 (d, 3H, $^3J_{H,F}$ = 22.4 Hz, CH_3).
^{13}C NMR (100 MHz, $CDCl_3$) δ: 140.3 (d, 1C, $^2J_{C,F}$ = 22.7 Hz, Ph C-3), 132.5 (d, 1C, $^5J_{C,F}$ = 1.1 Hz, Ph C-6), 130.3 (d, 1C, $^3J_{C,F}$ = 8.8 Hz, Ph C-4), 129.8 (d, 1C, $^3J_{C,F}$ = 9.6 Hz, Ph C-2), 129.1 (d, 1C, $^4J_{C,F}$ = 1.4 Hz, Ph C-5), 118.3 (1C, CN), 112.6 (d, 1C, $^4J_{C,F}$ = 1.6 Hz, Ph C-1), 96.6 (d, 1C, $^1J_{C,F}$ = 186.3 Hz, CF), 63.4 (d, 1C, $^2J_{C,F}$ = 34.5 Hz, CHClBr), 23.0 (d, 1C, $^2J_{C,F}$ = 23.2 Hz, CH_{3}).
^{19}F NMR (470 MHz, $CDCl_3$) δ: -144.0 (m, 1F, F-1).
HRMS (ESI), *m/z*: calcd. for $C_{10}H_8BrClFNNa^+$: 297.9410 $[M+Na]^+$; found:297.9412.

Compound 23
1-(2-chloro -1-fluoroethyl)-4-methylbenzene[4]

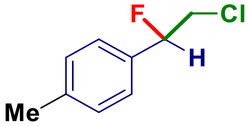

By following the **General procedure 1**, starting from 4-methylbenzaldehyde (200 mg, 1.7 mmol, 1.0 equiv) in dry THF (3 mL), chloroiodomethane (0.18 mL, 2.5 mmol, 1.5 equiv), MeLi-LiBr 2.2 M solution in Et_2O (1.1 mL, 2.4 mmol, 1.4 equiv), Deoxo-Fluor 2.7 M solution in Toluene (1.4 mL, 3.7 mmol, 2.2 equiv), **compound 23** was obtained in 93% yield (273 mg) as colorless oil without any further purification.

^{1}H NMR (400 MHz, $CDCl_3$) δ: 7.28-7.26 (m, 2H, Ph H-2,6), 7.24-7.14 (m, 2H, Ph H-3,5), 5.57 (ddd, 1H, $^2J_{H,F}$ = 47.1 Hz, $^3J_{H,H}$ = 7.8 Hz, $^3J_{H,H}$ = 3.9 Hz, CHF), 3.89-3.68 (m, 2H, CH_2Cl), 2.38 (s, 3H, CH_3).
^{13}C NMR (100 MHz, $CDCl_3$) δ: 139.2 (d, 1C, $^5J_{C,F}$ = 1.9 Hz, Ph C-4), 133.6 (d, 1C, $^2J_{C,F}$ = 20.2 Hz, Ph C-1), 129.4 (2C, Ph C-3,5), 125.8 (d, 2C, $^3J_{C,F}$ = 6.4 Hz, Ph C-2,6), 93.0 (d, 1C, $^1J_{C,F}$ = 177.6 Hz, CHF), 46.8 (d, 1C, $^2J_{C,F}$ = 28.7 Hz, CH_2Cl), 21.2 (1C, CH_3).
^{19}F NMR (470 MHz, $CDCl_3$) δ: -176.9 (m, 1F, F-1).
HRMS (ESI), *m/z*: calcd. for $C_9H_{10}ClFNa^+$: 195.0353 $[M+Na]^+$; found:195.0355.

Compound 24
1-chloro-4-(2-chloro-1-fluoroethyl) benzene[4]

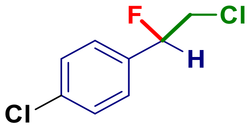

By following the **General procedure 1**, starting from 4-chlorobenzaldehyde (200 mg, 1.4 mmol, 1.0 equiv) in dry THF (3 mL), chloroiodomethane (0.15 mL, 2.1 mmol, 1.5 equiv), MeLi-LiBr 2.2 M solution in Et_2O (0.9 mL, 2.0 mmol, 1.4 equiv), Deoxo-Fluor 2.7 M solution in Toluene (1.2 mL, 3.1 mmol, 2.2 equiv), **compound 24** was obtained in 91% yield (246 mg) as colorless oil after column chromatography on neutral alumina grade IV (*n*-hexane/ethyl ether 5:5 as eluent).

^{1}H NMR (400 MHz, $CDCl_3$) δ: 7.40-7.38 (m, 2H, Ph H-2,6), 7.32-7.30 (m, 2H, Ph H-3,5), 5.58 (ddd, $^2J_{H,F}$ = 46.6 Hz, $^3J_{H,H}$ = 7.2 Hz, $^3J_{H,H}$ = 4.3 Hz, 1H, CHF), 3.86-3.68 (m, 1H, CH_2Cl).
^{13}C NMR (100 MHz, $CDCl_3$) δ: 135.2 (d, 1C, $^2J_{C,F}$ = 20.5 Hz, Ph C-1), 135.1 (d, 1C, $^2J_{C,F}$ = 20.6 Hz, Ph C-4), 128.9 (2C, Ph C-2,6), 127.2 (d, 2C, $^3J_{C,F}$ = 6.8 Hz, Ph C-3,5), 92.2 (d, $^1J_{C,F}$ = 178.9 Hz, CHF), 46.5 (d, 1C, $^2J_{C,F}$ = 28.5 Hz, CH_2Cl).
^{19}F NMR (470 MHz, $CDCl_3$) δ: -178.3 (ddd, 1F, $^2J_{H,F}$ = 46.6 Hz, $^2J_{C,F}$ = 23.5 Hz, $^3J_{H,F}$ = 16.3 Hz, F-1).
HRMS (ESI), *m/z*: calcd. for $C_8H_7Cl_2FNa^+$: 214.9807 $[M+Na]^+$; found:214.9810.

Compound 25
1,3-dichloro-2-(2-chloro -1-fluoroethyl) benzene

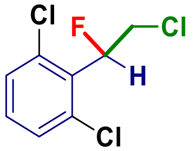

By following the **General procedure 1**, starting from 2,6-dichlorobenzaldehyde (200 mg, 1.14 mmol, 1.0 equiv) in dry THF (3 mL), chloroiodomethane (0.12 mL, 1.7 mmol, 1.5 equiv), MeLi-LiBr 2.2 M solution in Et_2O (0.7 mL, 1.6 mmol, 1.4 equiv), Deoxo-Fluor 2.7 M solution in Toluene (0.9 mL, 2.5 mmol, 2.2 equiv), **compound 25** was obtained in 79% yield (205 mg) as colorless oil after column chromatography on neutral alumina grade IV (*n*-hexane as eluent).

^{1}H NMR (400 MHz, $CDCl_3$) δ: 7.37-7.36 (m, 2H, Ph H-4,6), 7.27-7.23 (m, 1H, Ph H-5), 6.29 (ddd, 1H, $^2J_{H,F}$ = 46.0 Hz, $^3J_{H,H}$ = 8.7 Hz, $^3J_{H,H}$ = 4.8 Hz, CHF), 4.27 (m, 1H, CH_2Cl), 3.90 (ddd, 1H, $^3J_{H,F}$ = 24.1 Hz, $^2J_{H,H}$ = 11.9 Hz, $^3J_{H,H}$ = 4.8 Hz, CH_2Cl).
^{13}C NMR (100 MHz, $CDCl_3$) δ: 135.0 (d, 2C, $^3J_{C,F}$ = 3.1 Hz, Ph C-1,3), 130.9 (d, 1C, $^2J_{C,F}$ = 18.4 Hz, Ph C-2), 130.8 (d, 1C, $^5J_{C,F}$ = 1.5 Hz, Ph C-5), 129.5 (d, 2C, $^4J_{C,F}$ = 1.1 Hz, Ph C-4,6), 90.0 (d, 1C, $^1J_{C,F}$ = 182.9 Hz, CHF), 42.9 (d, 1C, $^2J_{C,F}$ = 23.2 Hz, CH_2Cl).
^{19}F NMR (470 MHz, $CDCl_3$) δ: -183.5 (dddq, 1F, $^2J_{H,F}$ = 46.0 Hz, $^3J_{H,F}$ = 34.3 Hz, $^3J_{H,F}$ = 24.1 Hz, $^2J_{C,F}$ = 10.1 Hz, $^5J_{C,F}$ = 1.1 Hz, F-1).
HRMS (ESI), *m/z*: calcd. for $C_8H_6Cl_3FNa^+$: 248.9417 $[M+Na]^+$; found:248.9420.

Compound 26
1-bromo-4-(2-chloro -1-fluoroethyl) benzene[4]

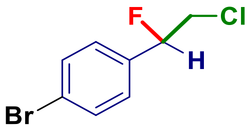

By following the **General procedure 1**, starting from 4-bromobenzaldehyde (200 mg, 1.1 mmol, 1.0 equiv) in dry THF (3 mL), chloroiodomethane (0.12 mL, 1.6 mmol, 1.5 equiv), MeLi-LiBr 2.2 M solution in Et_2O (0.7 mL, 1.5 mmol, 1.4 equiv), Deoxo-Fluor 2.7 M solution in Toluene (0.9 mL, 2.2 mmol, 2.2 equiv), **compound 26** was obtained in 89% yield (233 mg) as colorless oil after column chromatography on neutral alumina grade IV (*n*-hexane/ethyl ether 9:1 as eluent).

^{1}H NMR (400 MHz, $CDCl_3$) δ: 7.56-7.53 (m, 2H, Ph H-2,6), 7.25-7.23 (m, 2H, Ph H-3,5), 5.57 (ddd, 1H, $^2J_{H,F}$ = 46.6 Hz, $^3J_{H,H}$ = 7.1 Hz, $^3J_{H,H}$ = 4.3 Hz, CHF), 3.85-3.68 (m, 2H, CH_2Cl).
^{13}C NMR (100 MHz, $CDCl_3$) δ: 135.6 (d, 1C, $^2J_{C,F}$ = 20.5 Hz, Ph C-4), 131.8 (2C, Ph C-2,6), 127.4 (d, 2C, $^3J_{C,F}$ = 6.8 Hz, Ph C-3,5), 123.3 (d, 1C, $^5J_{C,F}$ = 2.2 Hz, Ph C-1), 91.3 (d, 1C, $^1J_{C,F}$ = 179.1 Hz, CHF), 46.4 (d, 1C, $^2J_{C,F}$ = 28.4 Hz, CH_2Cl).
^{19}F NMR (470 MHz, $CDCl_3$) δ: -178.8 (ddd, 1F, $^2J_{H,F}$ = 46.6 Hz, $^1J_{C,F}$ = 23.4 Hz, $^2J_{C,F}$ = 16.1 Hz, F-1).
HRMS (ESI), *m/z*: calcd. for $C_8H_7BrClFNa^+$: 258.9301 $[M+Na]^+$; found:258.9304.

Compound 27
1-(2-chloro -1-fluoroethyl)-4-fluorobenzene[4]

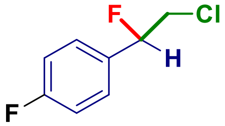

By following the **General procedure 1**, starting from 4-fluorobenzaldehyde (200 mg, 1.6 mmol, 1.0 equiv) in dry THF (3 mL), chloroiodomethane (0.18 mL, 2.4 mmol, 1.5 equiv), MeLi-LiBr 2.2 M solution in Et_2O (1.0 mL, 2.2 mmol, 1.4 equiv), Deoxo-Fluor 2.7 M solution in Toluene (1.6 mL, 3.5 mmol, 2.2 equiv), **compound 27** was obtained in 82% yield (232 mg) as colorless oil without any further purification.

^{1}H NMR (400 MHz, C_6D_6) δ: 6.70-6.68 (m, 2H, Ph H-2,6), 6.67-6.63 (m, 2H, Ph H-3,5), 5.03 (ddd, 1H, $^2J_{H,F}$ = 46.8 Hz, $^3J_{H,H}$ = 7.5 Hz, $^3J_{H,H}$ = 3.9 Hz, CHF), 3.27-3.18 (m, 1H, CH_2Cl), 3.13-3.01 (m, 1H, CH_2Cl).
^{13}C NMR (100 MHz, C_6D_6) δ: 163.3 (dd, 1C, $^1J_{C,F}$ = 247.5 Hz, $^5J_{C,F}$ = 1.9 Hz, Ph C-4), 132.8 (dd, 1C, $^2J_{C,F}$ = 20.7 Hz, $^4J_{C,F}$ = 3.3 Hz, Ph C-1), 128.0 (m, 2C, Ph C-2,6), 115.6 (d, 2C, $^2J_{C,F}$ = 21.7 Hz, Ph C-3,5), 92.2 (d, 1C, $^1J_{C,F}$ = 178.9 Hz, CHF), 46.6 (d, 1C, $^2J_{C,F}$ = 28.2 Hz, CH_2Cl).
^{19}F NMR (470 MHz, C_6D_6) δ: -177.0 (m, 1F, F-1), -112.4 (m, 1F, F-4).
HRMS (ESI), *m/z*: calcd. for $C_8H_7ClF_2Na^+$: 199.0102 $[M+Na]^+$; found:199.0107.

Compound 28
1-(2-chloro -1-fluoroethyl)-4-(trifluoromethyl) benzene

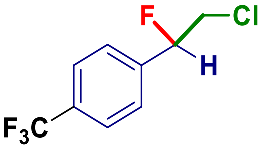

By following the **General procedure 1**, starting from 4-(trifluoromethyl)benzaldehyde (200 mg, 1.15 mmol, 1.0 equiv) in dry THF (3 mL), chloroiodomethane (0.12 mL, 1.7 mmol, 1.5 equiv), MeLi-LiBr 2.2 M solution in Et_2O (0.7 mL, 1.6 mmol, 1.4 equiv), Deoxo-Fluor 2.7 M solution in

Toluene (0.94 mL, 2.5 mmol, 2.2 equiv), **compound 28** was obtained in 80% yield (208 mg) as colorless oil after column chromatography on neutral alumina grade IV (*n*-hexane as eluent).

^{1}H NMR (400 MHz, $CDCl_3$) δ: 7.70-7.68 (m, 2H, Ph H-3,5), 7.51-7.49 (m, 2H, Ph H-2,6), 5.68 (ddd, 1H, $^2J_{H,F}$ = 46.5 Hz, $^3J_{H,H}$ = 6.6 Hz, $^3J_{H,H}$ = 4.7 Hz, CHF), 3.88-3.74 (m, 2H, CH_2Cl).
^{13}C NMR (100 MHz, $CDCl_3$) δ: 140.4 (d, 1C, $^2J_{C,F}$ = 20.5 Hz, Ph C-1), 131.4 (q, 1C, $^2J_{C,F}$ = 32.0 Hz, Ph C-4), 126.1 (d, 2C, $^3J_{C,F}$ = 7.2 Hz, Ph C-2,6), 125.7 (d, 2C, $^4J_{C,F}$ = 3.7 Hz, Ph C-3,5), 123.8 (q, 1C, $^1J_{C,F}$ = 272.2 Hz, CF_3), 92.0 (d, 1C, $^1J_{C,F}$ = 180.1 Hz, CHF), 46.4 (d, 1C, $^2J_{C,F}$ = 27.7 Hz, CH_2Cl).
^{19}F NMR (470 MHz, $CDCl_3$) δ: -180.9 (ddd, 1F, $^2J_{H,F}$ = 46.5 Hz, $^3J_{H,F}$ = 22.6 Hz, $^2J_{C,F}$ = 17.7 Hz, F-1), -62.8 (s, 1F, CF_3).
HRMS (ESI), *m/z*: calcd. for $C_9H_7ClF_4Na^+$: 249.0070 $[M+Na]^+$; found:249.0074.

Compound 29
4-(2-chloro-1-fluoroethyl)benzonotrile

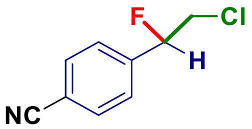

By following the **General procedure 1**, starting from 4-cyanobenzaldehyde (200 mg, 1.5 mmol, 1.0 equiv) in dry THF (3 mL), chloroiodomethane (0.16 mL, 2.25 mmol, 1.5 equiv), MeLi-LiBr 2.2 M solution in Et_2O (1.0 mL, 2.1 mmol, 1.4 equiv), Deoxo-Fluor 2.7 M solution in Toluene (1.2 mL, 3.3 mmol, 2.2 equiv), **compound 29** was obtained in 87% yield (240 mg) as colorless oil after column chromatography on neutral alumina grade IV (*n*-hexane/ethyl ether 5:5 as eluent).

^{1}H NMR (400 MHz, $CDCl_3$) δ: 7.73-7.71 (m, 2H, Ph H-2,6), 7.50-7.48 (m, 2H, Ph H-3,5), 5.67-5.51 (m, 1H, CHF), 3.84-3.82 (m, 1H, CH_2Cl), 3.79-3.77 (m, 1H, CH_2Cl).
^{13}C NMR (100 MHz, $CDCl_3$) δ: 141.5 (d, 1C, $^2J_{C,F}$ = 20.5 Hz, Ph C-4), 132.5 (Ph C-2,6), 126.4 (d, 2C, $^3J_{C,F}$ = 7.5 Hz, Ph C-3,5), 118.2 (CN), 113.1 (d, 1C, $^5J_{C,F}$ = 1.5 Hz, Ph C-1), 91.7 (d, $^1J_{C,F}$ = 181.0 Hz, CHF), 46.1 (d, 1C, $^2J_{C,F}$ = 27.7 Hz, CH_2Cl).
^{19}F NMR (470 MHz, $CDCl_3$) δ: -181.8 (m, 1F, F-1).
HRMS (ESI), *m/z*: calcd. for $C_9H_7ClFNNa^+$: 206.0149 $[M+Na]^+$; found:206.0150.

Compound 30
1-(2-chloro -1-fluoroethyl)-4-nitrobenzene

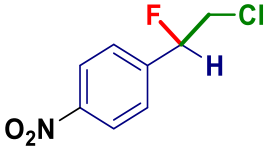

By following the **General procedure 1**, starting from 4-nitrobenzaldehyde (200 mg, 1.3 mmol, 1.0 equiv) in dry THF (3 mL), chloroiodomethane (0.15 mL, 2.0 mmol, 1.5 equiv), MeLi-LiBr 2.2 M solution in Et_2O (0.8 mL, 1.8 mmol, 1.4 equiv), Deoxo-Fluor 2.7 M solution in Toluene (1.1 mL 2.9 mmol, 2.2 equiv), **compound 30** was obtained in 80% yield (211 mg) as colorless oil without any further purification.

^{1}**H NMR** (400 MHz, $CDCl_3$) δ: 8.30-8.27 (m, 2H, Ph H-3,5), 7.57-7.55 (m, 2H, Ph H-2,6), 5.74 (ddd, 1H, $^{2}J_{H,F}$ = 46.7 Hz, $^{3}J_{H,H}$ = 6.4 Hz, $^{3}J_{H,H}$ = 4.4 Hz, CHF), 3.87-2.80 (m, 2H, CH_2Cl).
^{13}**C NMR** (100 MHz, $CDCl_3$) δ: 148.4 (1C, Ph C-4), 142.8 (d, 1C, $^{2}J_{C,F}$ = 20.4 Hz, Ph C-1), 126.7 (d, 2C, $^{3}J_{C,F}$ = 7.4 Hz, Ph C-2,6), 123.9 (2C, Ph C-3,5), 90.7 (d, 1C, $^{1}J_{C,F}$ = 181.3 Hz, CHF), 46.1 (d, 1C, $^{2}J_{C,F}$ = 26.7 Hz, CH_2Cl).
^{19}**F NMR** (470 MHz, C_6D_6) δ: -182.3 (m, 1F, F-1).
HRMS (ESI), *m/z*: calcd. for $C_8H_7ClFNO_2Na^+$: 226.0047 $[M+Na]^+$; found:226.0049.

Compound 31
1-(2-chloro -1-fluoroethyl)-4-methoxybenzene

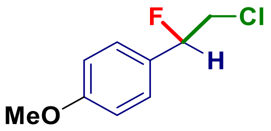

By following the **General procedure 1**, starting from 4-methoxybenzaldehyde (200 mg, 1.5 mmol, 1.0 equiv) in dry THF (3 mL), chloroiodomethane (0.16 mL, 2.25 mmol, 1.5 equiv), MeLi-LiBr 2.2 M solution in Et_2O (1.0 mL, 2.1 mmol, 1.4 equiv), Deoxo-Fluor 2.7 M solution in Toluene (1.2 mL, 3.3 mmol, 2.2 equiv), **compound 31** was obtained in 90% yield (255 mg) as colorless oil after column chromatography on neutral alumina grade IV (*n*-hexane/ethyl ether 8:2 as eluent).

^{1}**H NMR** (400 MHz, $CDCl_3$) δ: 7.31-7.28 (m, 2H, Ph H-2,6), 6.94-6.92 (m, 2H, Ph H-3,5), 5.54 (ddd, 1H, $^{2}J_{H,F}$ = 46.9 Hz, $^{3}J_{H,H}$ = 7.9 Hz, $^{3}J_{H,H}$ = 4.1 Hz, CHF), 3.84 (ddd, 1H, $^{3}J_{H,F}$ = 14.5 Hz, $^{2}J_{H,H}$ = 12.1 Hz, $^{3}J_{H,H}$ = 7.9 Hz, CH_2Cl), 3.83 (s, 3H, OCH_3), 3.71 (ddd, 1H, $^{3}J_{H,F}$ = 25.3 Hz, $^{2}J_{H,H}$ = 12.1 Hz, $^{3}J_{H,H}$ = 7.9 Hz, CH_2Cl).
^{13}**C NMR** (100 MHz, $CDCl_3$) δ: 160.3 (d, 1C, $^{5}J_{C,F}$ = 1.8 Hz, Ph C-4), 128.6 (d, 1C, $^{2}J_{C,F}$ = 20.6 Hz, Ph C-1), 127.4 (d, 2C, $^{3}J_{C,F}$ = 6.1 Hz, Ph C-2,6), 114.1 (2C, Ph C-3,5), 92.9 (d, 1C, $^{1}J_{C,F}$ = 176.9 Hz, CHF), 55.3 (1C, OCH_3), 46.7 (d, 1C, $^{2}J_{C,F}$ = 29.6 Hz, CH_2Cl).
^{19}**F NMR** (470 MHz, $CDCl_3$) δ: -173.9 (ddd, 1F, $^{2}J_{H,F}$ = 46.9 Hz, $^{3}J_{H,F}$ = 25.1 Hz, $^{3}J_{H,F}$ = 14.4 Hz, F-1).
HRMS (ESI), *m/z*: calcd. for $C_9H_{10}ClFONa^+$: 211.0302 $[M+Na]^+$; found:211.0305.

Compound 32
3-(2-chloro-1-fluoroethyl) thiophene

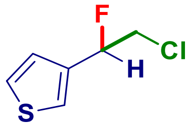

By following the **General procedure 1**, starting from 3-thiophenecarboxaldehyde (200 mg, 1.8 mmol, 1.0 equiv) in dry THF (3 mL), chloroiodomethane (0.2 mL, 2.7 mmol, 1.5 equiv), MeLi-LiBr 2.2 M solution in Et_2O (1.15 mL, 2.5 mmol, 1.4 equiv), Deoxo-Fluor 2.7 M solution in Toluene (1.47 mL, 4.0 mmol, 2.2 equiv), **compound 32** was obtained in 92% yield (273 mg) as colorless oil after column chromatography on neutral alumina grade IV (*n*-hexane as eluent).

^{1}**H NMR** (400 MHz, C_6D_6) δ: 6.80-6.77 (m, 1H, Th H-5), 6.76-6.74 (m, 1H, Th H-2), 6.65-6.61 (m, 1H, Th H-4), 5.28-5.11 (m, 1H, CHF), 3.39-3.28 (m, 1H, CH_2Cl), 3.23-3.10 (m 1H, CH_2Cl).

^{13}**C NMR** (100 MHz, C_6D_6) δ: 138.0 (d, 1C, $^2J_{C,F}$ = 22.0 Hz, Th C-3), 126.6 (Th C-5), 125.3 (d, 1C, $^3J_{C,F}$ = 3.8 Hz, Th C-4), 123.5 (d, 1C, $^3J_{C,F}$ = 7.1 Hz, Th C-2), 89.4 (d, 1C, $^1J_{C,F}$ = 176.5 Hz, CHF), 46.2 (d, 1C, $^2J_{C,F}$ = 27.7 Hz, CH_2Cl).
^{19}**F NMR** (470 MHz, C_6D_6) δ: -172.3 (m, 1F, F-1).
HRMS (ESI), *m/z*: calcd. for $C_6H_6ClFSNa^+$: 186.9761 $[M+Na]^+$; found:186.9763.

Compound 33
2-methyl-2-propanyl 4-(2-chloro-1-fluoroethyl) benzoate

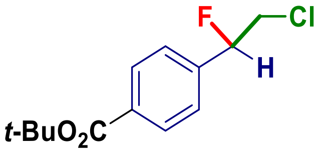

By following the **General procedure 1**, starting from *tert*-butyl 4-formylbenzoate (200 mg, 1.0 mmol, 1.0 equiv) in dry THF (3 mL), chloroiodomethane (0.15 mL, 2.0 mmol, 1.5 equiv), MeLi-LiBr 2.2 M solution in Et_2O (0.8 mL, 1.8 mmol, 1.4 equiv), Deoxo-Fluor 2.7 M solution in Toluene (0.8 mL, 2.2 mmol, 2.2 equiv), **compound 33** was obtained in 86% yield (222 mg) as colorless oil after column chromatography on neutral alumina grade IV (*n*-hexane/ ethyl ether 5:5 as eluent).

^{1}**H NMR** (400 MHz, $CDCl_3$) δ: 7.94-7.92 (m, 2H, Ph H-2,6), 7.32-7.15 (m, 2H, Ph H-3,5), 5.55 (ddd, 1H, $^2J_{H,F}$ = 47.0 Hz, $^3J_{H,H}$ = 7.3 Hz, $^3J_{H,H}$ = 3.8 Hz, CHF), 3.76-3.61 (m, 2H, CH_2Cl), 1.50 (s, 9H, *t*-Bu).
^{13}**C NMR** (100 MHz, C_6D_6) δ: 165.0 (d, 1C, $^6J_{C,F}$ = 0.4 Hz, CO_2 *t*-Bu), 140.7 (d, 1C, $^2J_{C,F}$ = 20.1 Hz, Ph C-4), 132.7 (d, 1C, $^5J_{C,F}$ = 1.4 Hz, Ph C-1), 129.7 (d, 2C, $^4J_{C,F}$ = 0.5 Hz, Ph C-2,6), 125.4 (d, 2C, $^3J_{C,F}$ = 7.1 Hz, Ph C-3,5), 92.3 (d, 1C, $^1J_{C,F}$ = 180.0 Hz, CHF), 81.3 (1C, CO_2 *t*-Bu), 46.5 (d, 1C, $^2J_{C,F}$ = 27.0 Hz, CH_2Cl), 28.1 (3C, *t*-Bu).
^{19}**F NMR** (470 MHz, C_6D_6) δ: -181.3 (m, 1F, $^2J_{H,F}$ = 47.0 Hz, $^3J_{H,F}$ = 24.3 Hz, $^3J_{H,F}$ = 17.7 Hz, F-1).
HRMS (ESI), *m/z*: calcd. for $C_{13}H_{16}ClFO_2Na^+$: 281.0721 $[M+Na]^+$; found:281.0725.

Compound 34
[4-(2-chloro-1-fluoroethyl)phenyl] (1-piperidinyl) methanone

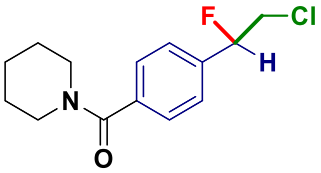

By following the **General procedure 1**, starting from 4-(piperidine-1-carbonyl)benzaldehyde (200 mg, 0.9 mmol, 1.0 equiv) in dry THF (3 mL), chloroiodomethane (0.1 mL, 1.4 mmol, 1.5 equiv), MeLi-LiBr 2.2 M solution in Et_2O (0.6 mL, 1.3 mmol, 1.4 equiv), Deoxo-Fluor 2.7 M solution in Toluene (0.7 mL, 2.0 mmol, 2.2 equiv), **compound 34** was obtained in 91% yield (221 mg) as colorless oil after column chromatography on neutral alumina grade IV (*n*-hexane/ ethyl ether 5:5 as eluent).

^{1}**H NMR** (400 MHz, $CDCl_3$) δ: 7.47-7.43 (m, 2H, Ph H-2,6), 7.41-7.37 (m, 2H, Ph H-3,5), 5.6 1(ddd, 1H, $^2J_{H,F}$ = 47.1 Hz, $^3J_{H,H}$ = 7.6 Hz, $^3J_{H,H}$ = 4.0 Hz, CHF), 3.81 (ddd, 1H, $^3J_{H,F}$ = 16.8 Hz, $^2J_{H,H}$ = 12.2 Hz, $^3J_{H,H}$ = 7.6 Hz, CH_2Cl), 3.72 (ddd, 1H, $^3J_{H,F}$ = 25.2 Hz, $^2J_{H,H}$ = 12.2 Hz, $^3J_{H,H}$ = 3.6 Hz, CH_2Cl), 3.70 (brs, 2H, H-6, CH_2N), 3.31 (brs, 2H, H-2, CH_2N), 1.83 (brs, 2H, H-4, CH_2), 1.67 (brs, 4H, H-3,5, CH_2).

^{13}C NMR (100 MHz, $CDCl_3$) δ: 169.6 (1C, CO), 137.9 (d, 1C, $^2J_{C,F}$ = 20.2 Hz, Ph C-4), 137.6 (d, 1C, $^5J_{C,F}$ = 1.6 Hz, Ph C-1), 127.4 (2C, Ph C-2,6), 126.0 (d, 2C, $^3J_{C,F}$ = 6.9 Hz, Ph C-3,5), 92.7 (d, 1C, $^1J_{C,F}$ = 179.0 Hz, CHF), 48.3 (1C, C-6, CH_2N), 46.7 (d, 1C, $^2J_{C,F}$ = 27.9 Hz, CH_2Cl), 42.8 (1C, C-2, CH_2N), 26.1 (1C, C-3, CH_2), 25.7 (1C, C-5, CH_2), 24.6 (1C, C-4, CH_2).
HRMS (ESI), *m/z*: calcd. for $C_{14}H_{17}ClFNONH^+$: 270.1055 $[M+Na]^+$; found:270.1057.

Compound 35
(4-chloro -3-fluorobuthyl) benzene[5]

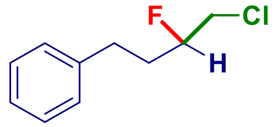

By following the **General procedure 1**, starting from 3-phenylpropionaldehyde (200 mg, 1.5 mmol, 1.0 equiv) in dry THF (3 mL), chloroiodomethane (0.16 mL, 2.25 mmol, 1.5 equiv), MeLi-LiBr 2.2 M solution in Et_2O (1.0 mL, 2.1 mmol, 1.4 equiv), Deoxo-Fluor 2.7 M solution in Toluene (1.2 mL, 3.3 mmol, 2.2 equiv), **compound 35** was obtained in 82% yield (230 mg) as colorless oil after column chromatography on neutral alumina grade IV (*n*-hexane as eluent).

^{1}H NMR (400 MHz, $CDCl_3$) δ: 7.33-7.28 (m, 2H, Ph H-3,5), 7.24-7.20 (m, 1H, Ph H-4), 7.20-7.19 (m, 2H, Ph H-2,6), 4.73-4.56 (m, 1H, $^2J_{H,F}$ = 47.8 Hz, CHF), 3.66-3.60 (m, 2H, CH_2Cl), 2.88-2.69 (m, 2H, C-3H_2), 2.17-1.88 (m, 2H, C-2H_2).
^{13}C NMR (100 MHz, $CDCl_3$) δ: 140.6 (1C, Ph C-1), 128.6 (2C, Ph C-3,5), 128.4 (2C, Ph C-2,6), 126.3 (1C, Ph C-4), 91.4 (d, 1C, $^1J_{C,F}$ = 175.4 Hz, CHF), 45.7 (d, 1C, $^2J_{C,F}$ = 25.4 Hz, CH_2Cl), 34.1 (d, 1C, $^2J_{C,F}$ = 20.7 Hz, CH_2), 30.9 (d, 1C, $^3J_{C,F}$ = 4.2 Hz, CH_2).
^{19}F NMR (470 MHz, $CDCl_3$) δ: -183.6 (m, 1F, F-1).
HRMS (ESI), *m/z*: calcd. for $C_{10}H_{12}ClFNa^+$: 209.0509 $[M+Na]^+$; found:209.0511.

Compound 36
1-chloro-2-fluorononane

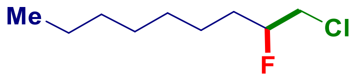

By following the **General procedure 1**, starting from octanal (200 mg, 1.6 mmol, 1.0 equiv) in dry THF (3 mL), chloroiodomethane (0.17 mL, 2.3 mmol, 1.5 equiv), MeLi-LiBr 2.2 M solution in Et_2O (1.0 mL, 2.2 mmol, 1.4 equiv), Deoxo-Fluor 2.7 M solution in Toluene (1.3 mL, 3.4 mmol, 2.2 equiv), **compound 36** was obtained in 80% yield (231 mg) as colorless oil after column chromatography on neutral alumina grade IV (*n*-hexane as eluent).

^{1}H NMR (400 MHz, $CDCl_3$) δ: 4.63 (ddt, 1H, $^2J_{H,F}$ = 48.0 Hz, $^3J_{H,H}$ = 12.6 Hz, $^3J_{H,H}$ = 4.8 Hz, CH_2Cl), 3.64-3.58 (m, 2H, CH_2Cl), 1.75-1.69 (m, 2H, H-3), 1.67-1.63 (m, 10H, H-4,5,6,7,8), 0.89 (t, 3H, $^3J_{H,H}$ = 6.8 Hz, CH_3).
^{13}C NMR (100 MHz, $CDCl_3$) δ: 92.6 (d, 1C, $^1J_{C,F}$ = 174.8 Hz, CF), 46.0 (d, 1C, $^2J_{C,F}$ = 25.4 Hz, CH_2Cl), 32.6 (d, 1C, $^2J_{C,F}$ = 20.7 Hz, C-3), 31.9 (1C, C-7), 29.3 (d, 1C, $^3J_{C,F}$ = 18.9 Hz, C-4), 24.8 (d, 1C, $^4J_{C,F}$ = 4.4 Hz, C-5), 22.6 (2C, C-6,8), 14.2 (1C, CH_3).
^{19}F NMR (470 MHz, $CDCl_3$) δ: -181.8 (m, 1F, F-1).

HRMS (ESI), *m/z*: calcd. for $C_9H_{18}ClFNa^+$: 203.0979 [M+Na]$^+$; found:203.0981.

Compound 37

[(1E)-4-chloro-3-fluoro-2-methyl-1-buten-1-yl] benzene

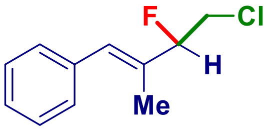

By following the **General procedure 1**, starting from α-methyl-trans-cinnamaldehyde (200 mg, 1.4 mmol, 1.0 equiv) in dry THF (3 mL),), chloroiodomethane (0.15 mL, 2.1 mmol, 1.5 equiv), MeLi-LiBr 2.2 M solution in Et_2O (0.9 mL, 2.0 mmol, 1.4 equiv), Deoxo-Fluor 2.7 M solution in Toluene (1.14 mL, 3.1 mmol, 2.2 equiv), **compound 37** was obtained in 88% yield (245 mg) as colorless oil after column chromatography on neutral alumina grade IV (*n*-hexane as eluent).

^{1}H NMR (400 MHz, $CDCl_3$) δ: 7.39-7.34 (m, 2H, Ph H-3,5), 7.32-7.29 (m, 2H, Ph H-2,6), 7.28-7.25 (m, 1H, Ph H-4), 6.64 (s, 1H, C-1), 5.09 (ddd, 1H, $^2J_{H,F}$ = 47.5 Hz, $^3J_{H,H}$ = 7.2 Hz, $^3J_{H,H}$ = 4.7 Hz, CHF), 3.86 -3.67 (m, 2H, CH_2Cl), 1.91 (d, 1H, $^3J_{H,H}$ = 1.4 Hz, CH_3).
^{13}C NMR (100 MHz, $CDCl_3$) δ: 136.2 (Ph C-1), 132.3 (d, 1C, $^2J_{C,F}$ = 17.0 Hz, C-2), 130.0 (d, 1C, $^3J_{C,F}$ = 10.6 Hz, C-1), 129.0 (d, 2C, $^5J_{C,F}$ = 1.4 Hz, Ph C-2,6), 128.3 (2C, Ph C-3,5), 127.3 (Ph C-4), 96.1 (d, 1C, $^1J_{C,F}$ = 178.2 Hz, CHF), 44.5 (d, 1C, $^2J_{C,F}$ = 29.3 Hz, CH_2Cl), 13.0 (d, 1C, $^3J_{C,F}$ = 3.1 Hz, CH_3).
^{19}F NMR (470 MHz, $CDCl_3$) δ: -178.0 (dddd, 1F, $^2J_{H,F}$ = 47.5 Hz, $^2J_{C,F}$ = 22.7 Hz, $^3J_{H,F}$ = 14.9 Hz, $^3J_{H,F}$ = 2.7 Hz, F-1).
HRMS (ESI), *m/z*: calcd. for $C_{11}H_{12}ClFNa^+$: 221.0509 [M+Na]$^+$; found:221.0512.

Compound 38

(2-bromo -1-fluoroethyl) benzene[6]

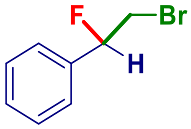

By following the **General procedure 1**, starting from benzaldehyde (200 mg, 1.9 mmol, 1.0 equiv) in dry THF (3 mL), bromoiodomethane (0.2 mL, 2.8 mmol, 1.5 equiv), MeLi-LiBr 2.2 M solution in Et_2O (1.2 mL, 2.7 mmol, 1.4 equiv), Deoxo-Fluor 2.7 M solution in Toluene (1.6 mL, 4.2 mmol, 2.2 equiv), **compound 38** was obtained in 83% yield (318 mg) as colorless oil after column chromatography on neutral alumina grade IV (*n*-hexane as eluent).

^{1}H NMR (400 MHz, $CDCl_3$) δ: 7.42-7.39 (m, 3H, Ph H-3,4,5), 7.38-7.35 (m, 2H, Ph H-2,6), 5.63 (ddd, 1H, $^2J_{H,F}$ = 46.9 Hz, $^3J_{H,H}$ = 7.9 Hz, $^3J_{H,H}$ = 4.1 Hz, CHF), 3.68 (ddd, 1H, $^2J_{H,H}$ = 11.4 Hz, $^3J_{H,F}$ = 15.2 Hz, $^3J_{H,F}$ = 7.9 Hz, CH_2Cl), 3.64 (ddd, 1H, $^2J_{H,H}$ = 11.4 Hz, $^3J_{H,F}$ = 25.8 Hz, $^3J_{H,F}$ = 4.1 Hz, CH_2Cl).
^{13}C NMR (100 MHz, $CDCl_3$) δ: 137.1 (d, 1C, $^2J_{C,F}$ = 20.2 Hz, Ph C-1), 129.3 (d, 1C, $^5J_{C,F}$ = 1.8 Hz, Ph C-4), 128.7 (2C, Ph C-3,5), 125.7 (d, 2C, $^3J_{C,F}$ = 6.6 Hz, Ph C-2,6), 92.8 (d, 1C, $^1J_{C,F}$ = 178.0 Hz, CHF), 34.3 (d, 1C, $^2J_{C,F}$ = 28.4 Hz, CH_2Br).
^{19}F NMR (470 MHz, $CDCl_3$) δ: -174.1 (ddd, 1F, $^2J_{H,F}$ = 46.9 Hz, $^3J_{H,F}$ = 25.8 Hz, $^3J_{H,F}$ = 15.2 Hz, F-1).
HRMS (ESI), *m/z*: calcd. for $C_8H_8BrFNa^+$: 224.9691 [M+Na]$^+$; found:224.9695.

Compound 39

(2,2-dibromo -1-fluoroethyl) benzene[7]

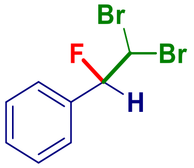

By following the **General procedure 2**, starting from benzaldehyde (200 mg, 1.9 mmol, 1.0 equiv) in dry THF (3 mL), dibromomethane (0.2 mL, 2.8 mmol, 1.5 equiv), LDA 2.0 M in THF (1.3 mL, 2.7 mmol, 1.4 equiv), Deoxo-Fluor 2.7 M solution in Toluene (1.6 mL, 4.2 mmol, 2.2 equiv), **compound 39** was obtained in 85% yield (455 mg) as colorless oil without any further purification.

^{1}H NMR (400 MHz, $CDCl_3$) δ: 7.43 (s, 5H, Ph H-2,3,4,5,6), 5.69 (dd, 1H, $^2J_{H,F}$ = 45.1 Hz, $^3J_{H,H}$ = 5.5 Hz, CHF), 5.80 (dd, 1H, $^3J_{H,F}$ = 13.1 Hz, $^3J_{H,H}$ = 5.5 Hz, $CHBr_2$).
^{13}C NMR (100 MHz, $CDCl_3$) δ: 134.7 (d, 1C, $^2J_{C,F}$ = 20.8 Hz, Ph C-1), 129.8 (d, 1C, $^5J_{C,F}$ = 1.6 Hz, Ph C-4), 128.5 (2C, Ph C-3,5), 126.9 (d, 2C, $^3J_{C,F}$ = 6.6 Hz, Ph C-2,6), 95.2 (d, 1C, $^1J_{C,F}$ = 187.0 Hz, CHF), 44.5 (d, 1C, $^2J_{C,F}$ = 31.4 Hz, $CHBr_2$).
^{19}F NMR (470 MHz, $CDCl_3$) δ: -167.5 (dd, 1F, $^2J_{H,F}$ = 45.1 Hz, $^3J_{H,F}$ = 13.1 Hz, F-1).
HRMS (ESI), *m/z*: calcd. for $C_8H_7Br_2FNa^+$: 304.8776 $[M+Na]^+$; found:304.8780.

Compound 40
1-chloro-4-(2,2,2-trichloro -1-fluoroethyl) benzene[8]

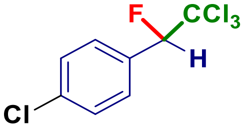

By following the **General procedure 3**, starting from 4-chlorobenzaldehyde (200 mg, 1.4 mmol, 1.0 equiv) in dry THF (3 mL), $TMSCCl_3$ (536 mg, 2.8 mmol, 2.0 equiv), potassium *tert*-pentoxide 0.9 M (2.8 ml, 2.5 mmol, 1.8 equiv), Deoxo-Fluor 2.7 M solution in Toluene (1.1 mL, 3.1 mmol, 2.2 equiv), **compound 40** was obtained in 93% yield (341 mg) as colorless oil without any further purification.

^{1}H NMR (400 MHz, $CDCl_3$) δ: 7.56-7.53 (m, 2H, Ph H-3,5), 7.42-7.40 (m, 2H, Ph H-2,6), 5.75 (d, 1H, $^2J_{H,F}$ = 43.5 Hz, CHF).
^{13}C NMR (100 MHz, $CDCl_3$) δ: 136.6 (d, 1C, $^5J_{C,F}$ = 1.5 Hz, Ph C-1), 130.3 (d, 1C, $^2J_{C,F}$ = 21.7 Hz, Ph C-4), 130.1 (d, 2C, $^3J_{C,F}$ = 6.7 Hz, Ph C-3,5), 128.2 (2C, Ph C-2,6), 97.8 (d, 1C, $^2J_{C,F}$ = 32.2 Hz, CCl_3), 97.8 (d, 1C, $^1J_{C,F}$ = 197.6 Hz, CHF).
^{19}F NMR (470 MHz, C_6D_6) δ: -165.6 (d, 1F, $^2J_{H,F}$ = 43.4 Hz, F-1).
HRMS (ESI), *m/z*: calcd. for $C_8H_5Cl_4FNa^+$: 284.8998 $[M+Na]^+$; found:284.9001.

Compound 41
[(1E)-3-fluoro-4-iodo-1-buten-1-yl] benzene

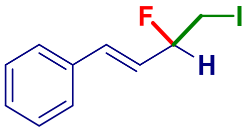

By following the **General procedure 1**, starting from cinnamaldehyde (200 mg, 1.5 mmol, 1.0 equiv) in dry THF (3 mL), diiodomethane (0.18 mL, 2.3 mmol, 1.5 equiv), MeLi-LiBr 2.2 M solution in Et_2O (1.0 mL, 2.1 mmol, 1.4 equiv), Deoxo-Fluor 2.7 M solution in Toluene (1.2 mL, 3.3 mmol, 2.2 equiv), **compound 41** was obtained in 84% yield (348 mg) as colorless oil without any further purification.

^{1}H NMR (400 MHz, $CDCl_3$) δ: 7.43-7.40 (m, 2H, Ph H-2,6), 7.37-7.30 (m, 2H, Ph H-3,5), 7.35-7.30 (m, 1H, Ph H-4), 6.74 (dd, 1H, $^3J_{H,H}$ = 16.0 Hz, $^4J_{H,F}$ = 3.7 Hz, C-1H), 6.21 (ddd, 1H, $^3J_{H,H}$ = 16.0 Hz, $^3J_{H,H}$ = 12.1 Hz, $^4J_{H,F}$ = 6.8 Hz, C-2H), 5.20-5.06 (m, 1H, CHF), 3.44 -3.37 (m, 2H, CH_2I).
^{13}C NMR (100 MHz, $CDCl_3$) δ: 135.4 (d, 1C, $^4J_{C,F}$ = 1.5 Hz, Ph C-1), 135.0 (d, 1C, $^3J_{C,F}$ = 11.4 Hz, C-1), 128.7 (2C, Ph C-3,5), 128.6 (1C, Ph C-4), 126.9 (d, 2C, $^5J_{C,F}$ = 1.4 Hz, Ph C-2,6), 125.0 (d, 1C, $^3J_{C,F}$ = 19.4 Hz, C-2), 91.8 (d, 1C, $^1J_{C,F}$ = 173.7 Hz, CHF), 6.4 (d, 1C, $^2J_{C,F}$ = 27.5 Hz, CH_2I).
^{19}F NMR (470 MHz, $CDCl_3$) δ: -162.3 (m, 1F, F-1).
HRMS (ESI), *m/z*: calcd. for $C_{10}H_{10}FINa^+$: 298.9709 $[M+Na]^+$; found:298.9711.

Compound 42
1-chloro-4-(1-fluoroethyl) benzene

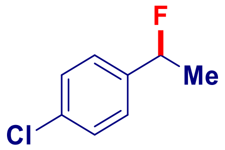

By following the **General procedure 1**, starting from 4-chloro acetophenone (200 mg, 1.3 mmol, 1.0 equiv) in dry THF (3 mL), MeLi 1.6 M solution in Et_2O (1.1 mL, 1.8 mmol, 1.4 equiv), Deoxo-Fluor 2.7 M solution in Toluene (1.1 mL, 2.9 mmol, 2.2 equiv), **compound 42** was obtained in 54% yield (110 mg) as colorless after column chromatography on silica gel (*n*-hexane as eluent).

^{1}H NMR (400 MHz, $CDCl_3$) δ: 7.35 (d, 2H, *J* = 8.4 Hz, Ph H-2,6), 7.28 (d, 2H, *J* = 8.4 Hz, Ph H-3,5), 5.60 (dq, 1H, $^2J_{H,F}$ = 47.5 Hz, $^3J_{H,H}$ = 6.5 Hz, CHF), 1.62 (dd, 3H, $^3J_{H,F}$ = 23.8 Hz, $^3J_{H,H}$ = 6.4 Hz, CH_3).
^{13}C NMR (100 MHz, $CDCl_3$) δ: 140.1 (d, 1C, $^2J_{C,F}$ = 20.0 Hz, Ph C-4), 134.2 (d, 1C, $J_{C,F}$ = 1.7 Hz, Ph C-1), 128.8 (2C, Ph C-3,5), 126.8 (d, 2C, $^3J_{C,F}$ = 6.9 Hz, Ph C-2,6), 90.4 (d, 1C, $^1J_{C,F}$ = 168.2 Hz, CF), 23.0 (d, 1C, $^2J_{C,F}$ = 28.3 Hz, CH_3).
^{19}F NMR (470 MHz, $CDCl_3$) δ: -167.5 (dq, 1F, $^2J_{H,F}$ = 47.9 Hz, $^3J_{H,F}$ = 23.9 Hz, F-1).
HRMS (ESI), *m/z*: calcd. for $C_8H_8ClFNa^+$: 181.0196 $[M+Na]^+$; found:181.0110.

4. References

[1] L. T. C. Crespo, R. d. S. Ribeiro, M. C. S. de Mattos, P. M. Esteves, *Synthesis* **2010**, 2379-2382.

[2] C. Ye, B. Twamley, J. n. M. Shreeve, *Org. Lett.* **2005**, *7*, 3961-3964.

[3] D. D. DesMarteau, Z. Q. Xu, M. Witz, *J. Org. Chem.* **1992**, *57*, 629-635.

[4] S. Hamman, C. Béguin, C. Charlon, C. Luu-Duc, *Org. Magn. Reson.* **1983**, *21*, 361-366.

[5] K.-J. Bian, D. Nemoto, X.-W. Chen, S.-C. Kao, J. Hooson, J. G. West, *Chem. Sci.* **2024**, *15*, 124-133.

[6] aC. J. Thomson, Q. Zhang, N. Al-Maharik, M. Bühl, D. B. Cordes, A. M. Z. Slawin, D. O'Hagan, *Chem. Commun* **2018**, *54*, 8415-8418; bS. Liang, F. J. Barrios, O. E. Okoromoba, Z. Hetman, B. Xu, G. B. Hammond, *J. Fluor. Chem.* **2017**, *203*, 136-139; cM. Stangier, A. Scheremetjew, L. Ackermann, *Chem. Eur. J.* **2022**, *28*, e202201654.

[7] D. R. Williams, M. W. Fultz, T. E. Christos, J. S. Carter, *Tetrahedron Lett.* **2010**, *51*, 121-124.

[8] R. Anilkumar, D. J. Burton, *J. Fluor. Chem.* **2005**, *126*, 1174-1184.

5. ^{1}H-, ^{13}C- and ^{19}F-NMR Spectra for all the Compounds

Compound 2

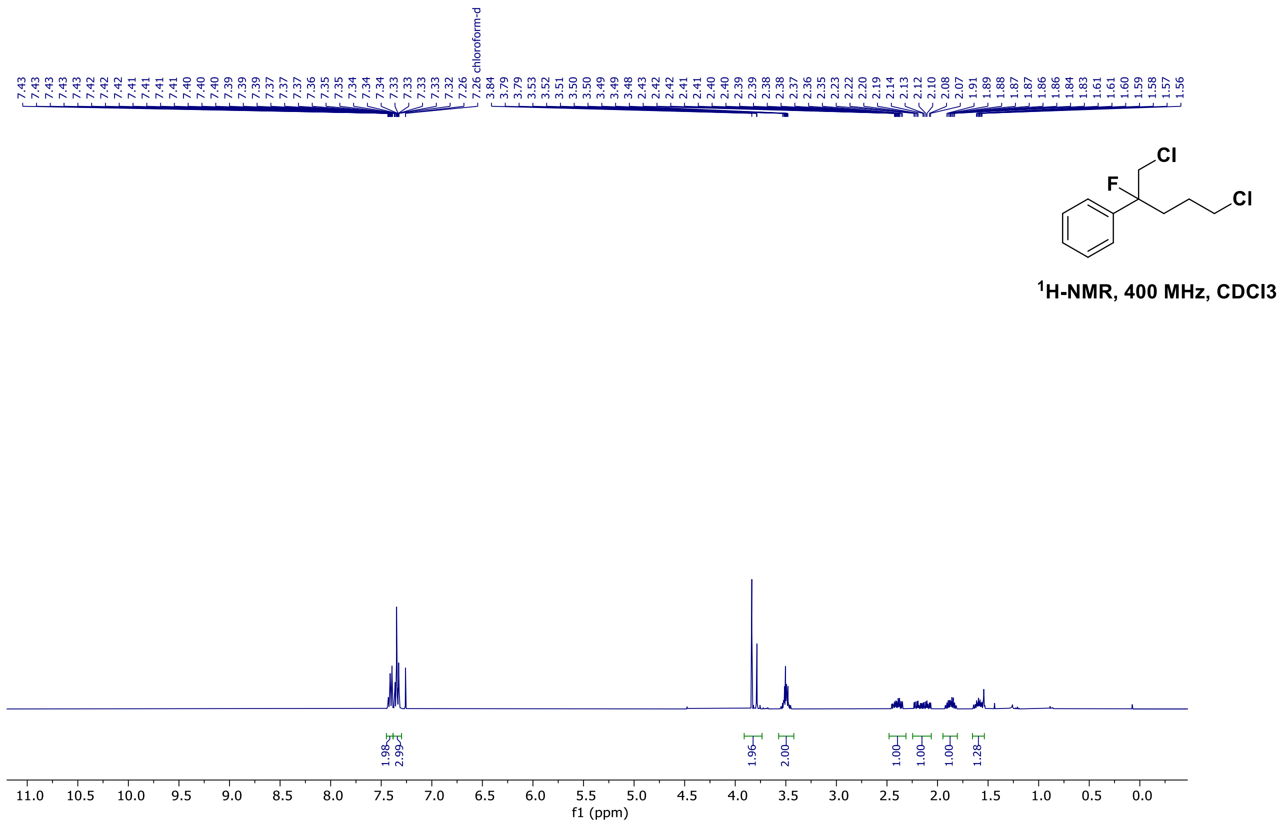
1H-NMR, 400 MHz, CDCl3
f1 (ppm)
Cl
F
Cl

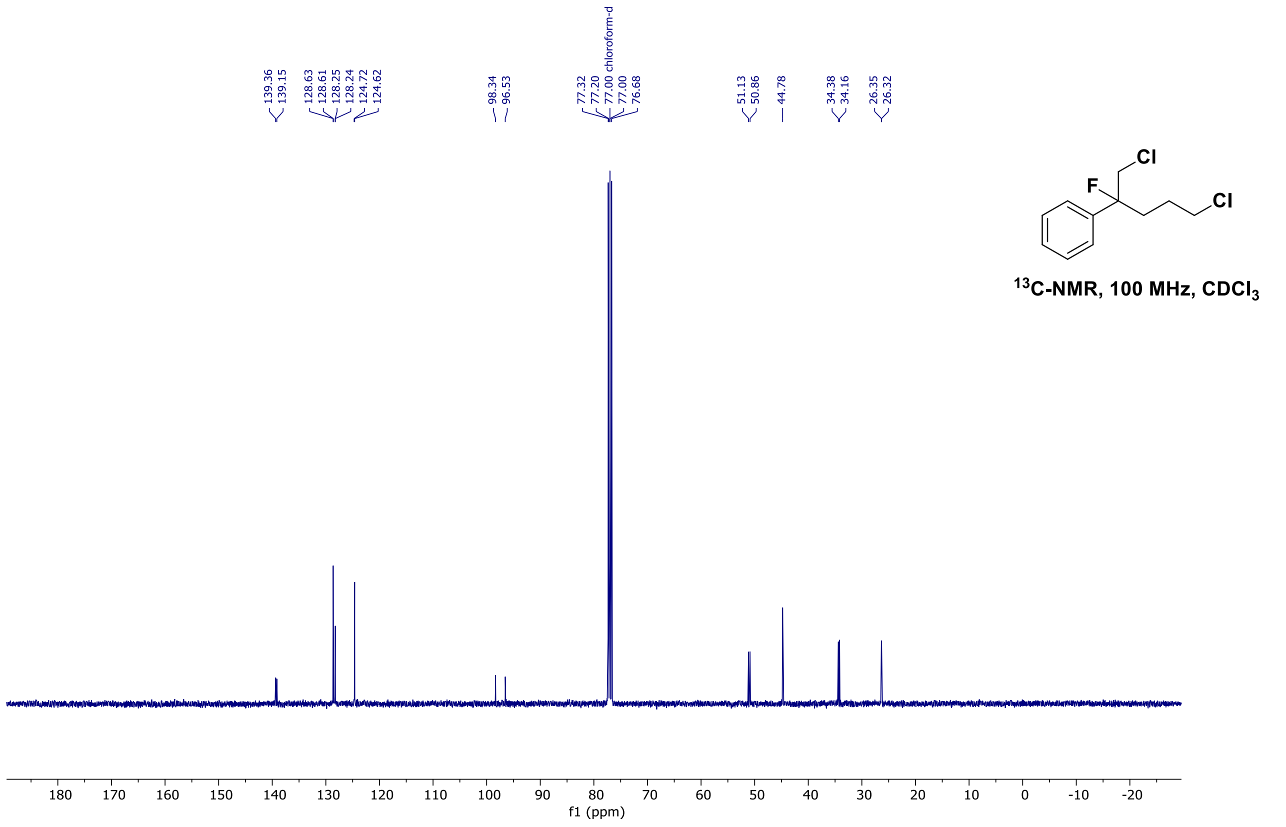
13C-NMR, 100 MHz, CDCl3
f1 (ppm)
Cl
F
Cl

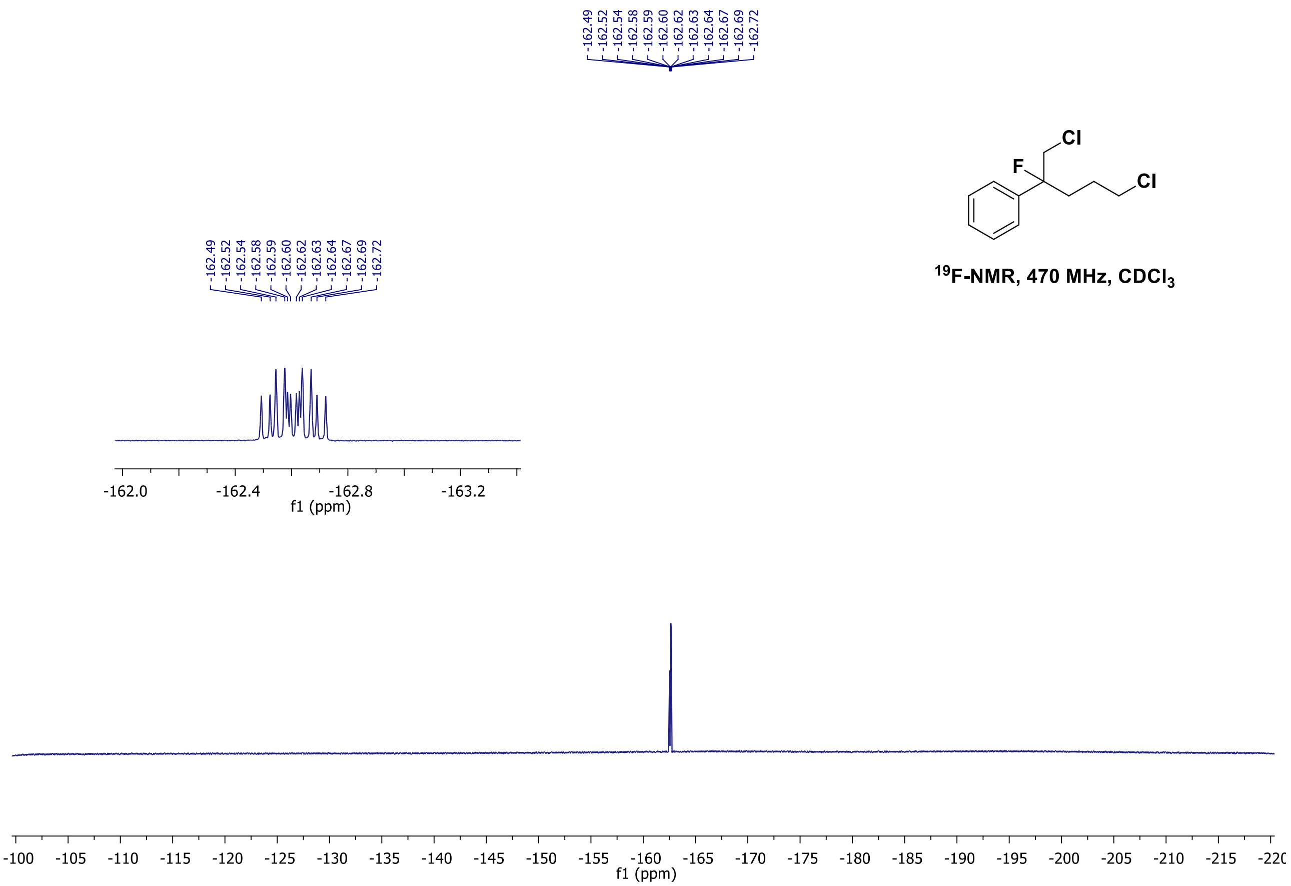

-162.49
-162.52
-162.54
-162.58
-162.59
-162.60
-162.62
-162.63
-162.64
-162.67
-162.69
-162.72
Cl
F
Cl
19F-NMR, 470 MHz, CDCl3
-162.0
-162.4
-162.8
-163.2
f1 (ppm)
-100
-105
-110
-115
-120
-125
-130
-135
-140
-145
-150
-155
-160
-165
-170
-175
-180
-185
-190
-195
-200
-205
-210
-215
-220
f1 (ppm)

Compound 2a

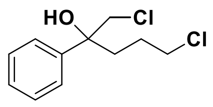

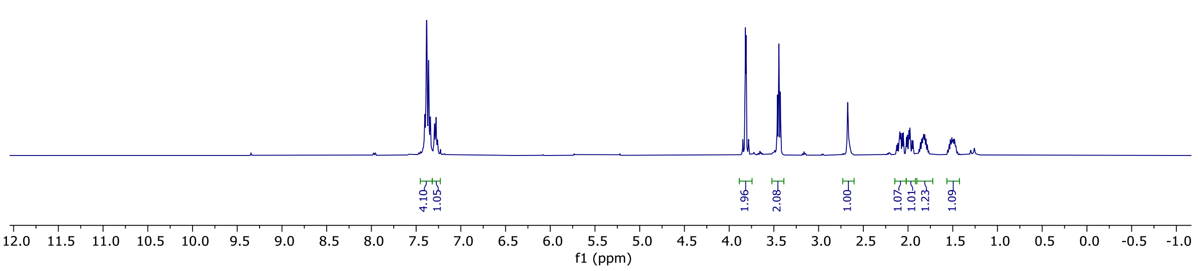

^{1}H NMR, 400 MHz, $CDCl_3$

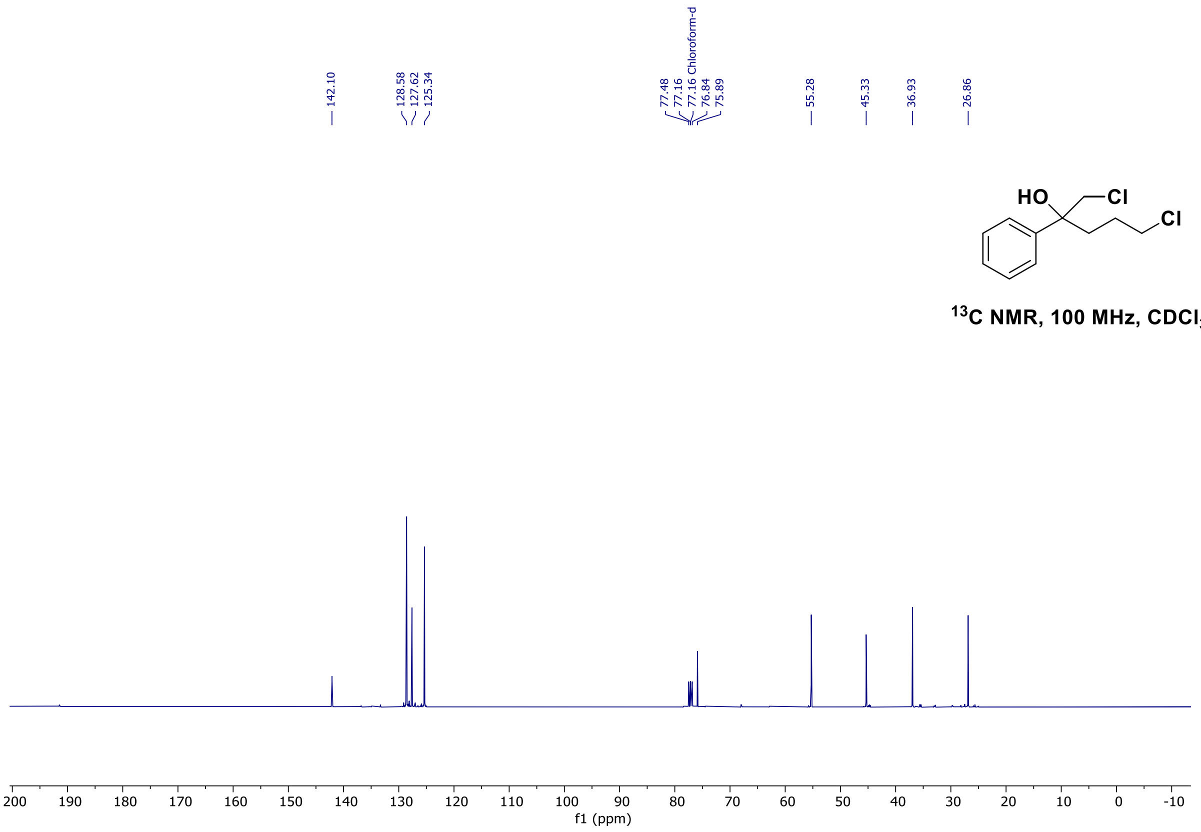

^{13}C NMR, 100 MHz, $CDCl_3$

Compound 3

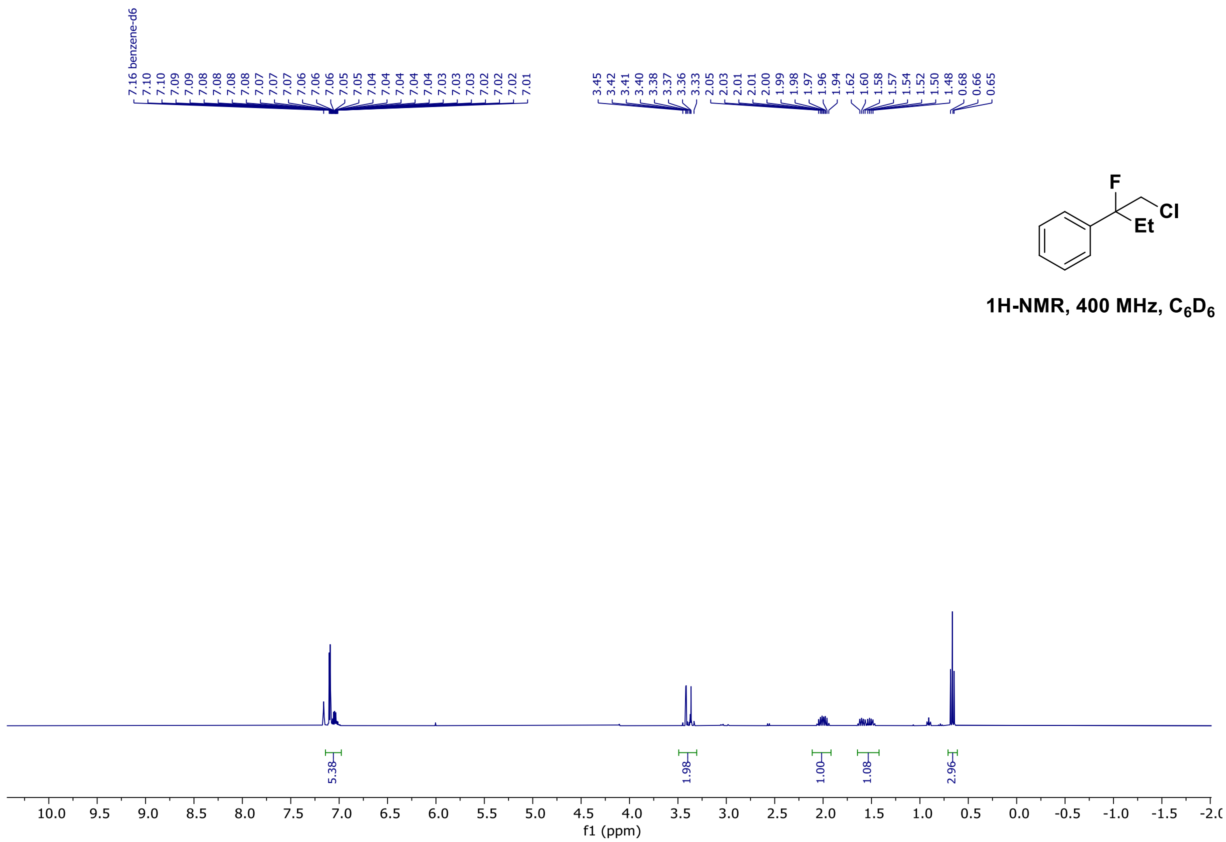

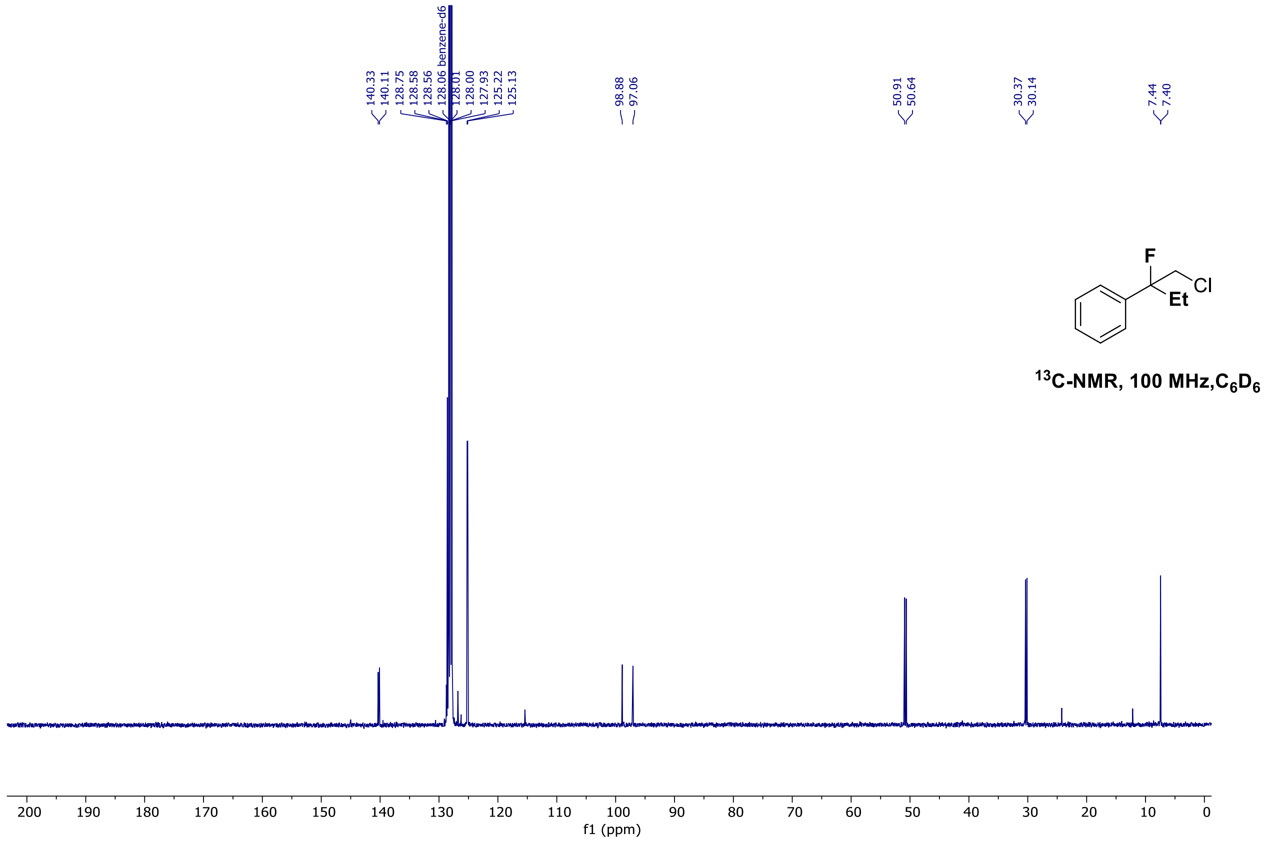

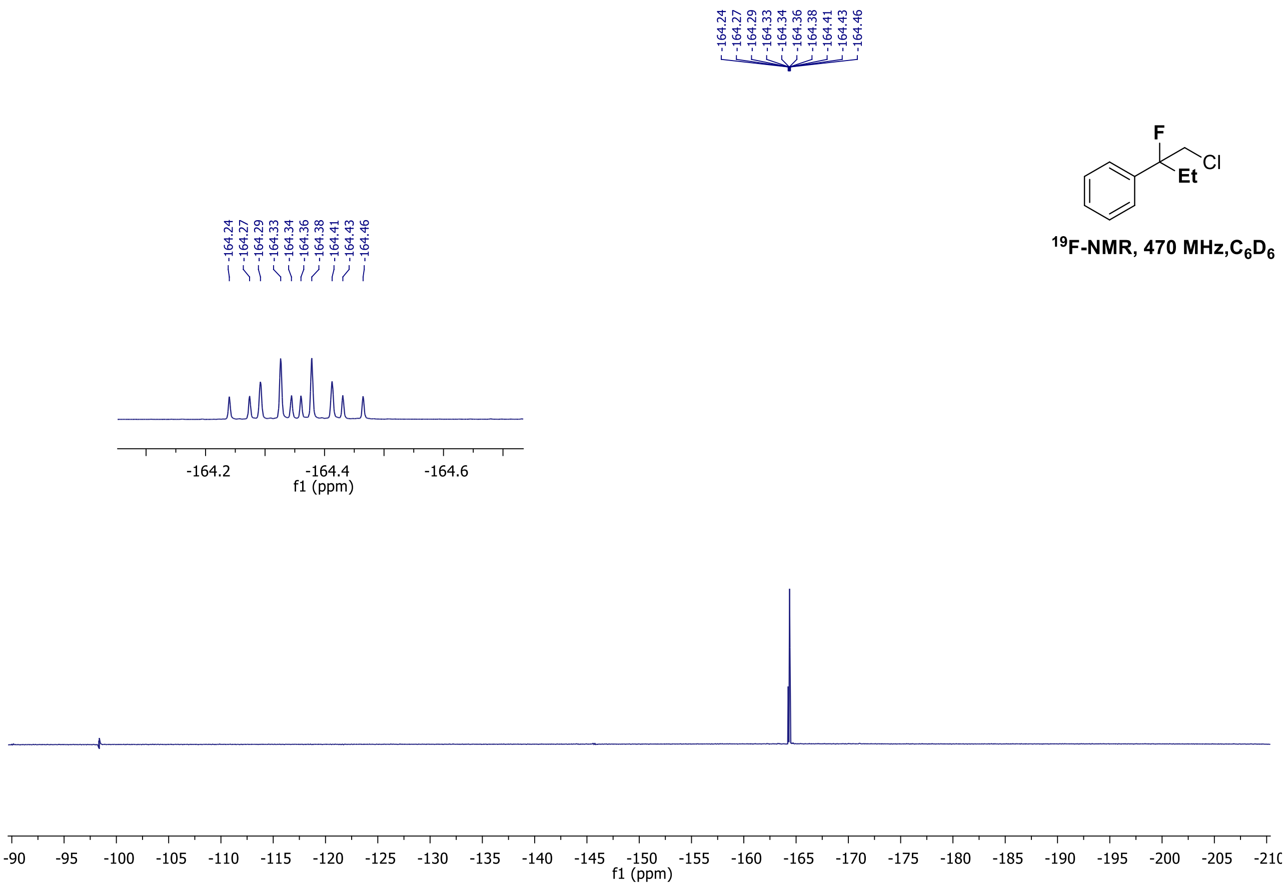

-164.24
-164.27
-164.29
-164.33
-164.34
-164.36
-164.38
-164.41
-164.43
-164.46
F
Cl
Et
^{19}F-NMR, 470 MHz,C_6D_6
-164.2
-164.4
-164.6
f1 (ppm)
-90 -95 -100 -105 -110 -115 -120 -125 -130 -135 -140 -145 -150 -155 -160 -165 -170 -175 -180 -185 -190 -195 -200 -205 -210
f1 (ppm)

Compound 4

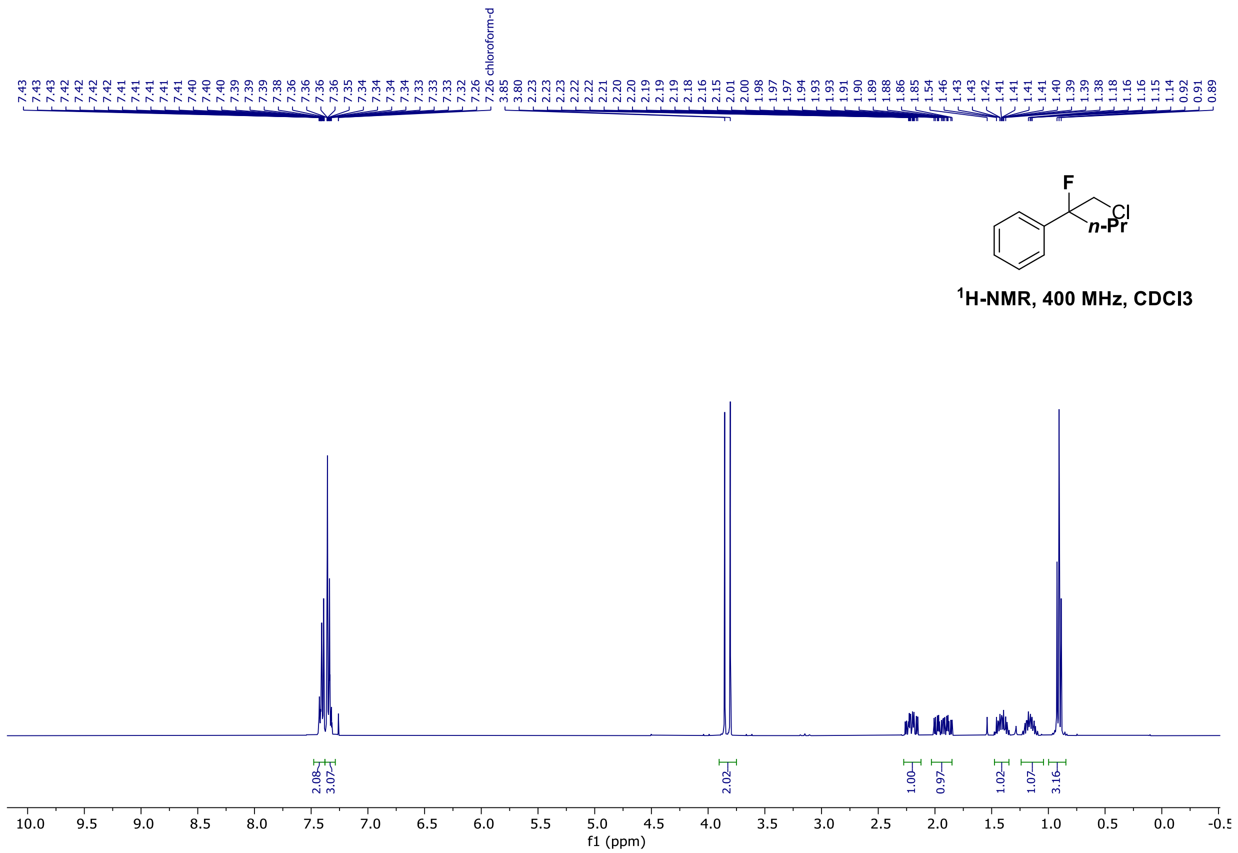

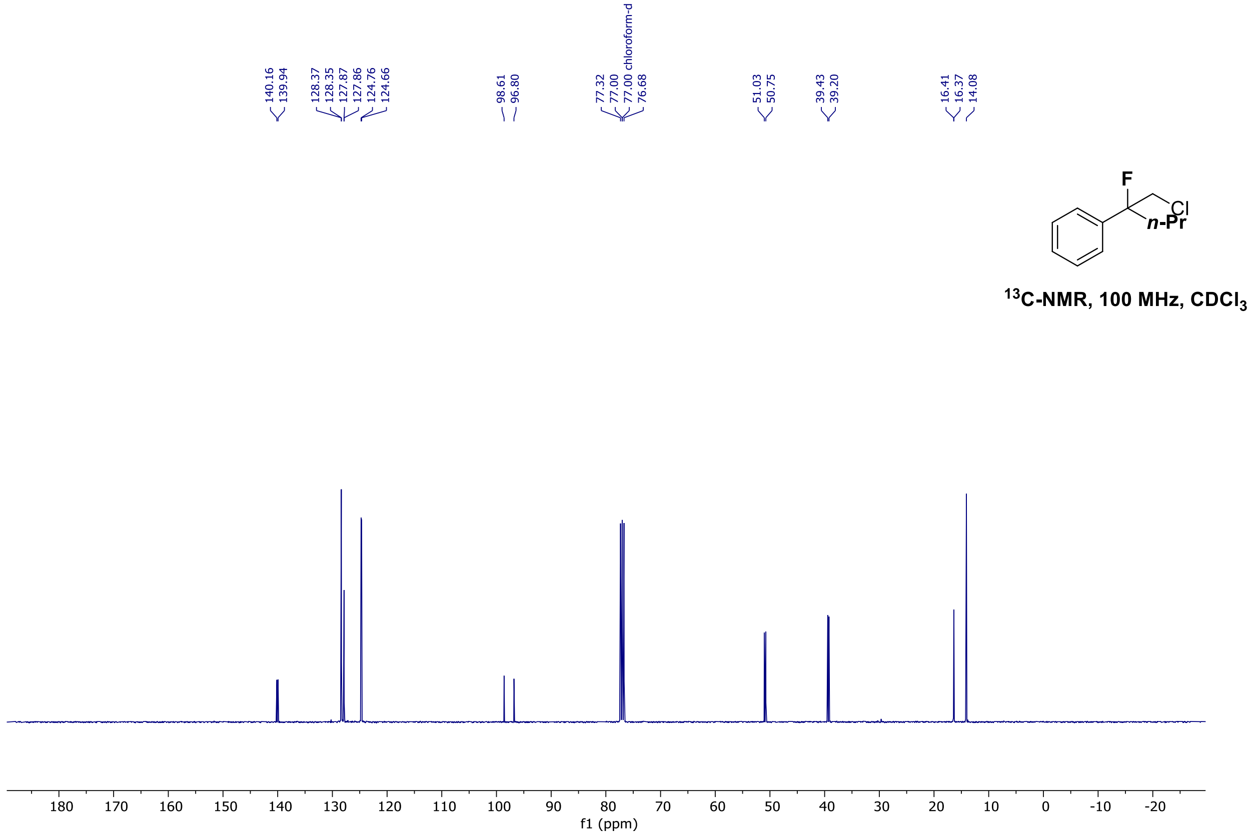

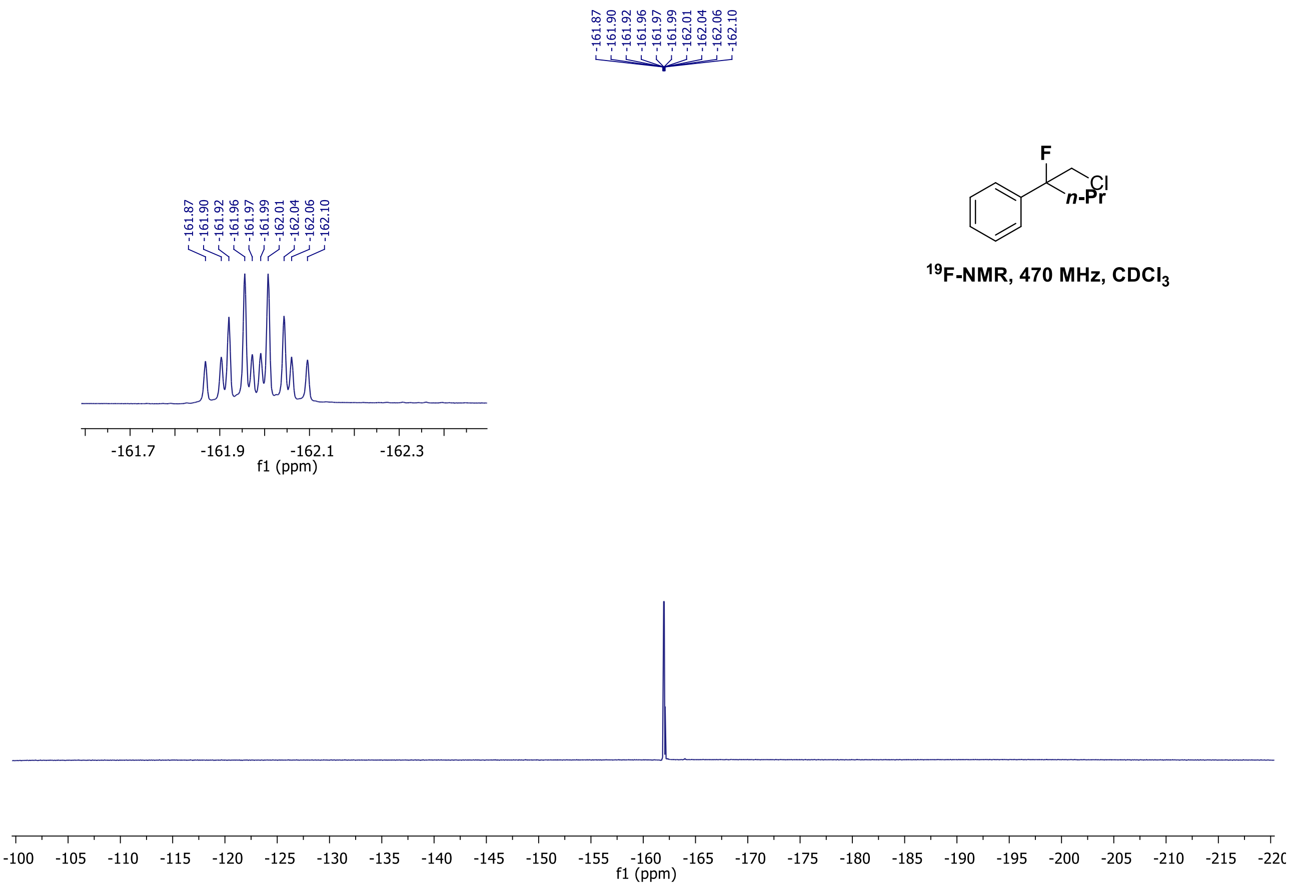
-161.87
-161.90
-161.92
-161.96
-161.97
-161.99
-162.01
-162.04
-162.06
-162.10
-161.7
-161.9
-162.1
-162.3
f1 (ppm)
F
Cl
n-Pr
19F-NMR, 470 MHz, CDCl3
-100
-105
-110
-115
-120
-125
-130
-135
-140
-145
-150
-155
-160
-165
-170
-175
-180
-185
-190
-195
-200
-205
-210
-215
-220
f1 (ppm)

Compound 5

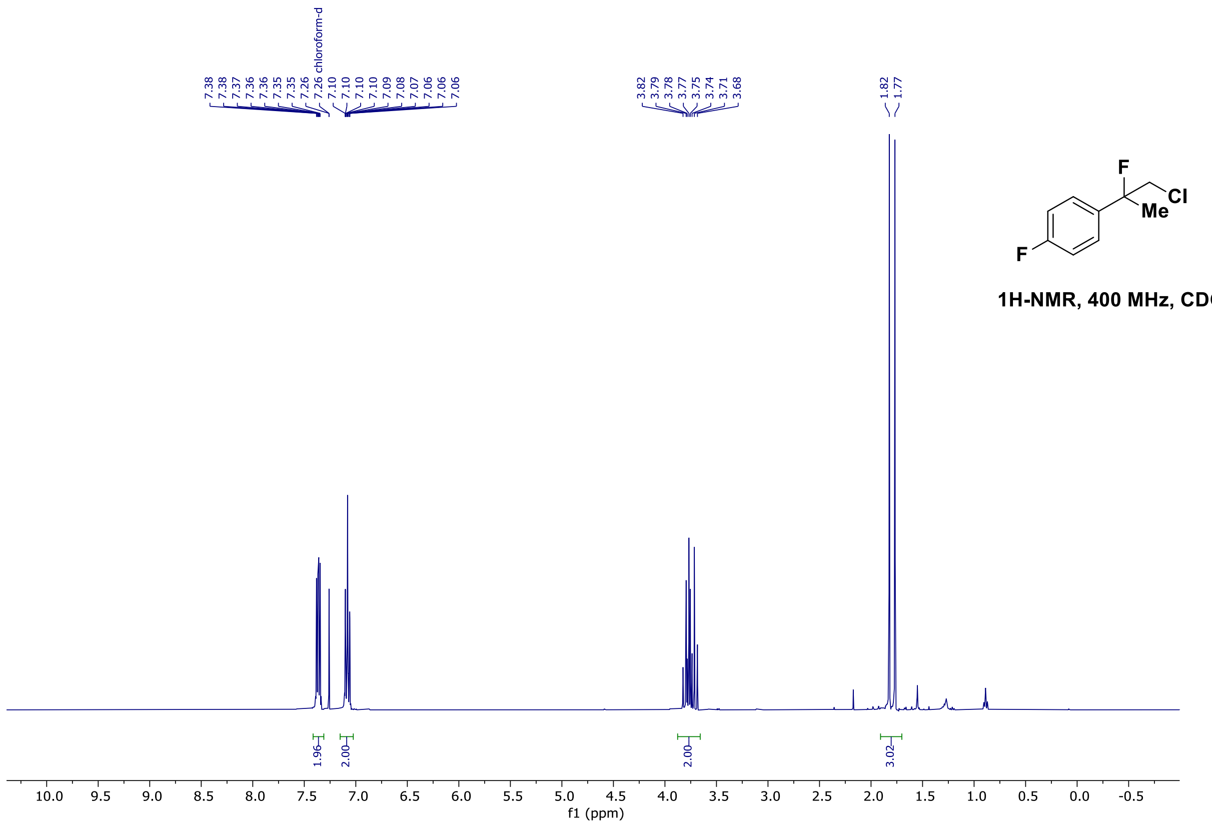

1H-NMR, 400 MHz, CDCl3

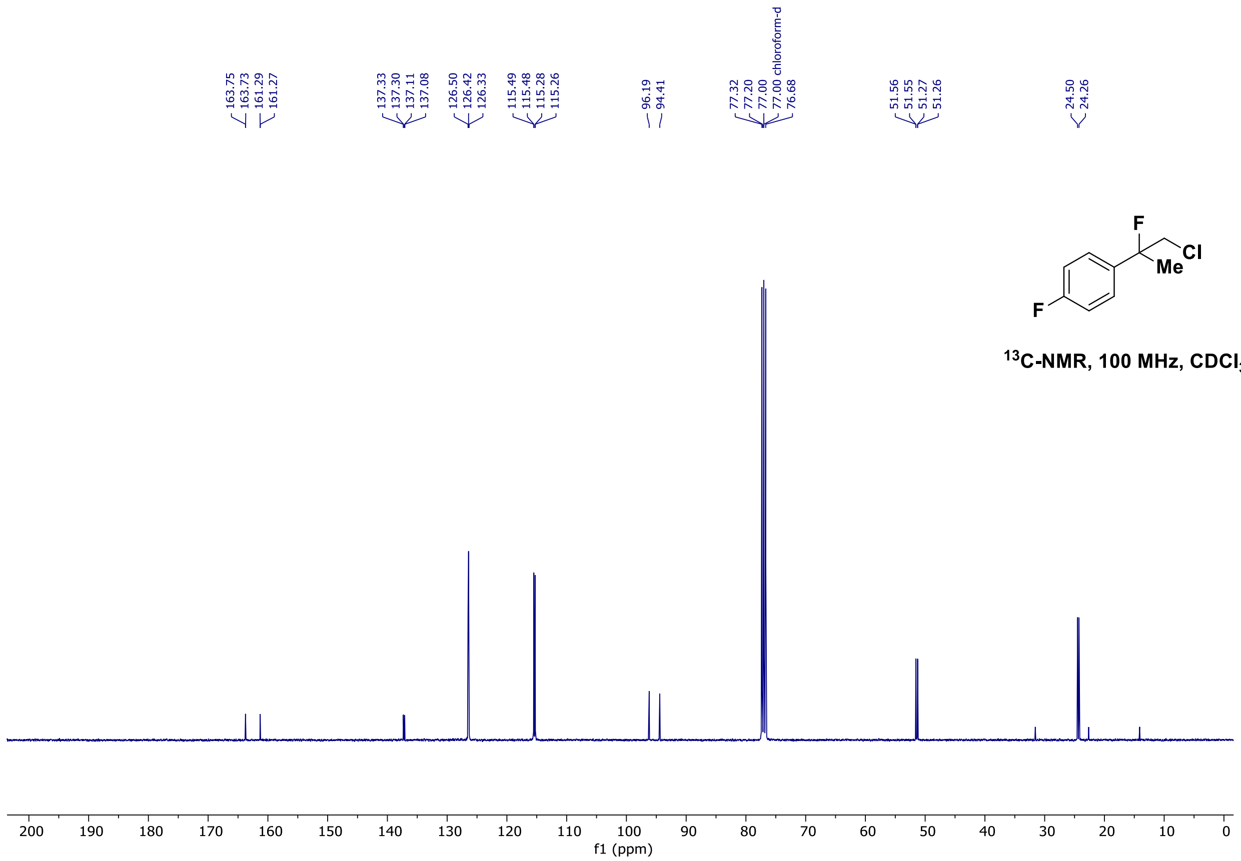

^{13}C-NMR, 100 MHz, $CDCl_3$

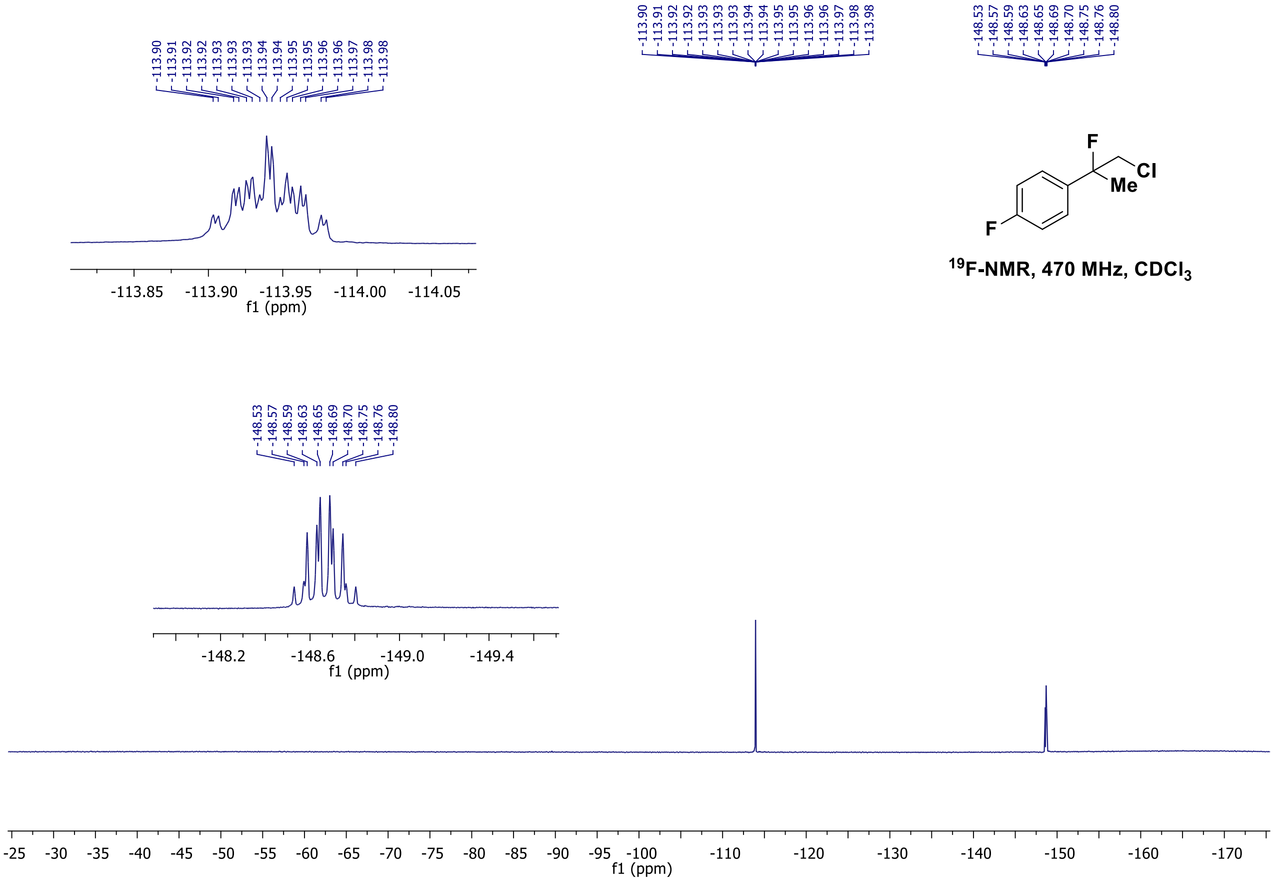
^{19}F-NMR, 470 MHz, CDCl$_3$
F
Cl
Me
F
f1 (ppm)

Compound 6

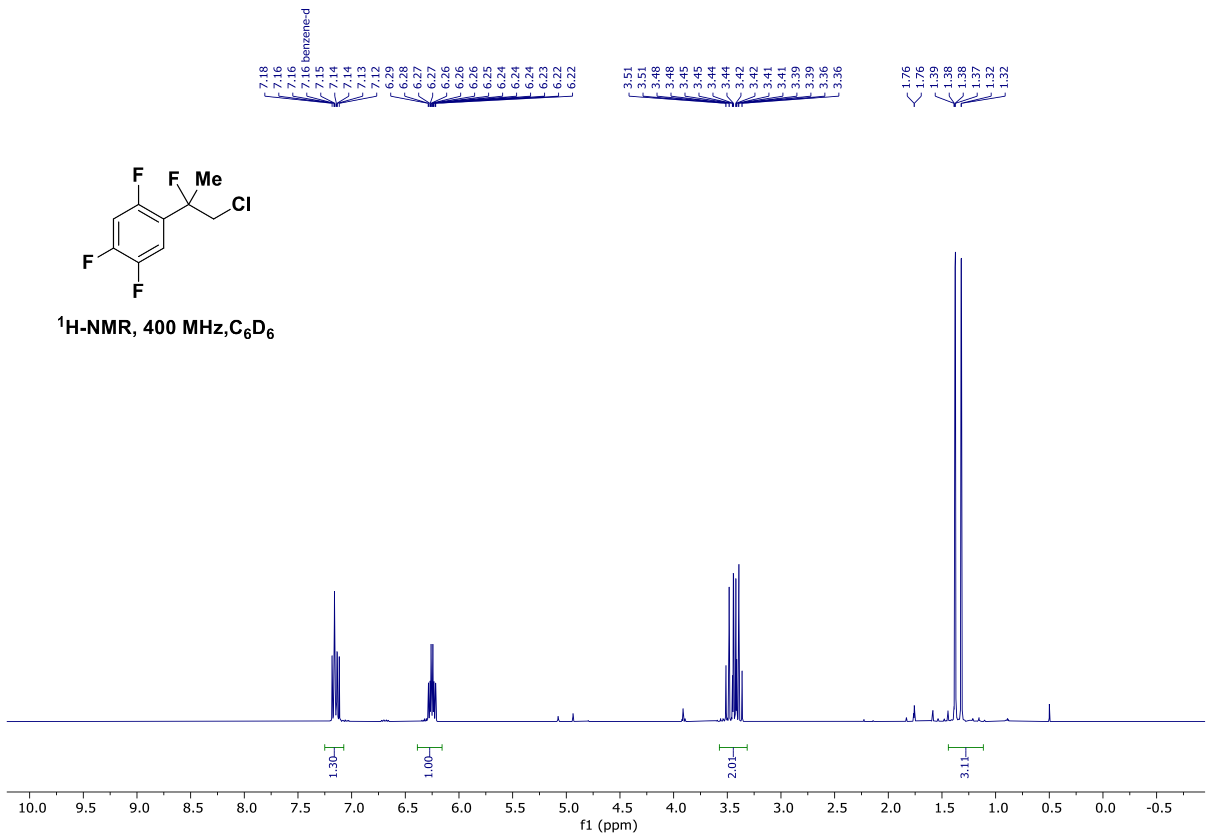

F
F
Me
Cl
F
F
¹H-NMR, 400 MHz, C6D6
f1 (ppm)

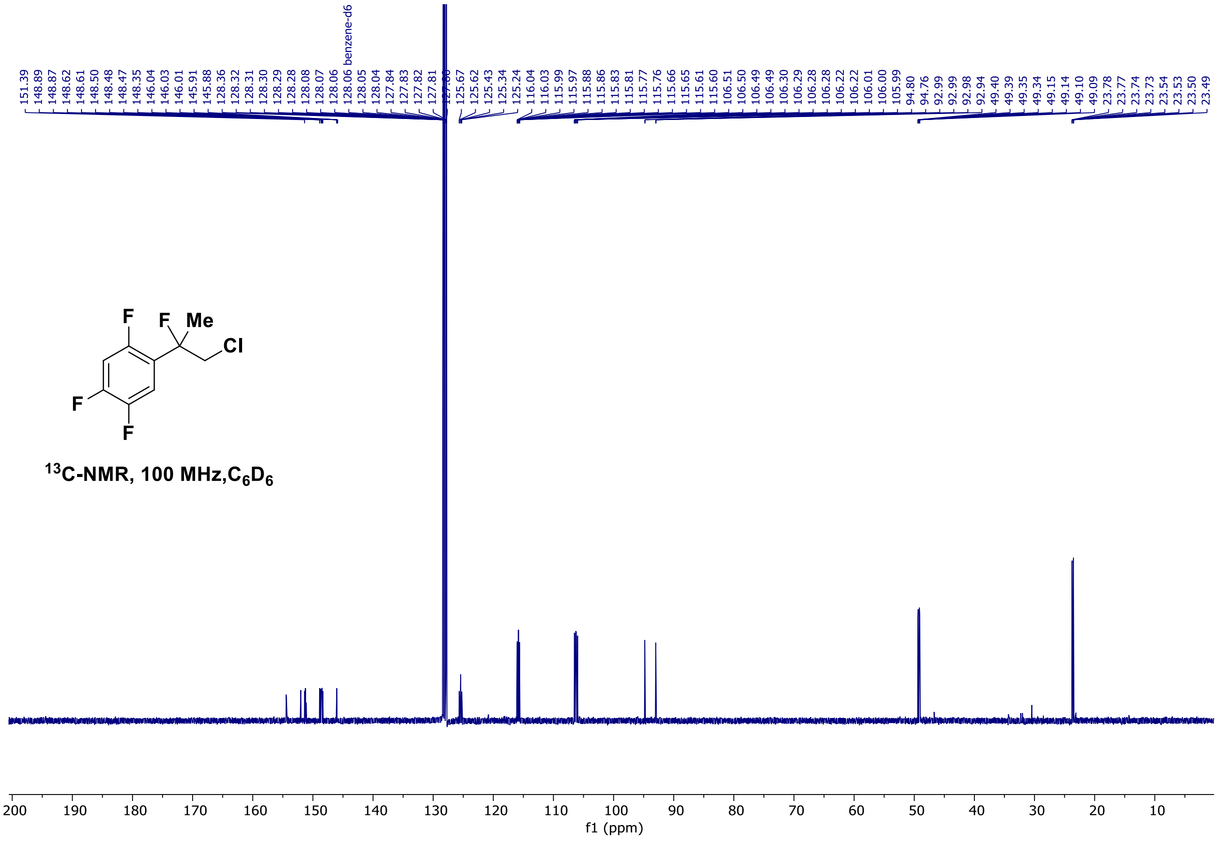

F
F
Me
Cl
F
F
¹³C-NMR, 100 MHz, C6D6
f1 (ppm)

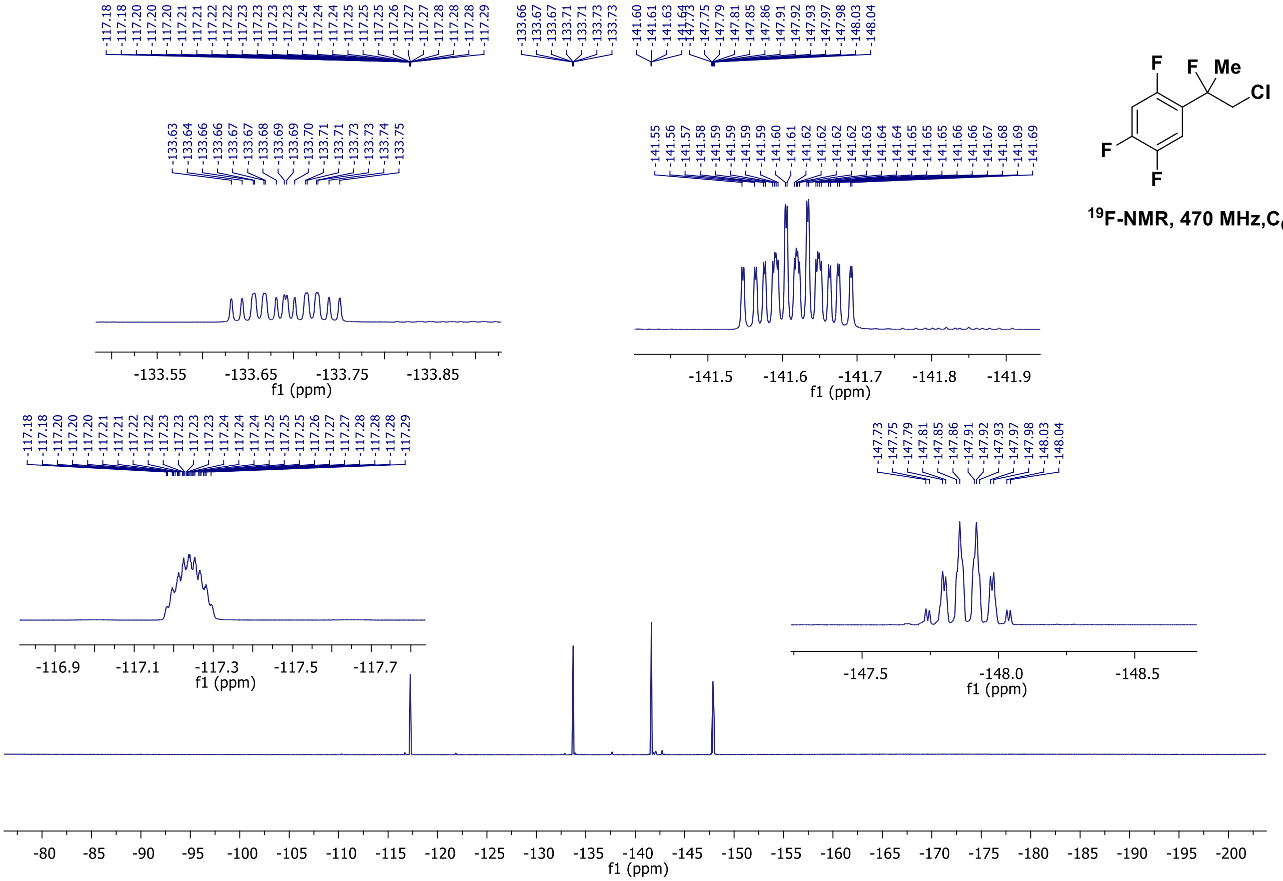

^{19}F-NMR, 470 MHz, C_6D_6

Compound 7

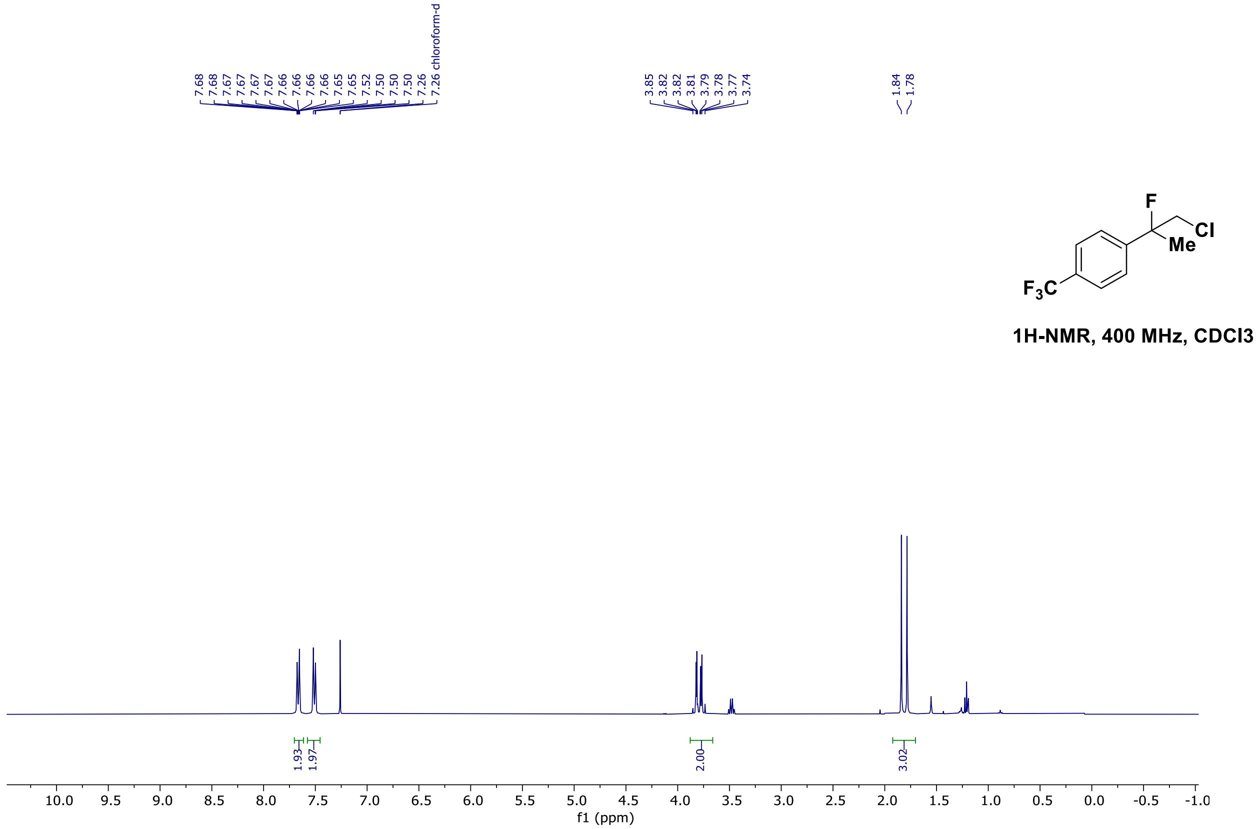

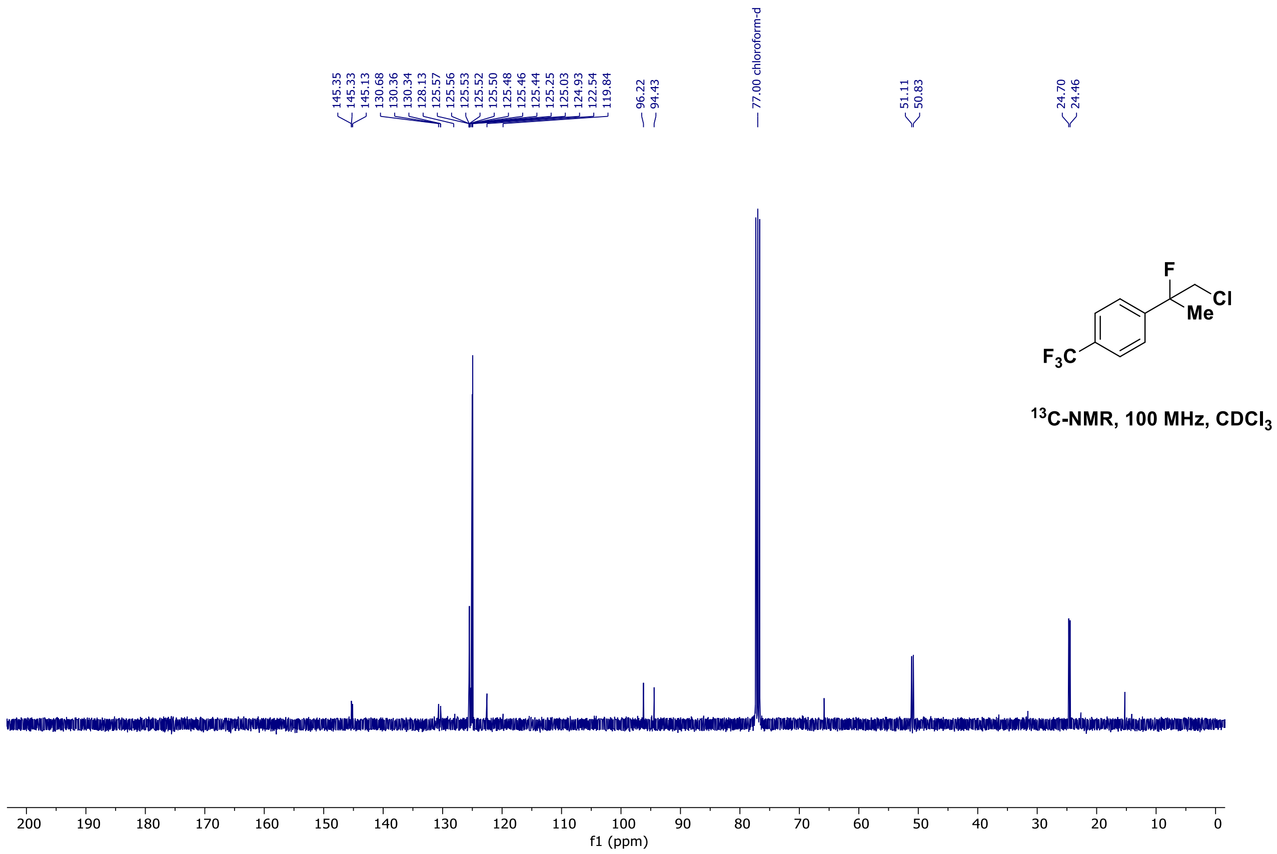
145.35
145.33
145.13
130.68
130.36
130.34
128.13
125.57
125.56
125.53
125.52
125.50
125.48
125.46
125.44
125.25
125.03
124.93
122.54
119.84
96.22
94.43
77.00 chloroform-d
51.11
50.83
24.70
24.46
F
Cl
Me
F_3C
^{13}C-NMR, 100 MHz, $CDCl_3$
200 190 180 170 160 150 140 130 120 110 100 90 80 70 60 50 40 30 20 10 0
f1 (ppm)

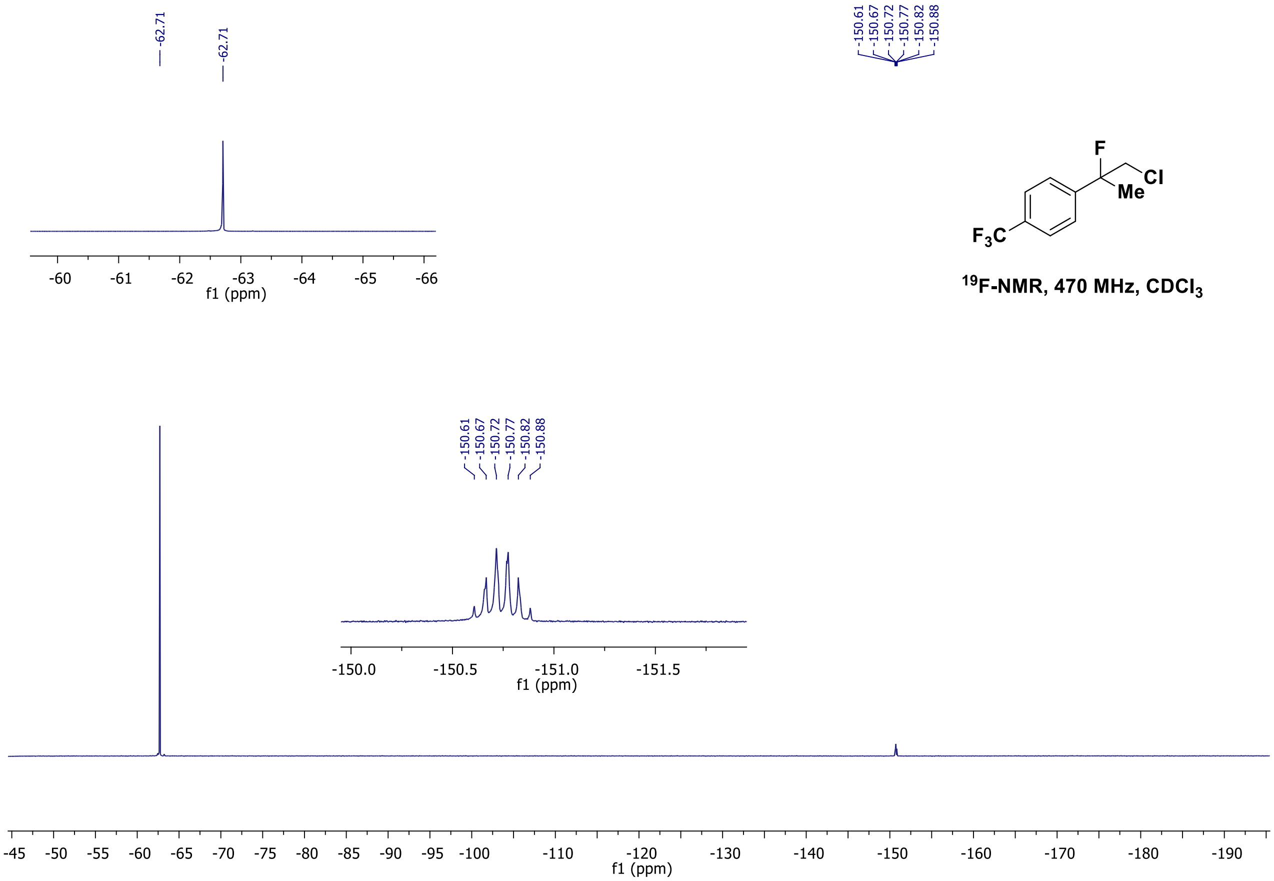
-62.71
-62.71
-150.61
-150.67
-150.72
-150.77
-150.82
-150.88
F
Cl
Me
F3C
19F-NMR, 470 MHz, CDCl3
f1 (ppm)

Compound 8

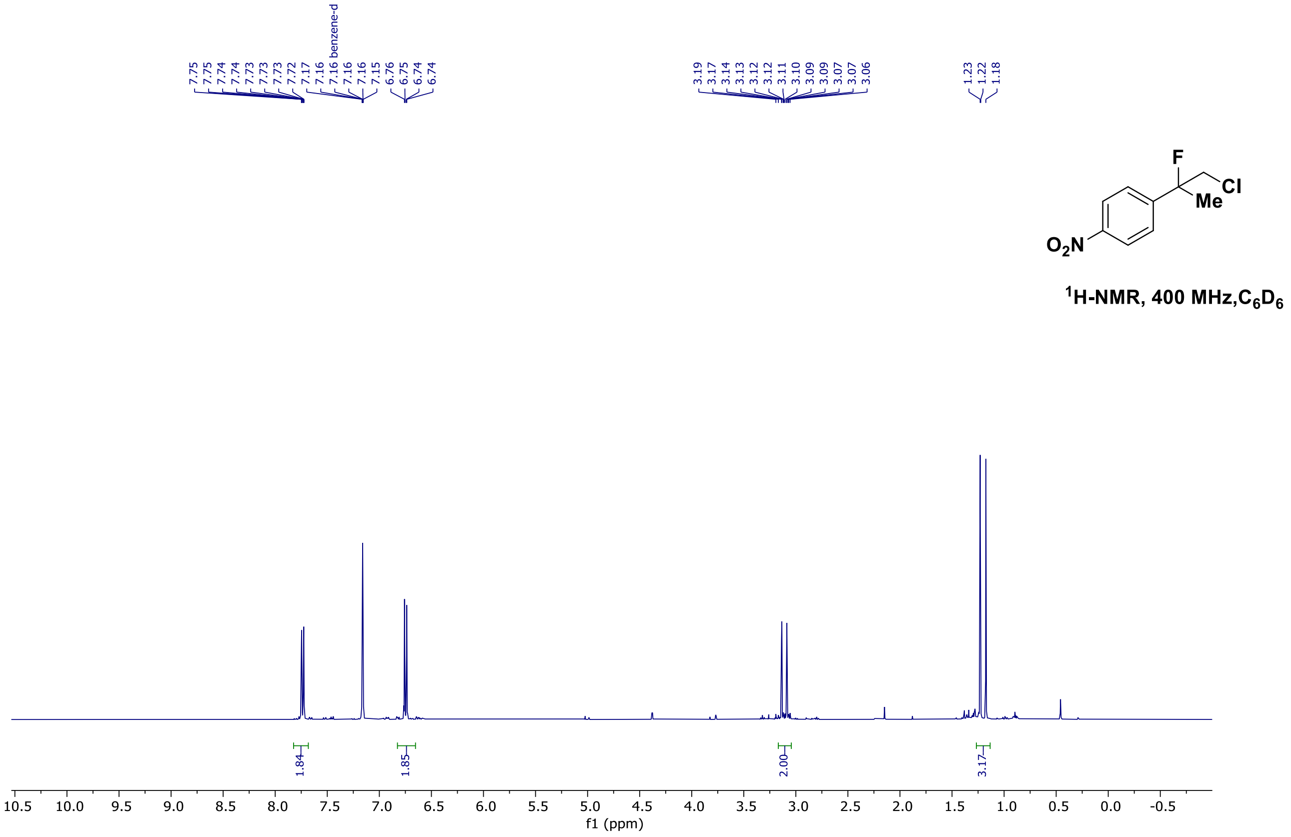

7.75
7.75
7.74
7.74
7.73
7.73
7.73
7.72
7.17
7.16 benzene-d
7.16
7.16
7.15
6.76
6.75
6.74
6.74
3.19
3.17
3.14
3.13
3.12
3.11
3.10
3.09
3.07
3.06
1.23
1.22
1.18
F
Cl
Me
O_2N
^{1}H-NMR, 400 MHz,C_6D_6
1.84
1.85
2.00
3.17
f1 (ppm)

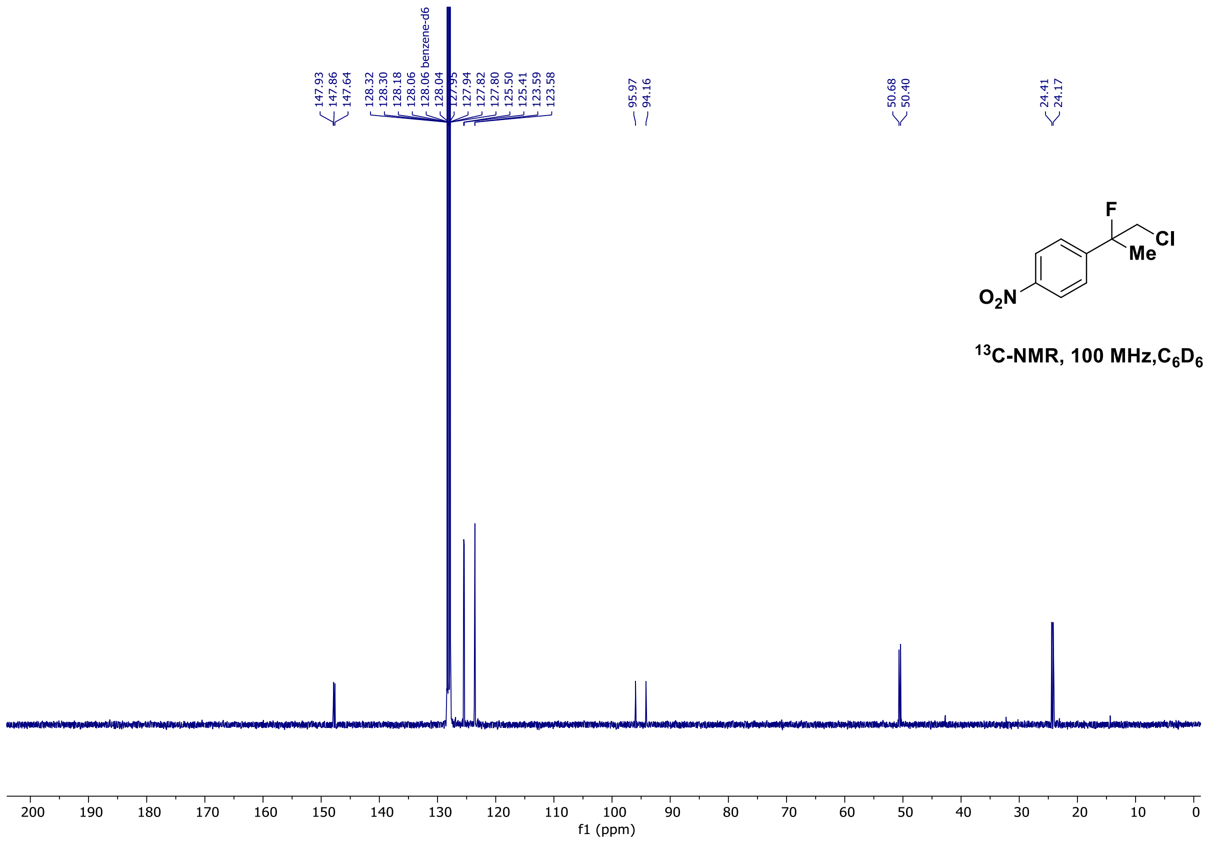
147.93
147.86
147.64
128.32
128.30
128.18
128.06
128.06 benzene-d6
128.04
127.95
127.94
127.82
127.80
125.50
125.41
123.59
123.58
95.97
94.16
50.68
50.40
24.41
24.17
F
Cl
Me
O2N
13C-NMR, 100 MHz,C6D6
f1 (ppm)

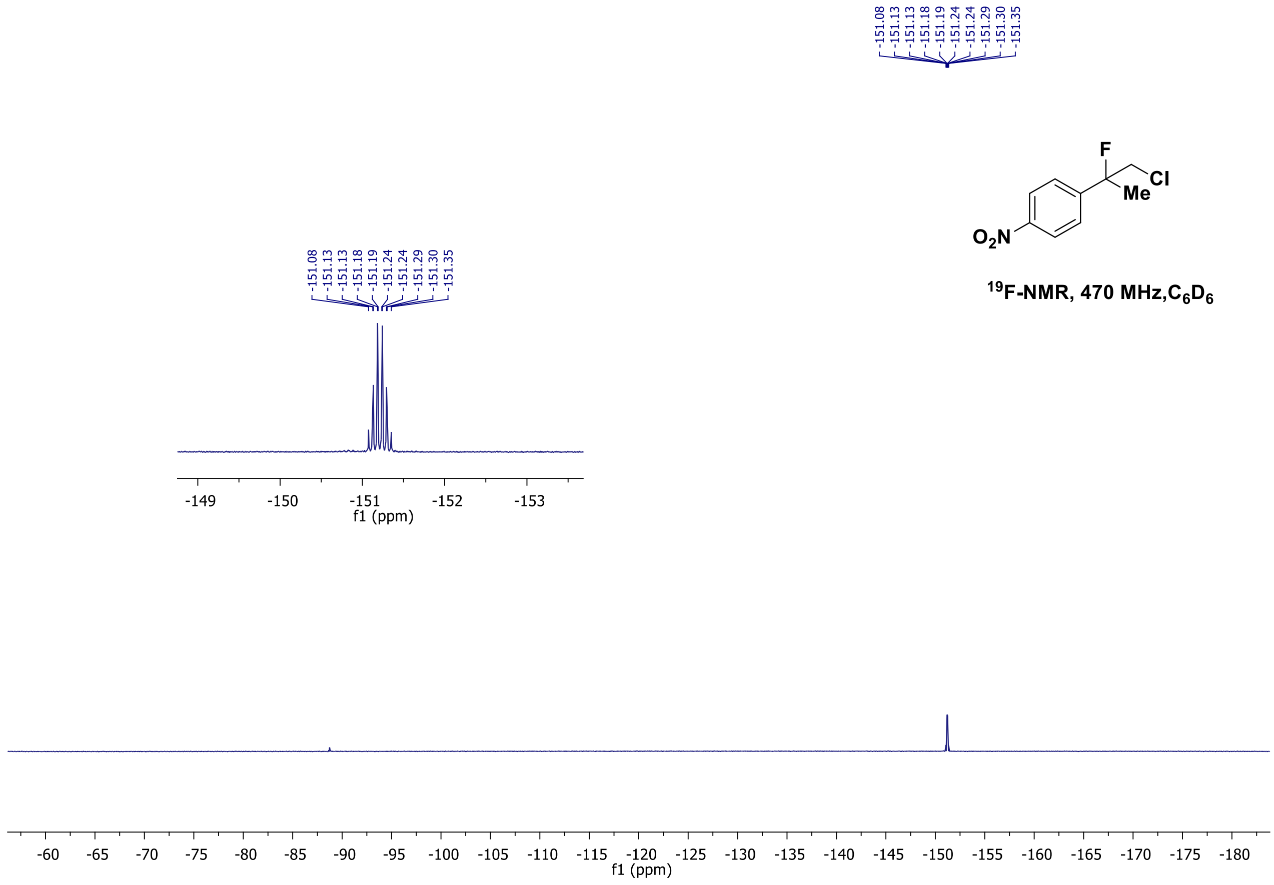
-151.08
-151.13
-151.13
-151.18
-151.19
-151.24
-151.24
-151.29
-151.30
-151.35
F
Cl
Me
O2N
19F-NMR, 470 MHz,C6D6
f1 (ppm)

Compound 9

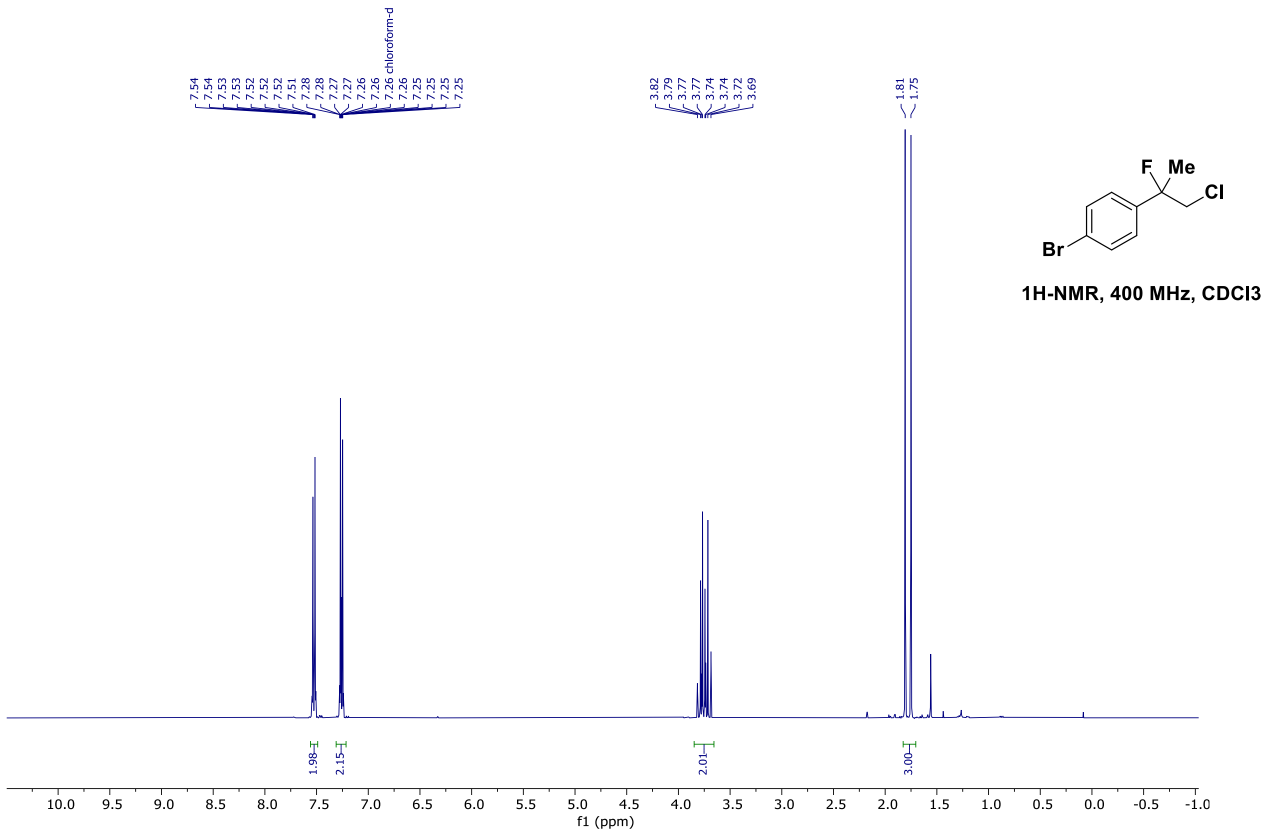
7.54
7.54
7.53
7.53
7.52
7.52
7.52
7.51
7.28
7.28
7.27
7.27
7.26
7.26
7.26 chloroform-d
7.26
7.25
7.25
7.25
7.25
3.82
3.79
3.77
3.77
3.74
3.74
3.72
3.69
1.81
1.75
F
Me
Cl
Br
1H-NMR, 400 MHz, CDCl3
1.98
2.15
2.01
3.00
10.0
9.5
9.0
8.5
8.0
7.5
7.0
6.5
6.0
5.5
5.0
4.5
4.0
3.5
3.0
2.5
2.0
1.5
1.0
0.5
0.0
-0.5
-1.0
f1 (ppm)

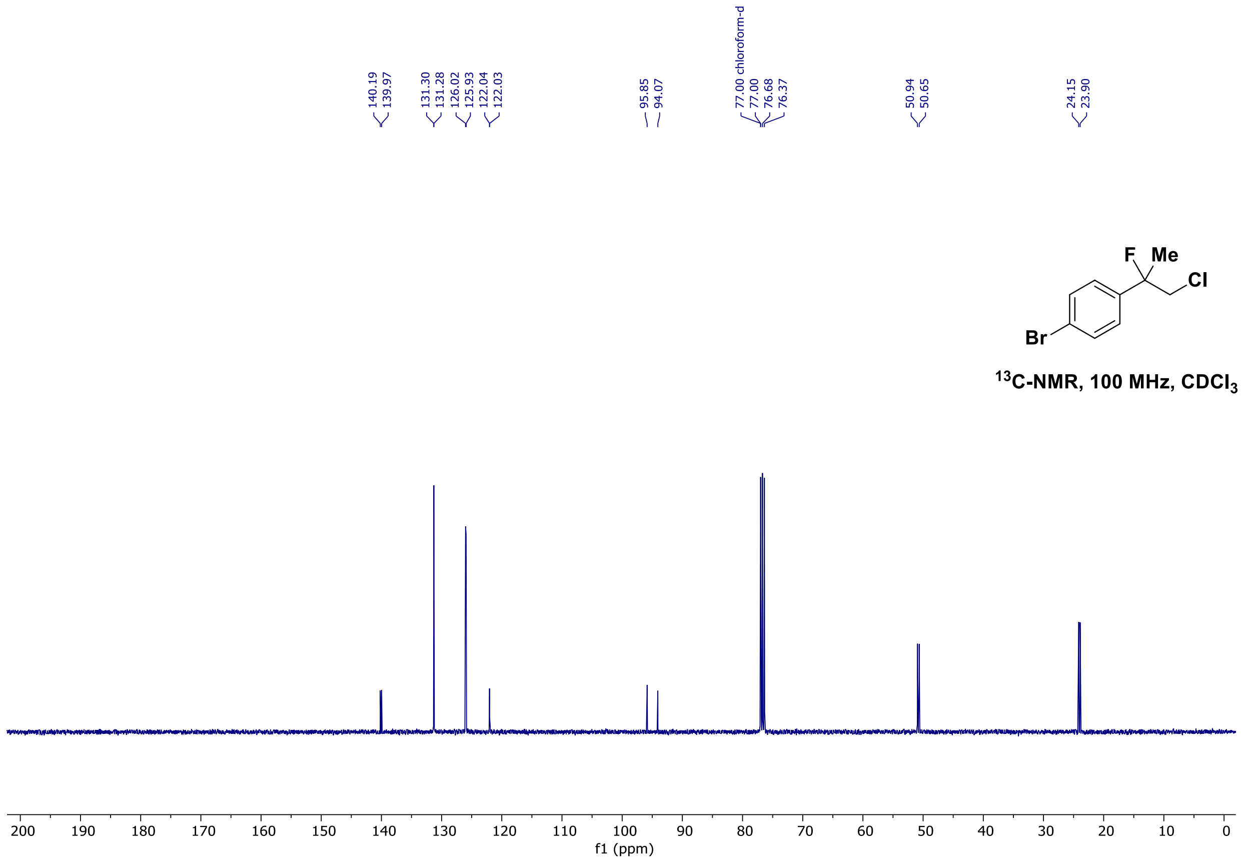
140.19
139.97
131.30
131.28
126.02
125.93
122.04
122.03
95.85
94.07
77.00 chloroform-d
77.00
76.68
76.37
50.94
50.65
24.15
23.90
F
Me
Cl
Br
^{13}C-NMR, 100 MHz, CDCl$_3$
200
190
180
170
160
150
140
130
120
110
100
90
80
70
60
50
40
30
20
10
0
f1 (ppm)

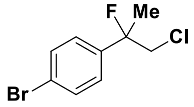

^{19}F-NMR, 470 MHz, $CDCl_3$

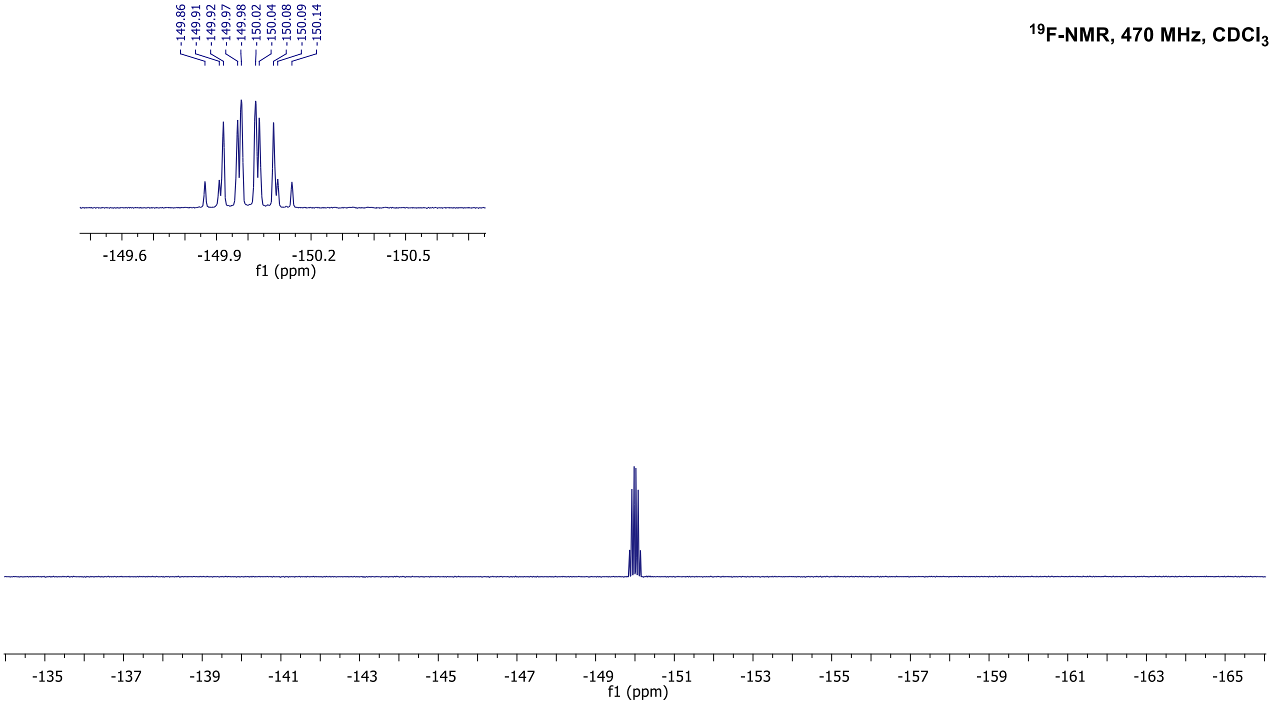

Compound 10

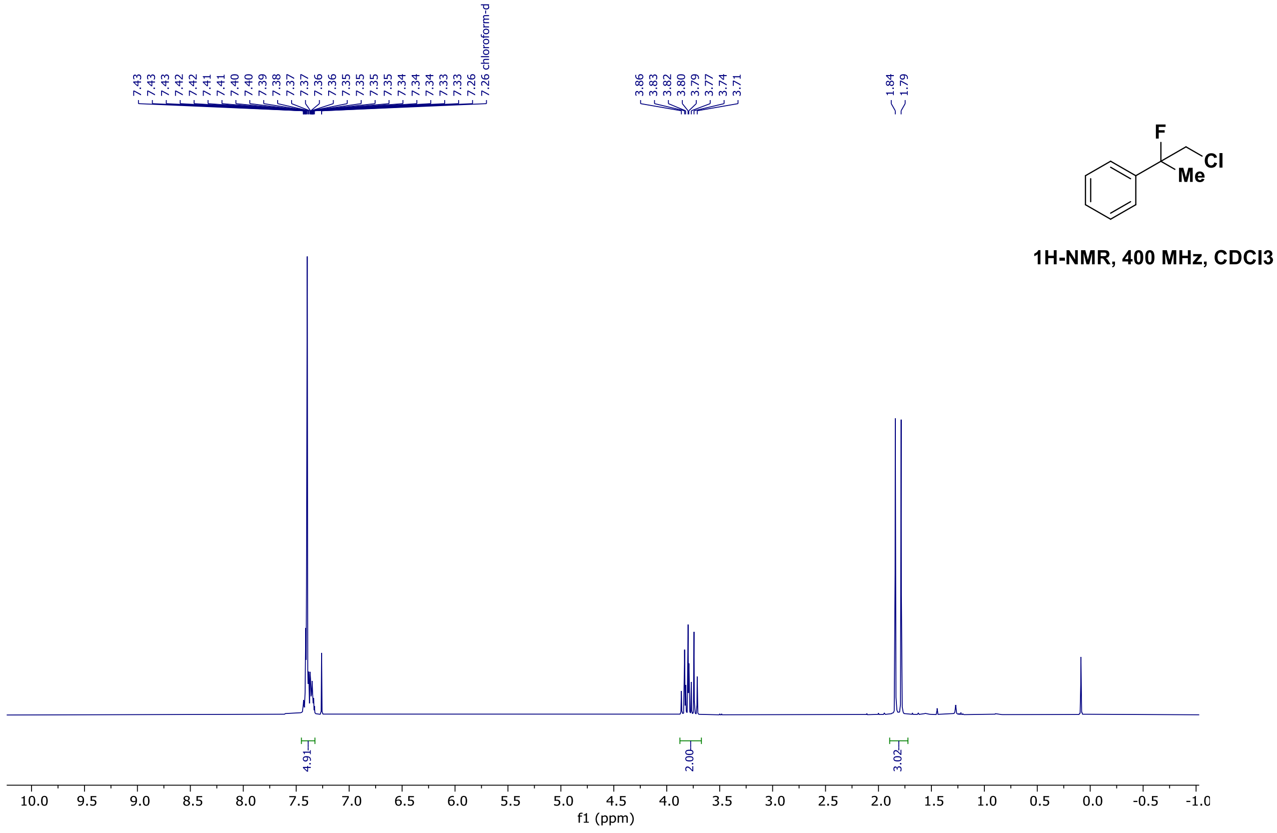

7.43
7.43
7.43
7.42
7.42
7.41
7.41
7.40
7.39
7.38
7.37
7.37
7.36
7.36
7.35
7.35
7.35
7.35
7.34
7.34
7.33
7.33
7.26 chloroform-d
3.86
3.83
3.82
3.80
3.79
3.77
3.74
3.71
1.84
1.79
F
Cl
Me
1H-NMR, 400 MHz, CDCl3
4.91
2.00
3.02
10.0
9.5
9.0
8.5
8.0
7.5
7.0
6.5
6.0
5.5
5.0
4.5
4.0
3.5
3.0
2.5
2.0
1.5
1.0
0.5
0.0
-0.5
-1.0
f1 (ppm)

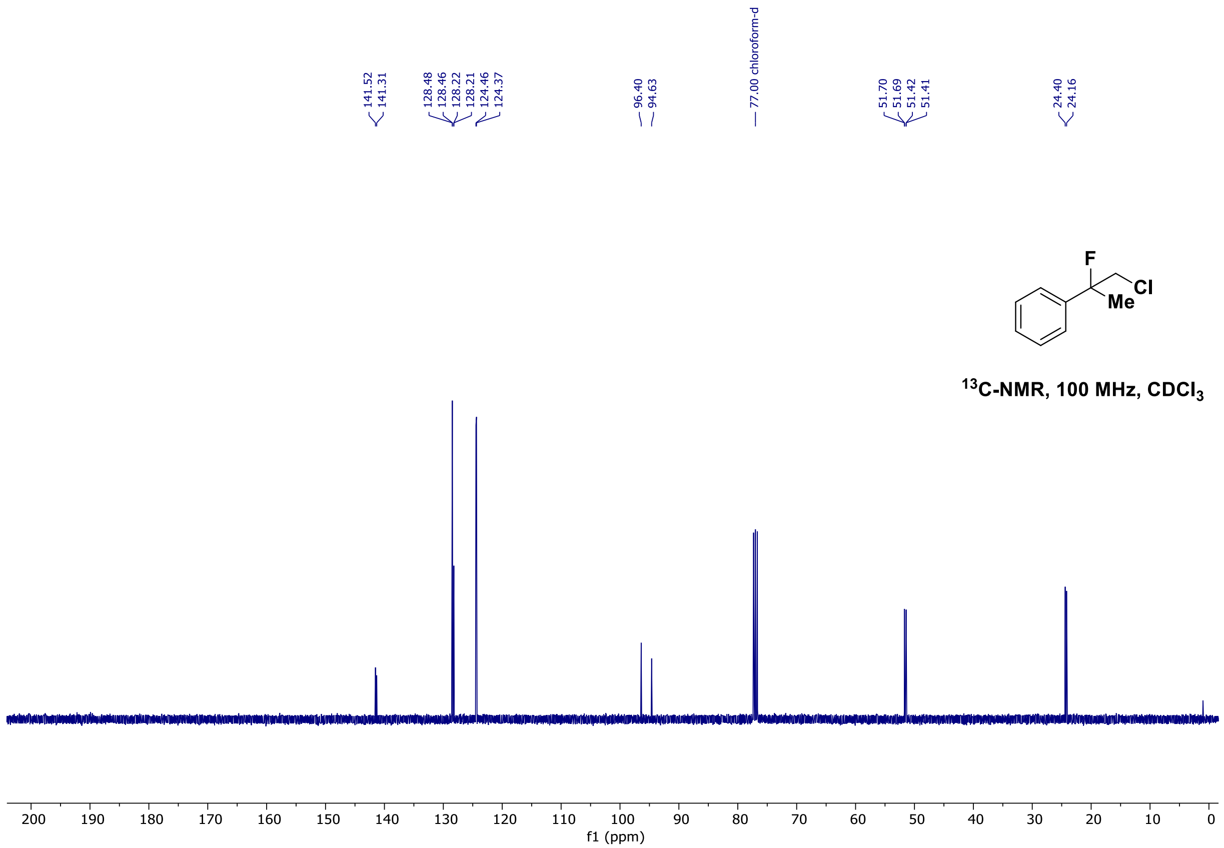

^{13}C-NMR, 100 MHz, $CDCl_3$

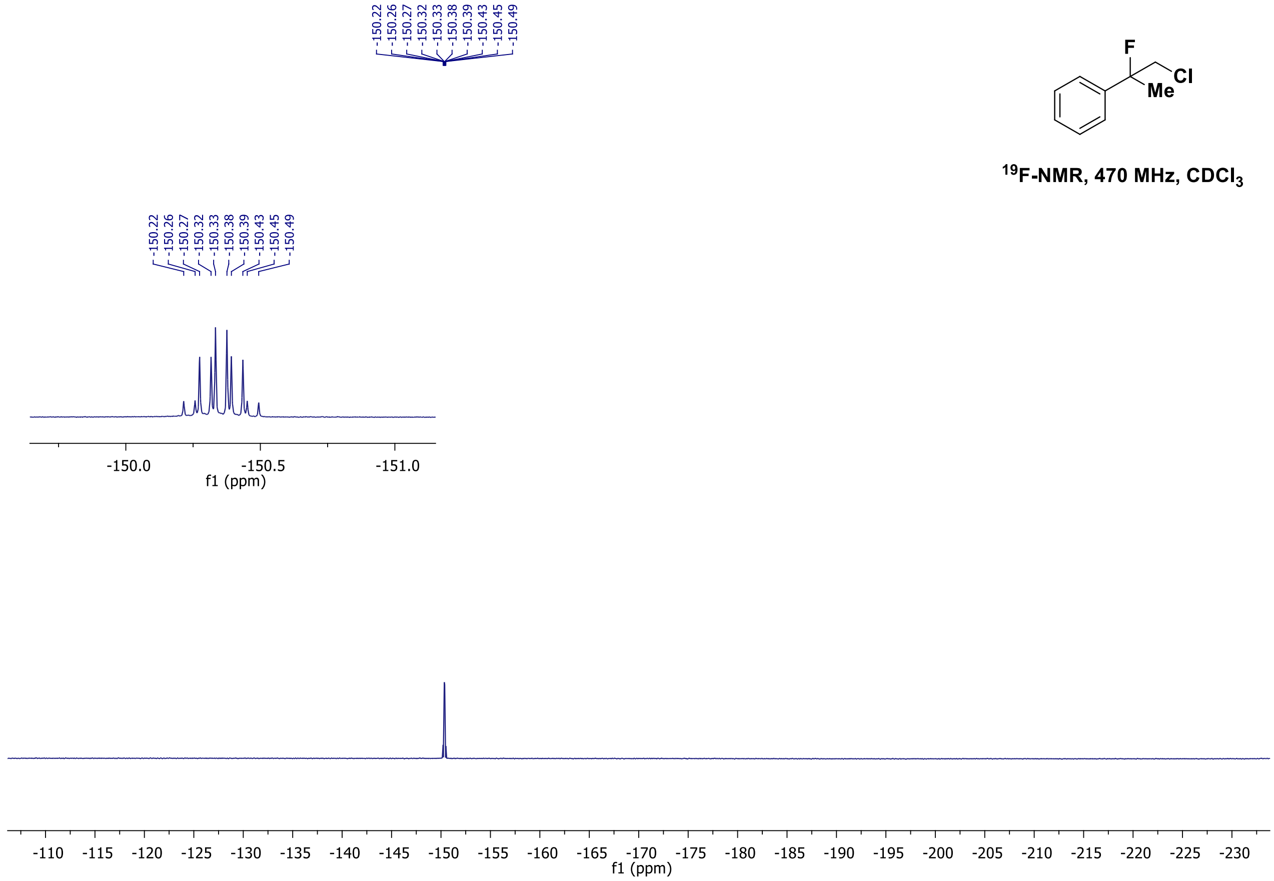

^{19}F-NMR, 470 MHz, $CDCl_3$

Compound 11

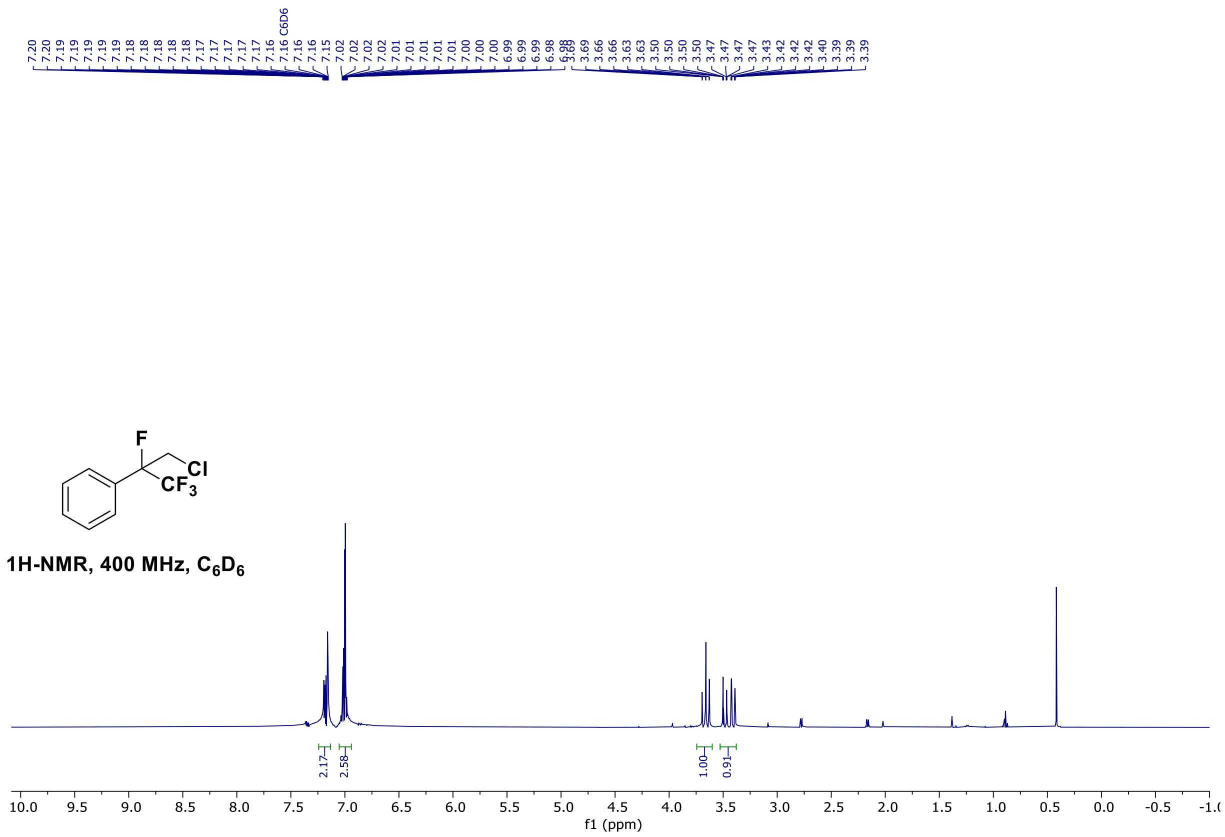
F
Cl
CF3
1H-NMR, 400 MHz, C6D6
C6D6
f1 (ppm)

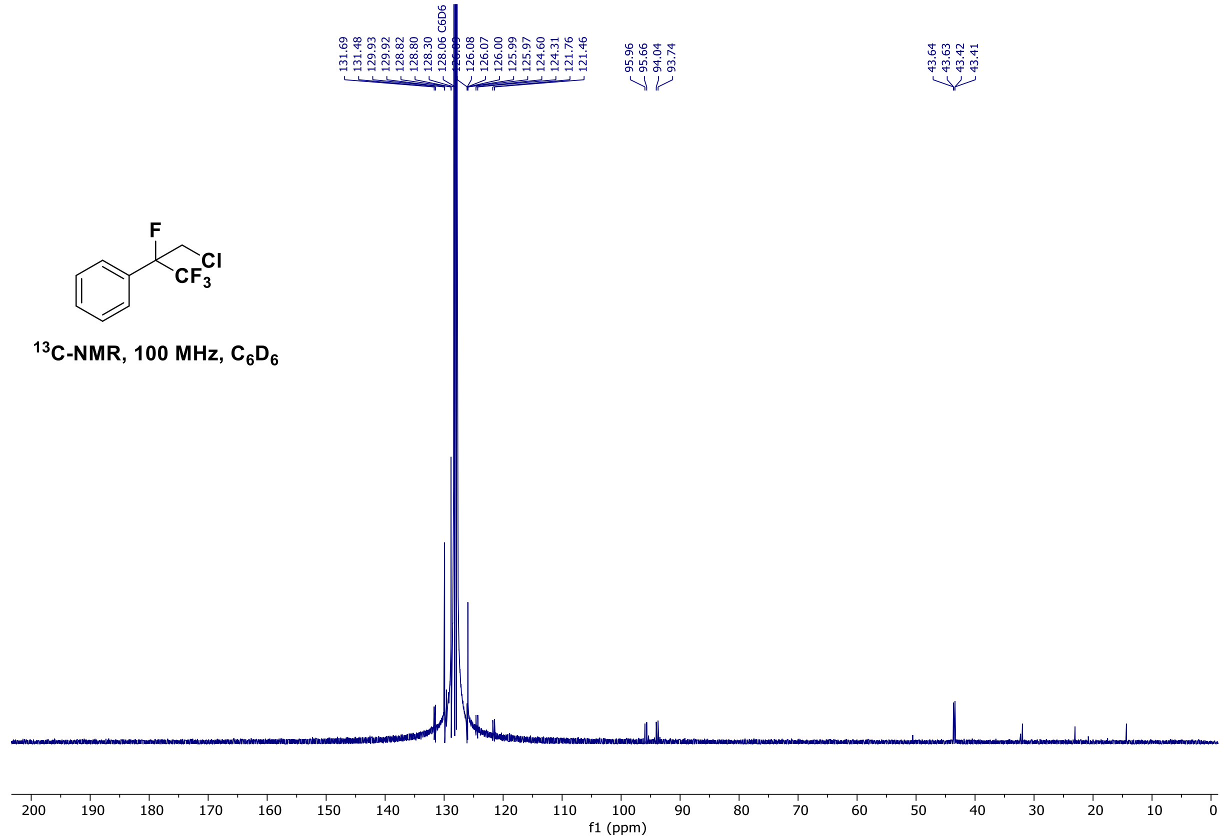
F
Cl
CF3
13C-NMR, 100 MHz, C6D6
C6D6
f1 (ppm)

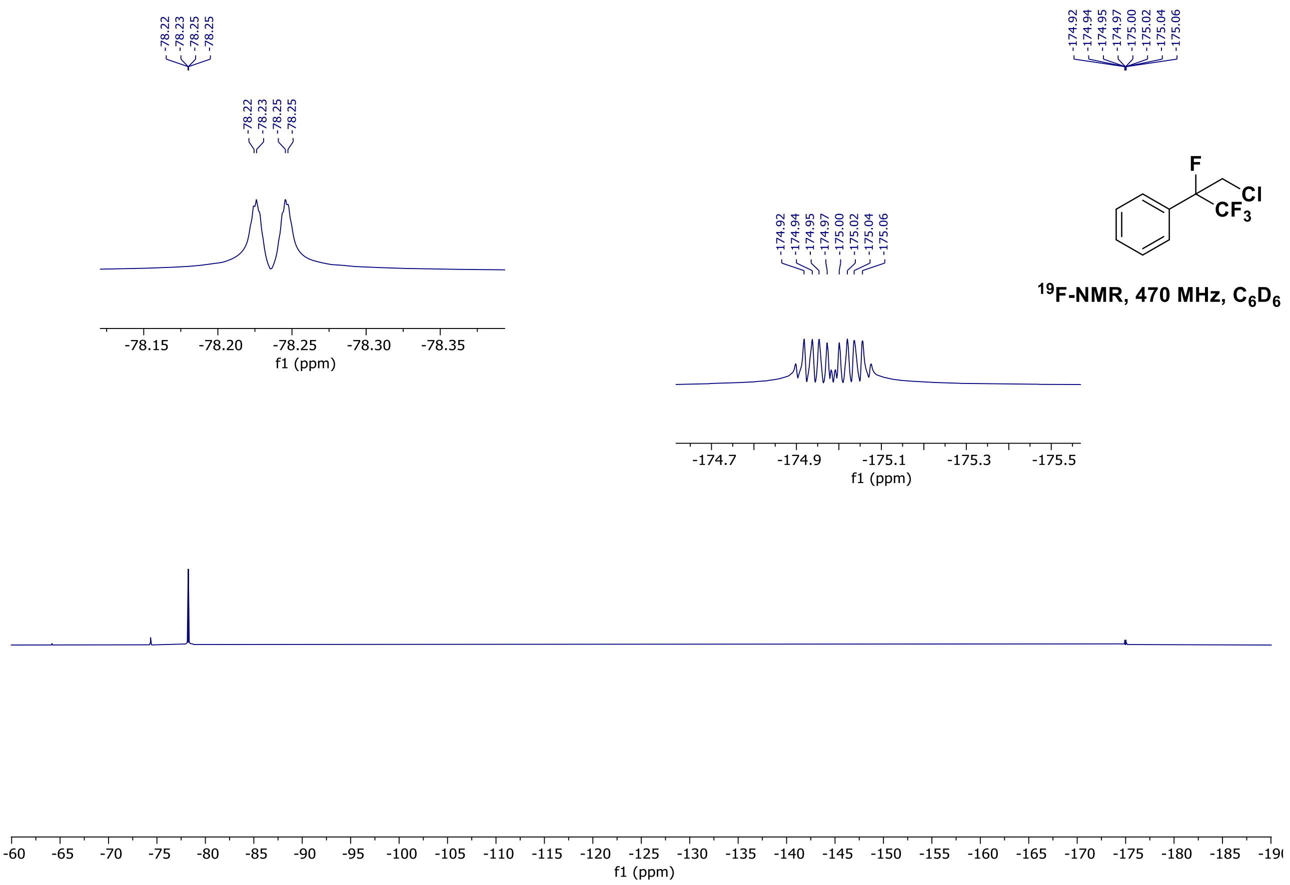

-78.22
-78.23
-78.25
-78.25
-174.92
-174.94
-174.95
-174.97
-175.00
-175.02
-175.04
-175.06
F
Cl
CF3
19F-NMR, 470 MHz, C6D6
-78.15 -78.20 -78.25 -78.30 -78.35
f1 (ppm)
-174.7 -174.9 -175.1 -175.3 -175.5
f1 (ppm)
-60 -65 -70 -75 -80 -85 -90 -95 -100 -105 -110 -115 -120 -125 -130 -135 -140 -145 -150 -155 -160 -165 -170 -175 -180 -185
f1 (ppm)

Compound 12

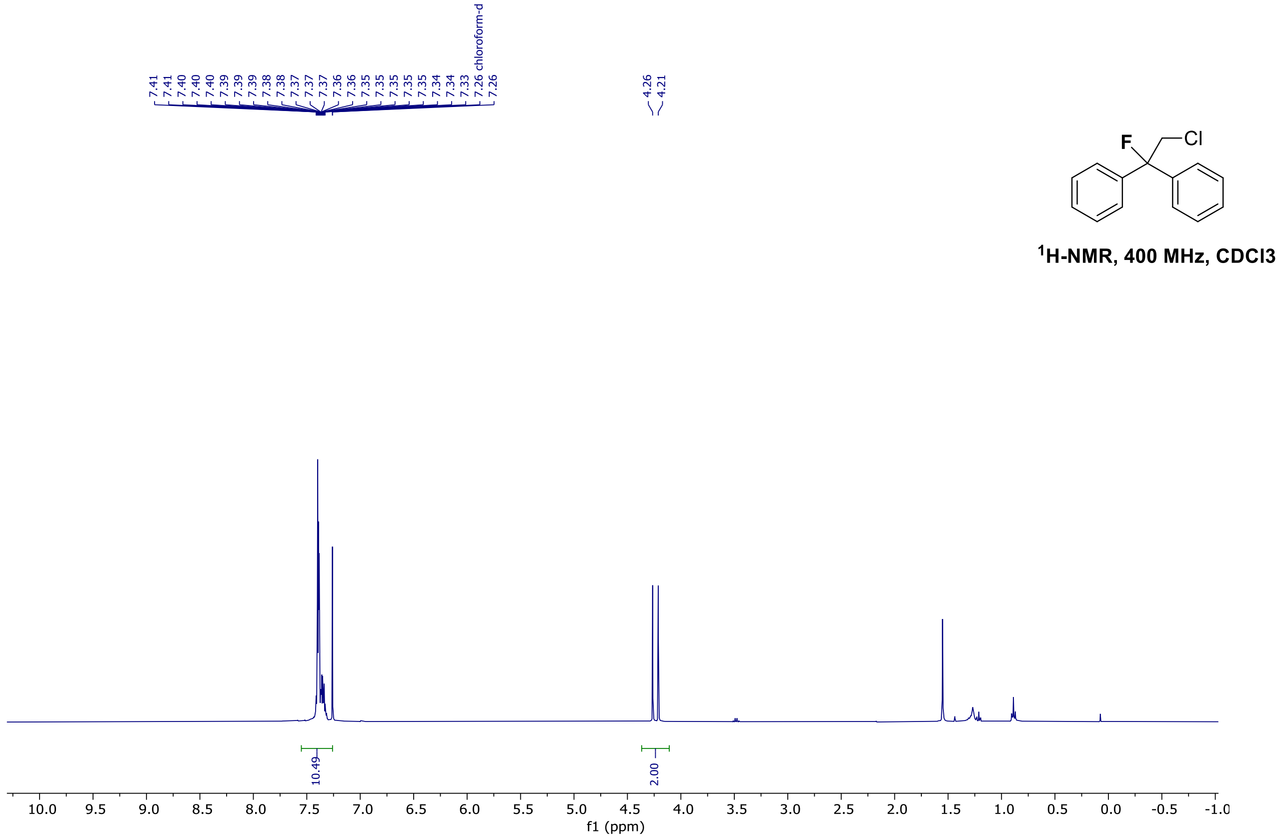

7.41 7.41 7.40 7.40 7.39 7.39 7.39 7.38 7.38 7.37 7.37 7.37 7.36 7.36 7.35 7.35 7.35 7.35 7.34 7.34 7.33 7.26 chloroform-d 7.26
4.26 4.21
F
Cl
¹H-NMR, 400 MHz, CDCl3
10.49
2.00
f1 (ppm)

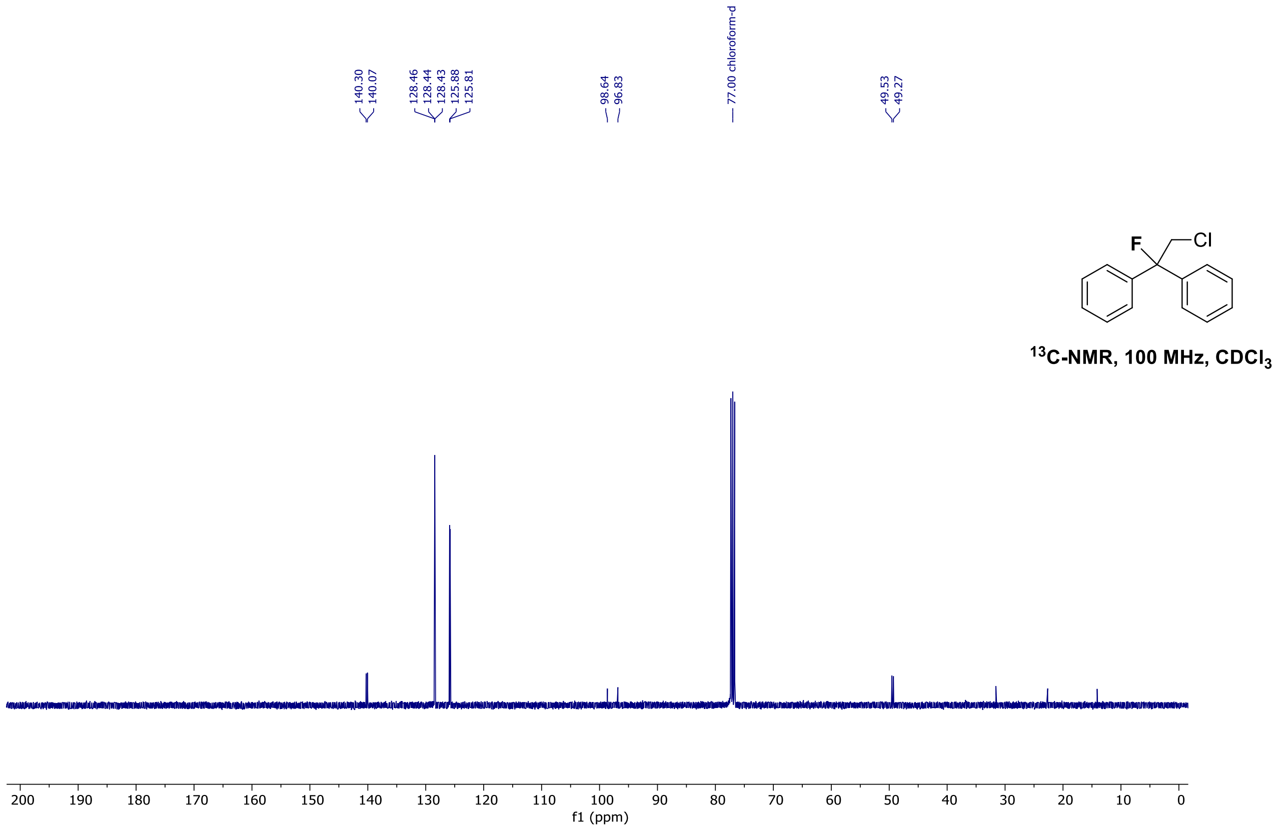

140.30 140.07
128.46 128.44 128.43 125.88 125.81
98.64 96.83
77.00 chloroform-d
49.53 49.27
F
Cl
¹³C-NMR, 100 MHz, CDCl₃
f1 (ppm)

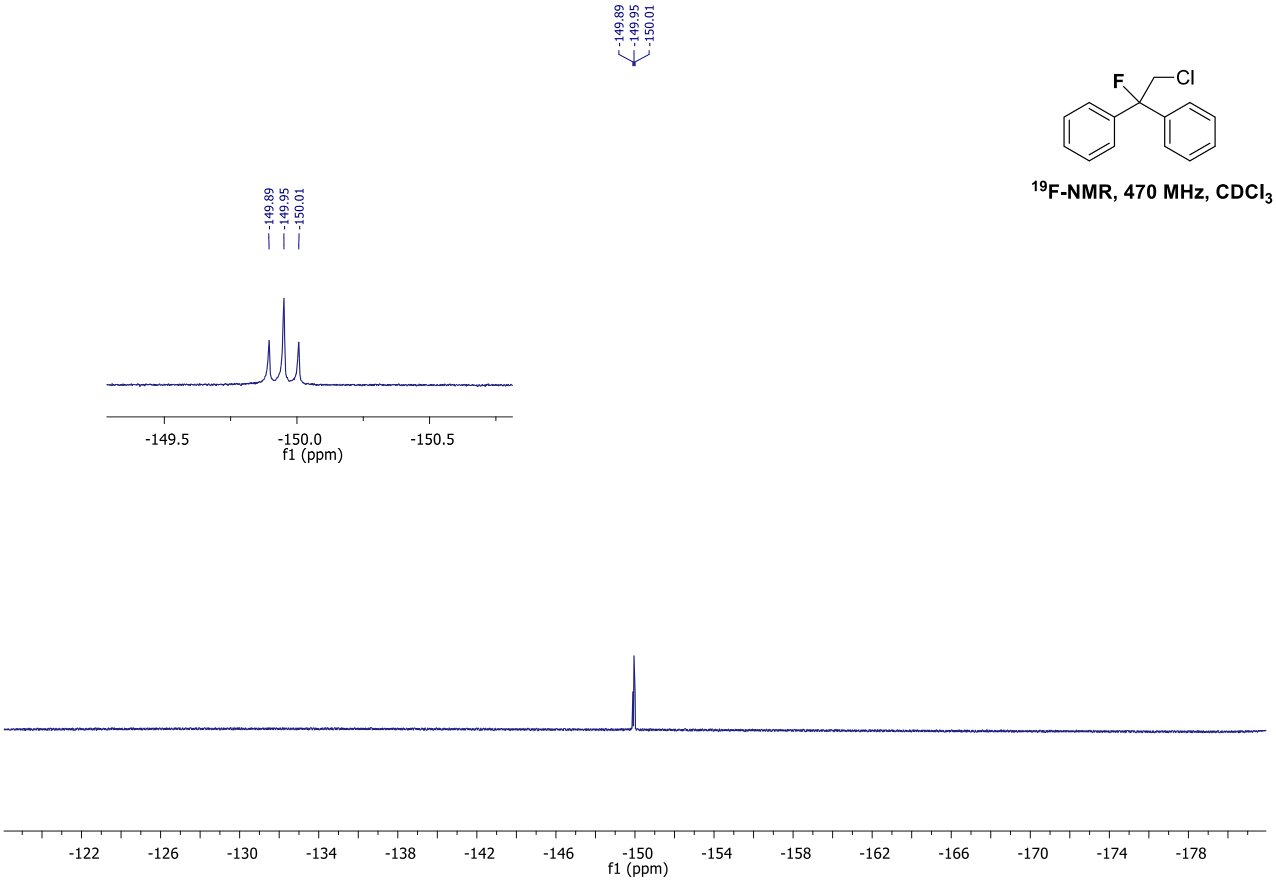

^{19}F-NMR, 470 MHz, $CDCl_3$

Compound 13

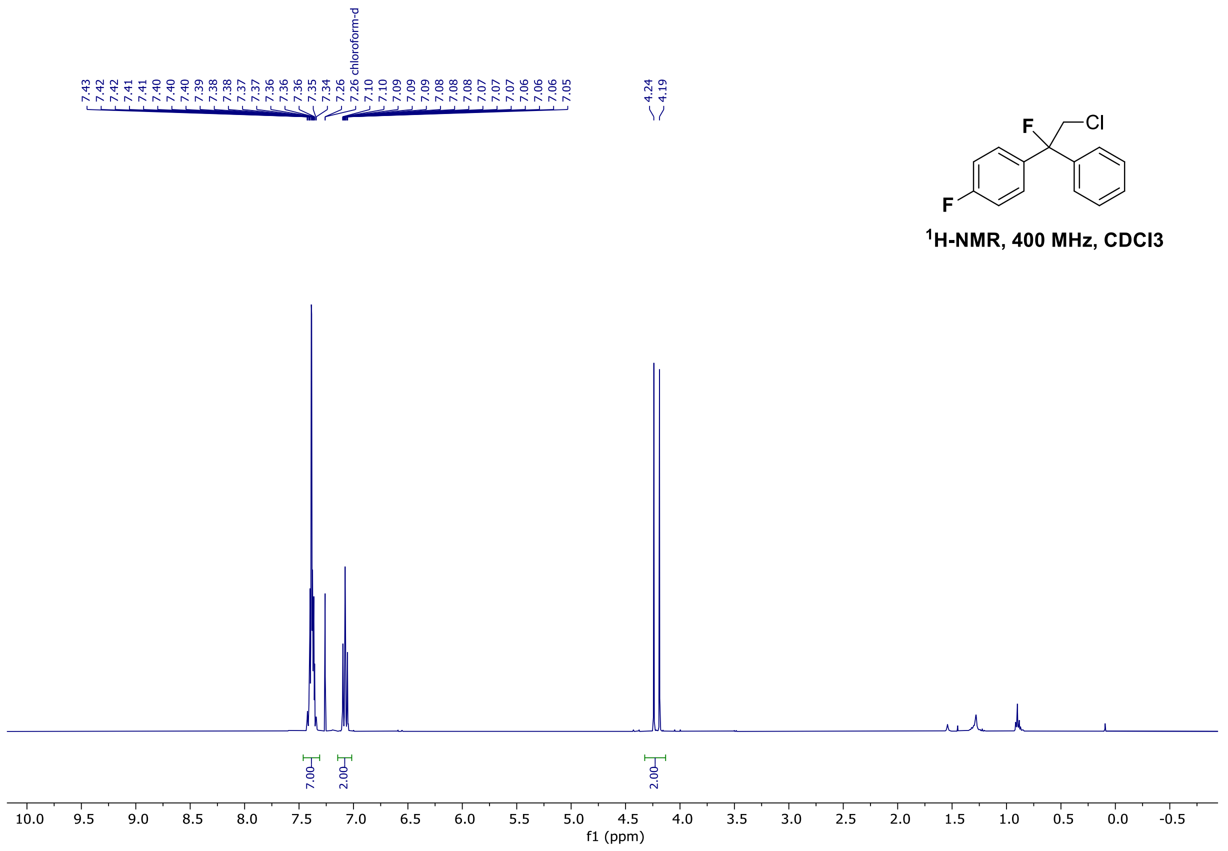
7.43
7.42
7.42
7.41
7.41
7.40
7.40
7.39
7.38
7.38
7.37
7.37
7.36
7.36
7.35
7.34
7.26 chloroform-d
7.26
7.10
7.10
7.09
7.09
7.09
7.08
7.08
7.08
7.07
7.07
7.06
7.06
7.06
7.05
4.24
4.19
F
Cl
F
^{1}H-NMR, 400 MHz, CDCl3
7.00
2.00
2.00
10.0
9.5
9.0
8.5
8.0
7.5
7.0
6.5
6.0
5.5
5.0
4.5
4.0
3.5
3.0
2.5
2.0
1.5
1.0
0.5
0.0
-0.5
f1 (ppm)

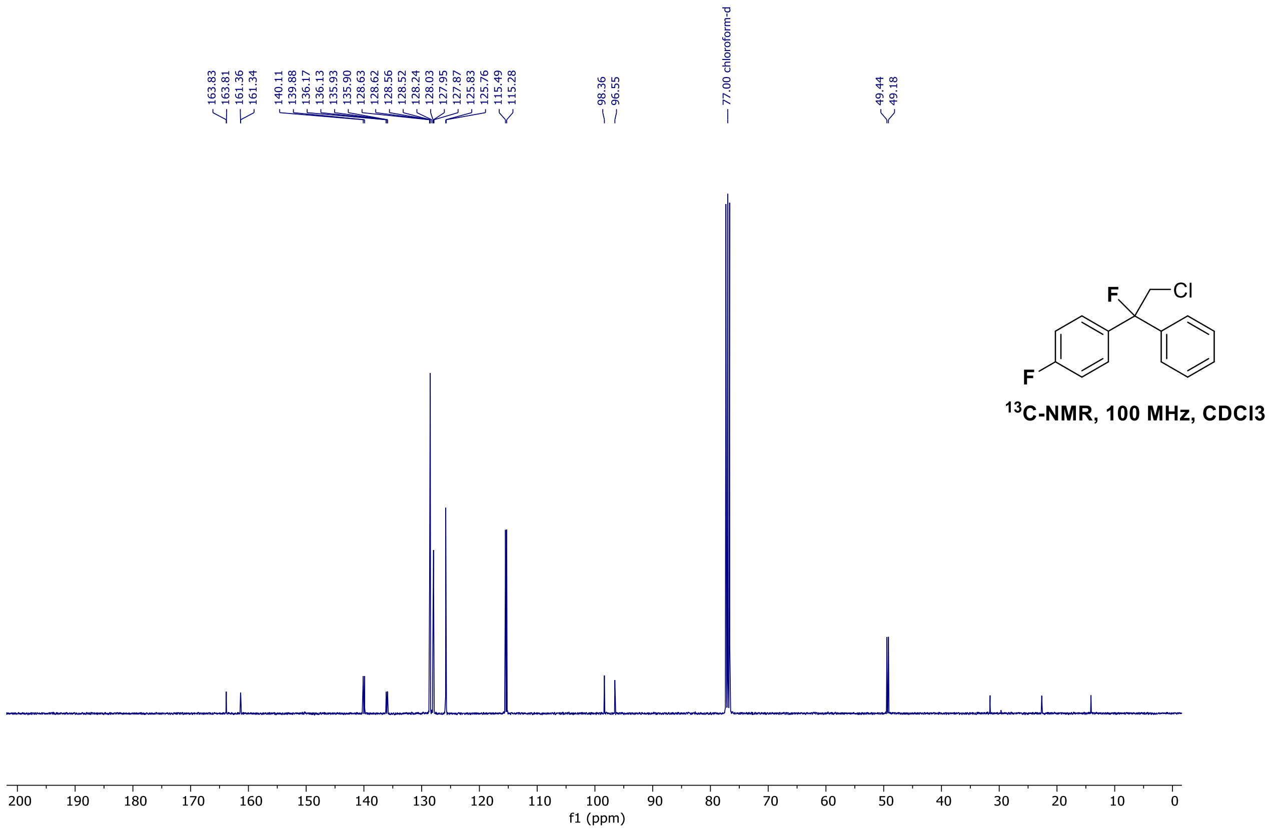
163.83
163.81
161.36
161.34
140.11
139.88
136.17
136.13
135.93
135.90
128.63
128.62
128.56
128.52
128.24
128.03
127.95
127.87
125.83
125.76
115.49
115.28
98.36
96.55
77.00 chloroform-d
49.44
49.18
F
Cl
F
^{13}C-NMR, 100 MHz, CDCl3
200
190
180
170
160
150
140
130
120
110
100
90
80
70
60
50
40
30
20
10
0
f1 (ppm)

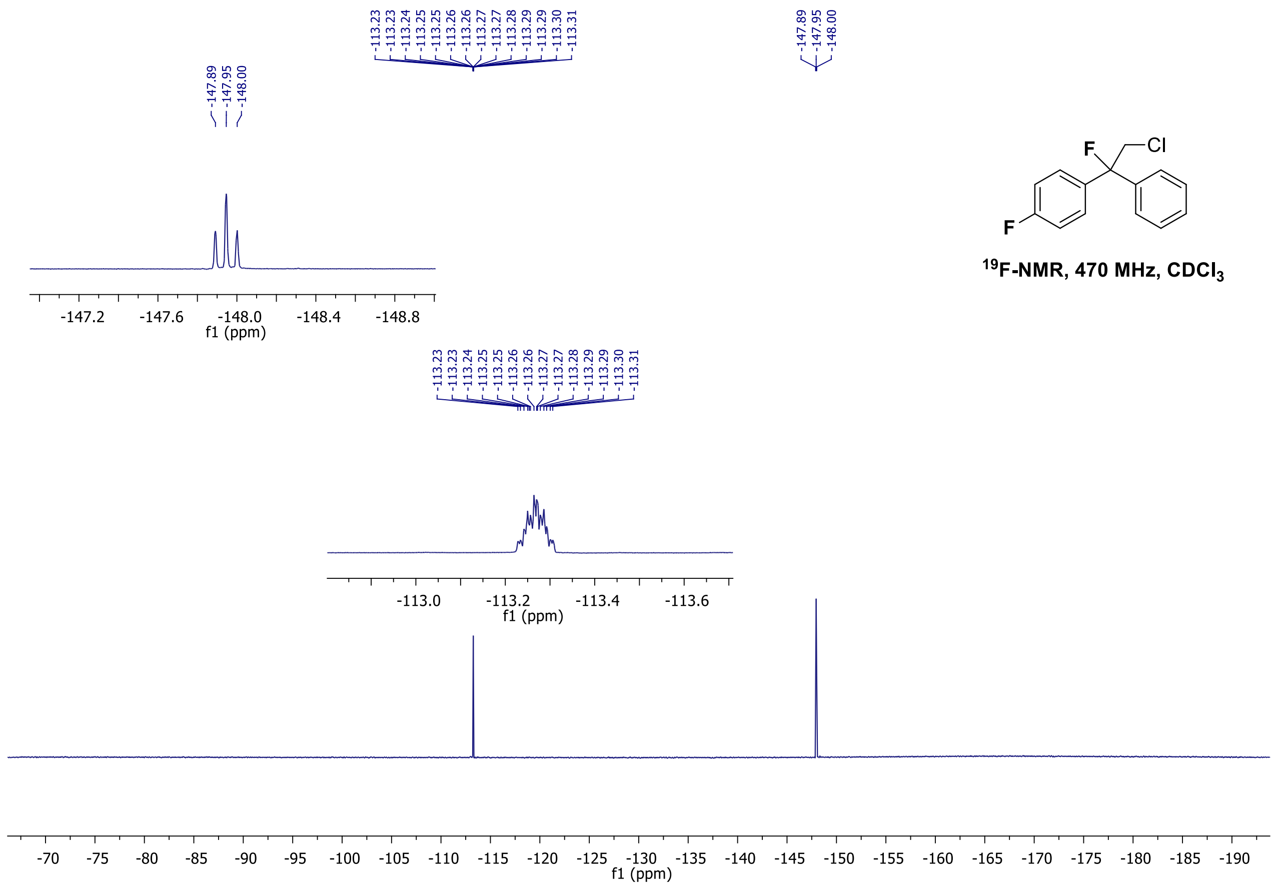
-113.23
-113.23
-113.24
-113.25
-113.25
-113.26
-113.26
-113.27
-113.27
-113.28
-113.29
-113.29
-113.30
-113.31
-147.89
-147.95
-148.00
F
Cl
F
^{19}F-NMR, 470 MHz, CDCl$_3$
-147.2
-147.6
-148.0
-148.4
-148.8
f1 (ppm)
-113.0
-113.2
-113.4
-113.6
f1 (ppm)
-70 -75 -80 -85 -90 -95 -100 -105 -110 -115 -120 -125 -130 -135 -140 -145 -150 -155 -160 -165 -170 -175 -180 -185 -190
f1 (ppm)

Compound 14

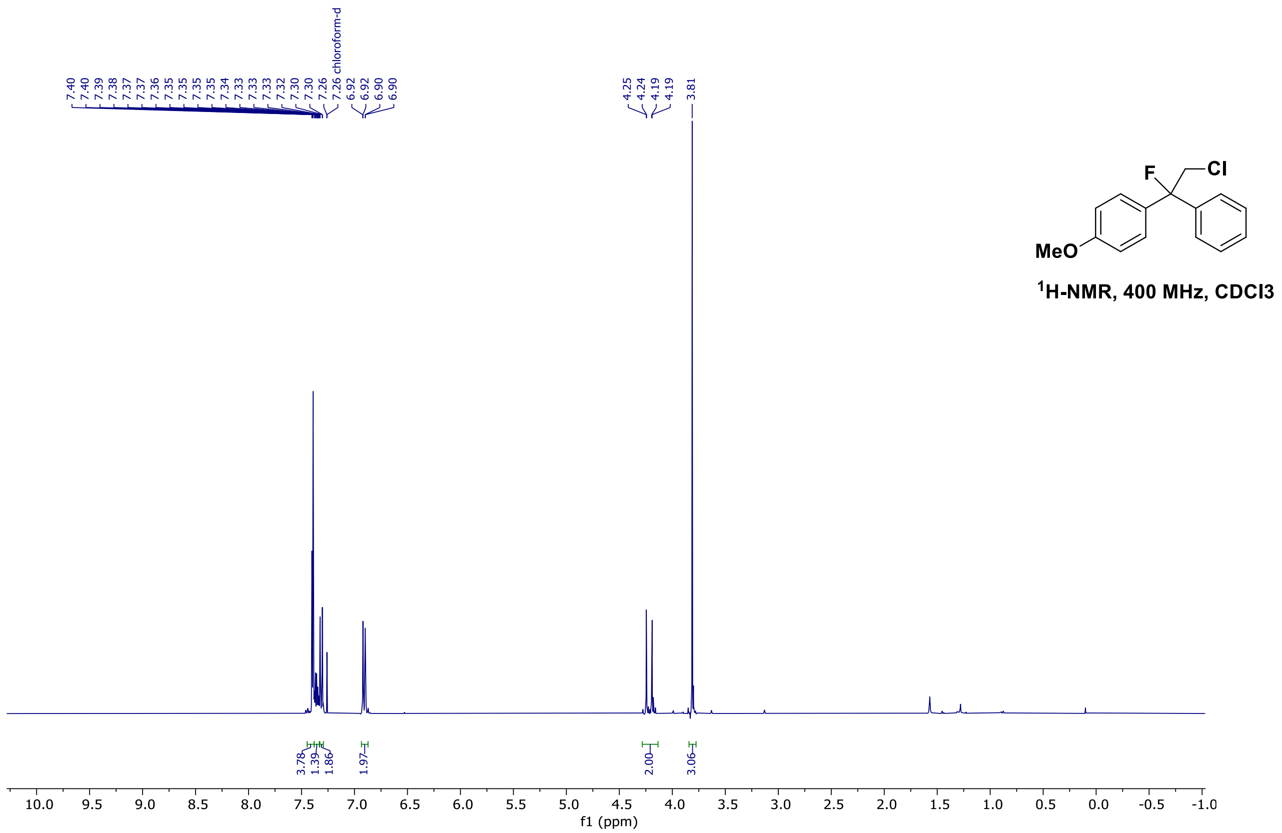

7.40
7.40
7.39
7.38
7.37
7.37
7.36
7.35
7.35
7.35
7.35
7.34
7.33
7.33
7.33
7.32
7.30
7.30
7.26 chloroform-d
6.92
6.92
6.90
6.90
4.25
4.24
4.19
4.19
3.81
F
Cl
MeO
^{1}H-NMR, 400 MHz, CDCl3
3.78
1.39
1.86
1.97
2.00
3.06
f1 (ppm)

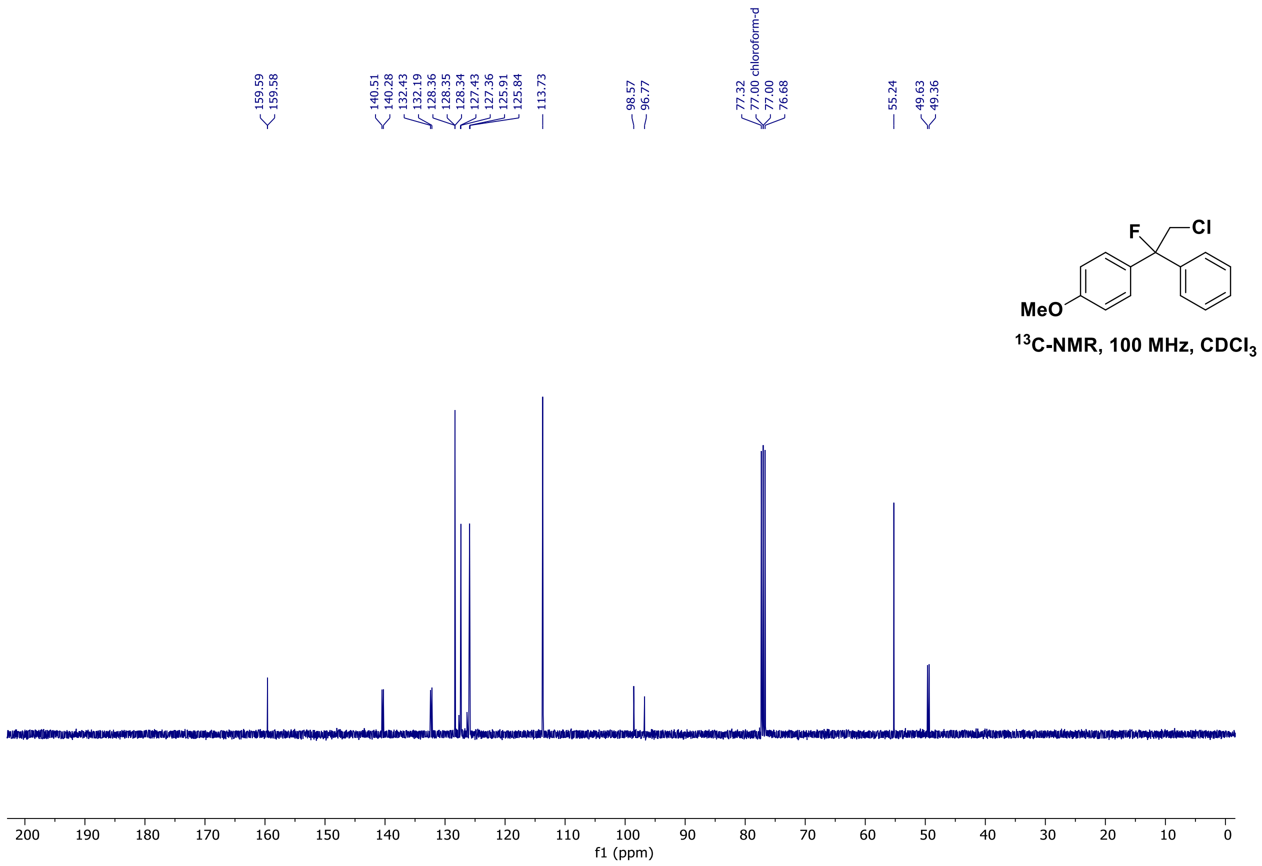
159.59
159.58
140.51
140.28
132.43
132.19
128.36
128.35
128.34
127.43
127.36
125.91
125.84
113.73
98.57
96.77
77.32
77.00 chloroform-d
77.00
76.68
55.24
49.63
49.36
F
Cl
MeO
^{13}C-NMR, 100 MHz, $CDCl_3$
200
190
180
170
160
150
140
130
120
110
100
90
80
70
60
50
40
30
20
10
0
f1 (ppm)

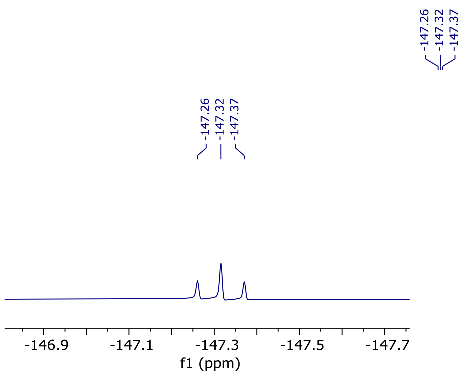

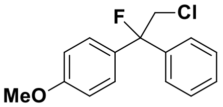

^{19}F-NMR, 470 MHz, $CDCl_3$

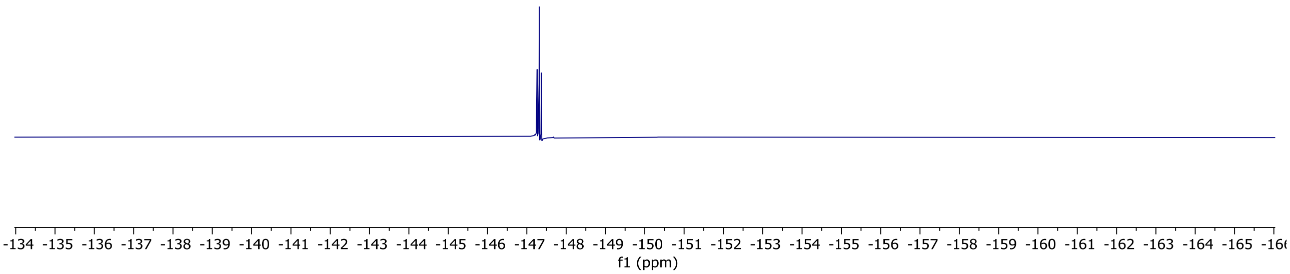

Compound 15

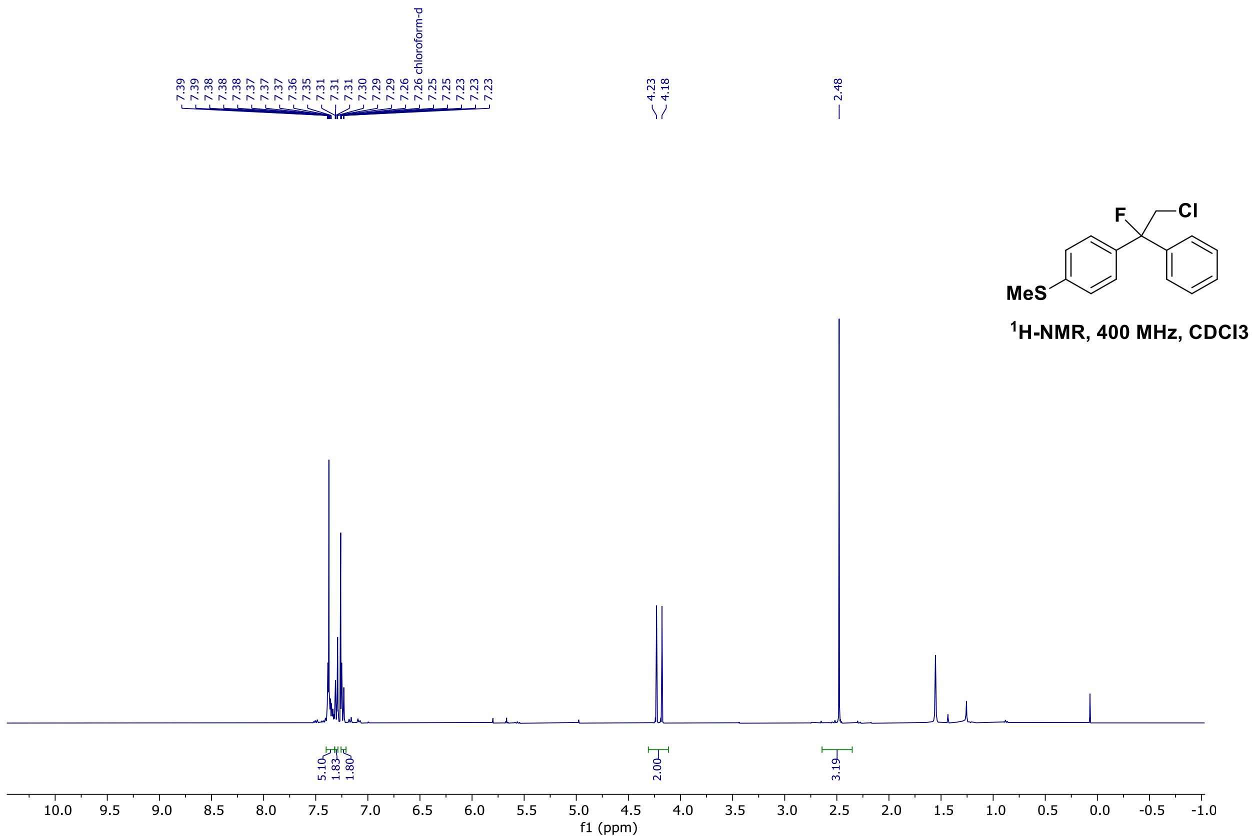
7.39
7.39
7.38
7.38
7.38
7.37
7.37
7.37
7.36
7.35
7.31
7.31
7.31
7.30
7.29
7.29
7.29
7.26 chloroform-d
7.25
7.25
7.23
7.23
7.23
4.23
4.18
2.48
F
Cl
MeS
^{1}H-NMR, 400 MHz, CDCl3
5.10
1.83
1.80
2.00
3.19
f1 (ppm)

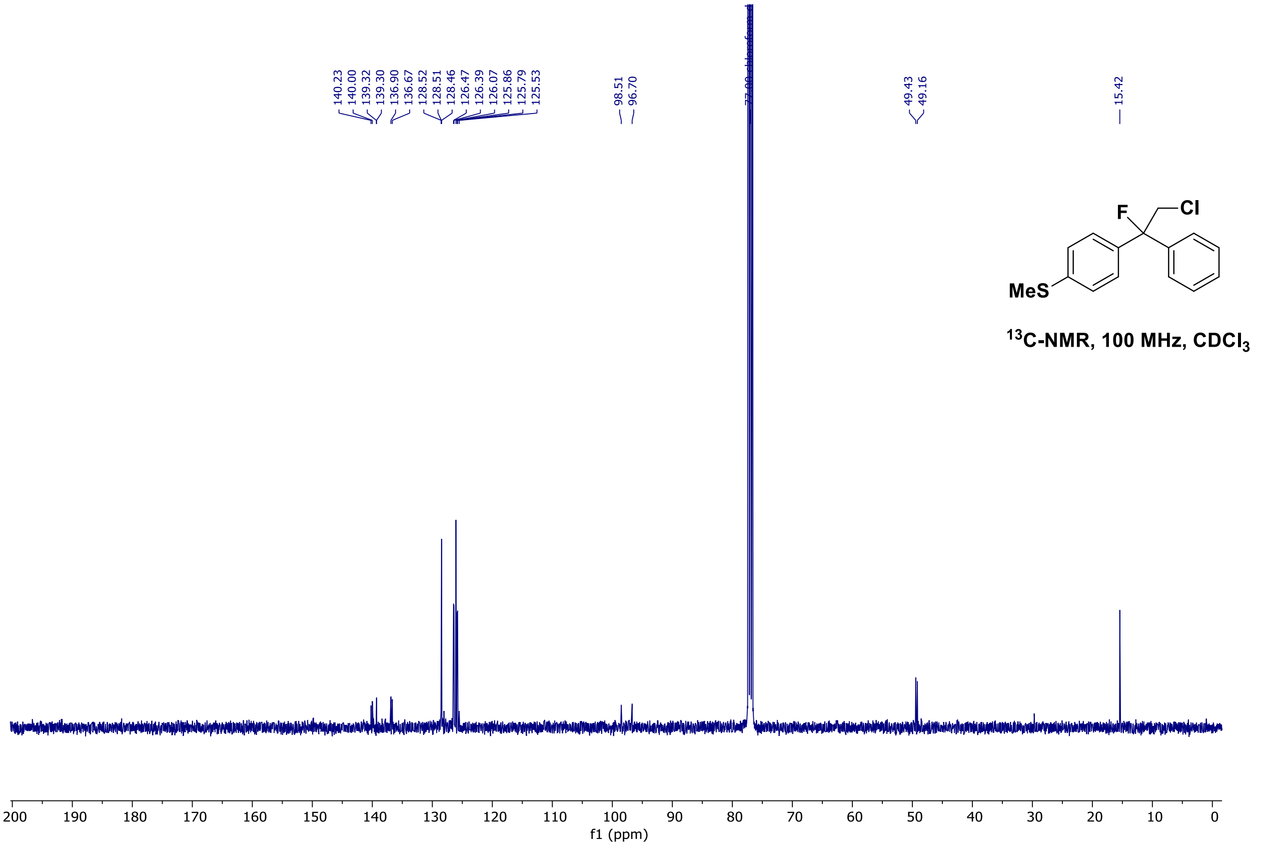
140.23
140.00
139.32
139.30
136.90
136.67
128.52
128.51
128.46
126.47
126.39
126.07
125.86
125.79
125.53
98.51
96.70
49.43
49.16
15.42
F
Cl
MeS
^{13}C-NMR, 100 MHz, $CDCl_3$
f1 (ppm)

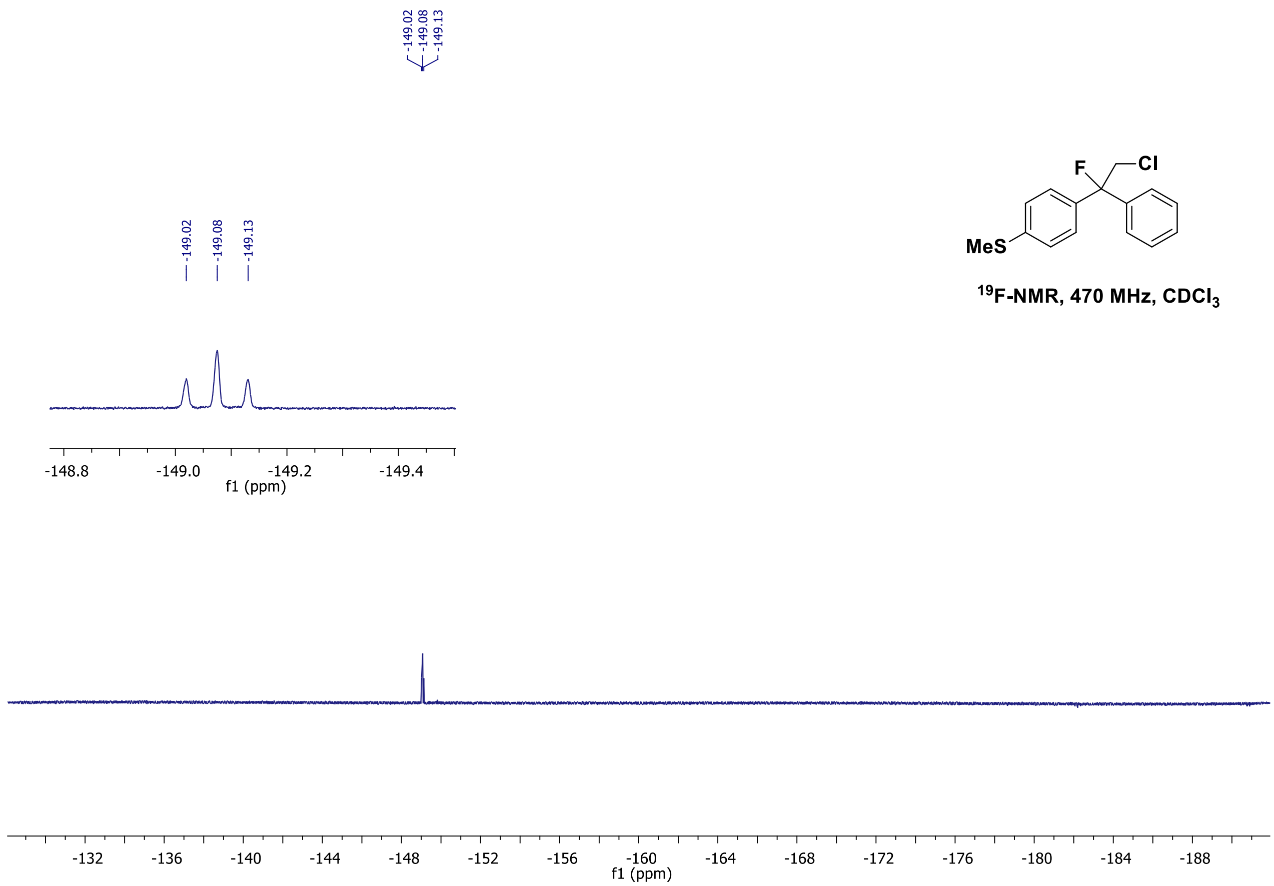
-149.02
-149.08
-149.13
F
Cl
MeS
^{19}F-NMR, 470 MHz, $CDCl_3$
-148.8
-149.0
-149.2
-149.4
f1 (ppm)
-132
-136
-140
-144
-148
-152
-156
-160
-164
-168
-172
-176
-180
-184
-188
f1 (ppm)

Compound 16

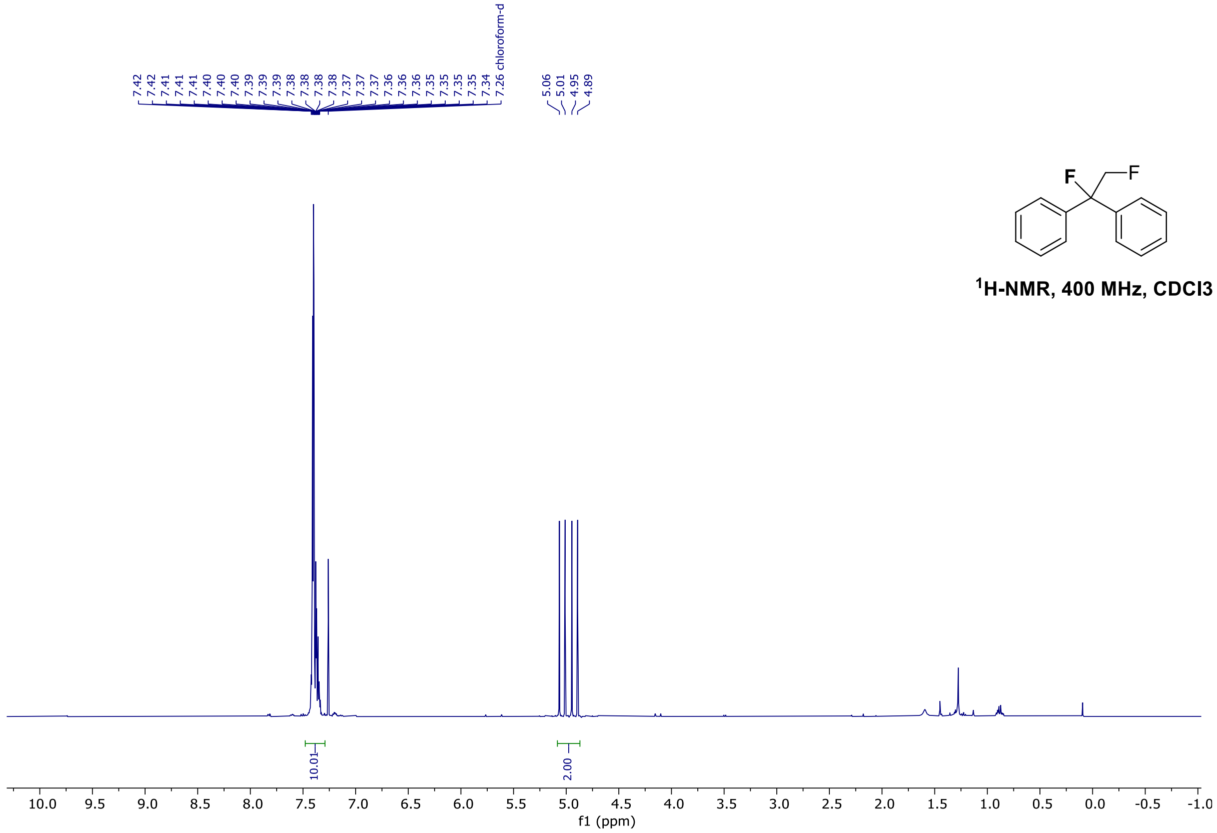

7.42
7.42
7.41
7.41
7.41
7.40
7.40
7.40
7.39
7.39
7.39
7.38
7.38
7.38
7.38
7.38
7.37
7.37
7.37
7.36
7.36
7.36
7.35
7.35
7.35
7.35
7.34
7.26 chloroform-d
5.06
5.01
4.95
4.89
F
F
¹H-NMR, 400 MHz, CDCl3
10.01
2.00
10.0 9.5 9.0 8.5 8.0 7.5 7.0 6.5 6.0 5.5 5.0 4.5 4.0 3.5 3.0 2.5 2.0 1.5 1.0 0.5 0.0 -0.5 -1.0
f1 (ppm)

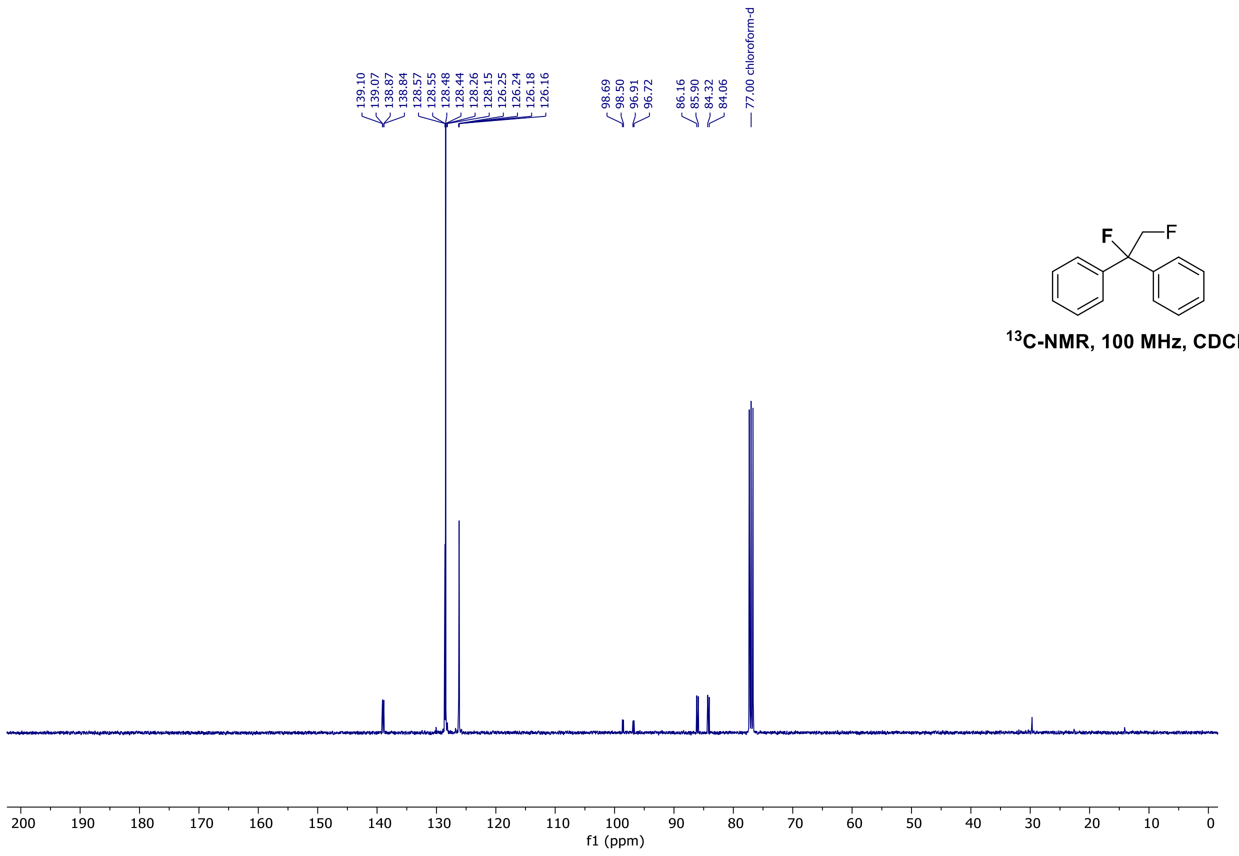

^{13}C-NMR, 100 MHz, $CDCl_3$

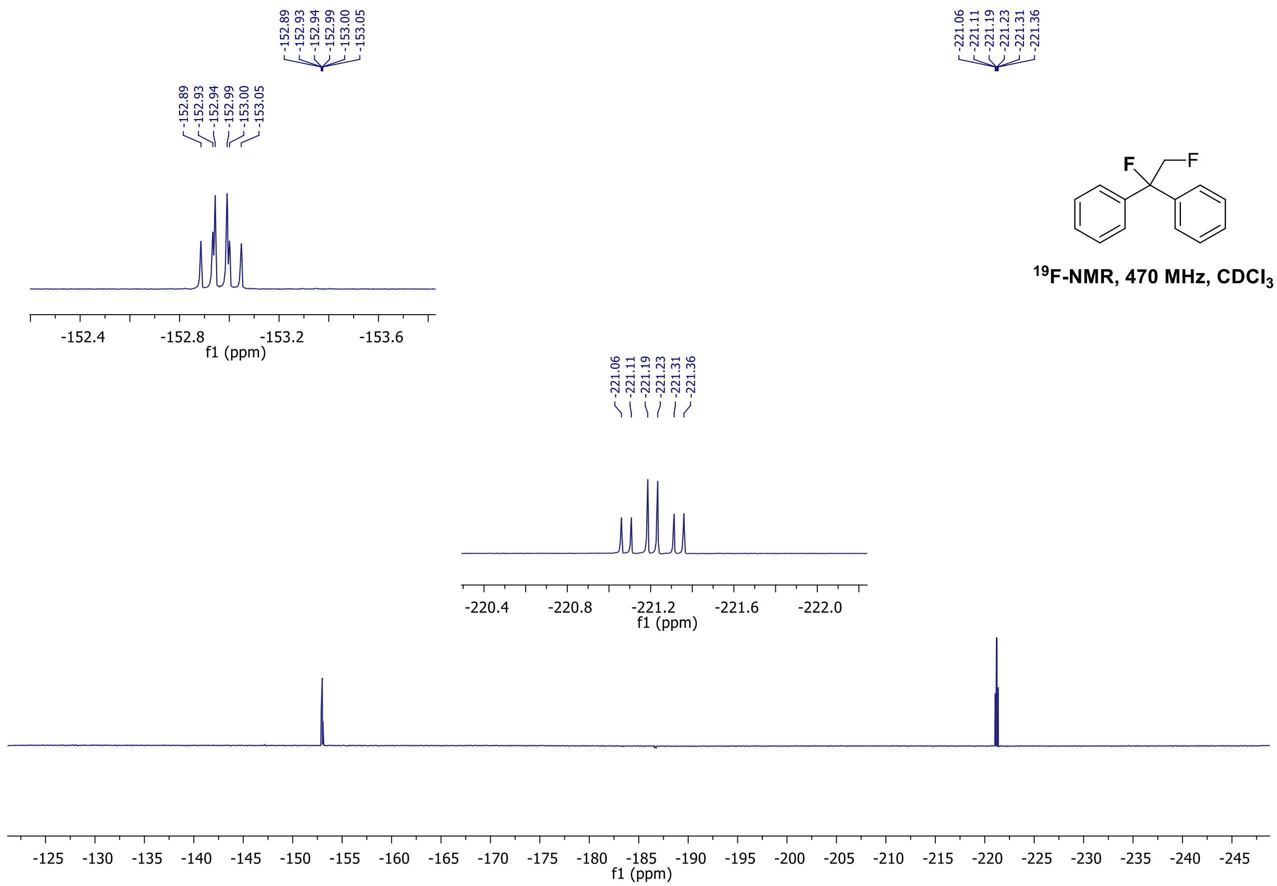
-152.89
-152.93
-152.94
-152.99
-153.00
-153.05
-221.06
-221.11
-221.19
-221.23
-221.31
-221.36
F
F
^{19}F-NMR, 470 MHz, CDCl$_3$
f1 (ppm)

Compound 17

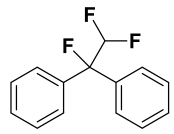

^{1}H-NMR, 400 MHz, $CDCl_3$

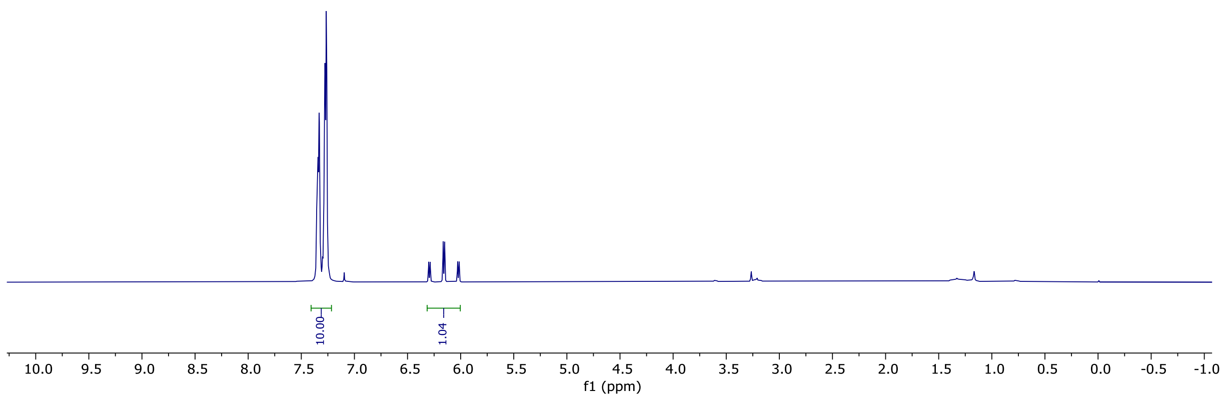

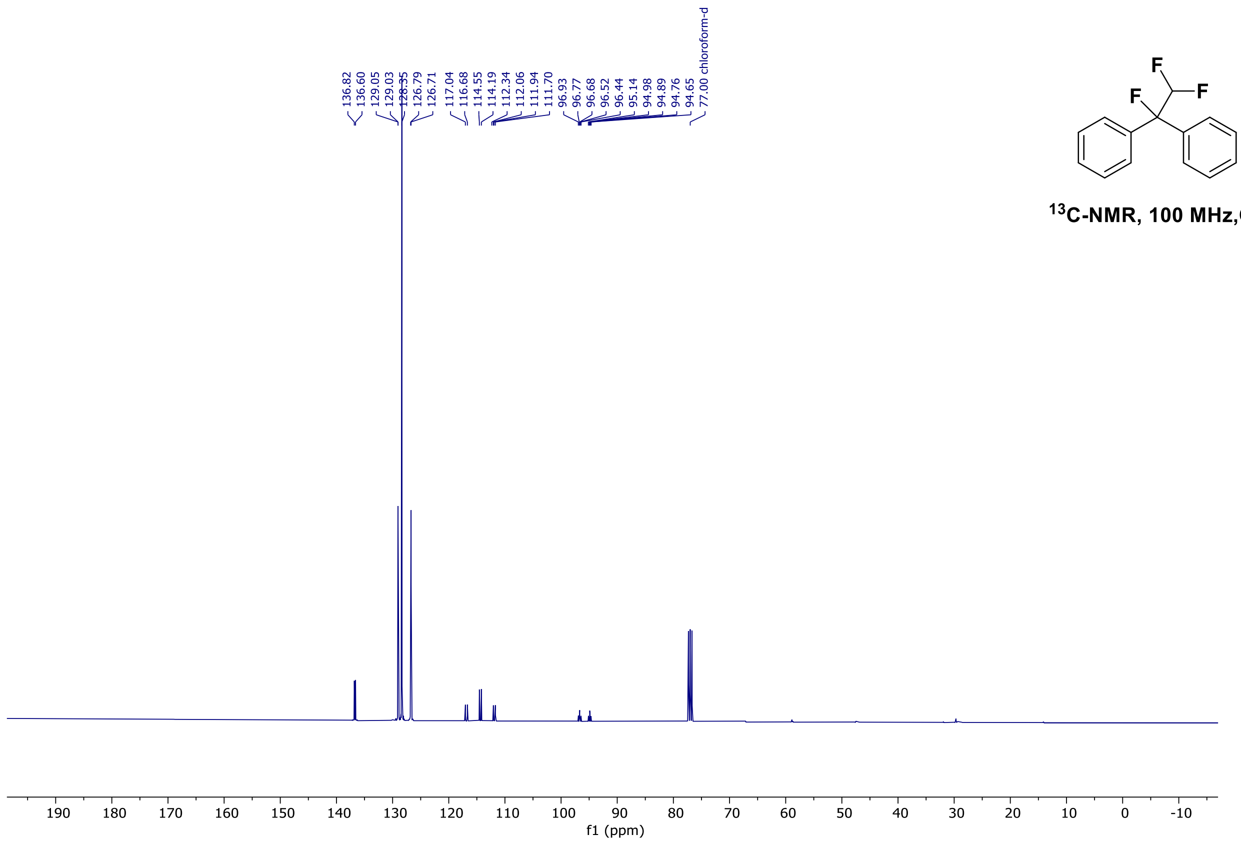

^{13}C-NMR, 100 MHz, $CDCl_3$

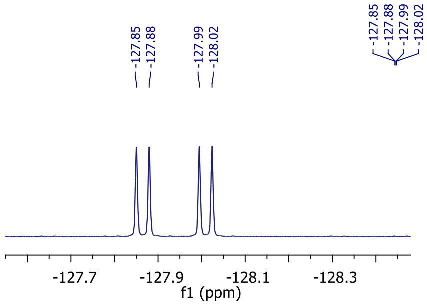

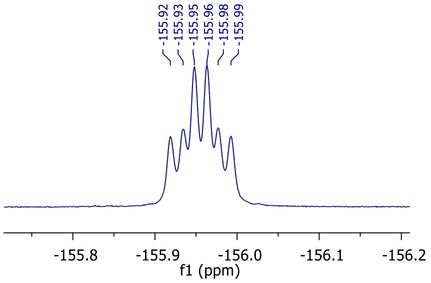

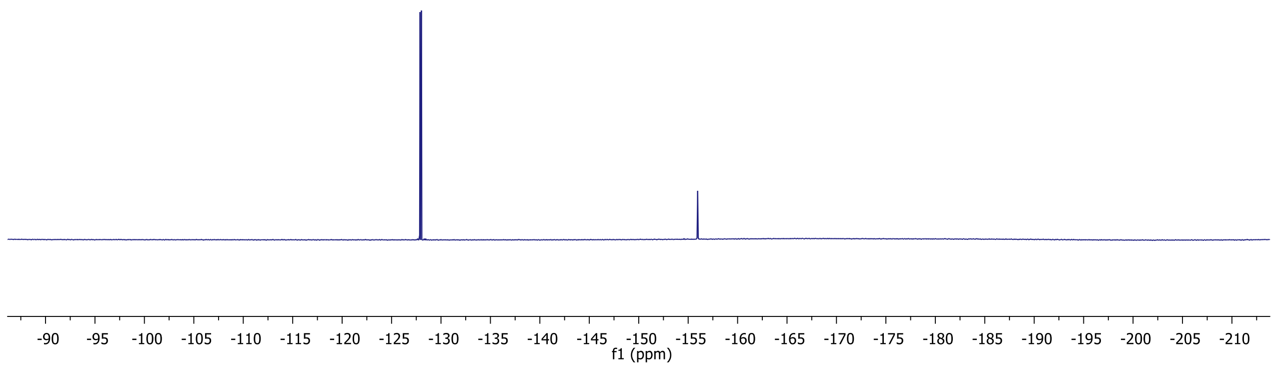

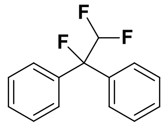

^{19}F-NMR, 470 MHz, C_6D_6

Compound 18

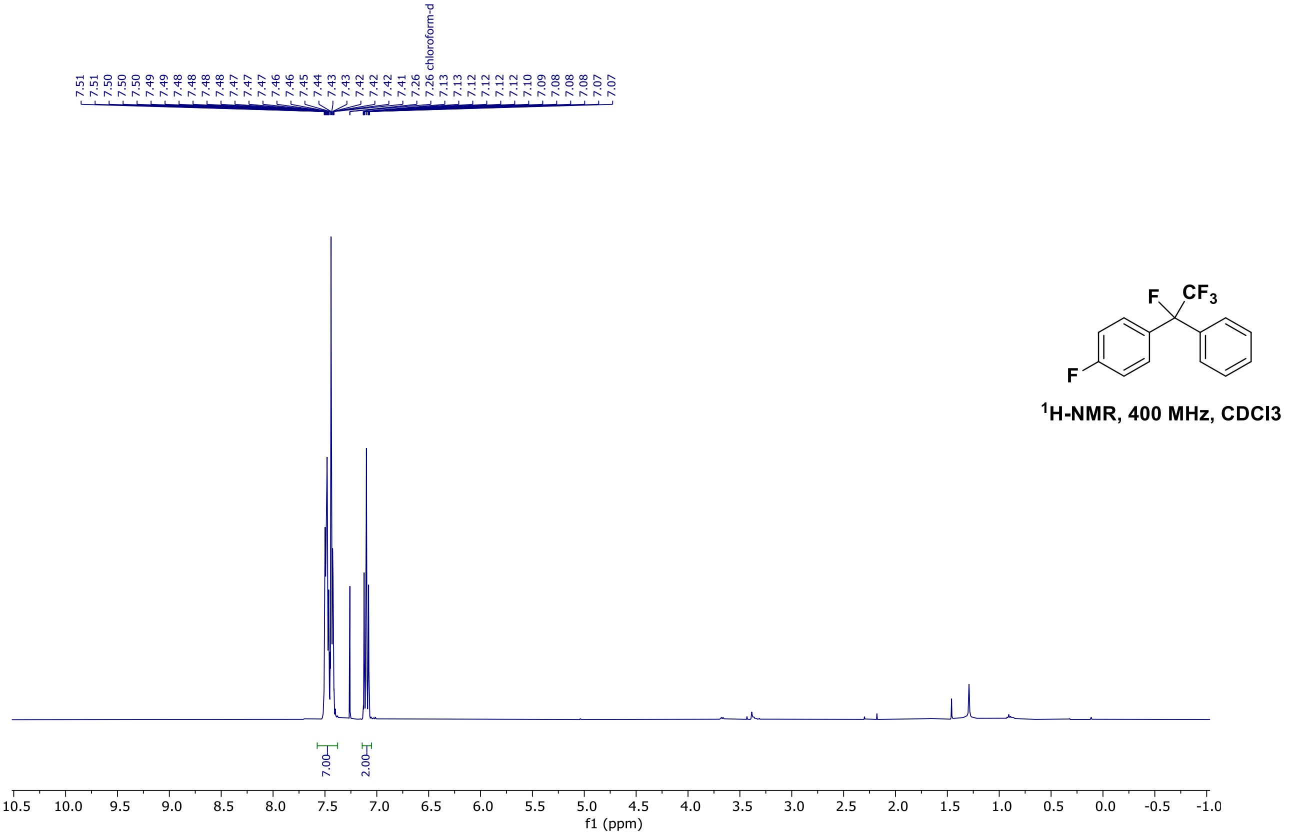
7.51 7.51 7.50 7.50 7.50 7.49 7.49 7.48 7.48 7.48 7.48 7.47 7.47 7.47 7.46 7.45 7.44 7.43 7.43 7.42 7.42 7.42 7.41 7.26 chloroform-d 7.13 7.13 7.12 7.12 7.12 7.12 7.10 7.09 7.08 7.08 7.08 7.07 7.07
F
CF3
F
1H-NMR, 400 MHz, CDCl3
7.00
2.00
f1 (ppm)

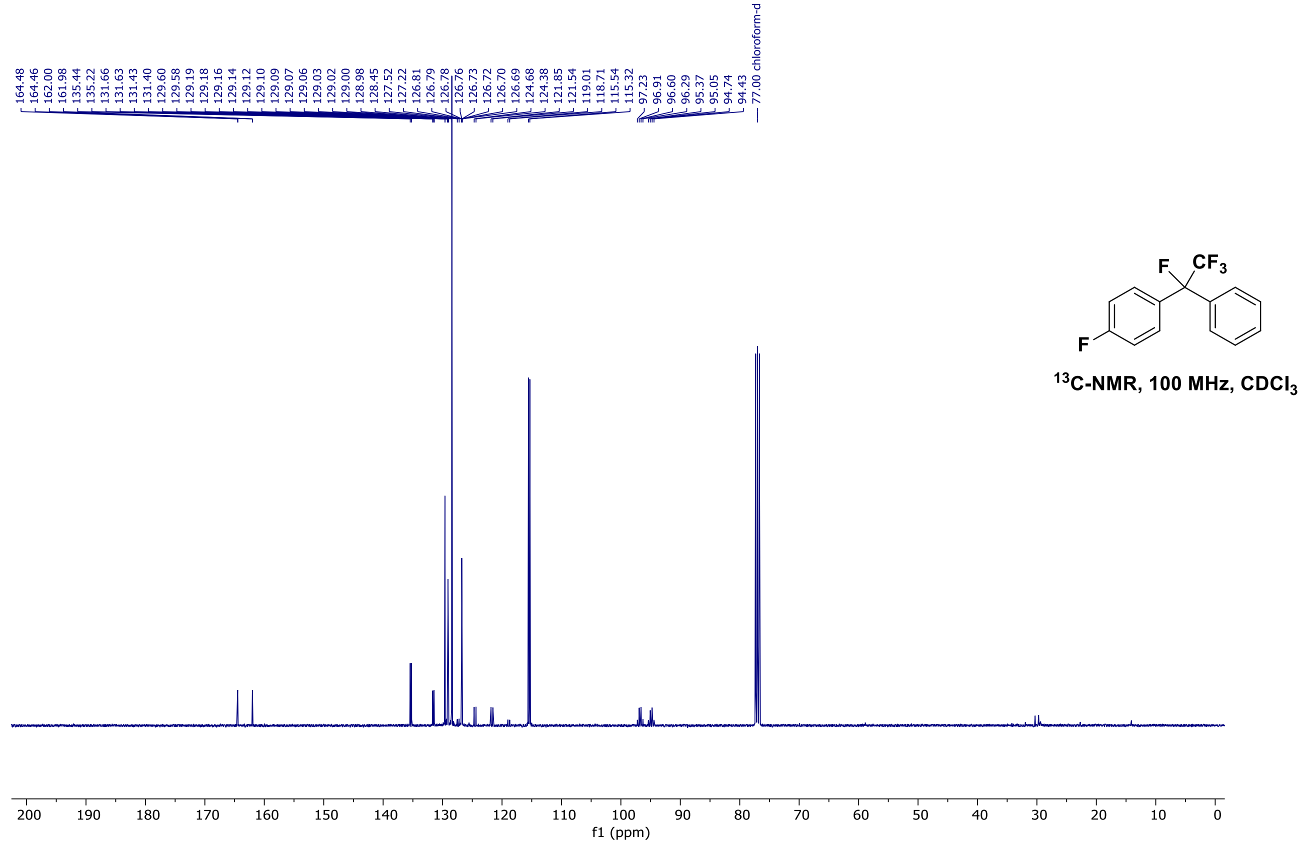
164.48 164.46 162.00 161.98 135.44 135.22 131.66 131.63 131.43 131.40 129.60 129.58 129.19 129.18 129.16 129.14 129.12 129.10 129.09 129.07 129.06 129.03 129.02 129.00 128.98 128.45 127.52 127.22 126.81 126.79 126.78 126.76 126.73 126.72 126.70 126.69 124.68 124.38 121.85 121.54 119.01 118.71 115.54 115.32 97.23 96.91 96.60 96.29 95.37 95.05 94.74 94.43 77.00 chloroform-d
F
CF3
F
13C-NMR, 100 MHz, CDCl3
f1 (ppm)

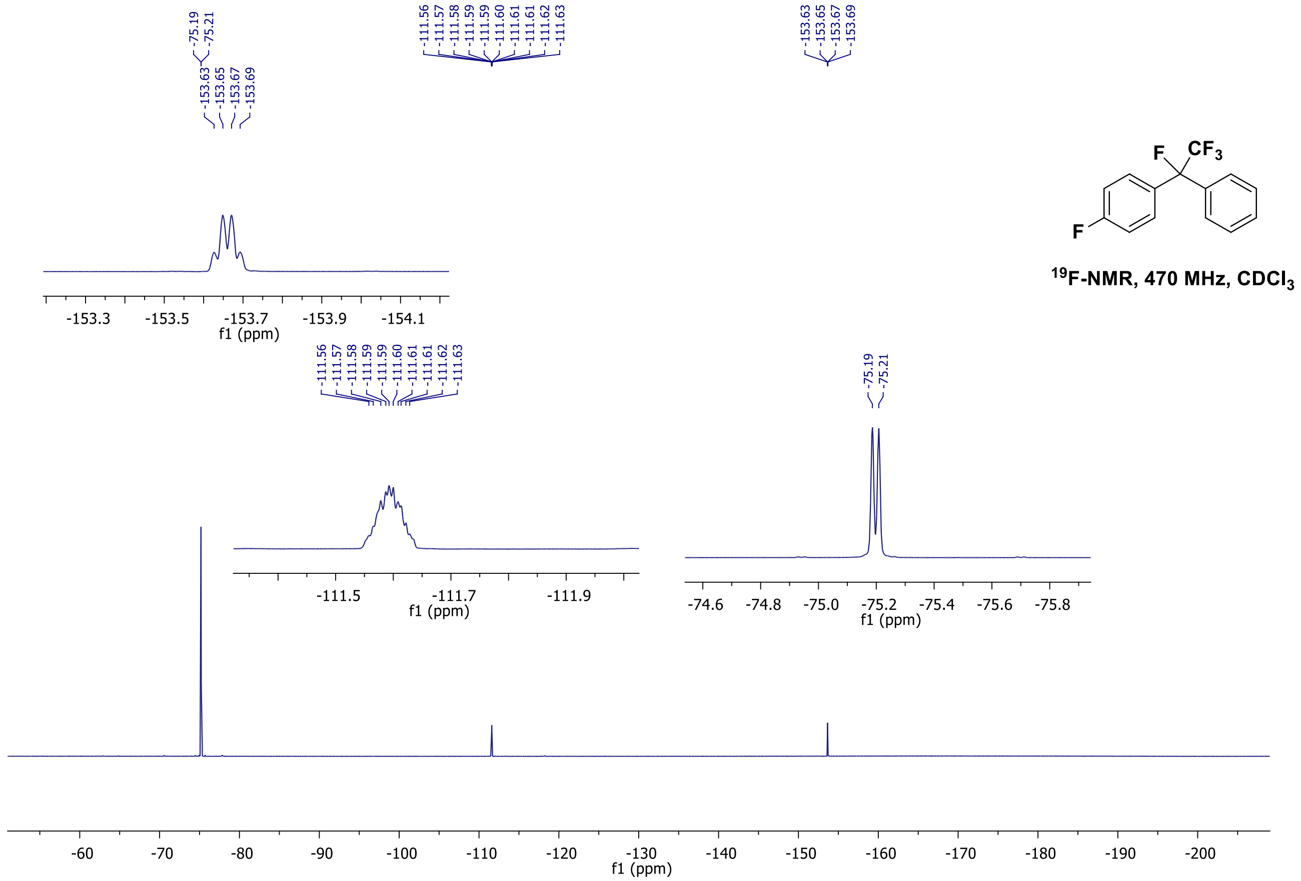

-75.19
-75.21
-153.63
-153.65
-153.67
-153.69
-111.56
-111.57
-111.58
-111.59
-111.59
-111.60
-111.61
-111.61
-111.62
-111.63
F
CF3
F
19F-NMR, 470 MHz, CDCl3
-153.3 -153.5 -153.7 -153.9 -154.1
-111.5 -111.7 -111.9
-74.6 -74.8 -75.0 -75.2 -75.4 -75.6 -75.8
-60 -70 -80 -90 -100 -110 -120 -130 -140 -150 -160 -170 -180 -190 -200
f1 (ppm)

Compound 19

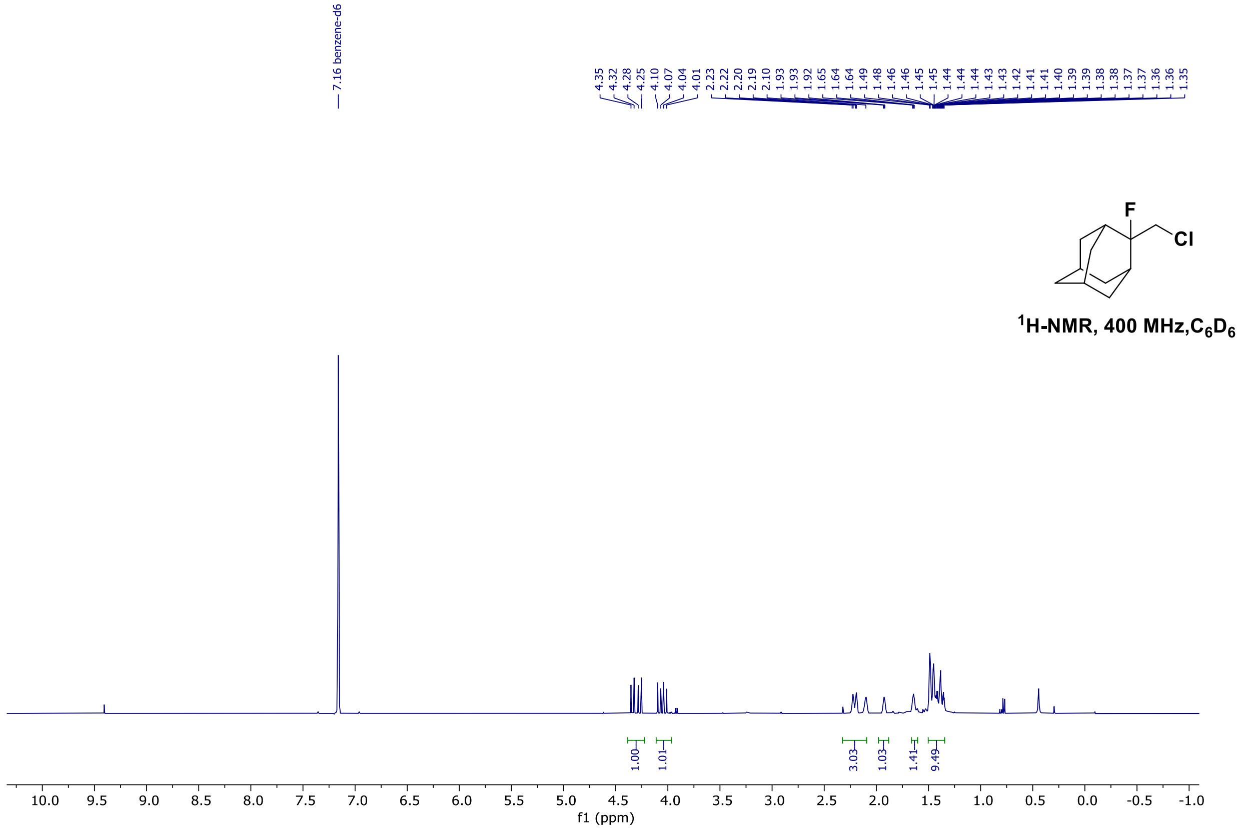
7.16 benzene-d6
4.35
4.32
4.28
4.25
4.10
4.07
4.04
4.01
2.23
2.22
2.20
2.19
2.10
1.93
1.93
1.92
1.65
1.64
1.64
1.49
1.48
1.46
1.46
1.45
1.45
1.44
1.44
1.44
1.43
1.43
1.42
1.41
1.41
1.40
1.39
1.39
1.38
1.38
1.37
1.37
1.36
1.36
1.35
F
Cl
^{1}H-NMR, 400 MHz,C_6D_6
1.00
1.01
3.03
1.03
1.41
9.49
f1 (ppm)

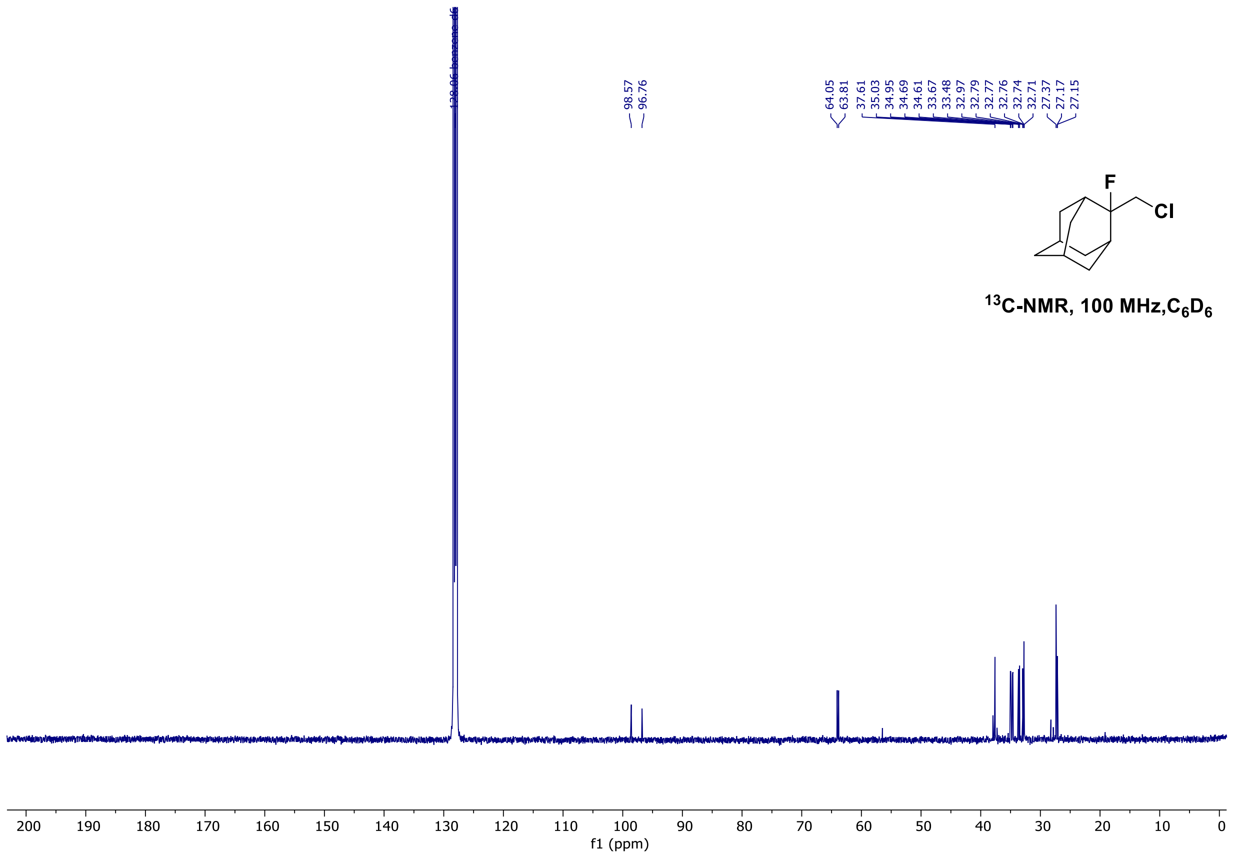
128.06 benzene-d6
98.57
96.76
64.05
63.81
37.61
35.03
34.95
34.69
34.61
33.67
33.48
32.97
32.79
32.77
32.76
32.74
32.71
27.37
27.17
27.15
F
Cl
13C-NMR, 100 MHz,C6D6
200 190 180 170 160 150 140 130 120 110 100 90 80 70 60 50 40 30 20 10 0
f1 (ppm)

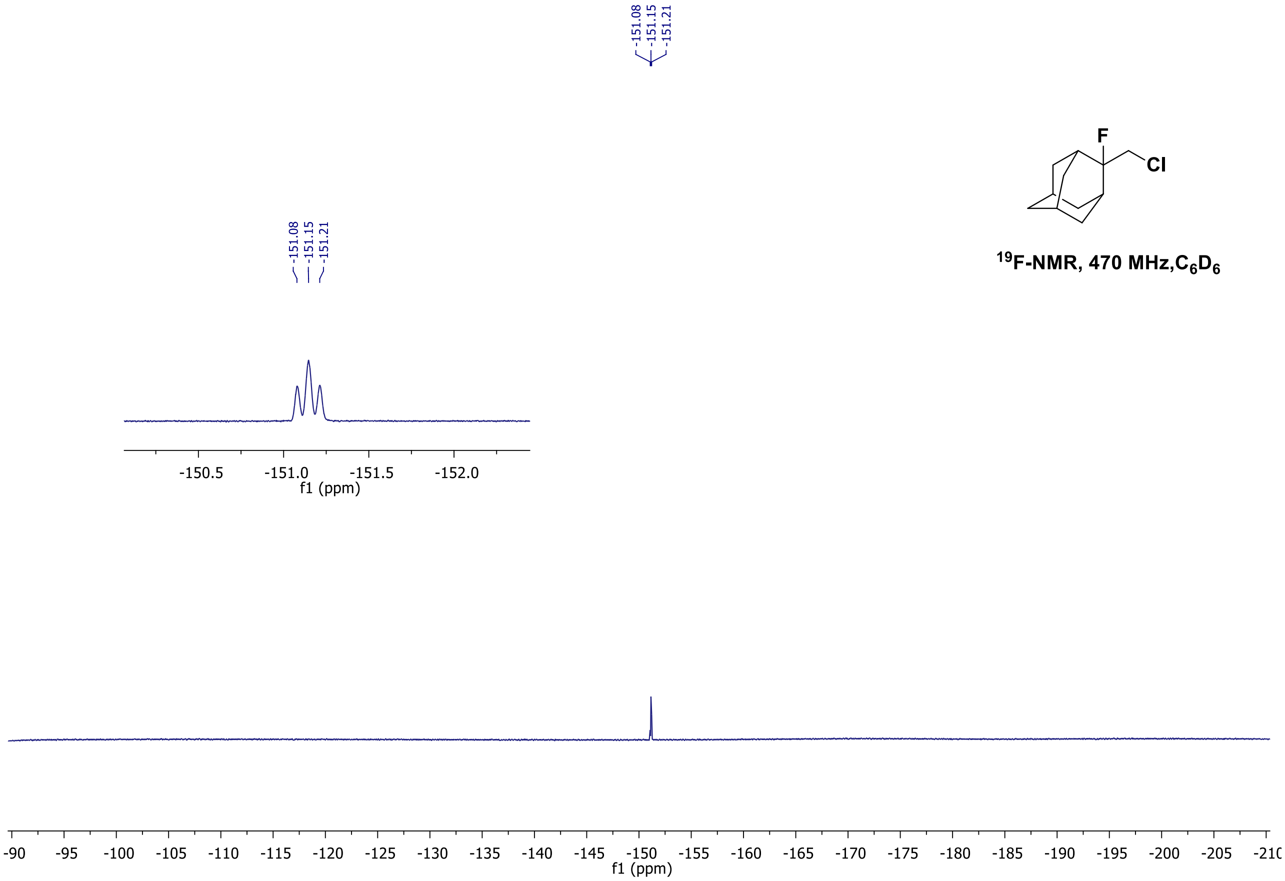
-151.08
-151.15
-151.21
F
Cl
19F-NMR, 470 MHz,C6D6
-150.5
-151.0
-151.5
-152.0
f1 (ppm)
-90
-95
-100
-105
-110
-115
-120
-125
-130
-135
-140
-145
-150
-155
-160
-165
-170
-175
-180
-185
-190
-195
-200
-205
-210
f1 (ppm)

Compound 20

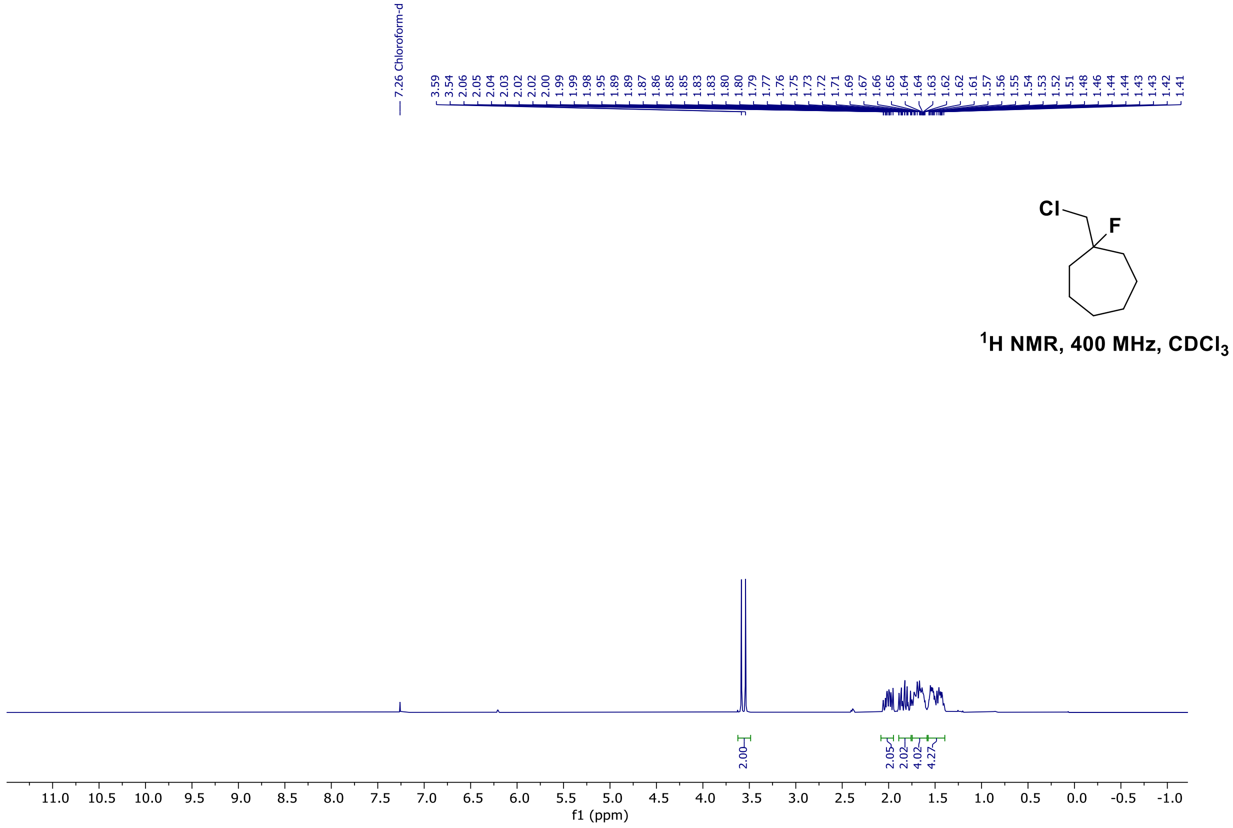
7.26 Chloroform-d
3.59
3.54
2.06
2.05
2.04
2.03
2.02
2.02
2.00
1.99
1.99
1.98
1.95
1.89
1.89
1.87
1.86
1.85
1.85
1.83
1.83
1.80
1.80
1.79
1.77
1.76
1.75
1.73
1.72
1.71
1.69
1.67
1.66
1.65
1.64
1.64
1.63
1.62
1.62
1.61
1.57
1.56
1.55
1.54
1.53
1.52
1.51
1.48
1.46
1.44
1.44
1.43
1.43
1.42
1.41
Cl
F
^{1}H NMR, 400 MHz, CDCl$_3$
2.00
2.05
2.02
4.02
4.27
11.0 10.5 10.0 9.5 9.0 8.5 8.0 7.5 7.0 6.5 6.0 5.5 5.0 4.5 4.0 3.5 3.0 2.5 2.0 1.5 1.0 0.5 0.0 -0.5 -1.0
f1 (ppm)

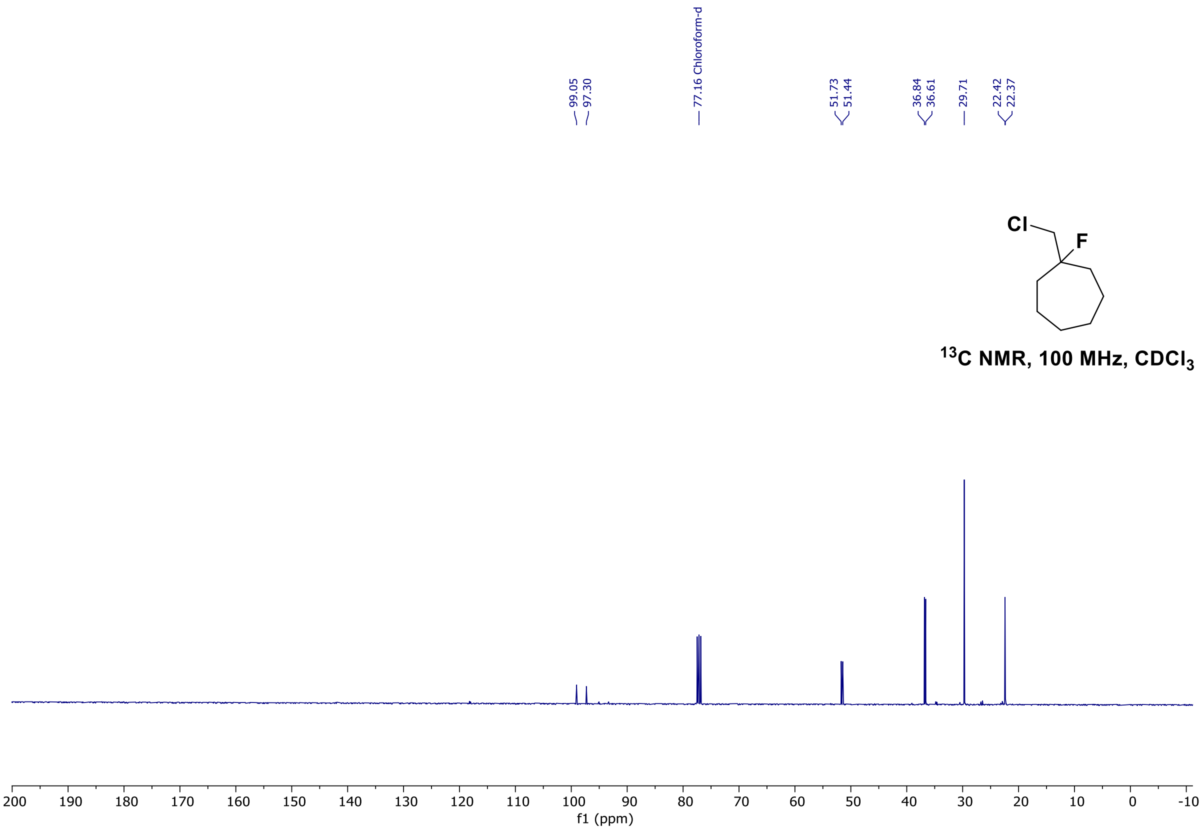
99.05
97.30
77.16 Chloroform-d
51.73
51.44
36.84
36.61
29.71
22.42
22.37
Cl
F
13C NMR, 100 MHz, CDCl3
f1 (ppm)

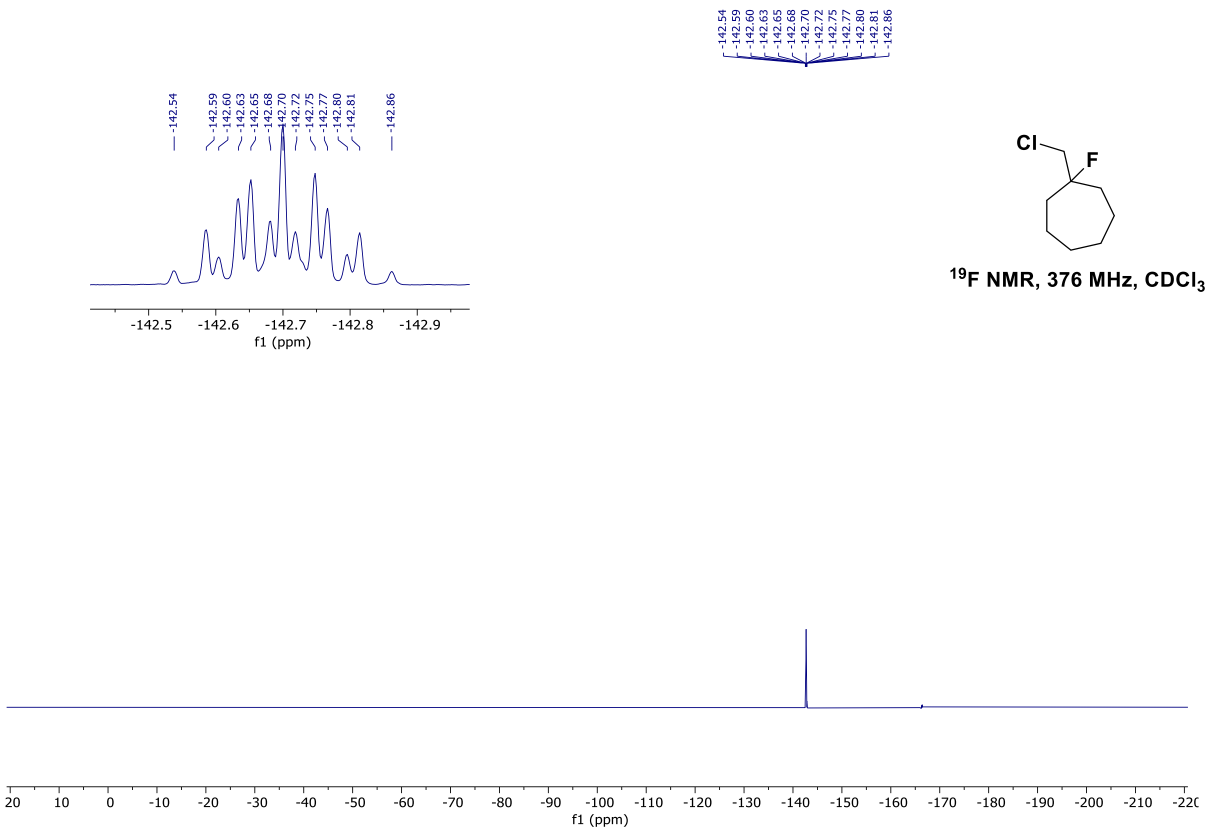
-142.54
-142.59
-142.60
-142.63
-142.65
-142.68
-142.70
-142.72
-142.75
-142.77
-142.80
-142.81
-142.86
Cl
F
19F NMR, 376 MHz, CDCl3
f1 (ppm)

Compound 21

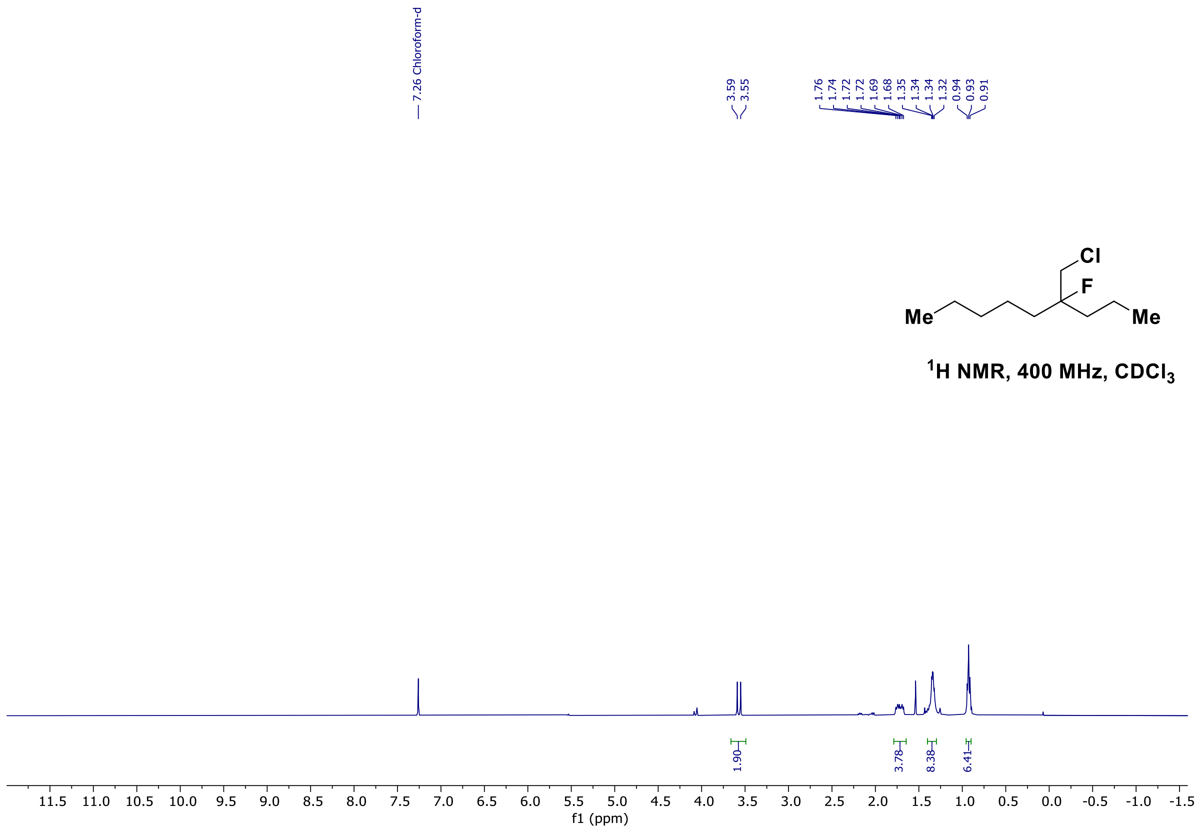
7.26 Chloroform-d
3.59
3.55
1.76
1.74
1.72
1.72
1.69
1.68
1.35
1.34
1.34
1.32
0.94
0.93
0.91
Cl
F
Me
Me
^{1}H NMR, 400 MHz, CDCl$_3$
1.90
3.78
8.38
6.41
f1 (ppm)

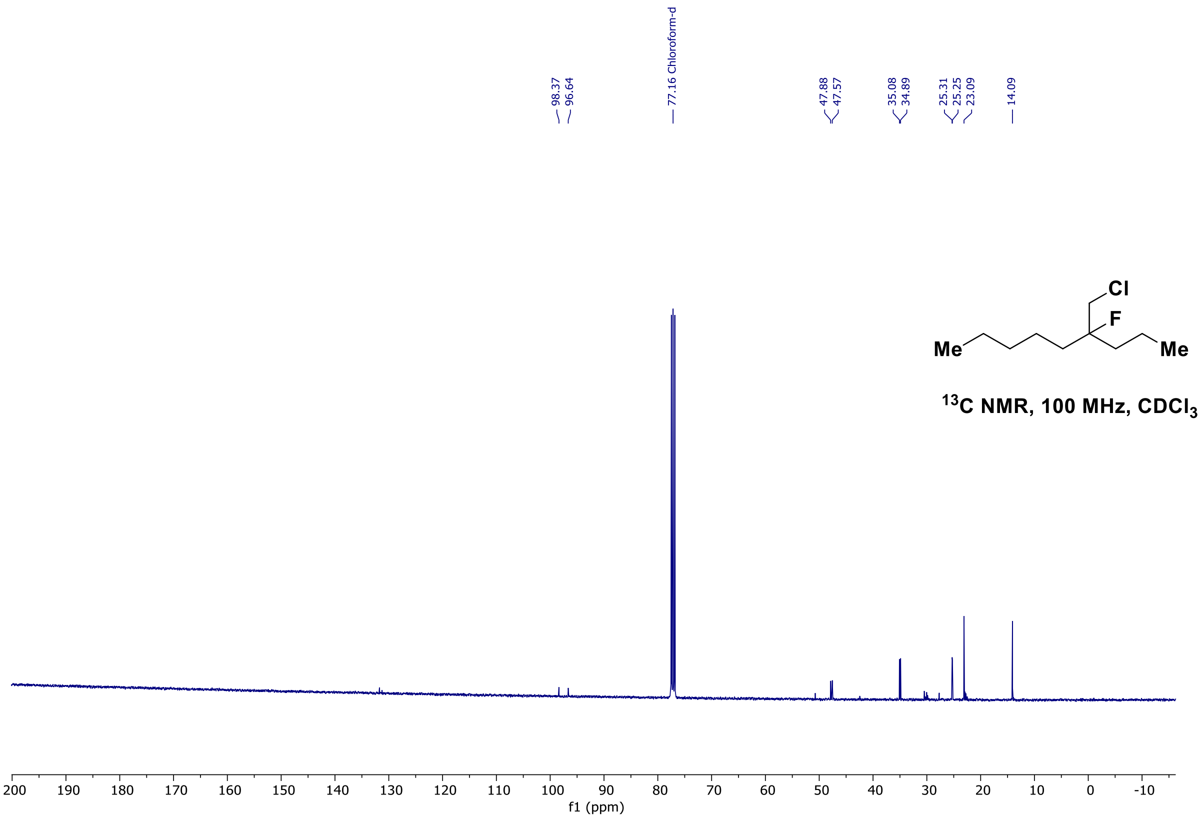
98.37
96.64
77.16 Chloroform-d
47.88
47.57
35.08
34.89
25.31
25.25
23.09
14.09
Cl
F
Me
Me
^{13}C NMR, 100 MHz, CDCl$_3$
f1 (ppm)

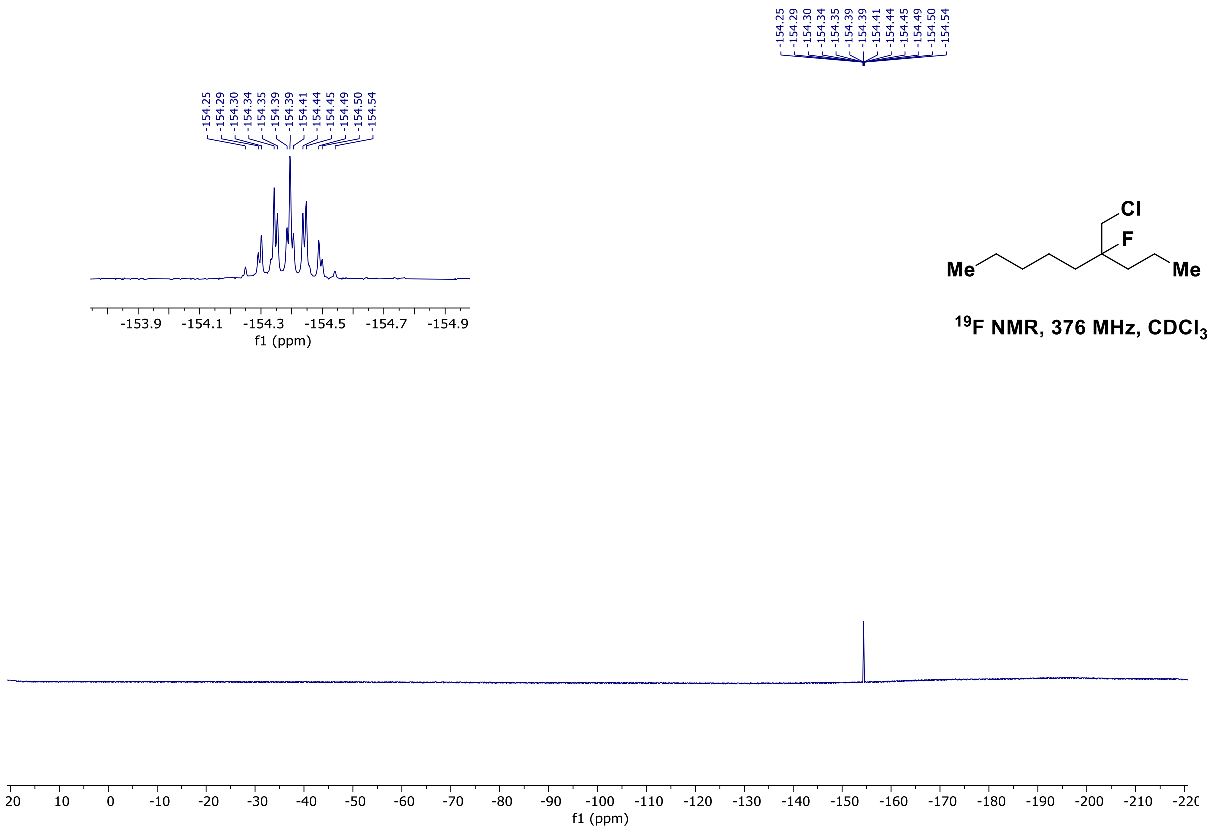

^{19}F NMR, 376 MHz, $CDCl_3$

Compound 22

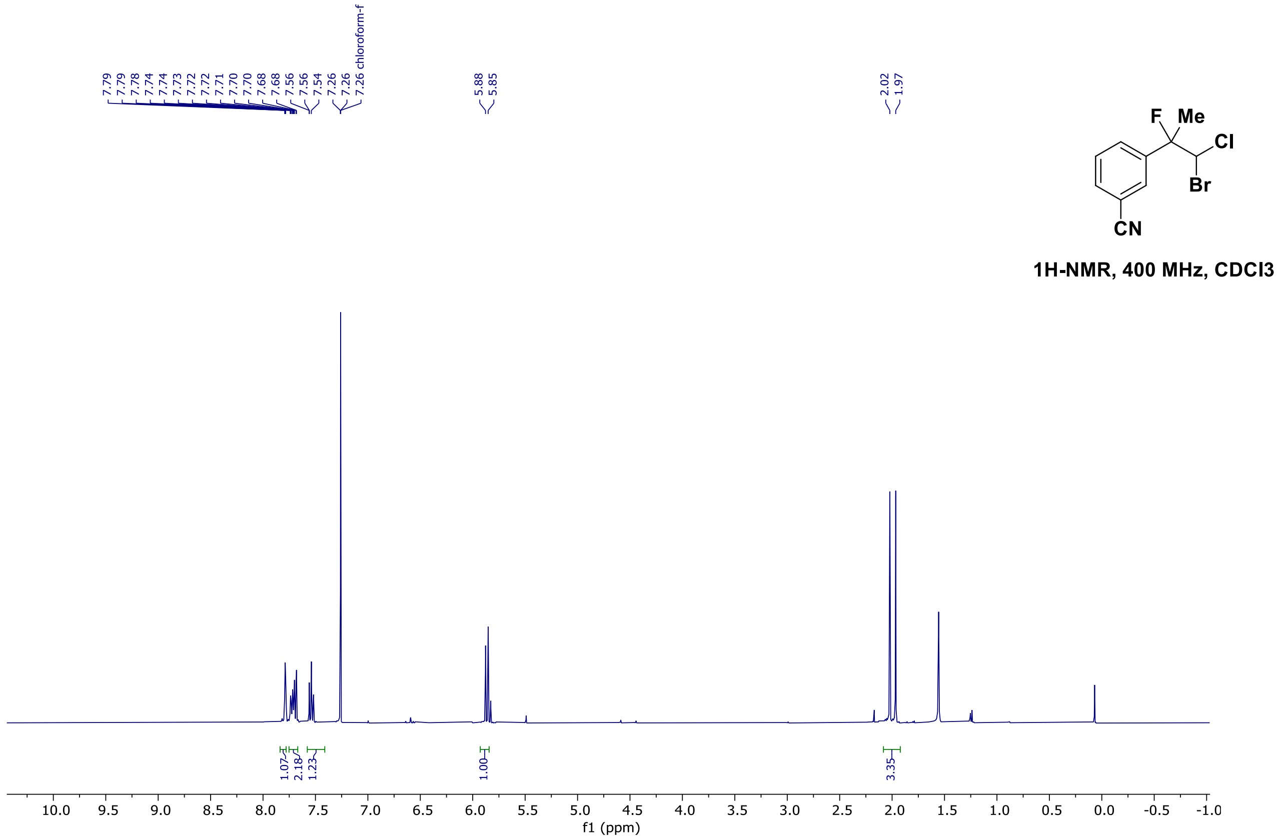
7.79 7.79 7.78 7.74 7.74 7.73 7.72 7.72 7.71 7.70 7.70 7.68 7.68 7.56 7.56 7.54 7.26 7.26 7.26 chloroform-f
5.88 5.85
2.02 1.97
F
Me
Cl
Br
CN
1H-NMR, 400 MHz, CDCl3
1.07 2.18 1.23
1.00
3.35
f1 (ppm)

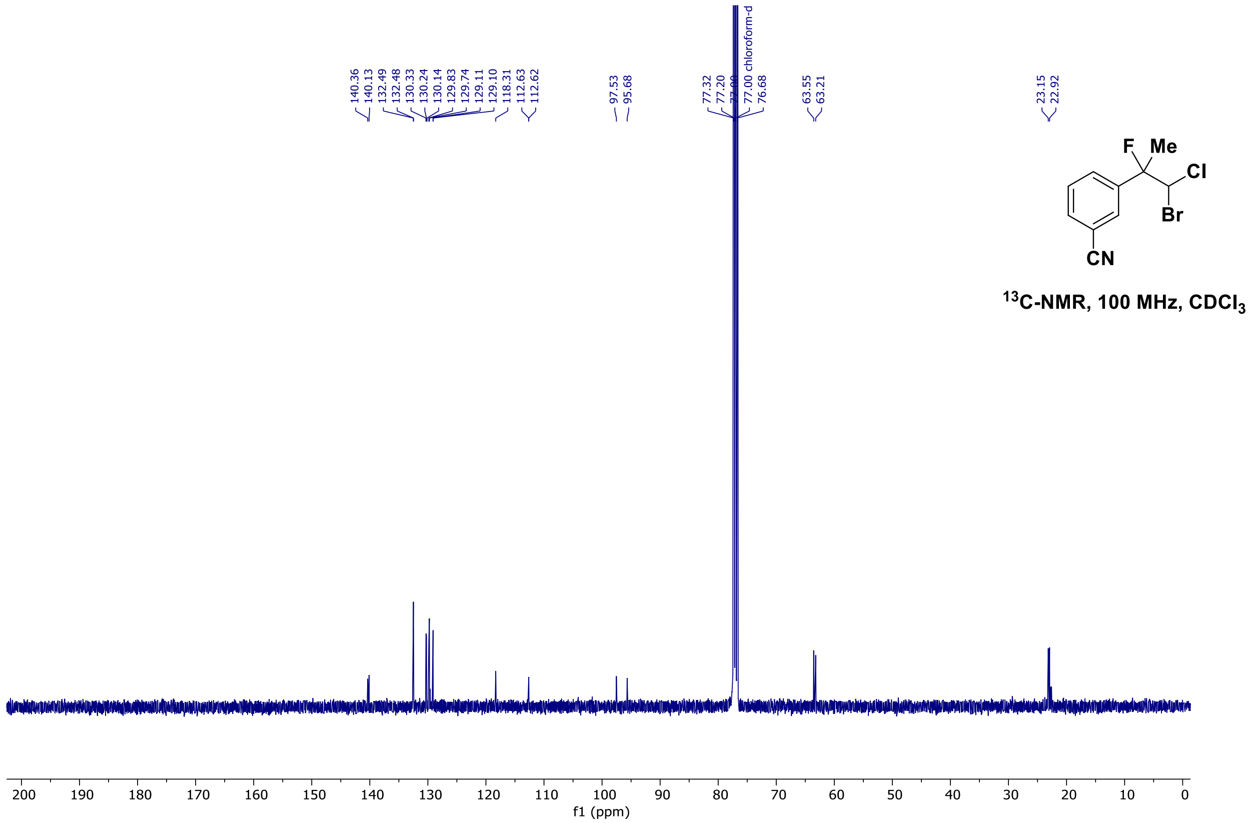
140.36 140.13 132.49 132.48 130.33 130.24 130.14 129.83 129.74 129.11 129.10 118.31 112.63 112.62
97.53 95.68
77.32 77.20 77.00 chloroform-d 76.68
63.55 63.21
23.15 22.92
F
Me
Cl
Br
CN
¹³C-NMR, 100 MHz, CDCl₃
f1 (ppm)

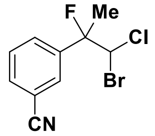

^{19}F-NMR, 470 MHz, $CDCl_3$

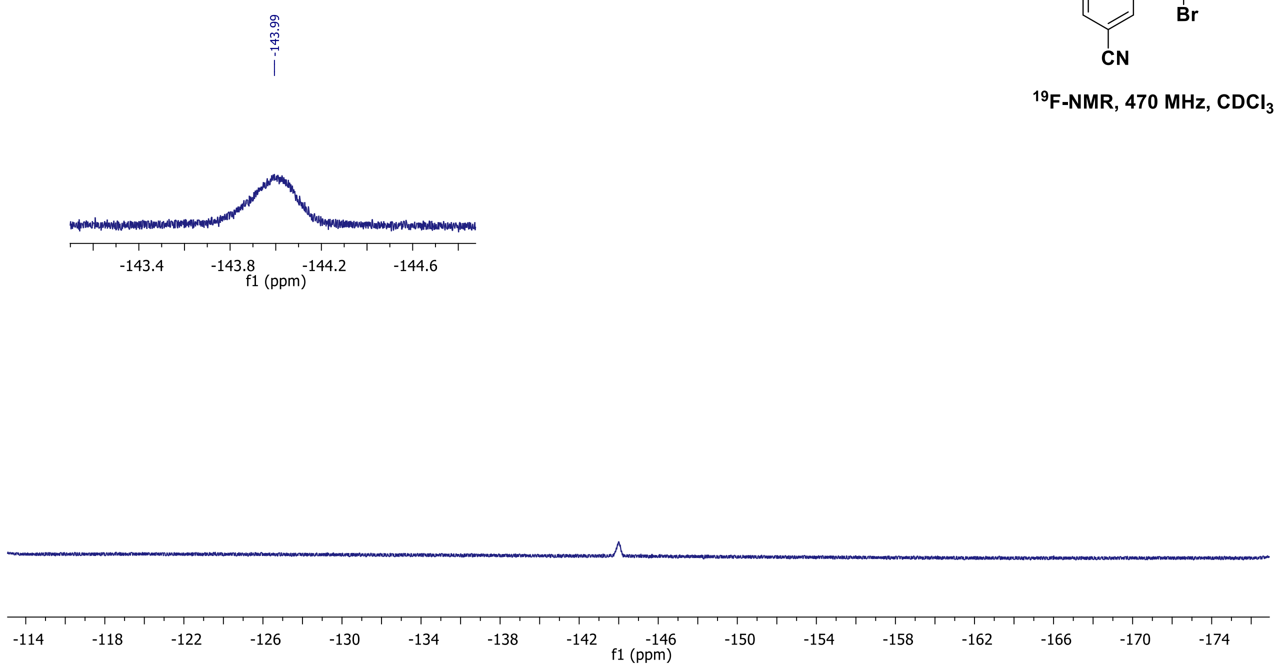

Compound 23

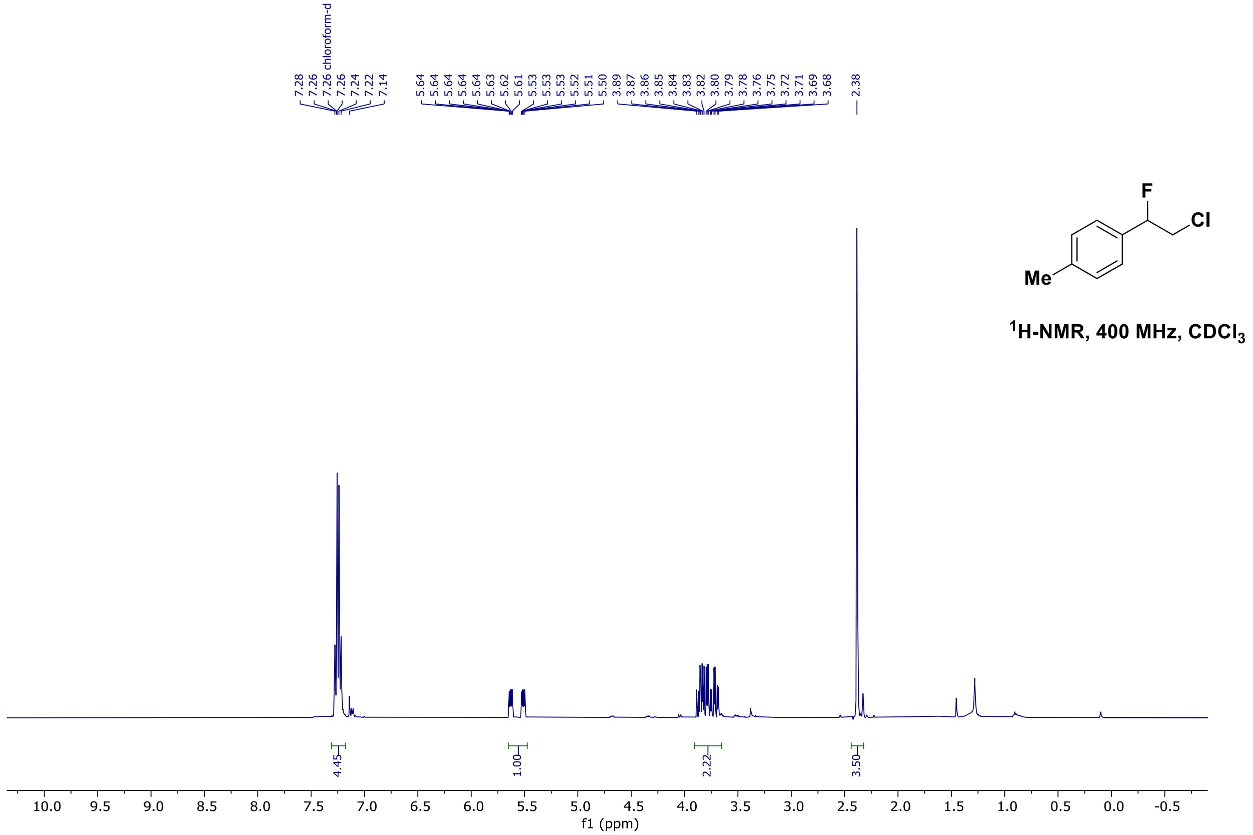

7.28
7.26
7.26 chloroform-d
7.26
7.24
7.22
7.14
5.64
5.64
5.64
5.64
5.64
5.63
5.62
5.61
5.53
5.53
5.53
5.52
5.51
5.50
3.89
3.87
3.86
3.85
3.84
3.83
3.82
3.80
3.79
3.78
3.76
3.75
3.72
3.71
3.69
3.68
2.38
F
Cl
Me
^{1}H-NMR, 400 MHz, CDCl$_3$
4.45
1.00
2.22
3.50
10.0
9.5
9.0
8.5
8.0
7.5
7.0
6.5
6.0
5.5
5.0
4.5
4.0
3.5
3.0
2.5
2.0
1.5
1.0
0.5
0.0
-0.5
f1 (ppm)

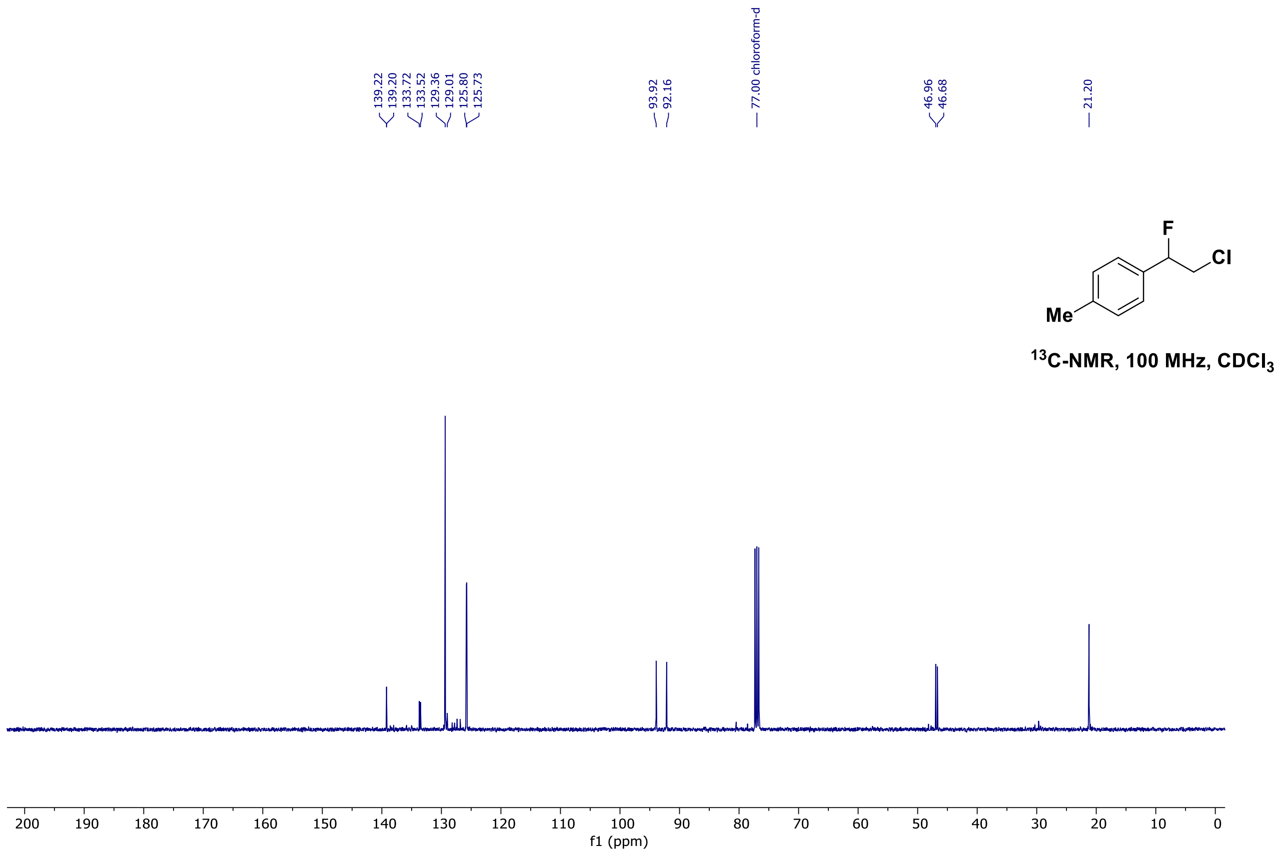
139.22
139.20
133.72
133.52
129.36
129.01
125.80
125.73
93.92
92.16
77.00 chloroform-d
46.96
46.68
21.20
F
Cl
Me
^{13}C-NMR, 100 MHz, CDCl$_3$
f1 (ppm)

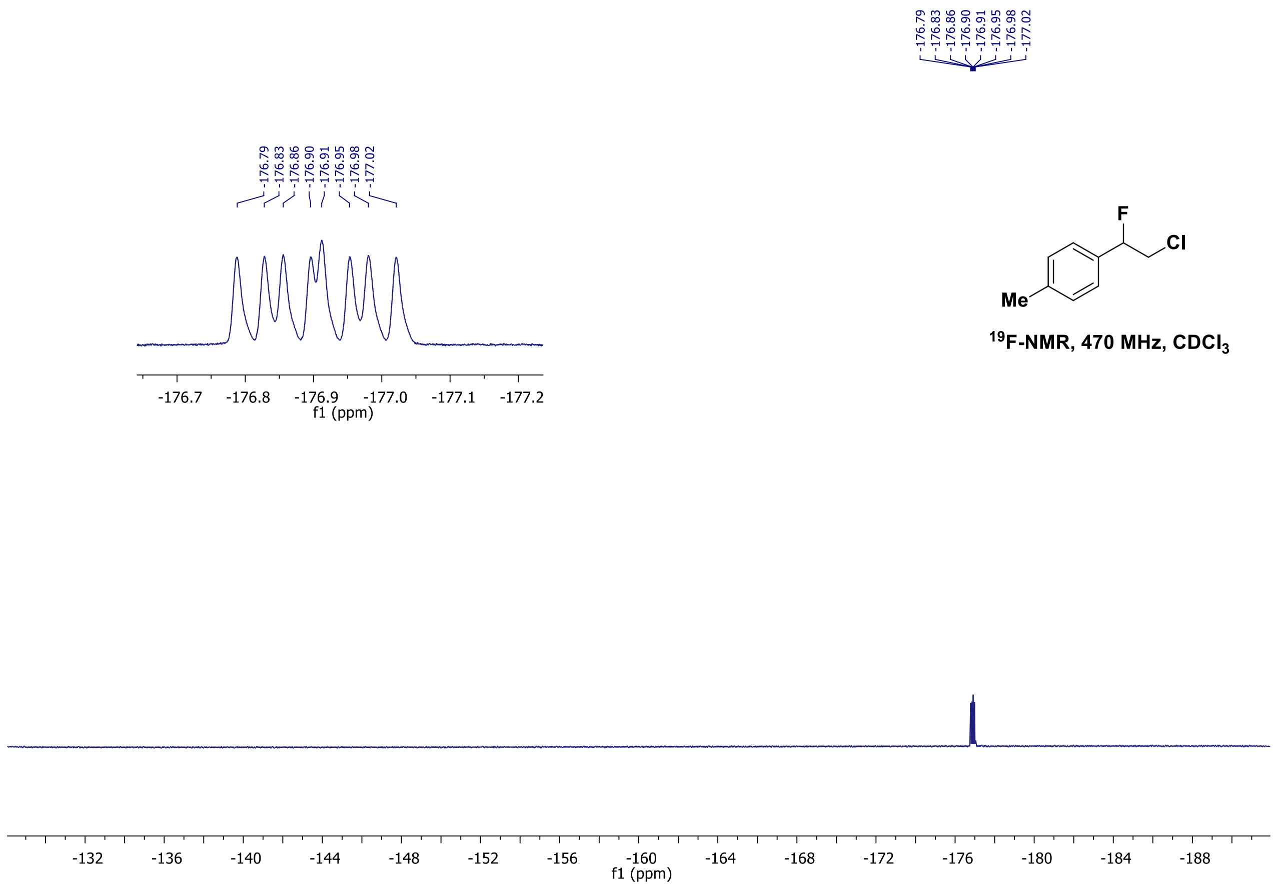
-176.79
-176.83
-176.86
-176.90
-176.91
-176.95
-176.98
-177.02
-176.7
-176.8
-176.9
-177.0
-177.1
-177.2
f1 (ppm)
F
Cl
Me
19F-NMR, 470 MHz, CDCl3
-132
-136
-140
-144
-148
-152
-156
-160
-164
-168
-172
-176
-180
-184
-188
f1 (ppm)

Compound 24

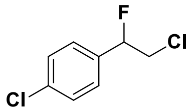

^{1}H-NMR, 400 MHz, $CDCl_3$

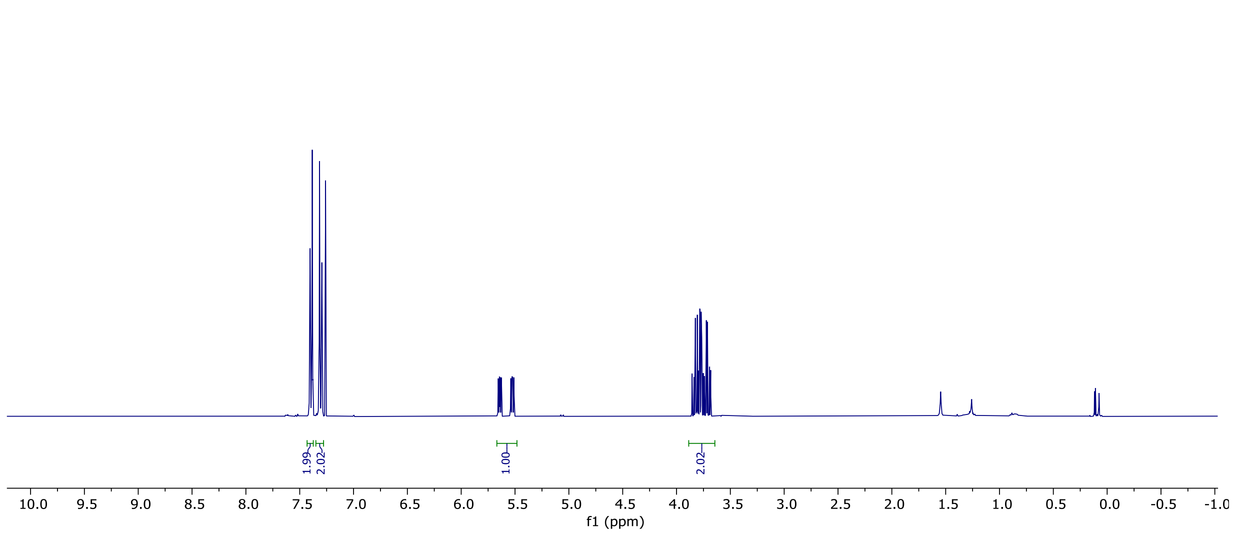

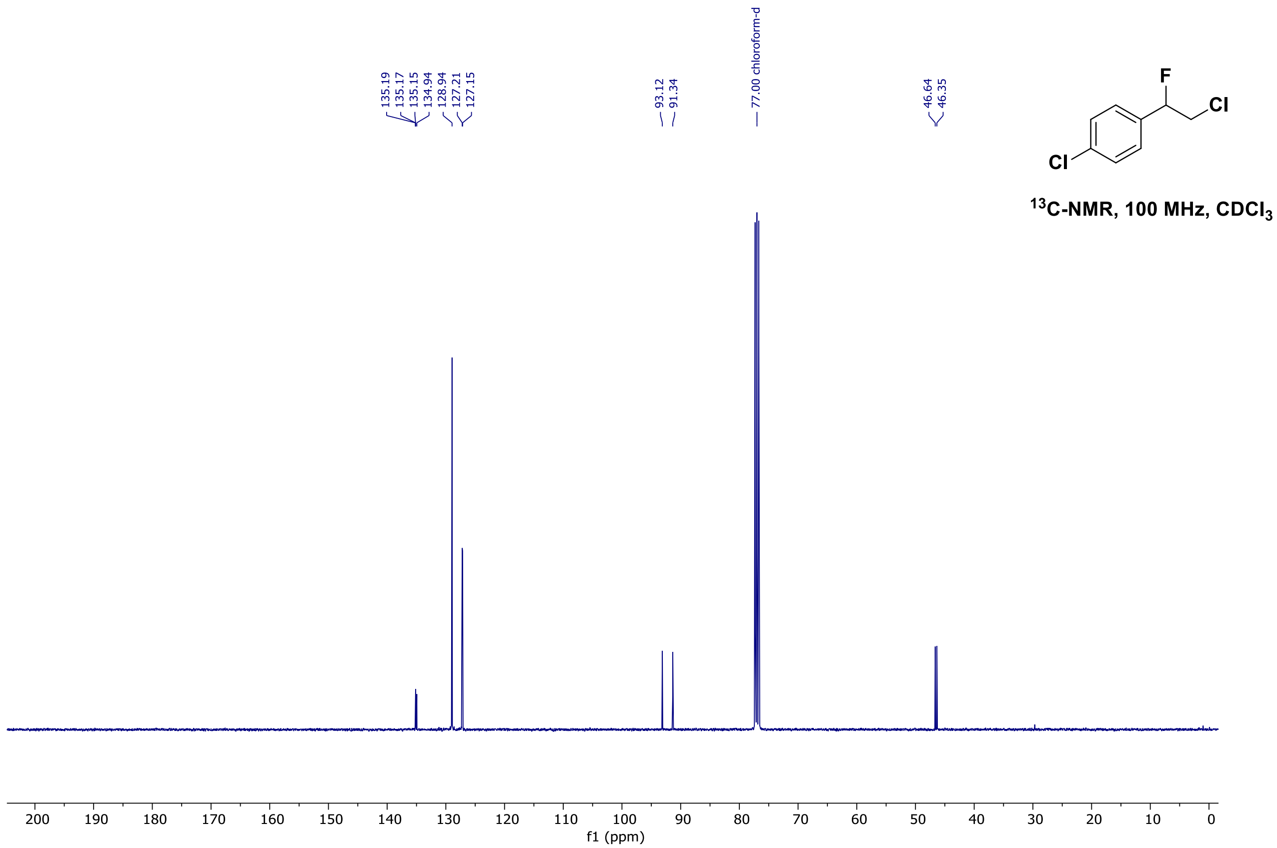
135.19
135.17
135.15
134.94
128.94
127.21
127.15
93.12
91.34
77.00 chloroform-d
46.64
46.35
F
Cl
Cl
^{13}C-NMR, 100 MHz, CDCl$_3$
200 190 180 170 160 150 140 130 120 110 100 90 80 70 60 50 40 30 20 10 0
f1 (ppm)

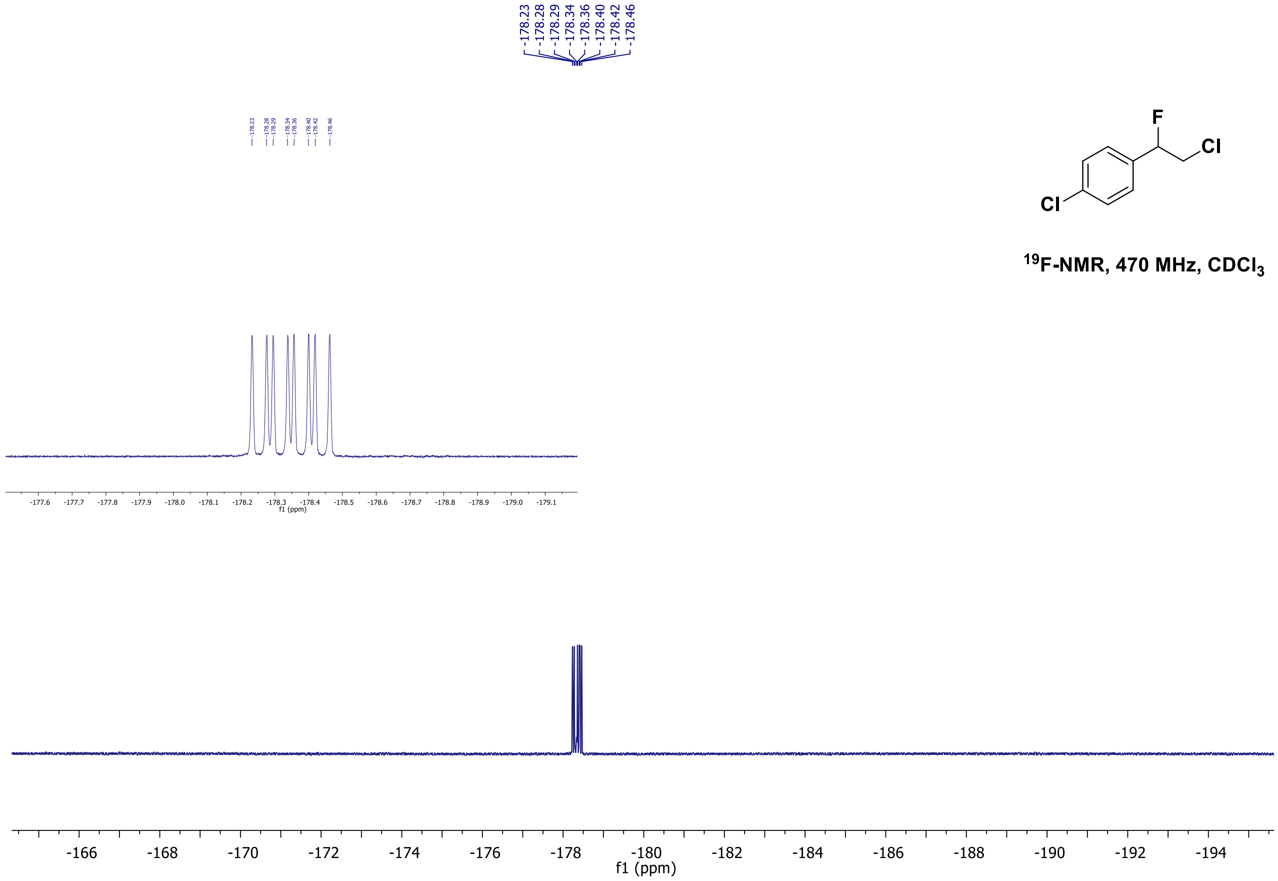
-178.23
-178.28
-178.29
-178.34
-178.36
-178.40
-178.42
-178.46
F
Cl
Cl
^{19}F-NMR, 470 MHz, CDCl$_3$
-177.6 -177.7 -177.8 -177.9 -178.0 -178.1 -178.2 -178.3 -178.4 -178.5 -178.6 -178.7 -178.8 -178.9 -179.0 -179.1
f1 (ppm)
-166 -168 -170 -172 -174 -176 -178 -180 -182 -184 -186 -188 -190 -192 -194
f1 (ppm)

Compound 25

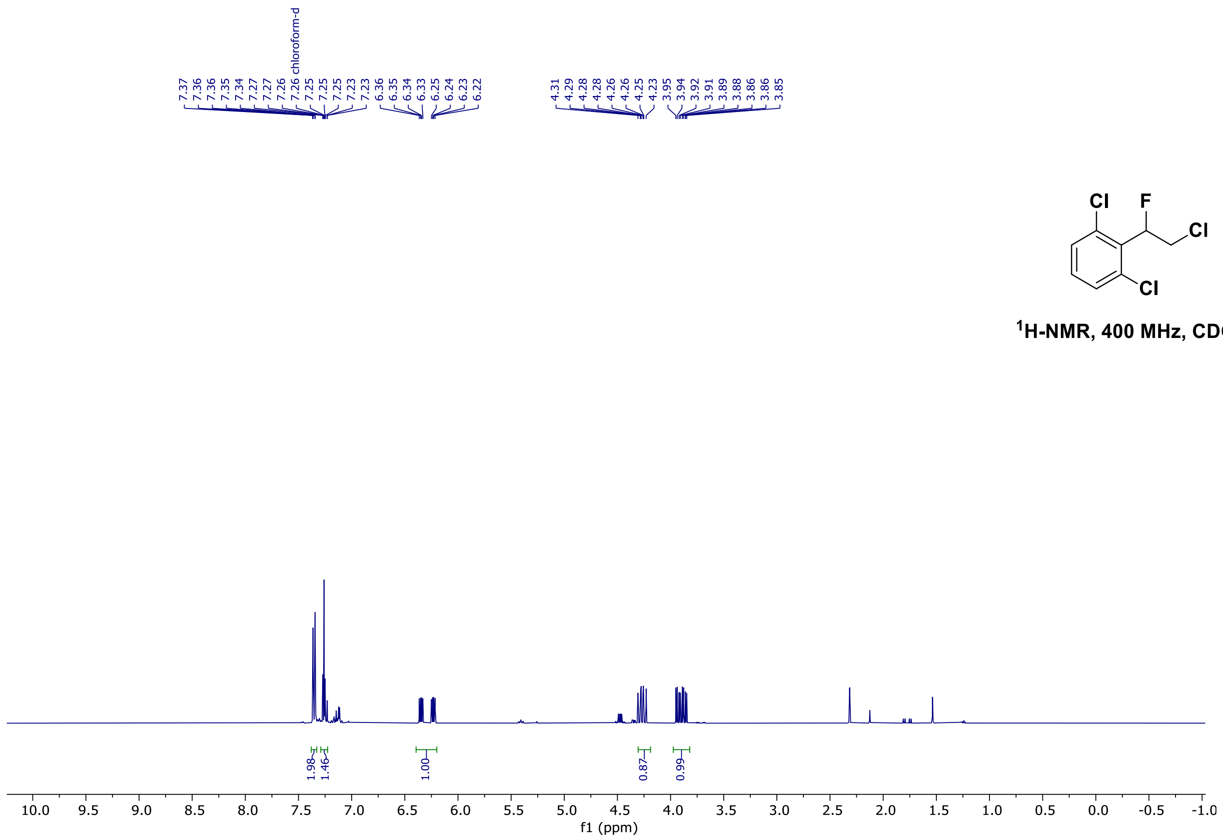

^{1}H-NMR, 400 MHz, $CDCl_3$

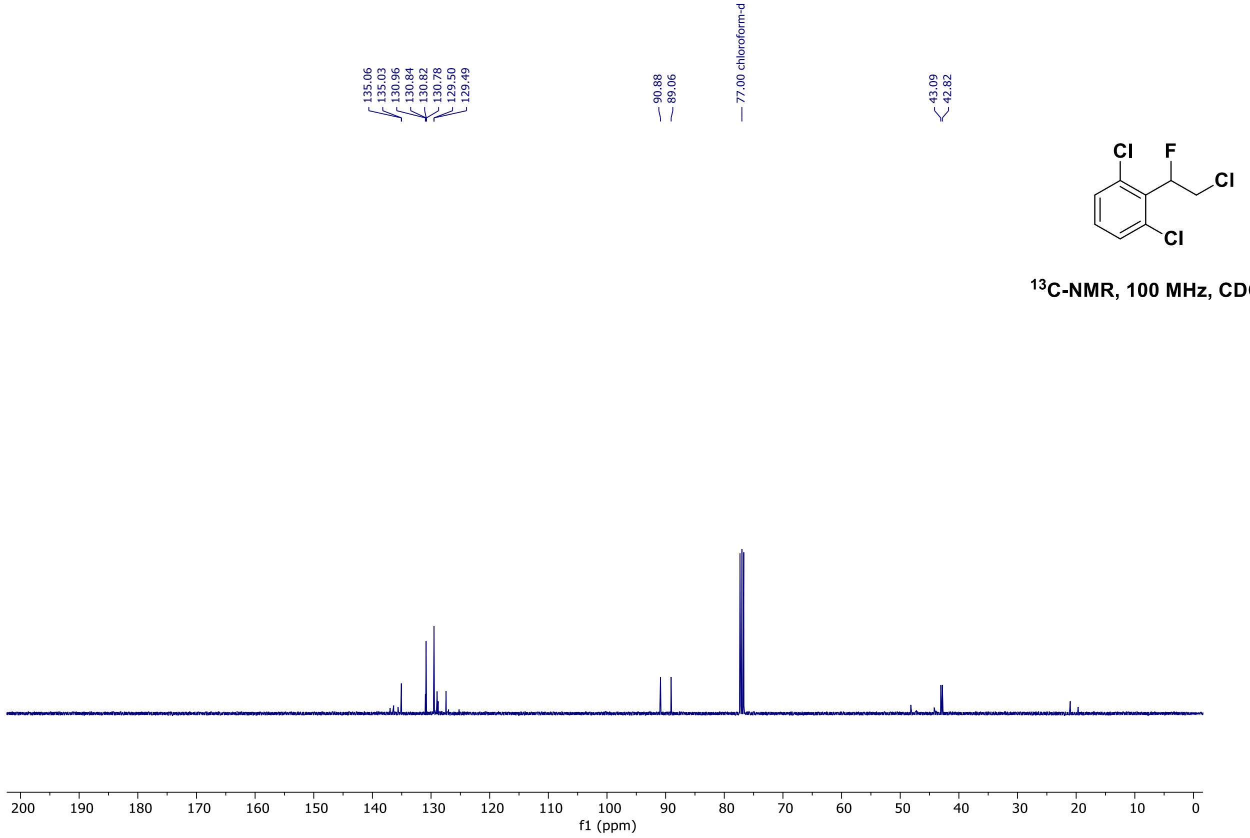

^{13}C-NMR, 100 MHz, $CDCl_3$

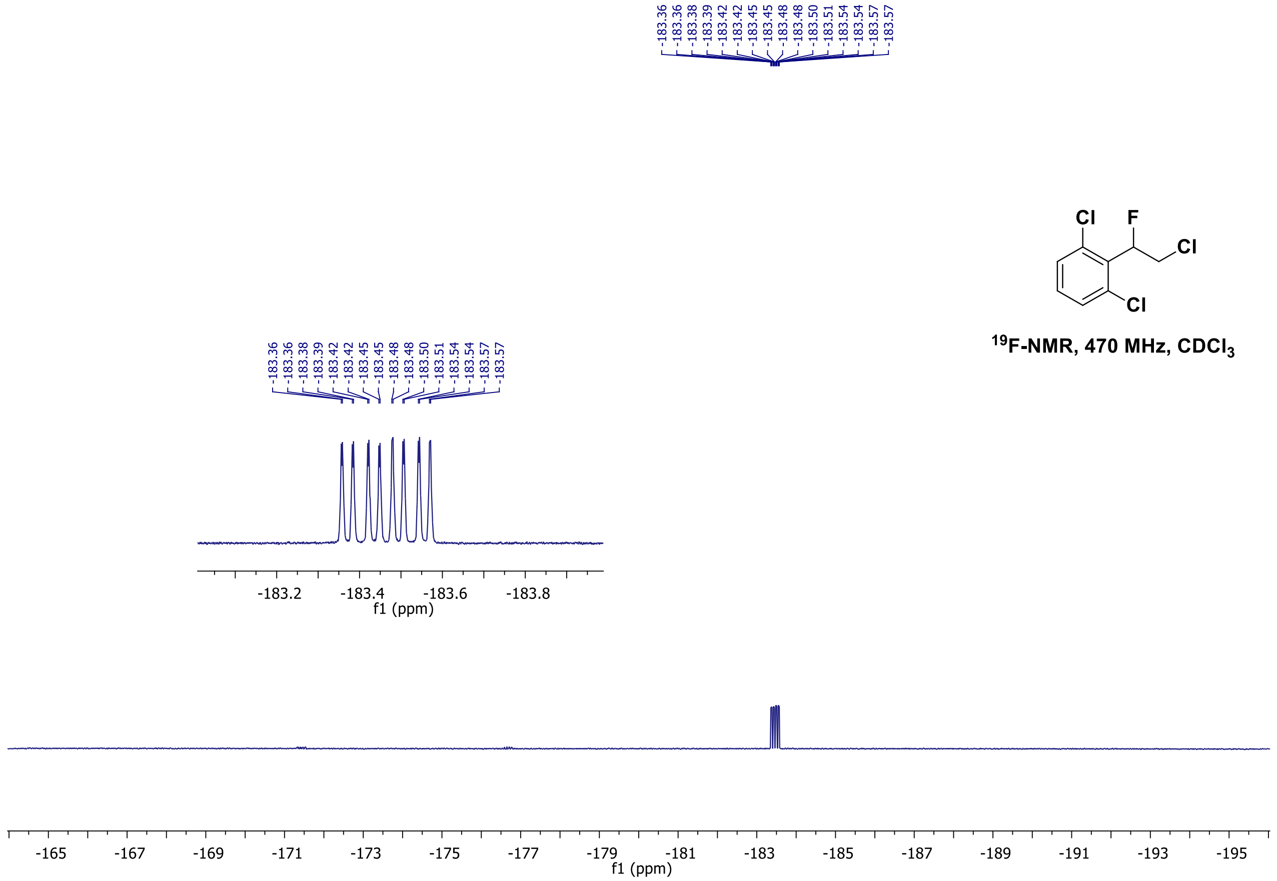
-183.36
-183.36
-183.38
-183.39
-183.42
-183.42
-183.45
-183.45
-183.48
-183.48
-183.50
-183.51
-183.54
-183.54
-183.57
-183.57
Cl
F
Cl
Cl
19F-NMR, 470 MHz, CDCl3
-183.2
-183.4
-183.6
-183.8
f1 (ppm)
-165
-167
-169
-171
-173
-175
-177
-179
-181
-183
-185
-187
-189
-191
-193
-195
f1 (ppm)

Compound 26

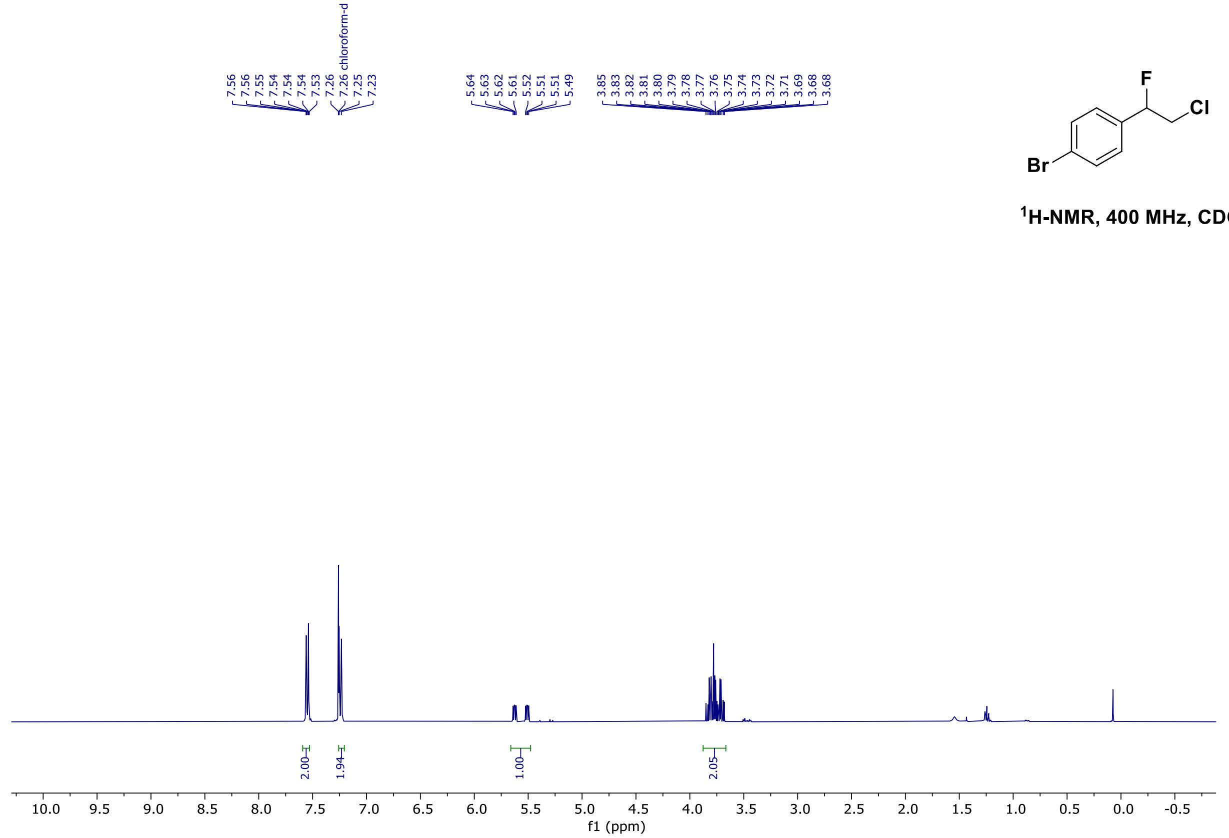

^{1}H-NMR, 400 MHz, CDCl$_3$

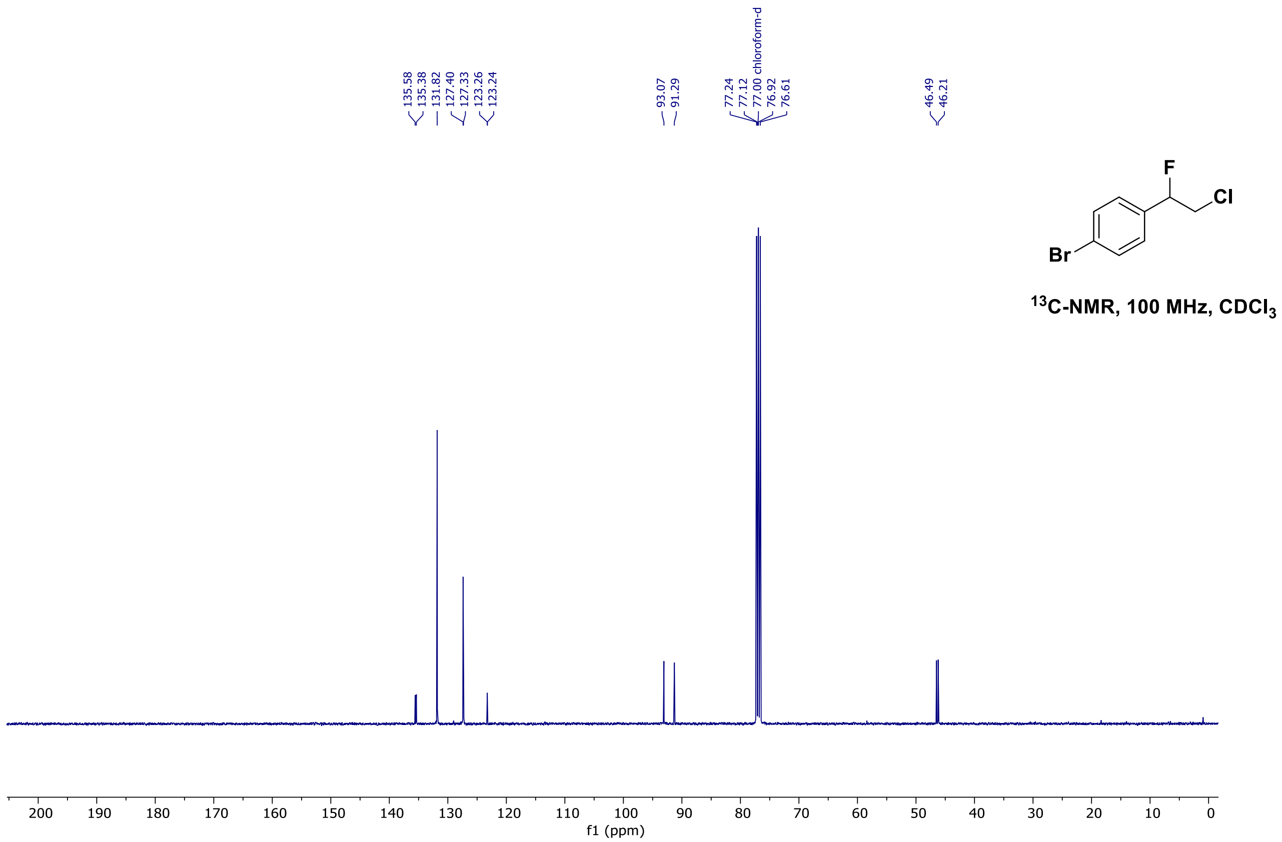
135.58
135.38
131.82
127.40
127.33
123.26
123.24
93.07
91.29
77.24
77.12
77.00 chloroform-d
76.92
76.61
46.49
46.21
F
Cl
Br
13C-NMR, 100 MHz, CDCl3
f1 (ppm)

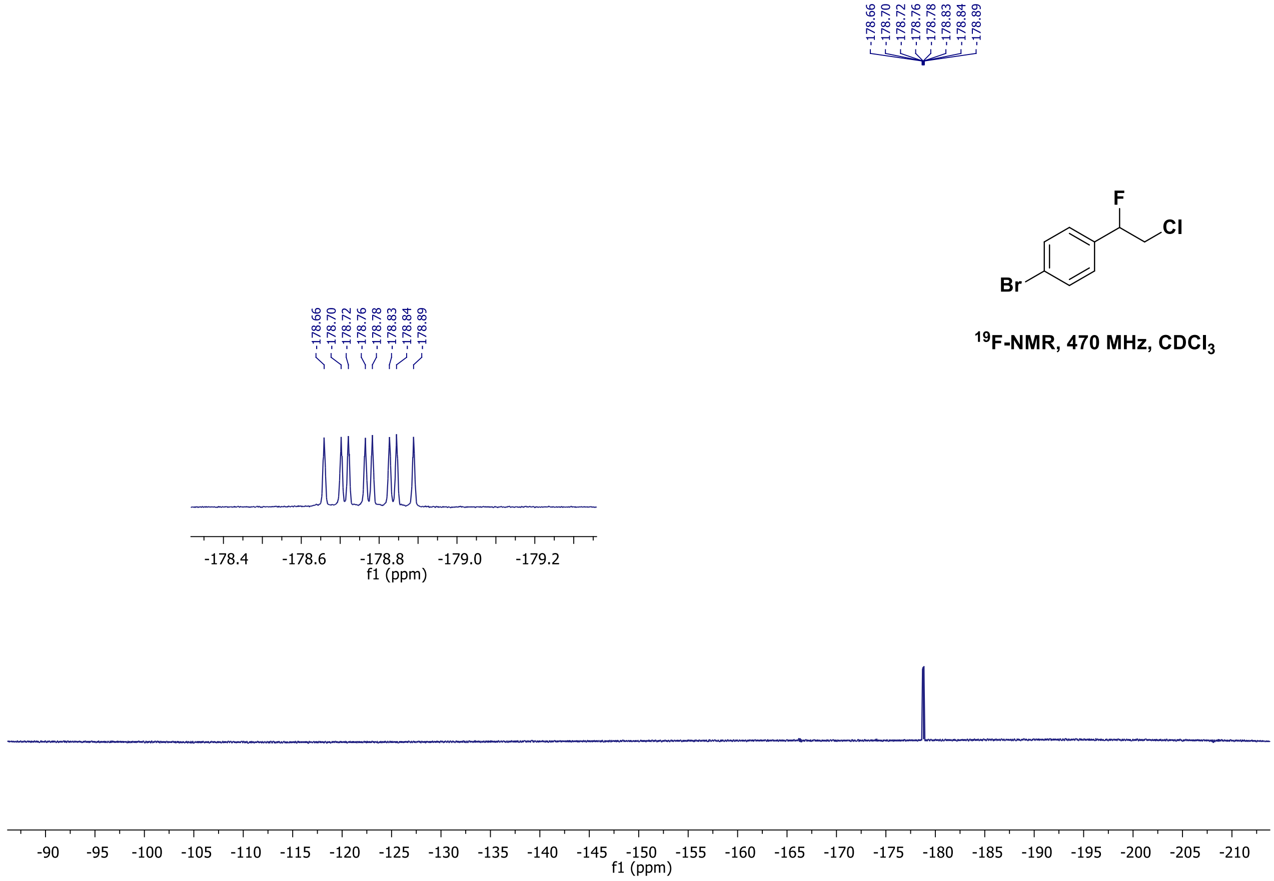
-178.66
-178.70
-178.72
-178.76
-178.78
-178.83
-178.84
-178.89
F
Cl
Br
19F-NMR, 470 MHz, CDCl3
f1 (ppm)

Compound 27

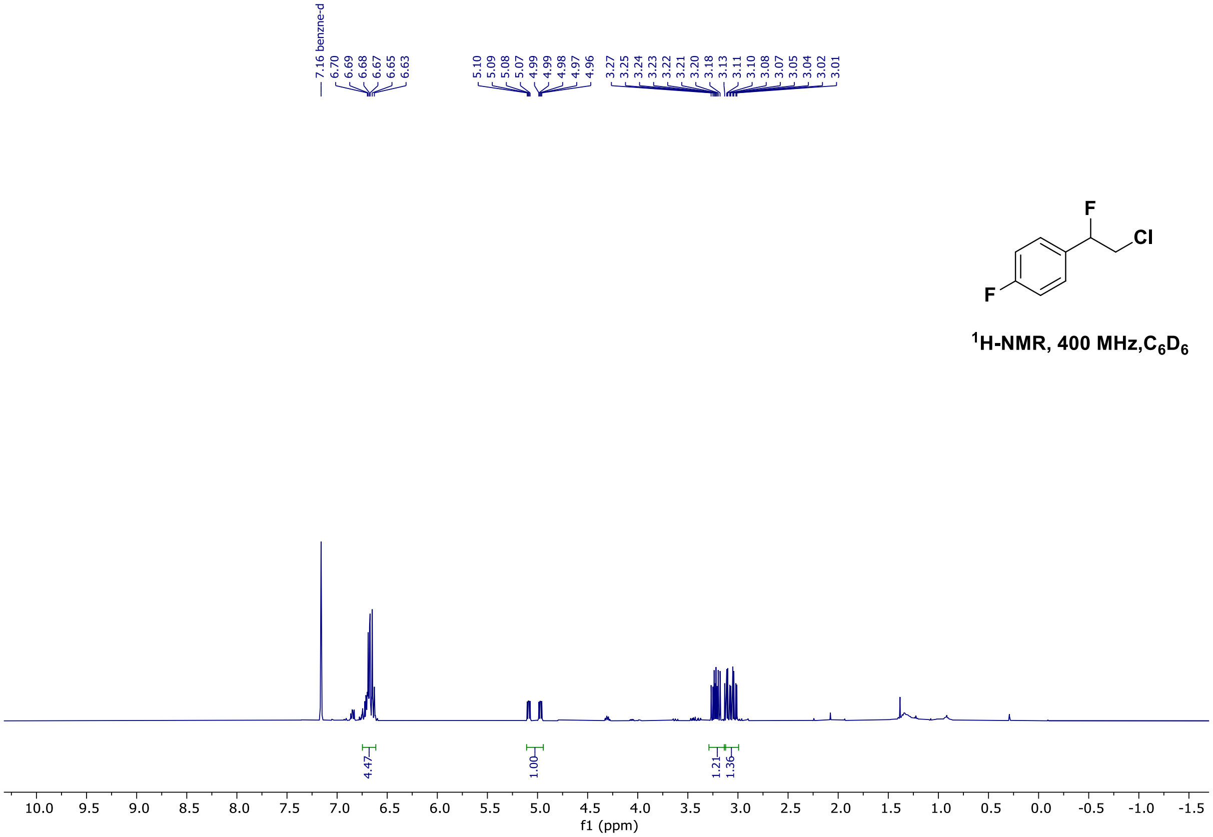

^{1}H-NMR, 400 MHz, C_6D_6

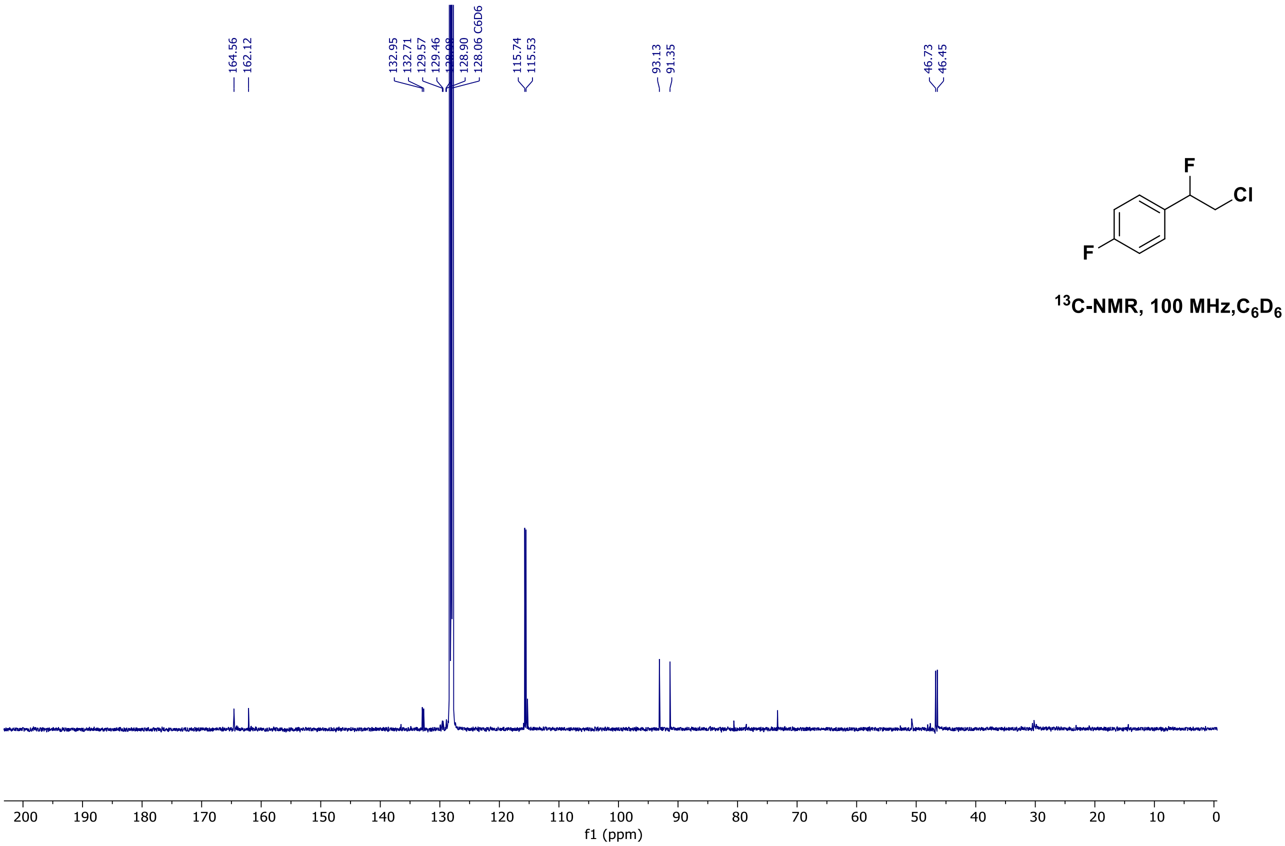

^{13}C-NMR, 100 MHz, C_6D_6

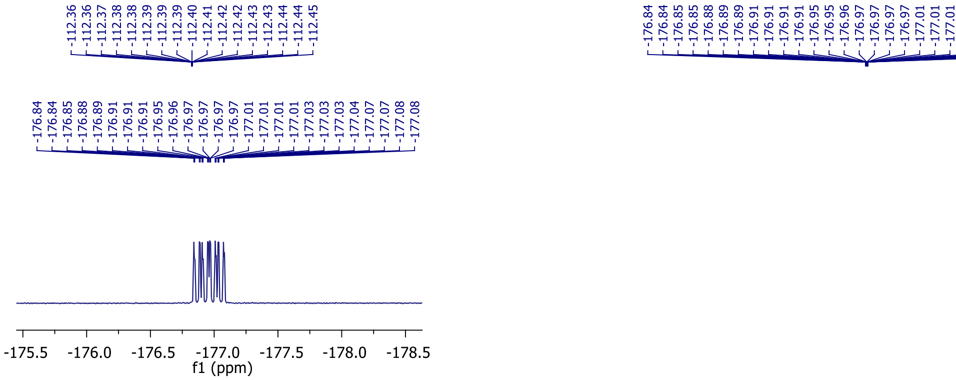

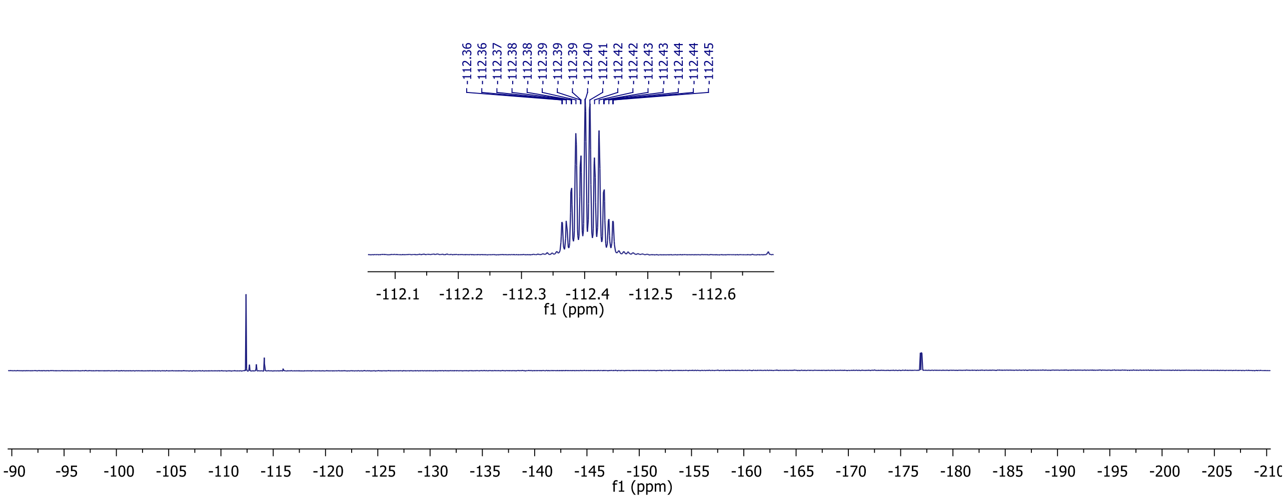

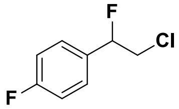

^{19}F-NMR, 470 MHz, C_6D_6

Compound 28

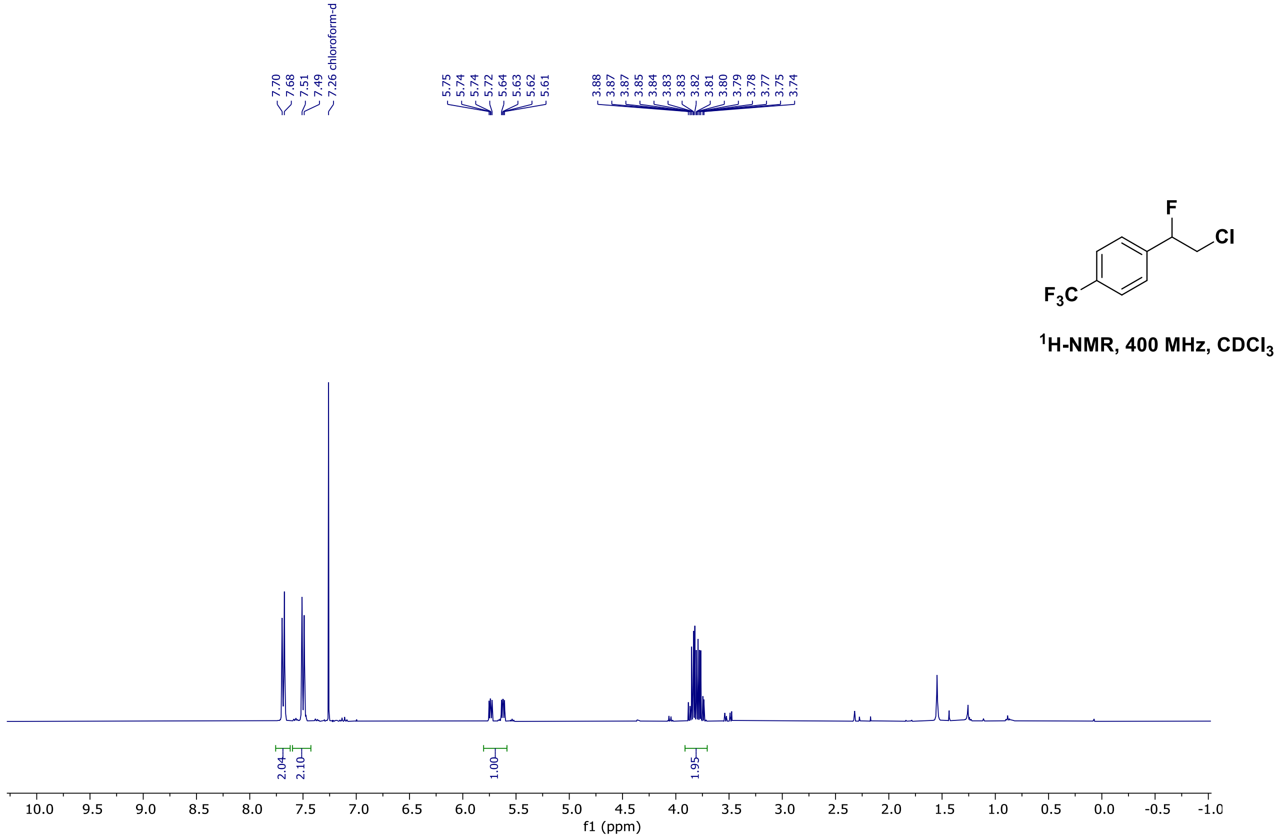

7.70
7.68
7.51
7.49
7.26 chloroform-d
5.75
5.74
5.74
5.72
5.64
5.63
5.62
5.61
3.88
3.87
3.87
3.85
3.84
3.83
3.83
3.82
3.81
3.80
3.79
3.78
3.77
3.75
3.74
F
Cl
F_3C
^{1}H-NMR, 400 MHz, $CDCl_3$
2.04
2.10
1.00
1.95
f1 (ppm)

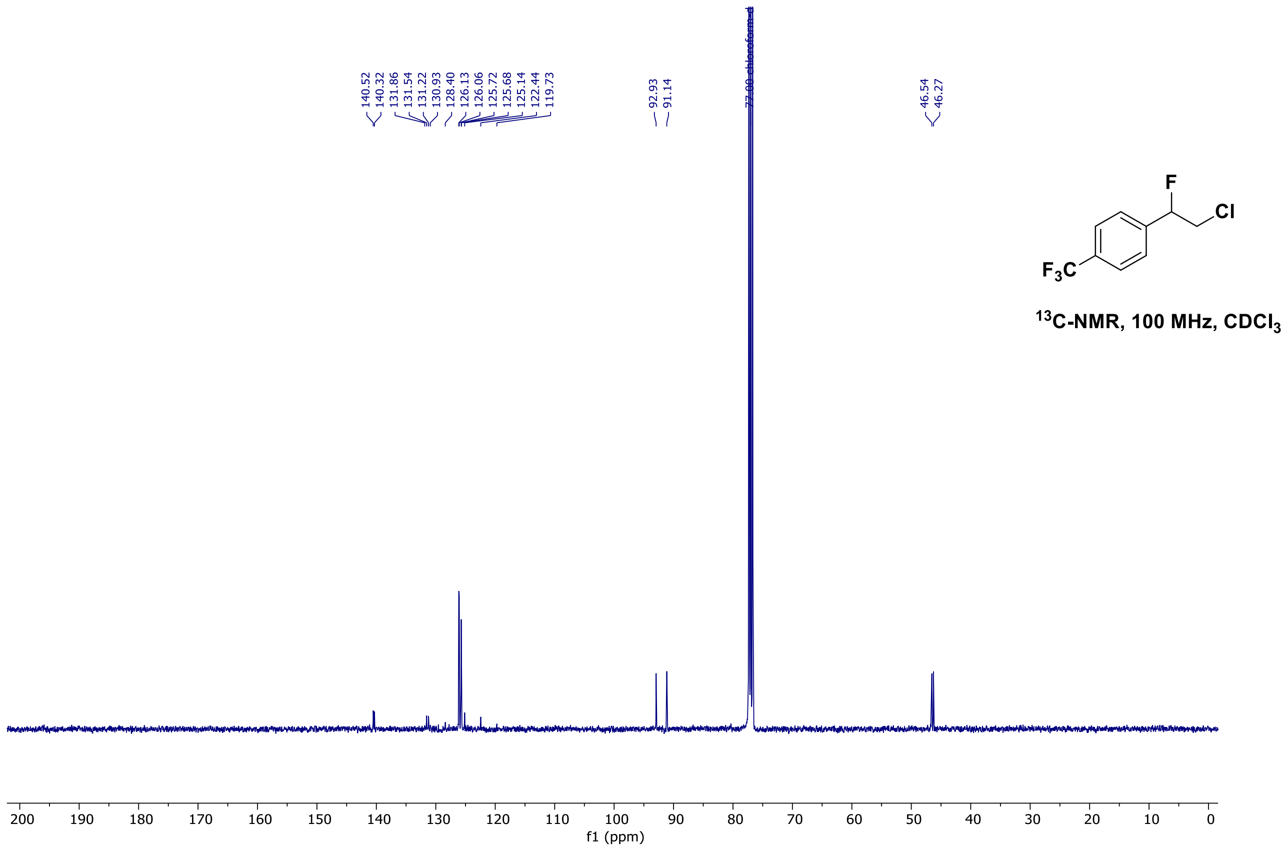
F
Cl
F_3C
^{13}C-NMR, 100 MHz, $CDCl_3$
f1 (ppm)

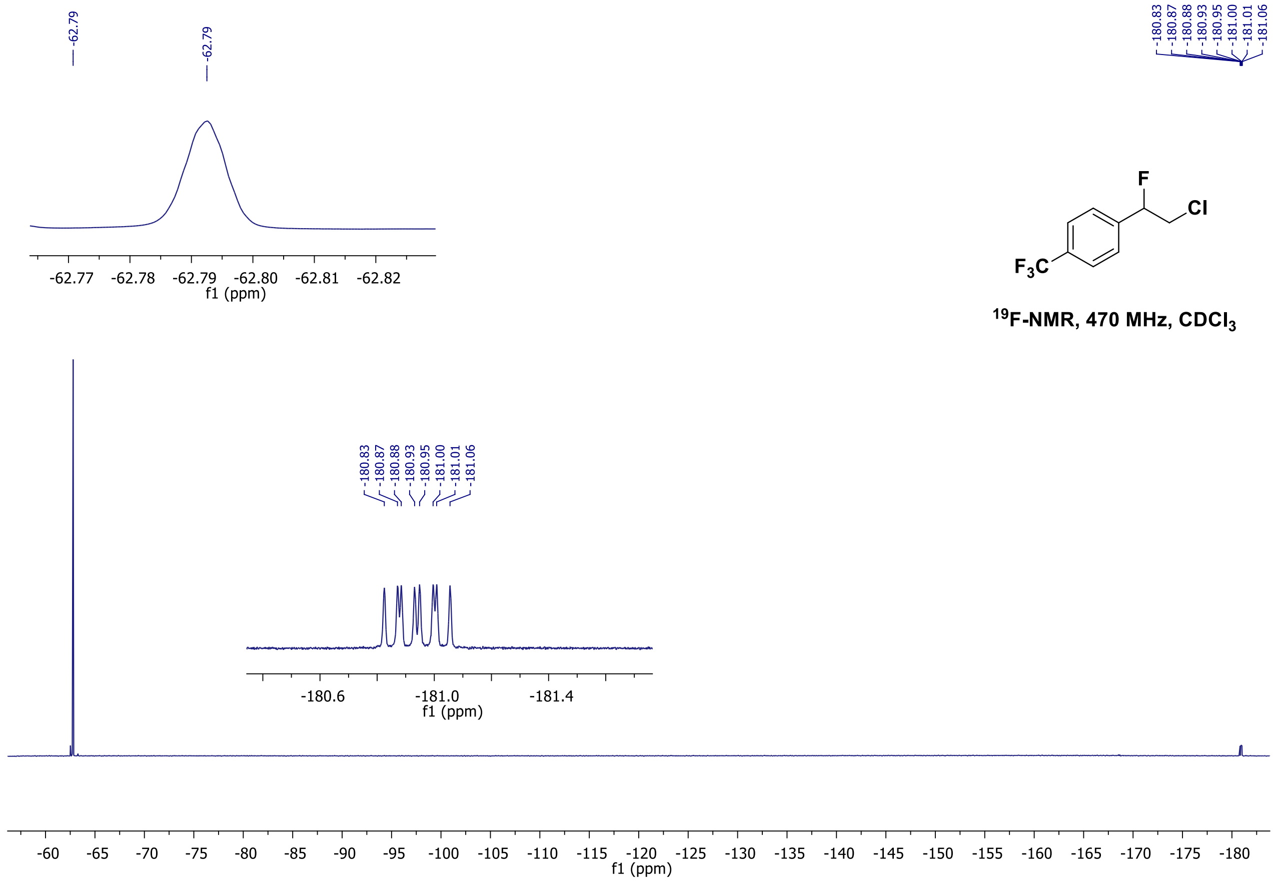
F
Cl
F_3C
^{19}F-NMR, 470 MHz, $CDCl_3$
f1 (ppm)

Compound 29

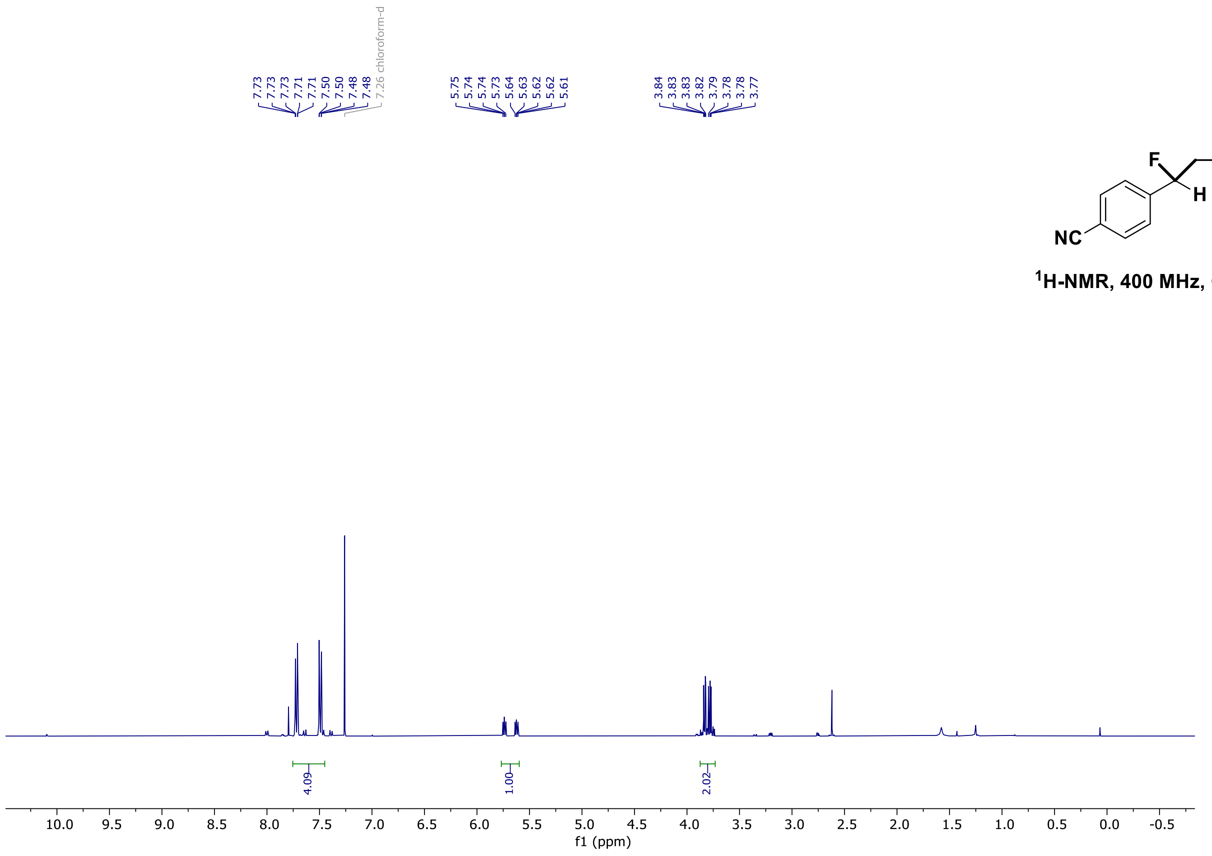

^{1}H-NMR, 400 MHz, $CDCl_3$

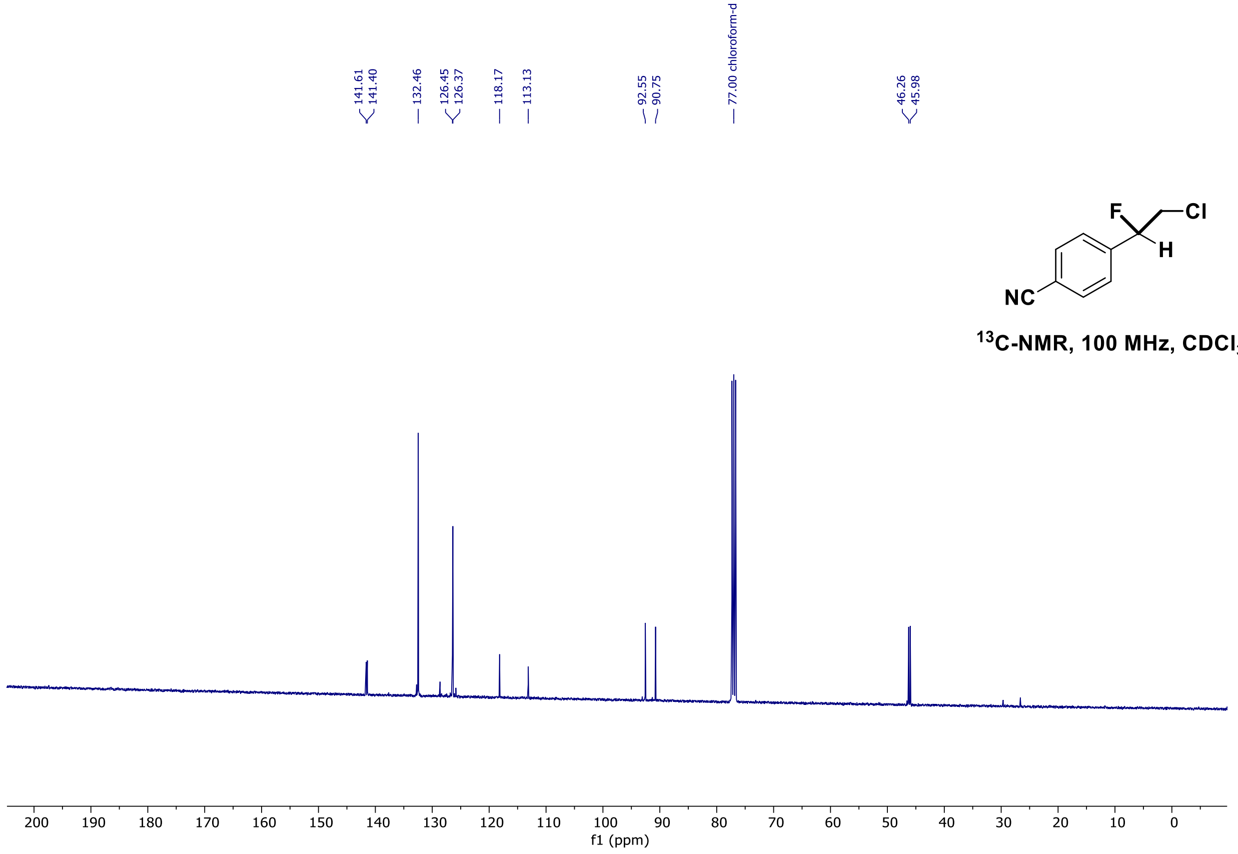

^{13}C-NMR, 100 MHz, $CDCl_3$

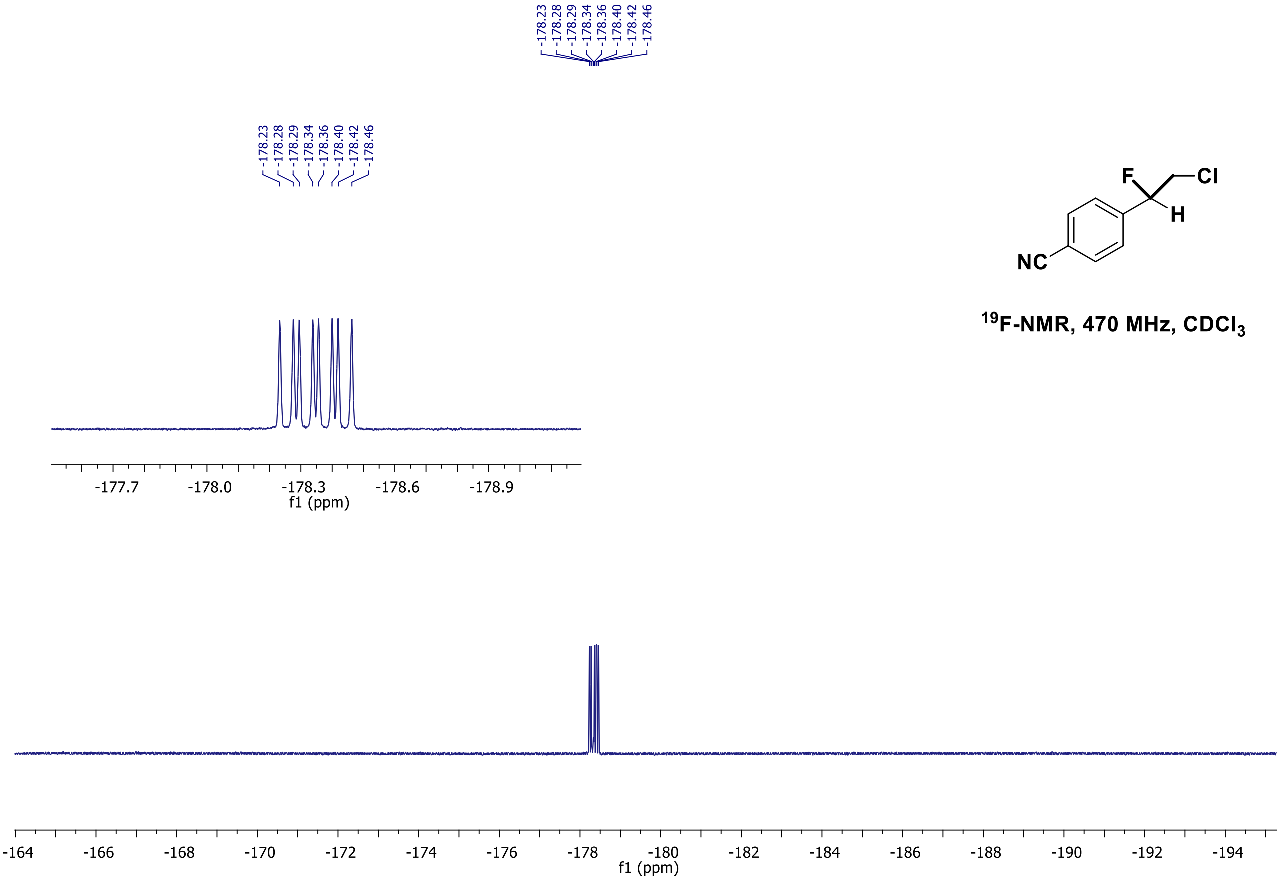

^{19}F-NMR, 470 MHz, $CDCl_3$

Compound 30

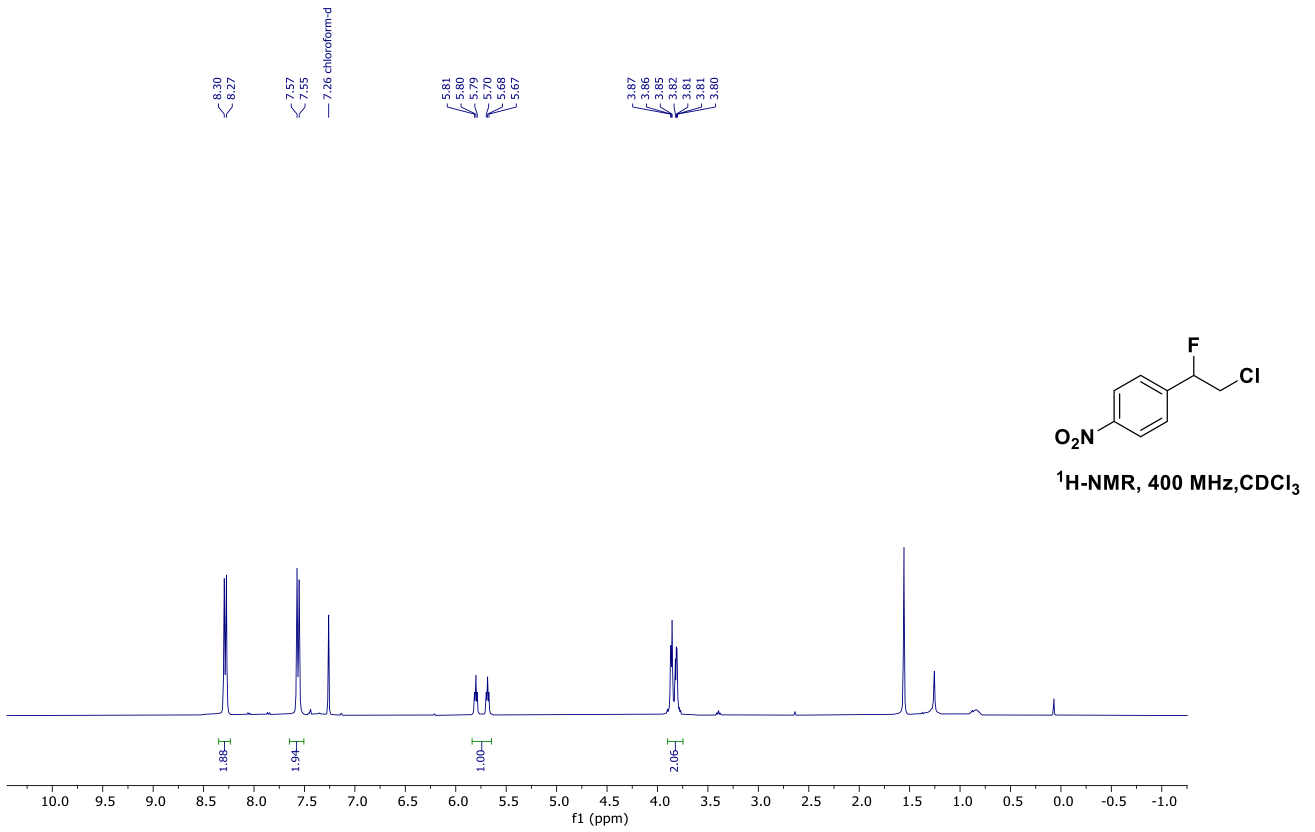
8.30
8.27
7.57
7.55
7.26 chloroform-d
5.81
5.80
5.79
5.70
5.68
5.67
3.87
3.86
3.85
3.82
3.81
3.81
3.80
F
Cl
O_2N
^{1}H-NMR, 400 MHz,$CDCl_3$
1.88
1.94
1.00
2.06
10.0 9.5 9.0 8.5 8.0 7.5 7.0 6.5 6.0 5.5 5.0 4.5 4.0 3.5 3.0 2.5 2.0 1.5 1.0 0.5 0.0 -0.5 -1.0
f1 (ppm)

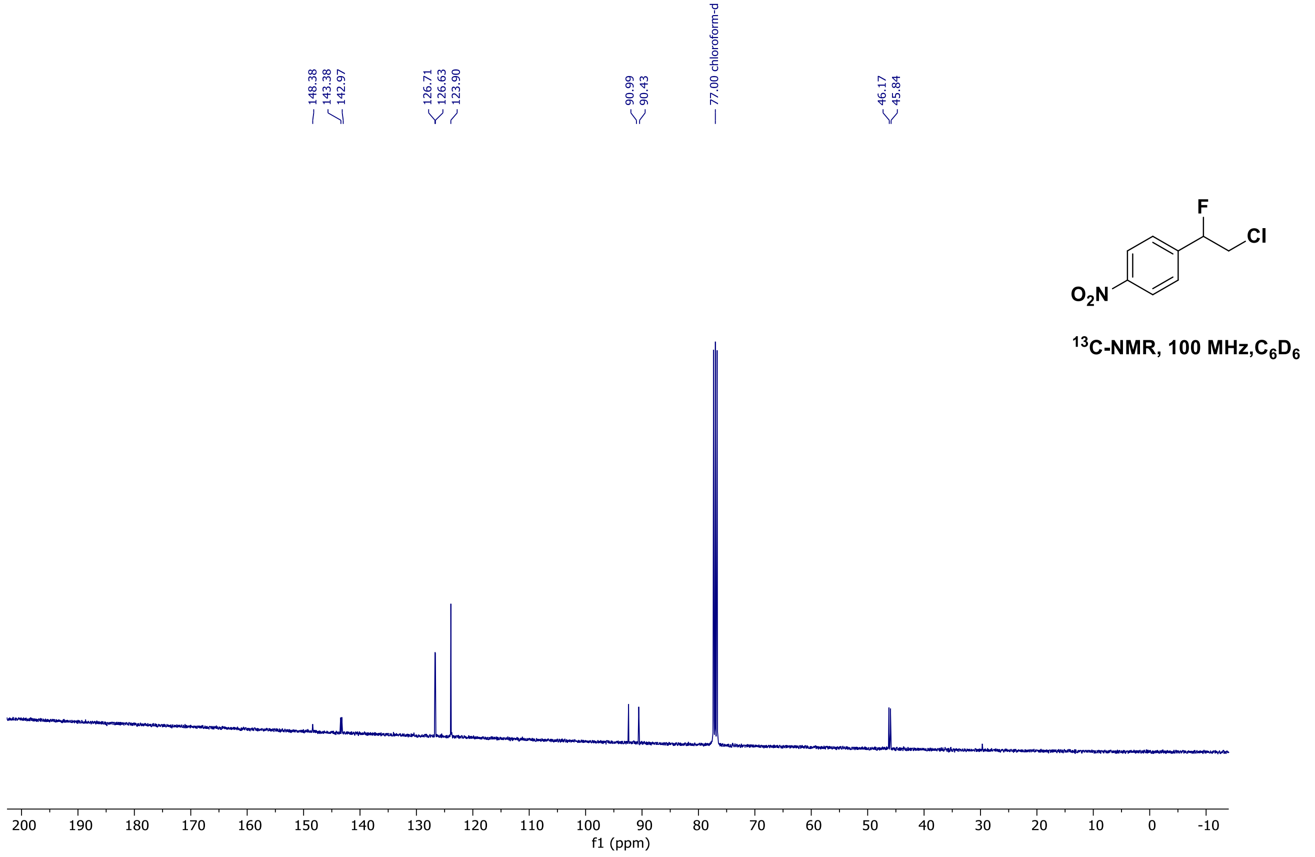
148.38
143.38
142.97
126.71
126.63
123.90
90.99
90.43
77.00 chloroform-d
46.17
45.84
F
Cl
O_2N
^{13}C-NMR, 100 MHz,C_6D_6
200 190 180 170 160 150 140 130 120 110 100 90 80 70 60 50 40 30 20 10 0 -10
f1 (ppm)

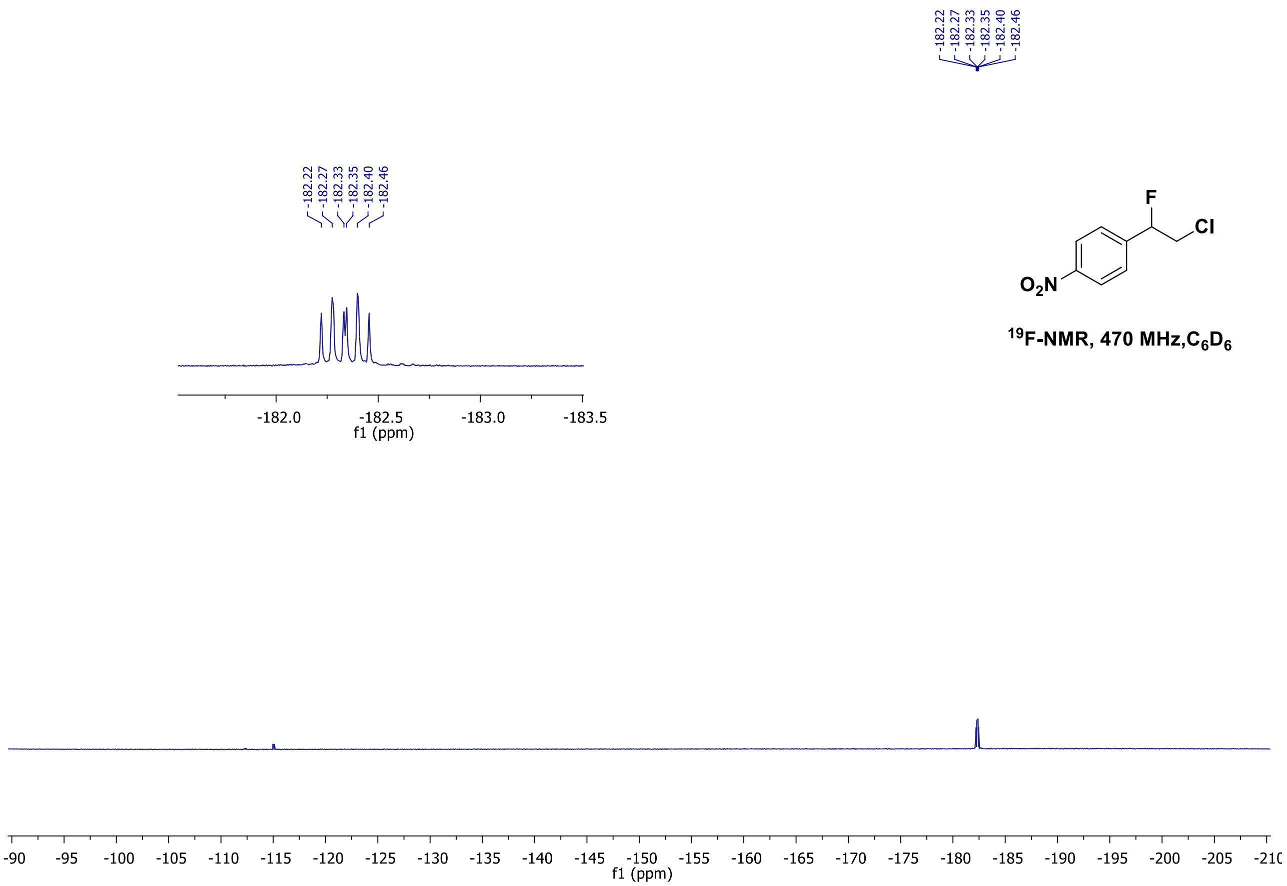
-182.22
-182.27
-182.33
-182.35
-182.40
-182.46
F
Cl
O2N
19F-NMR, 470 MHz,C6D6
-182.0
-182.5
-183.0
-183.5
f1 (ppm)
-90 -95 -100 -105 -110 -115 -120 -125 -130 -135 -140 -145 -150 -155 -160 -165 -170 -175 -180 -185 -190 -195 -200 -205 -210
f1 (ppm)

Compound 31

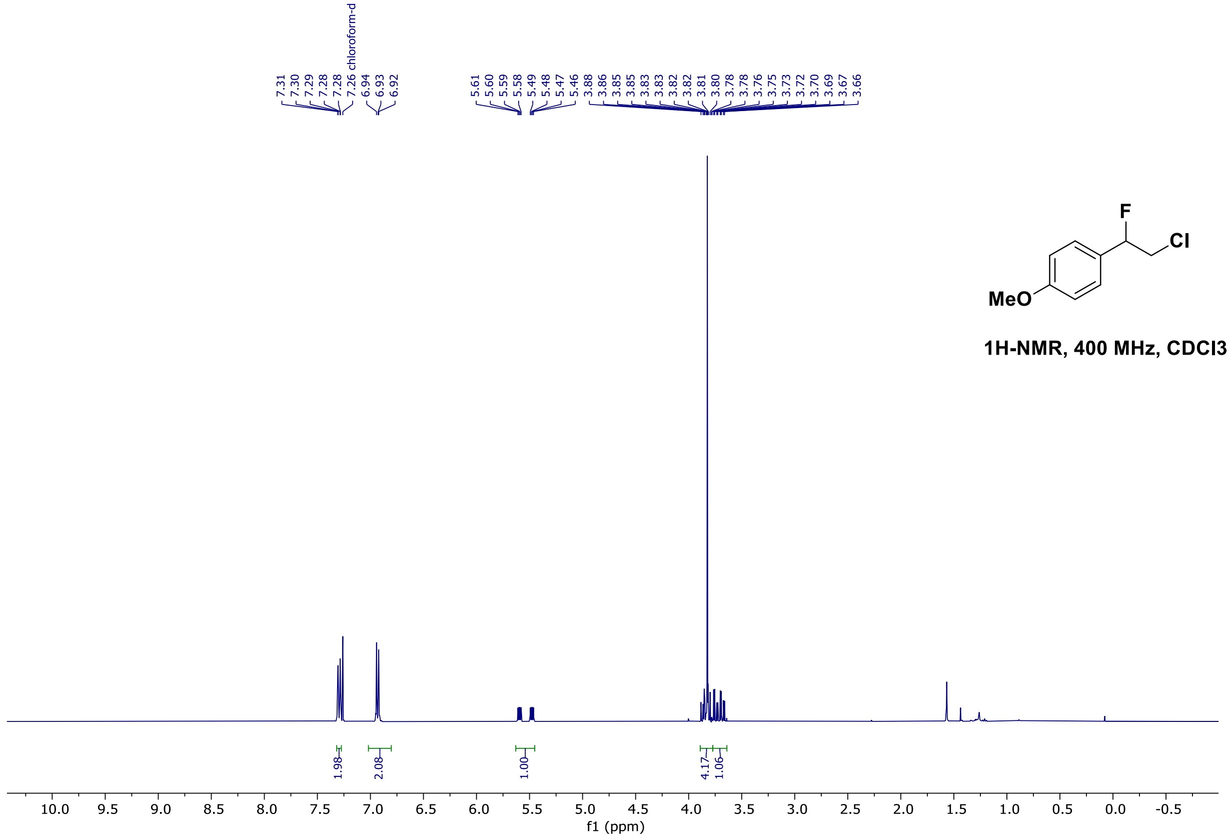
7.31
7.30
7.29
7.28
7.28
7.26 chloroform-d
6.94
6.93
6.92
5.61
5.60
5.59
5.58
5.49
5.48
5.47
5.46
3.88
3.86
3.85
3.85
3.83
3.83
3.82
3.82
3.81
3.80
3.78
3.78
3.76
3.75
3.73
3.72
3.70
3.69
3.67
3.66
F
Cl
MeO
1H-NMR, 400 MHz, CDCl3
1.98
2.08
1.00
4.17
1.06
10.0
9.5
9.0
8.5
8.0
7.5
7.0
6.5
6.0
5.5
5.0
4.5
4.0
3.5
3.0
2.5
2.0
1.5
1.0
0.5
0.0
-0.5
f1 (ppm)

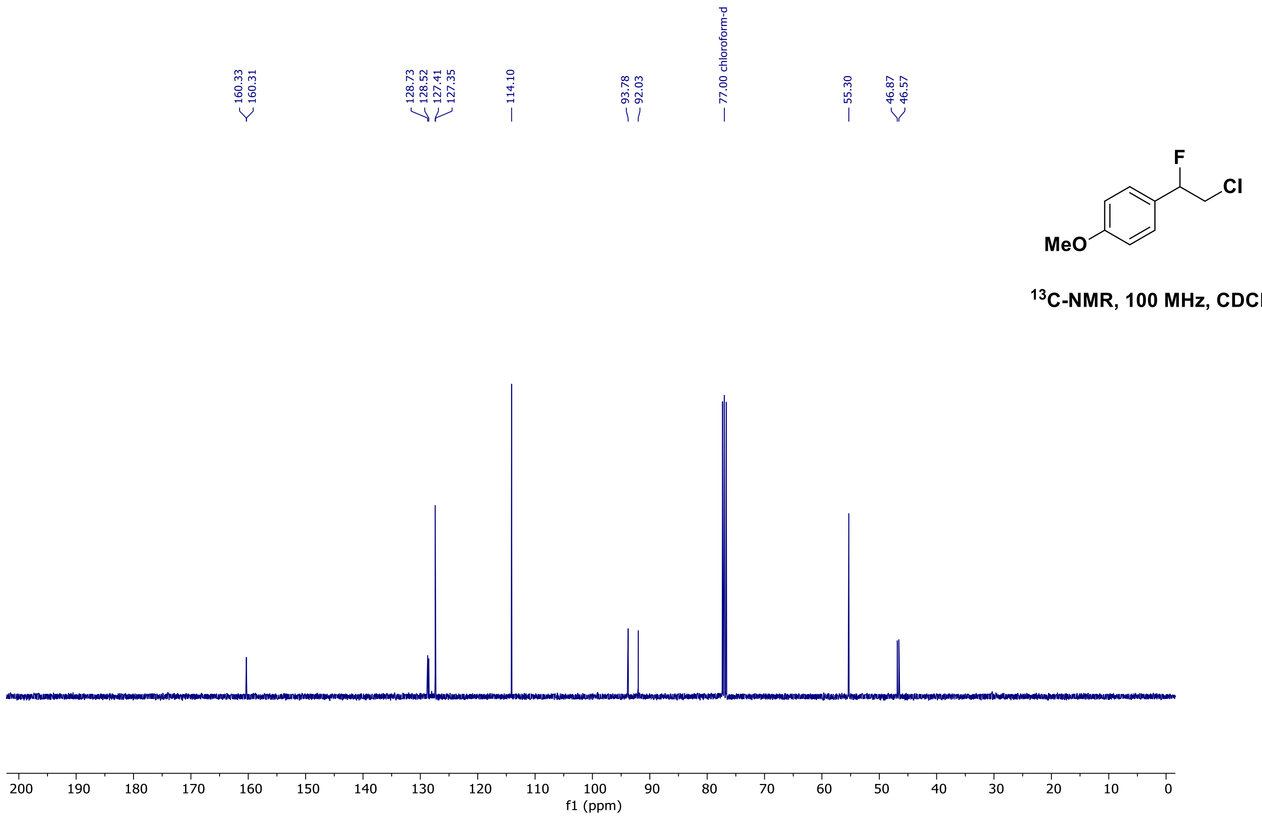

^{13}C-NMR, 100 MHz, $CDCl_3$

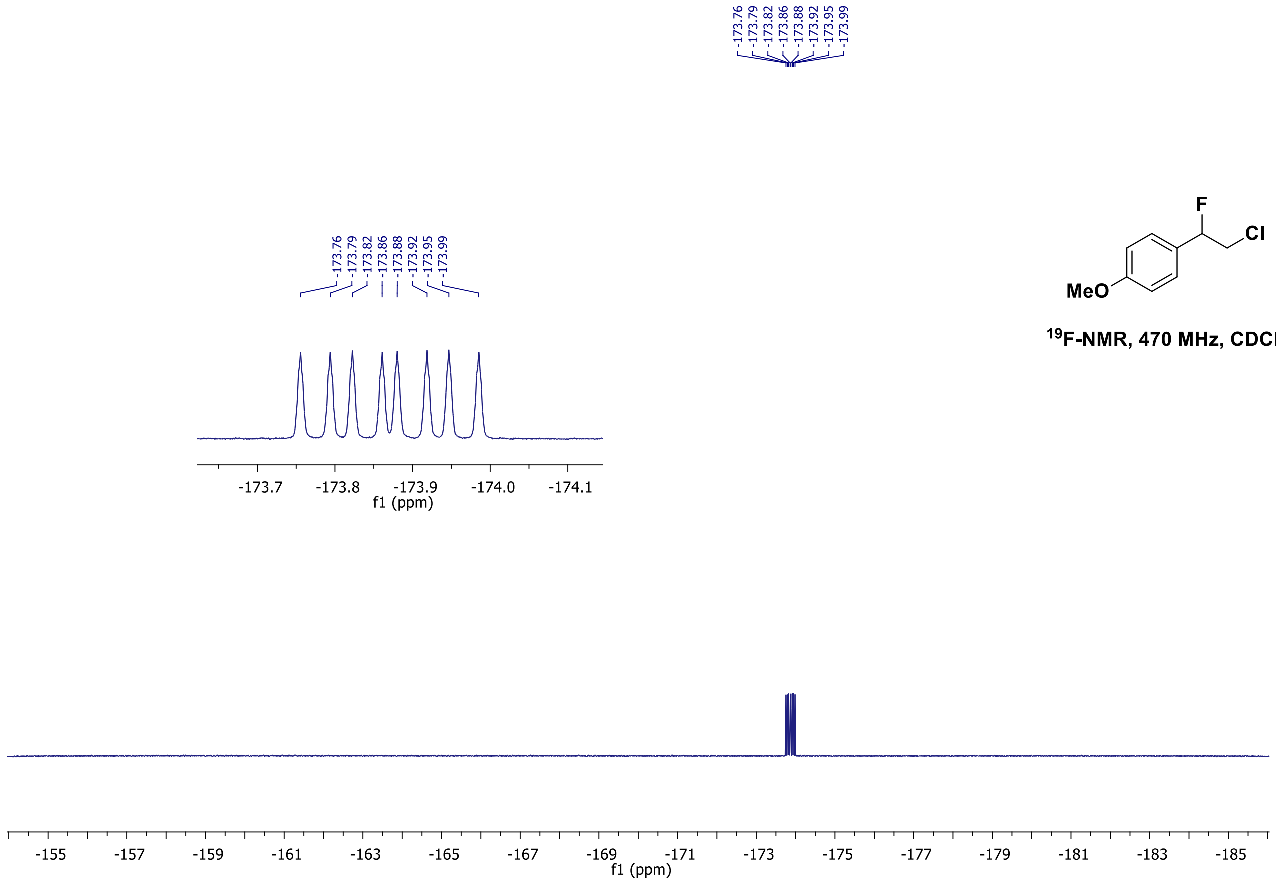

^{19}F-NMR, 470 MHz, $CDCl_3$

Compound 32

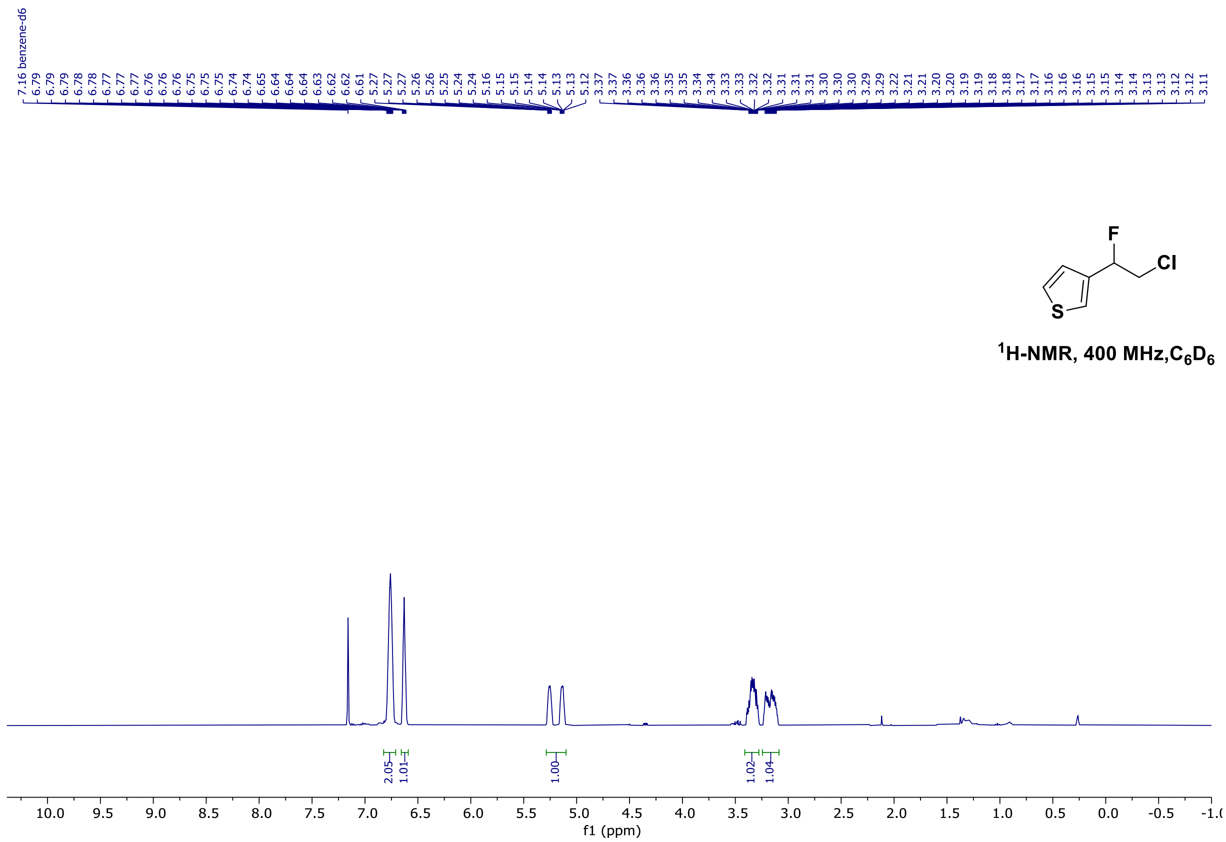
F
Cl
S
^{1}H-NMR, 400 MHz, C_6D_6
7.16 benzene-d6
f1 (ppm)
2.05
1.01
1.00
1.02
1.04

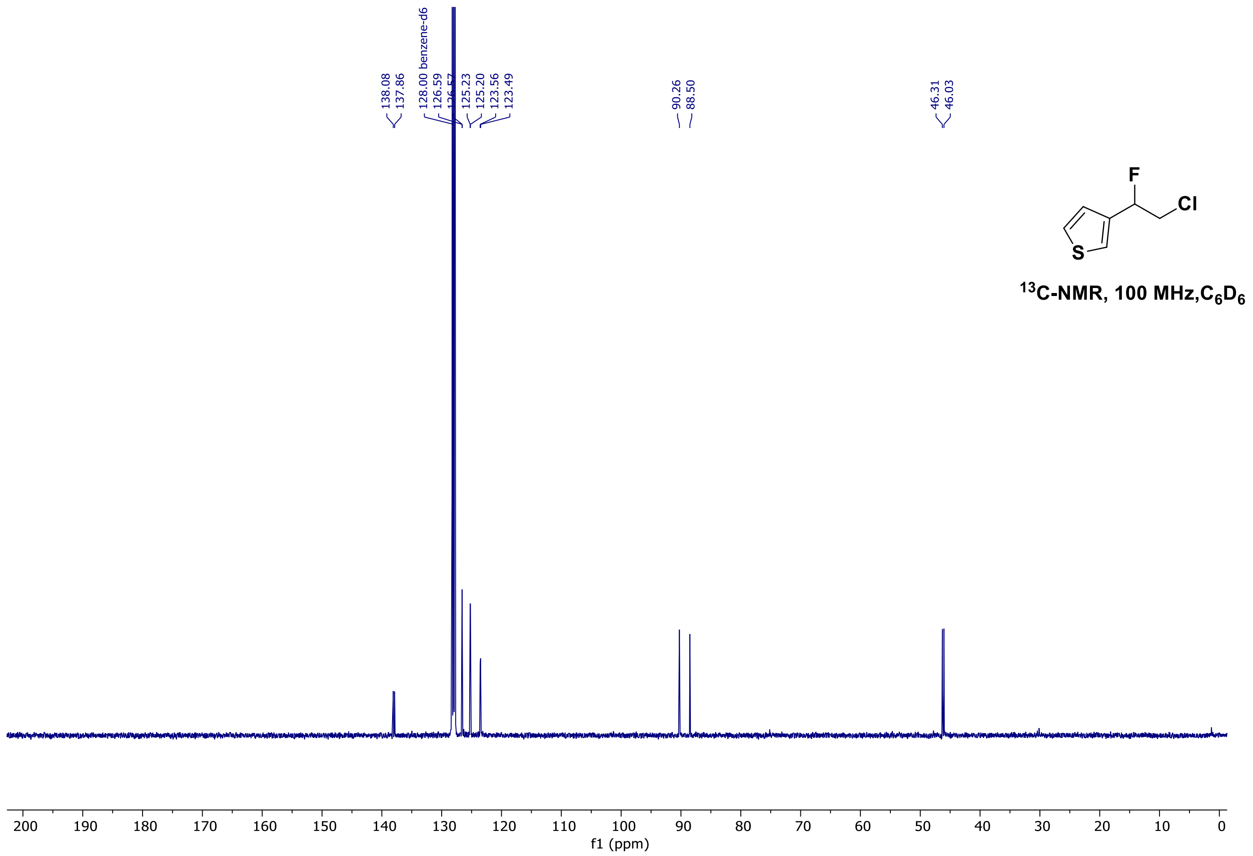
138.08
137.86
128.00 benzene-d6
126.59
126.57
125.23
125.20
123.56
123.49
90.26
88.50
46.31
46.03
F
Cl
S
13C-NMR, 100 MHz,C6D6
200
190
180
170
160
150
140
130
120
110
100
90
80
70
60
50
40
30
20
10
0
f1 (ppm)

^{19}F-NMR, 470 MHz,C_6D_6

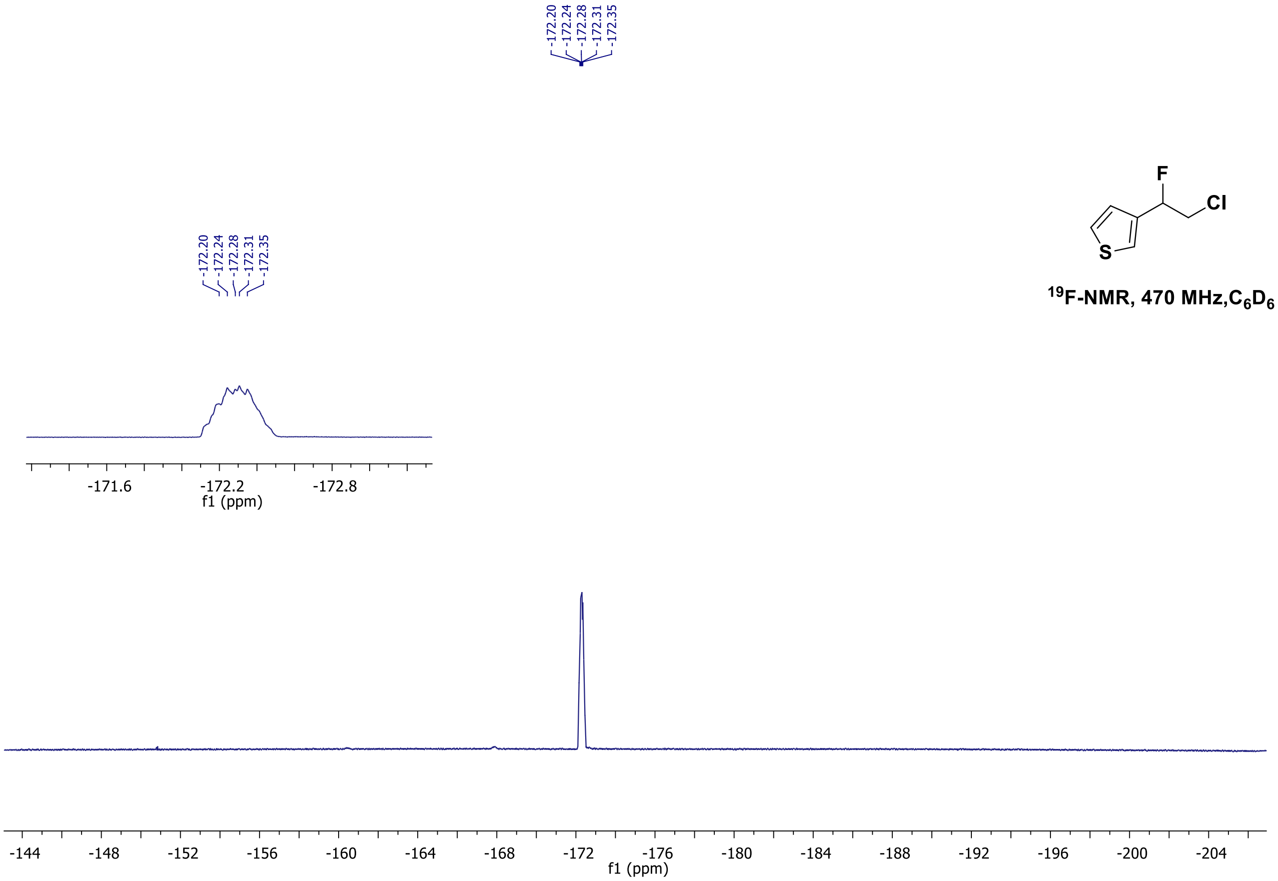

Compound 33

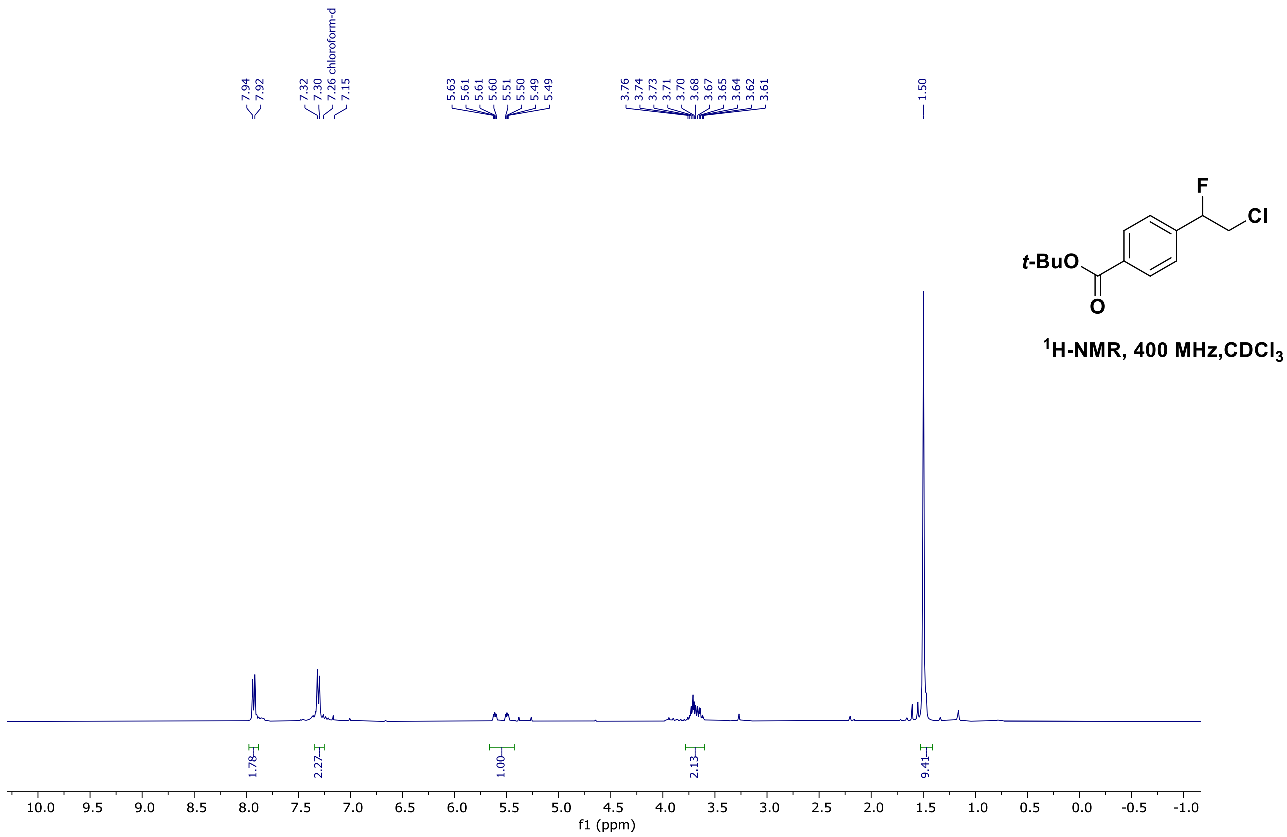

^{1}H-NMR, 400 MHz, $CDCl_3$

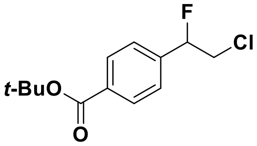

^{13}C-NMR, 100 MHz, $CDCl_3$

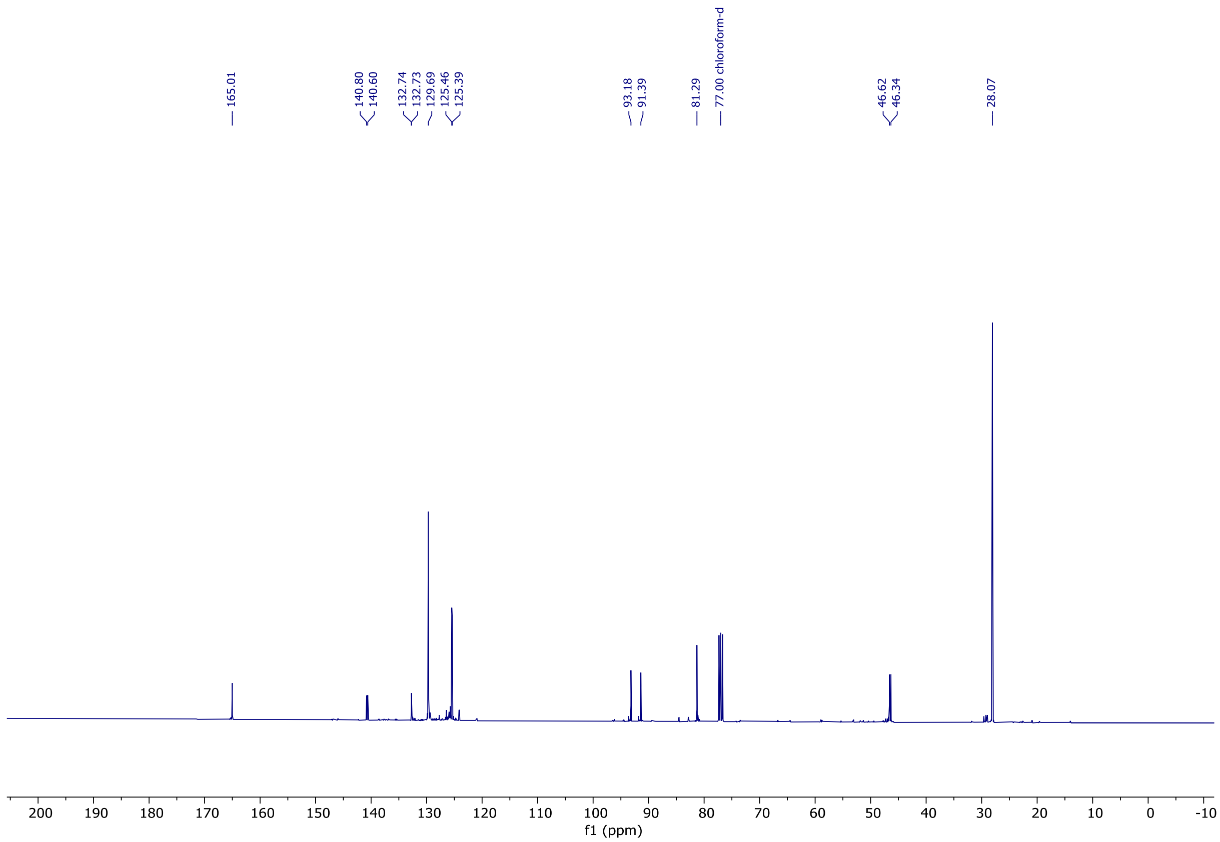

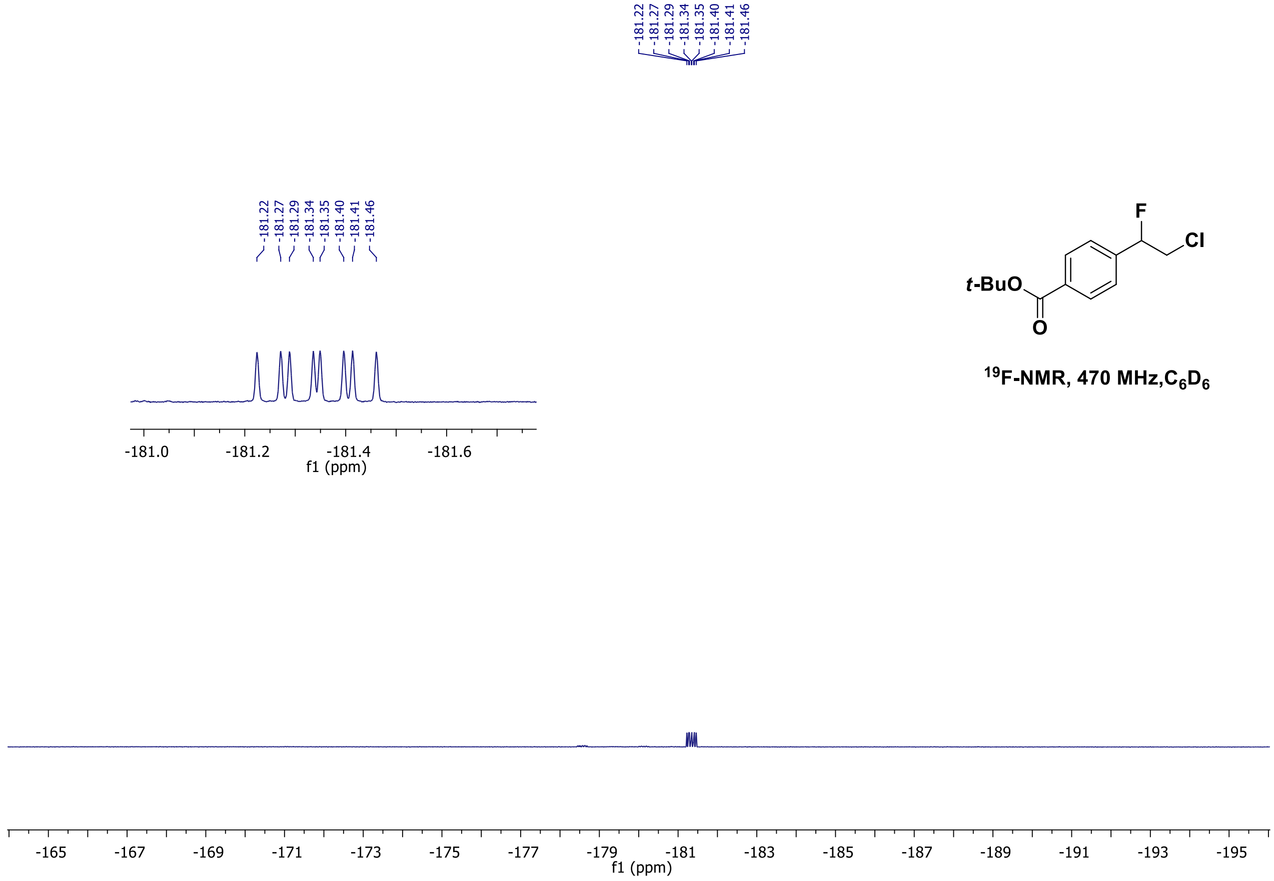

^{19}F-NMR, 470 MHz, C_6D_6

Compound 34

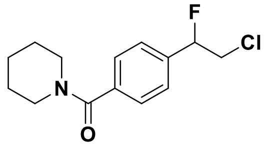

^{1}H-NMR, 400 MHz, $CDCl_3$

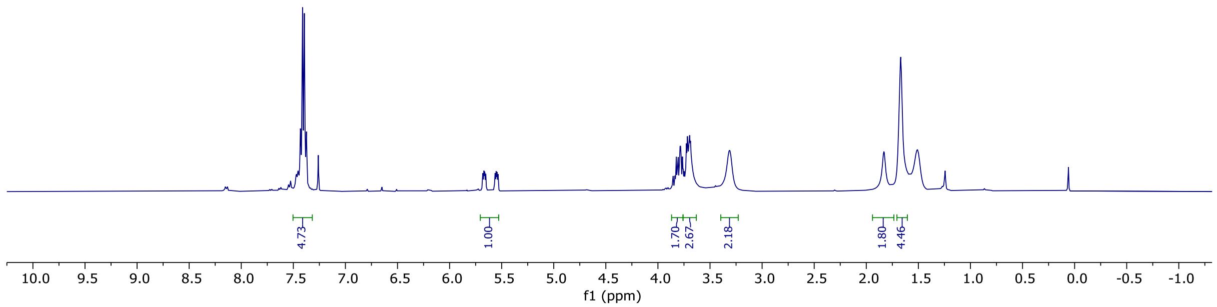

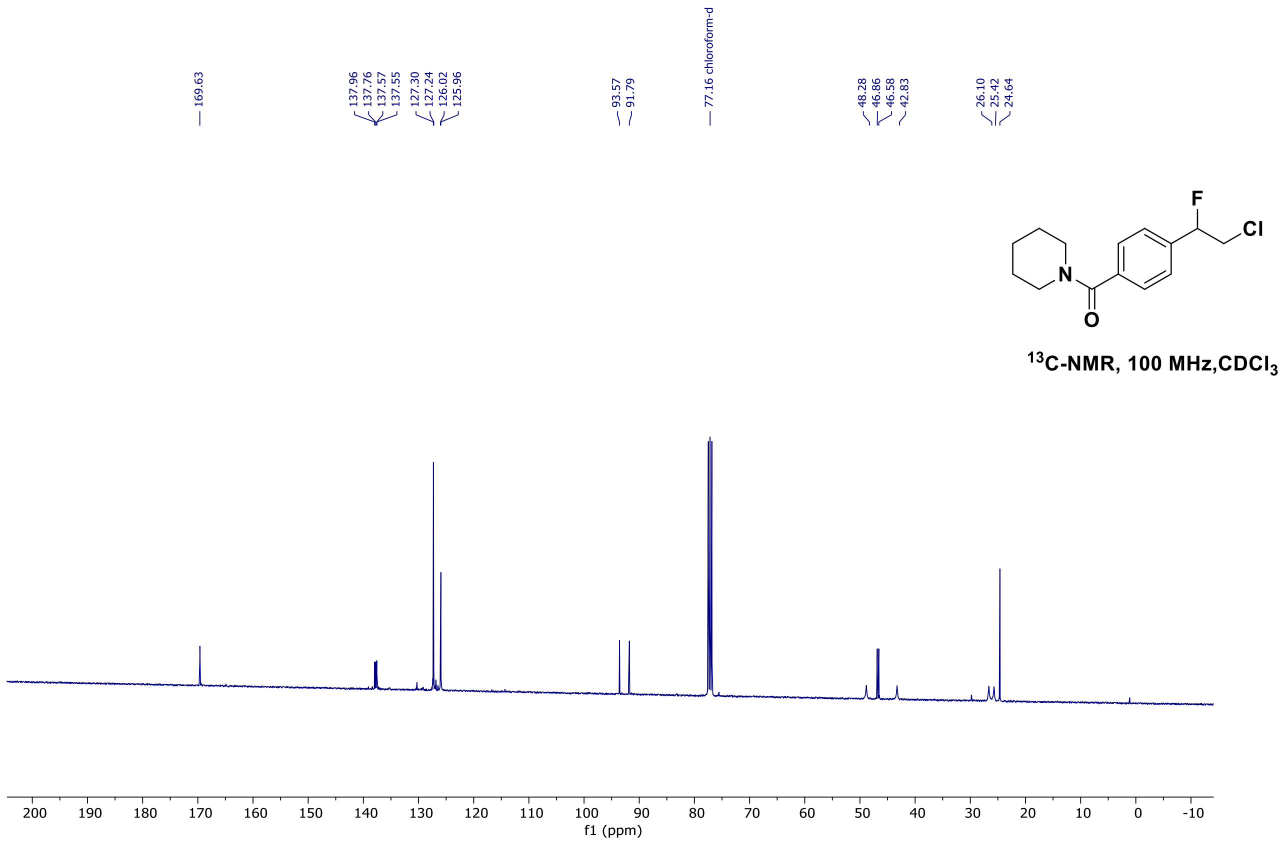
169.63
137.96
137.76
137.57
137.55
127.30
127.24
126.02
125.96
93.57
91.79
77.16 chloroform-d
48.28
46.86
46.58
42.83
26.10
25.42
24.64
F
Cl
N
O
13C-NMR, 100 MHz,CDCl3
f1 (ppm)

Compound 35

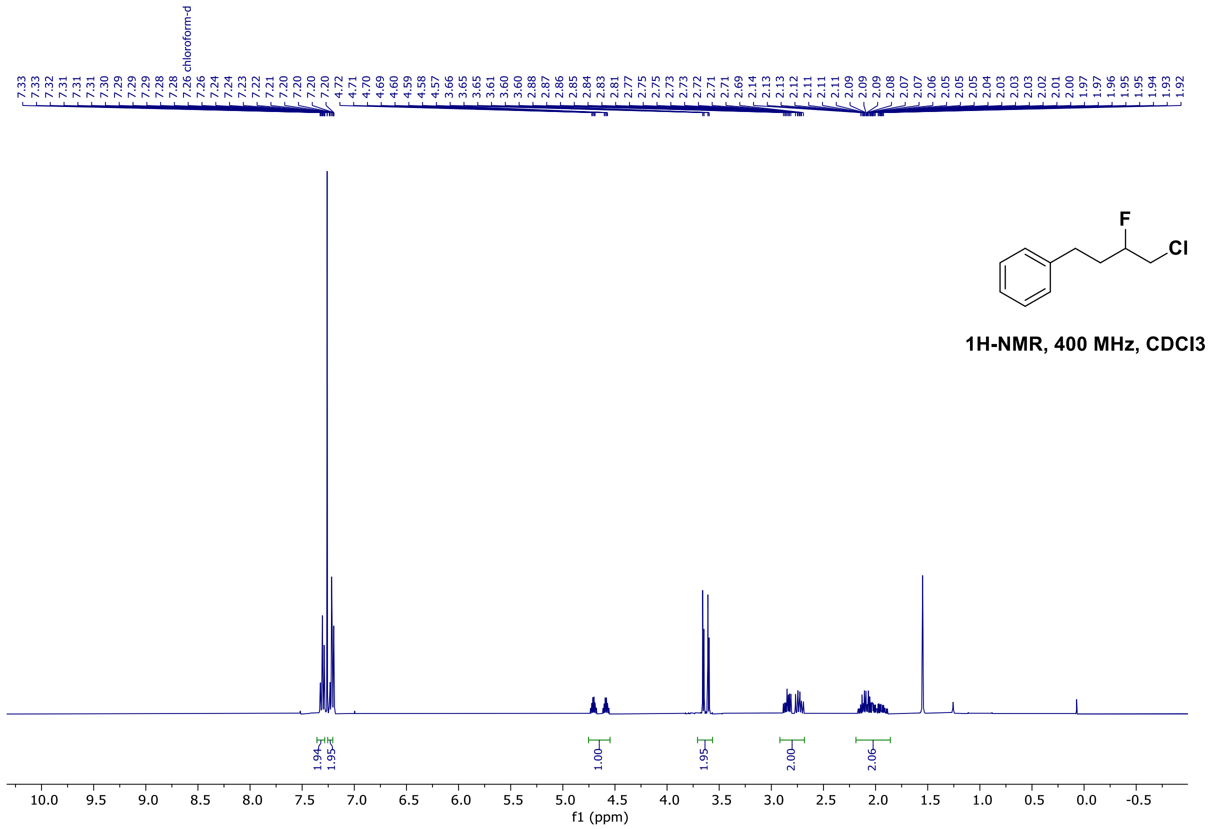
7.33
7.33
7.32
7.31
7.31
7.30
7.29
7.29
7.29
7.28
7.28
7.26 chloroform-d
7.26
7.24
7.24
7.23
7.22
7.21
7.20
7.20
7.20
7.20
4.72
4.71
4.69
4.60
4.59
4.58
4.57
3.66
3.65
3.65
3.61
3.60
3.60
2.88
2.87
2.86
2.85
2.84
2.83
2.81
2.77
2.75
2.75
2.73
2.73
2.72
2.71
2.71
2.69
2.14
2.13
2.13
2.12
2.11
2.11
2.11
2.09
2.09
2.09
2.08
2.07
2.07
2.06
2.05
2.05
2.05
2.04
2.04
2.03
2.03
2.03
2.02
2.02
2.01
2.00
1.97
1.97
1.96
1.95
1.95
1.94
1.93
1.92
F
Cl
1H-NMR, 400 MHz, CDCl3
1.94
1.95
1.00
1.95
2.00
2.06
f1 (ppm)

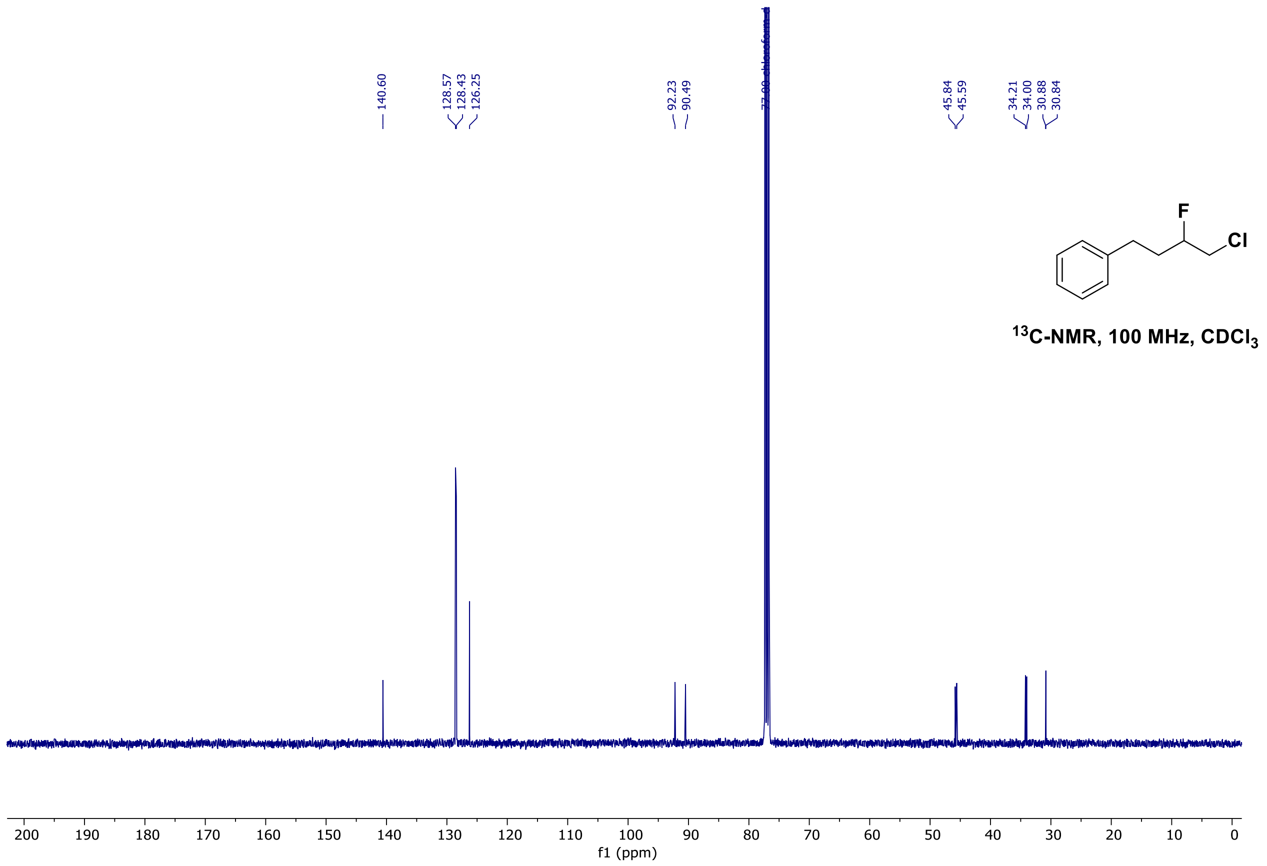

140.60
128.57
128.43
126.25
92.23
90.49
45.84
45.59
34.21
34.00
30.88
30.84
F
Cl
13C-NMR, 100 MHz, CDCl3
200 190 180 170 160 150 140 130 120 110 100 90 80 70 60 50 40 30 20 10 0
f1 (ppm)

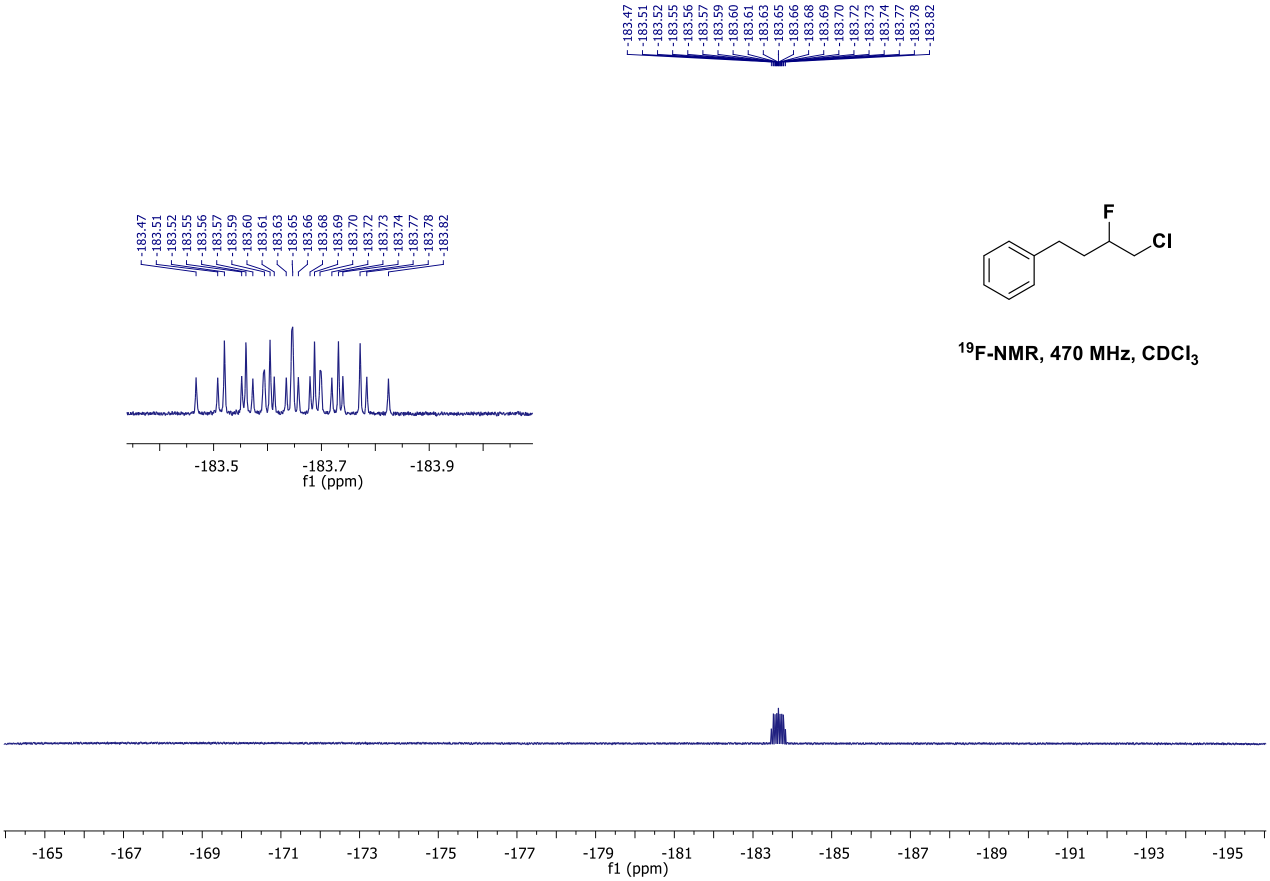

^{19}F-NMR, 470 MHz, $CDCl_3$

Compound 36

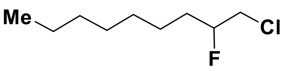

^{1}H NMR, 400 MHz, $CDCl_3$

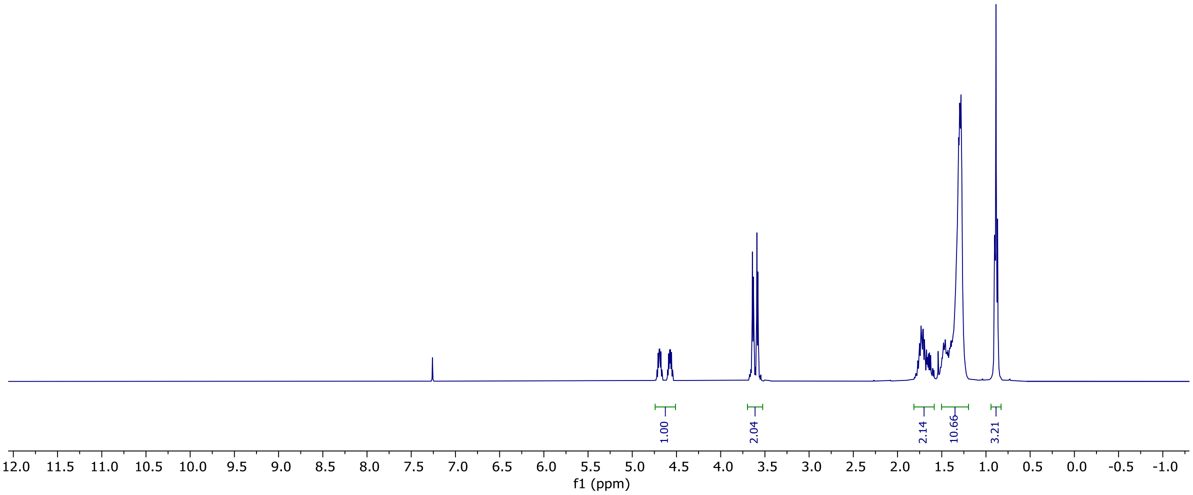

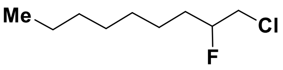

^{13}C NMR, 100 MHz, $CDCl_3$

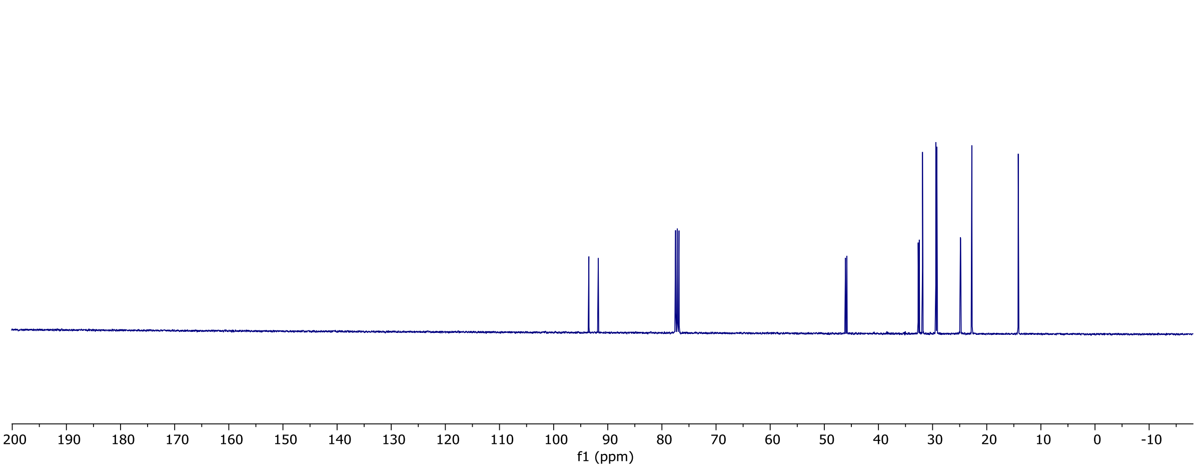

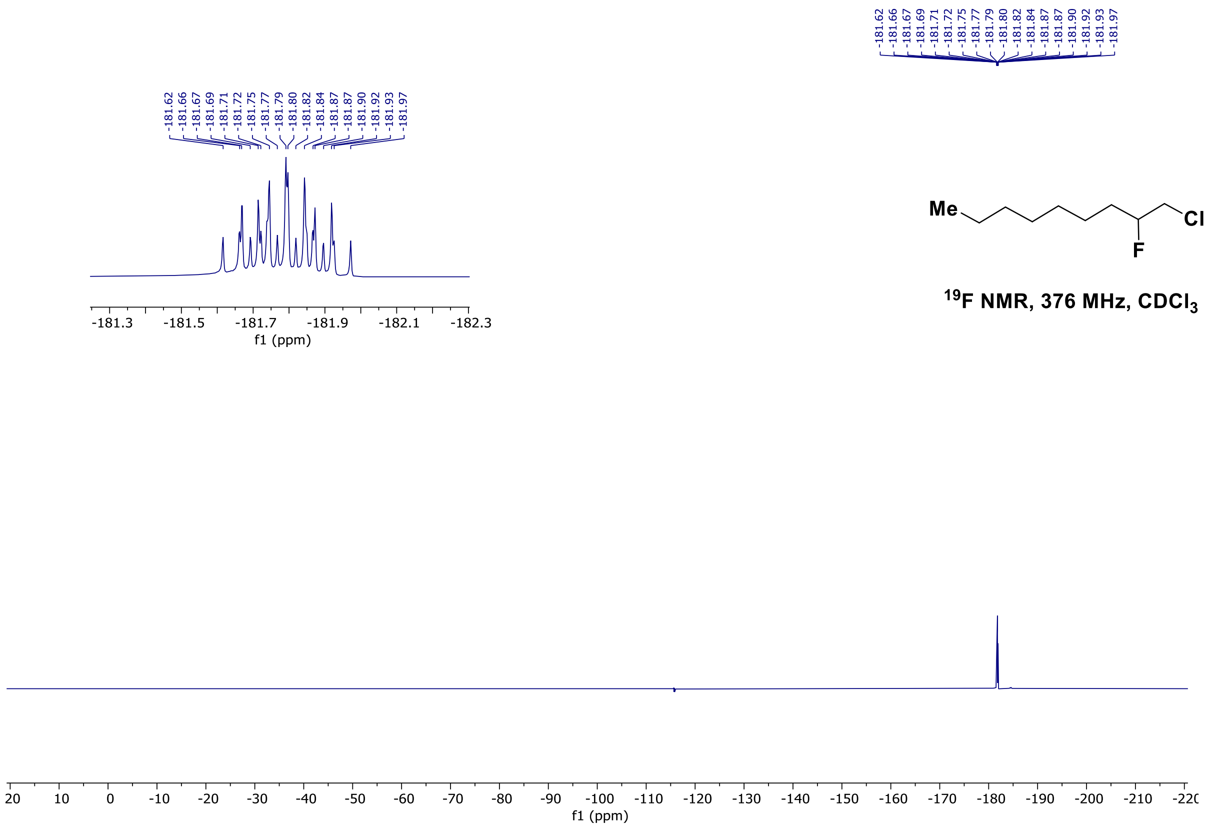

Compound 37

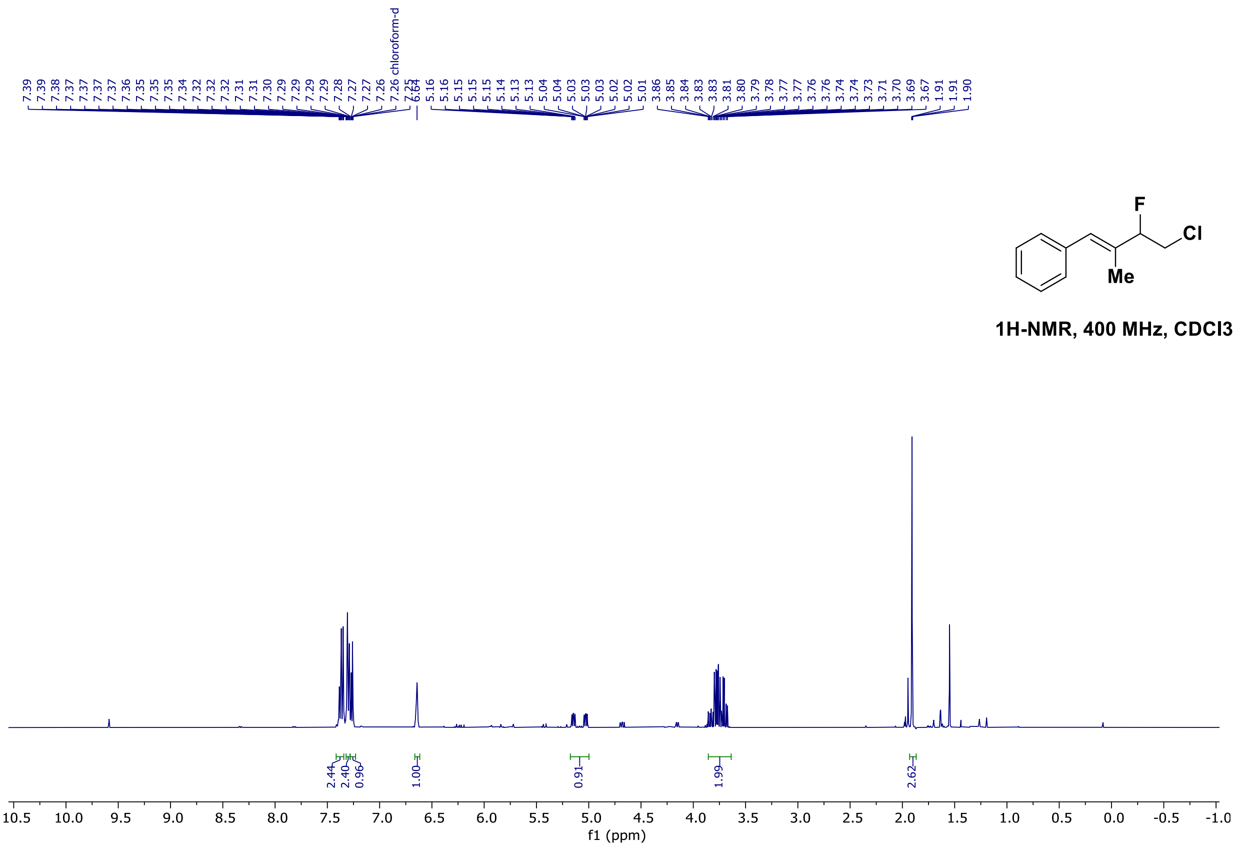

1H-NMR, 400 MHz, CDCl3

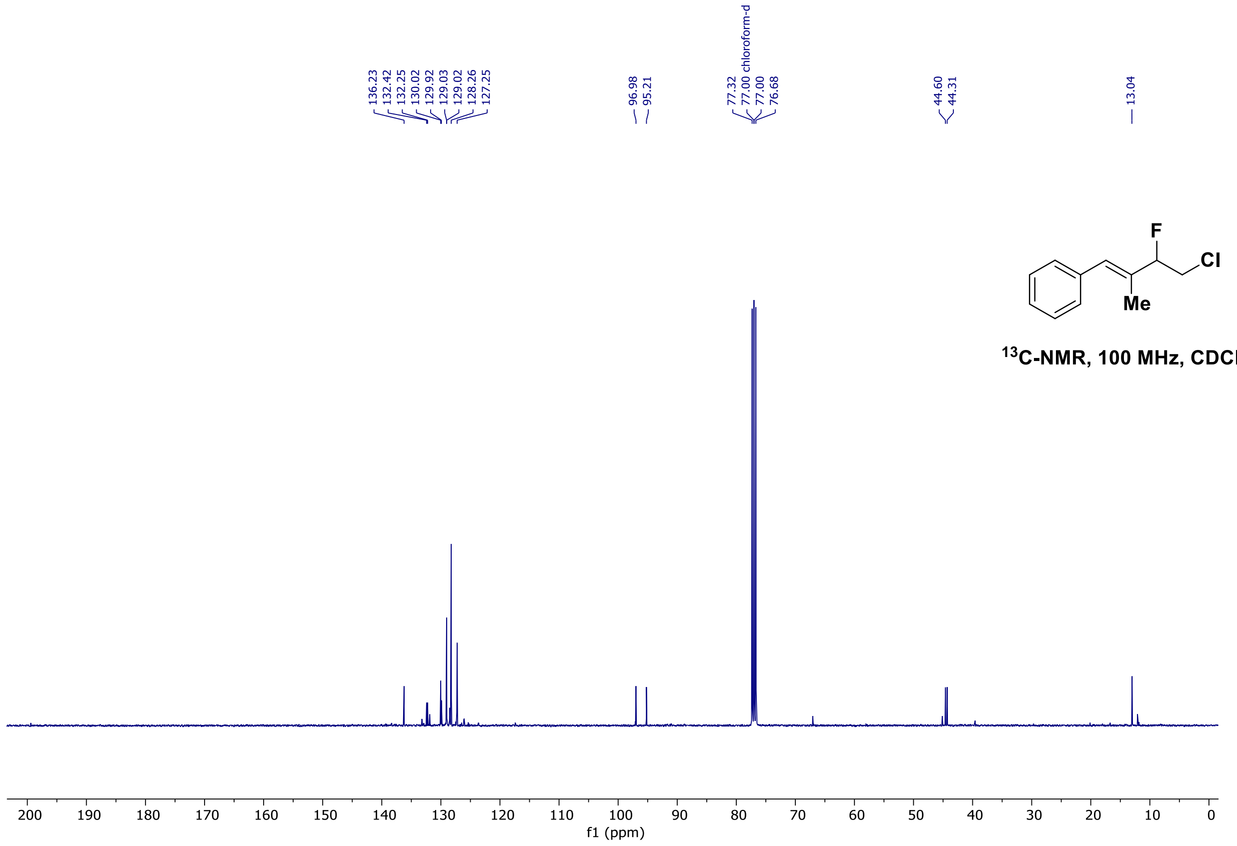

^{13}C-NMR, 100 MHz, $CDCl_3$

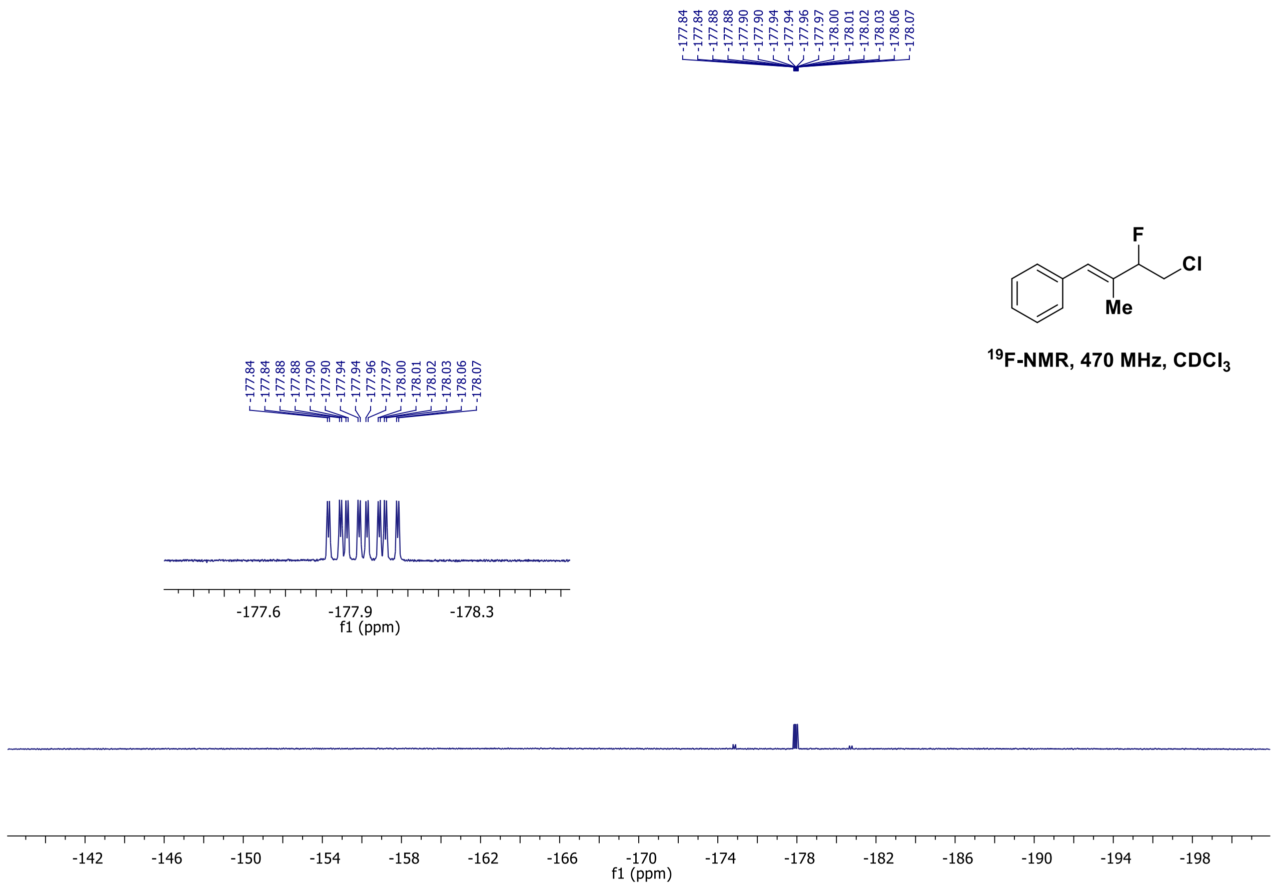

F
Cl
Me
^{19}F-NMR, 470 MHz, CDCl$_3$
-177.84
-177.84
-177.88
-177.88
-177.90
-177.90
-177.94
-177.94
-177.96
-177.97
-178.00
-178.01
-178.02
-178.03
-178.06
-178.07
-177.6
-177.9
-178.3
f1 (ppm)
-142
-146
-150
-154
-158
-162
-166
-170
-174
-178
-182
-186
-190
-194
-198
f1 (ppm)

Compound 38

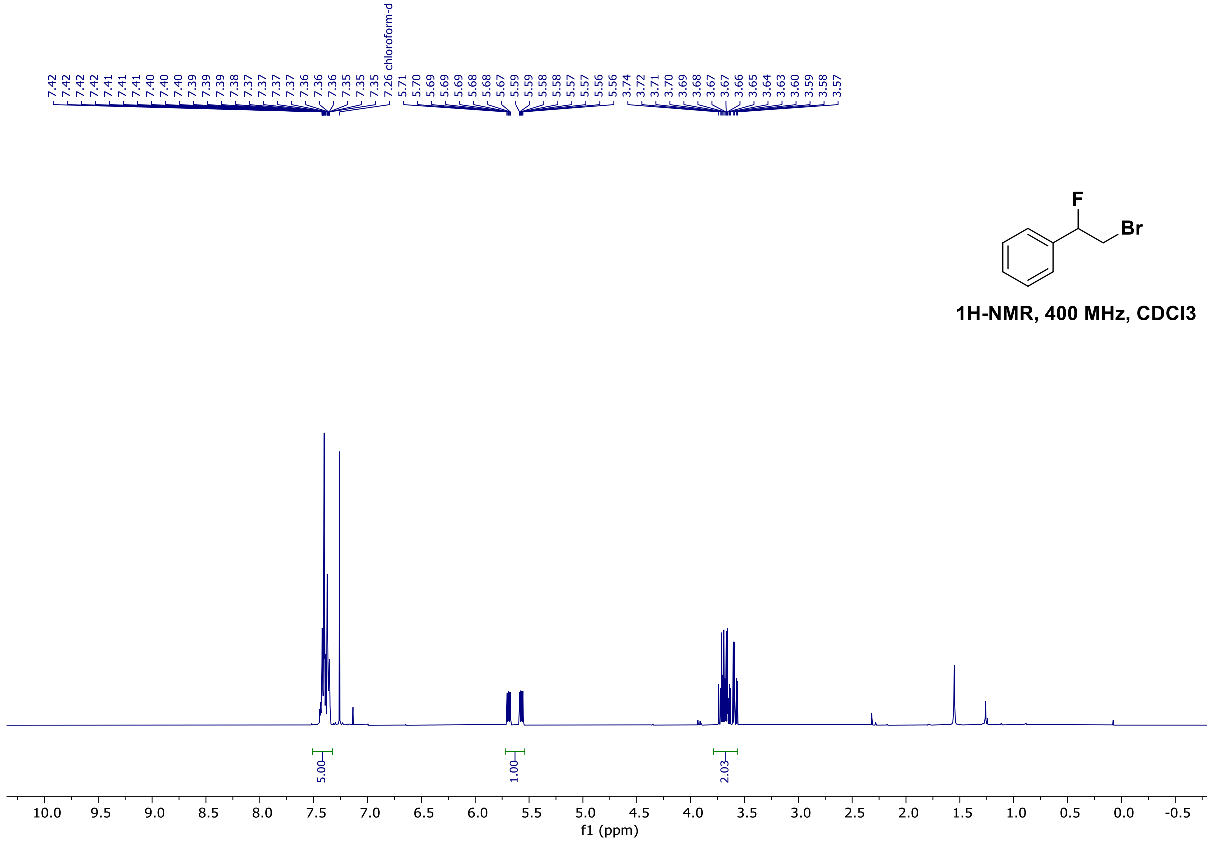

1H-NMR, 400 MHz, CDCl3

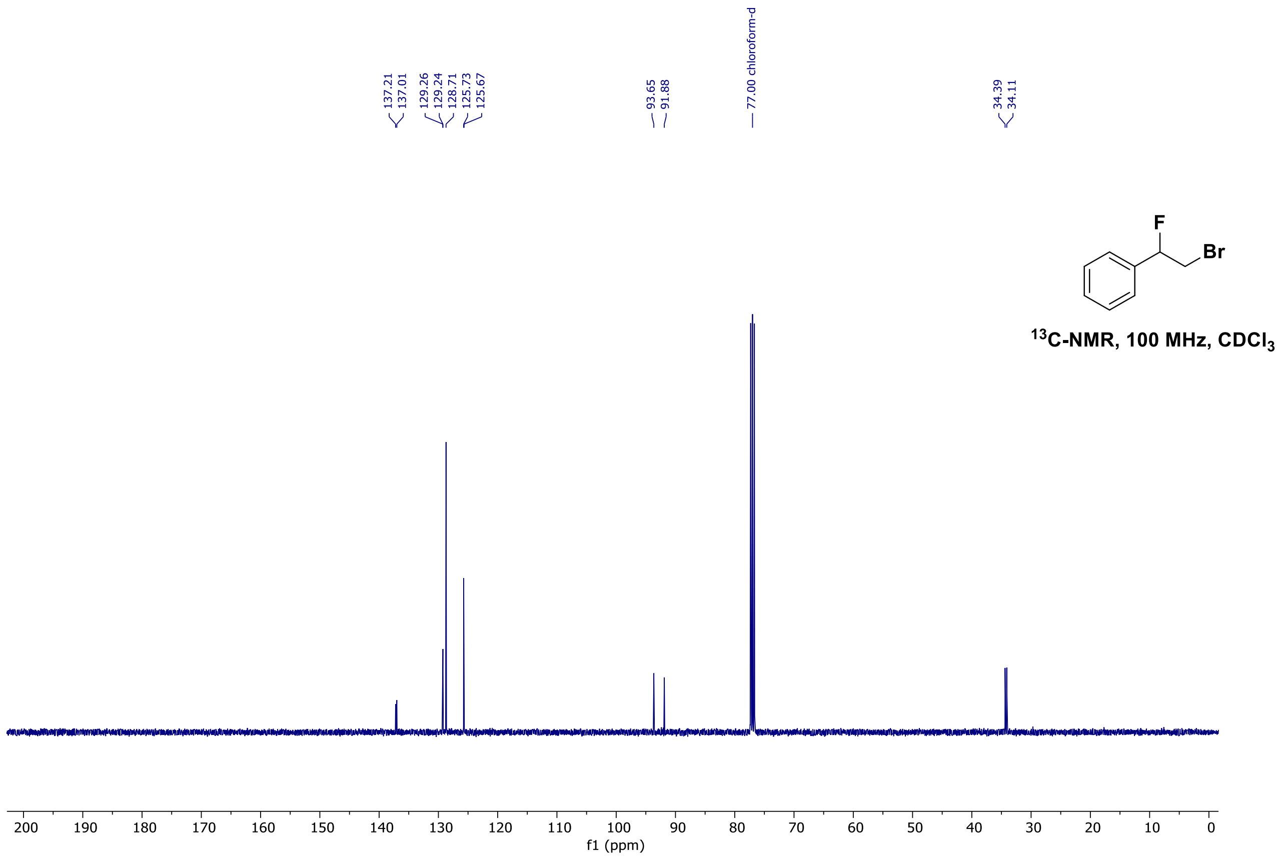

137.21
137.01
129.26
129.24
128.71
125.73
125.67
93.65
91.88
77.00 chloroform-d
34.39
34.11
F
Br
13C-NMR, 100 MHz, CDCl3
200
190
180
170
160
150
140
130
120
110
100
90
80
70
60
50
40
30
20
10
0
f1 (ppm)

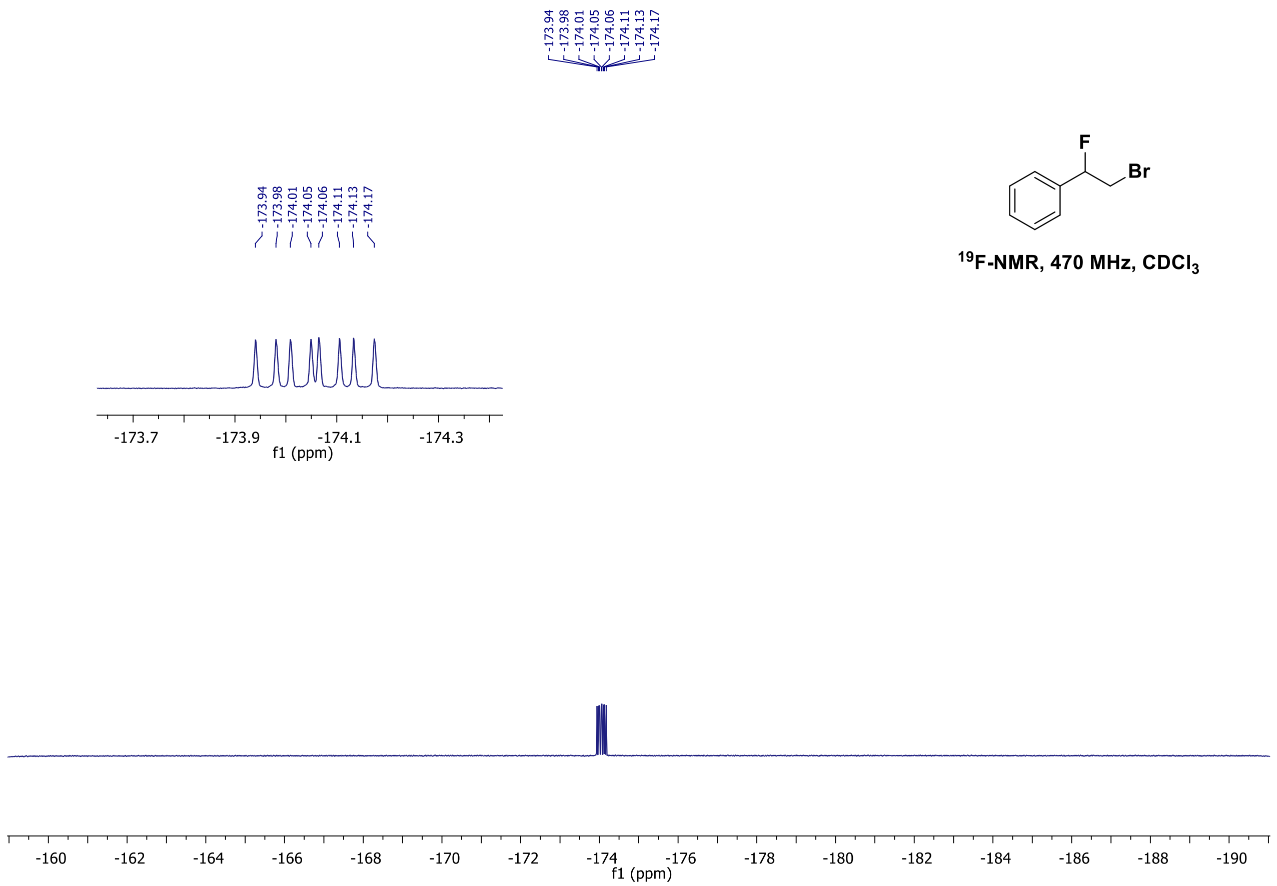

^{19}F-NMR, 470 MHz, CDCl$_3$

Compound 39

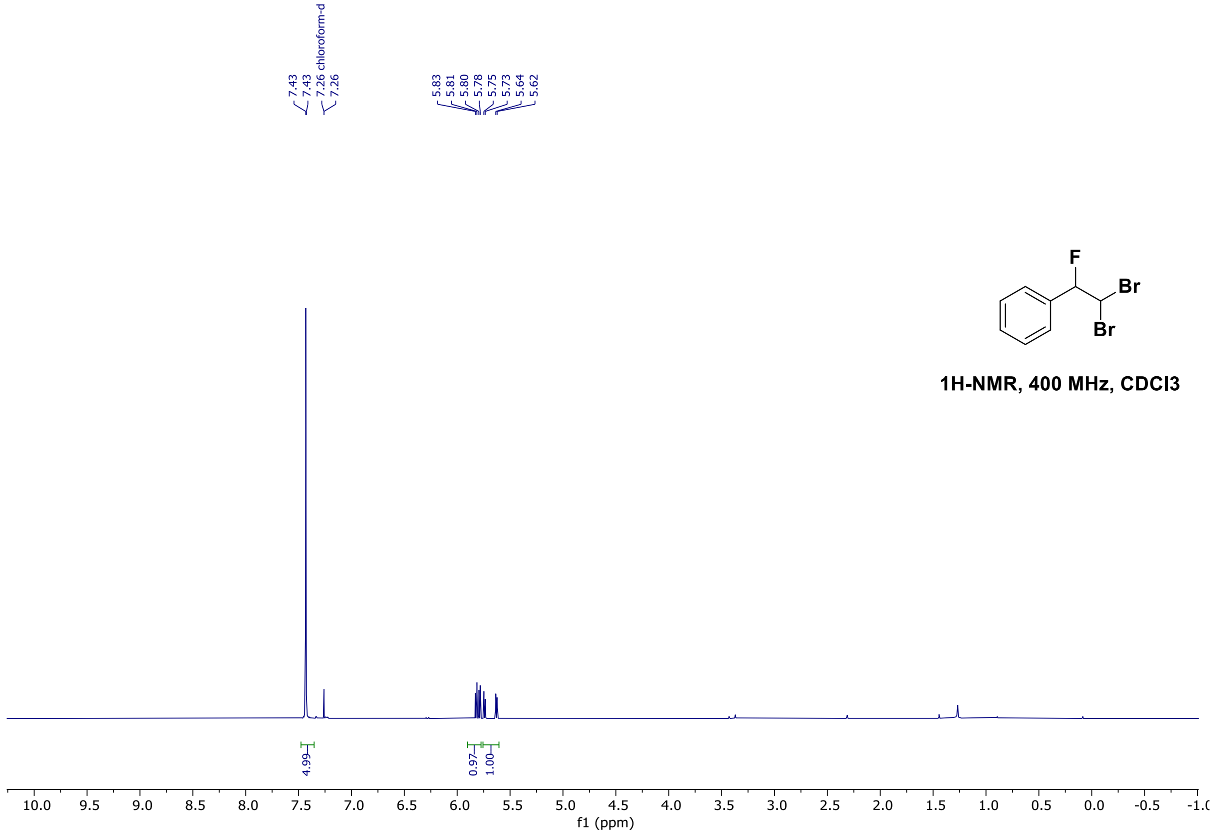

7.43
7.43
7.26 chloroform-d
7.26
5.83
5.81
5.80
5.78
5.75
5.73
5.64
5.62
F
Br
Br
1H-NMR, 400 MHz, CDCl3
4.99
0.97
1.00
10.0
9.5
9.0
8.5
8.0
7.5
7.0
6.5
6.0
5.5
5.0
4.5
4.0
3.5
3.0
2.5
2.0
1.5
1.0
0.5
0.0
-0.5
f1 (ppm)

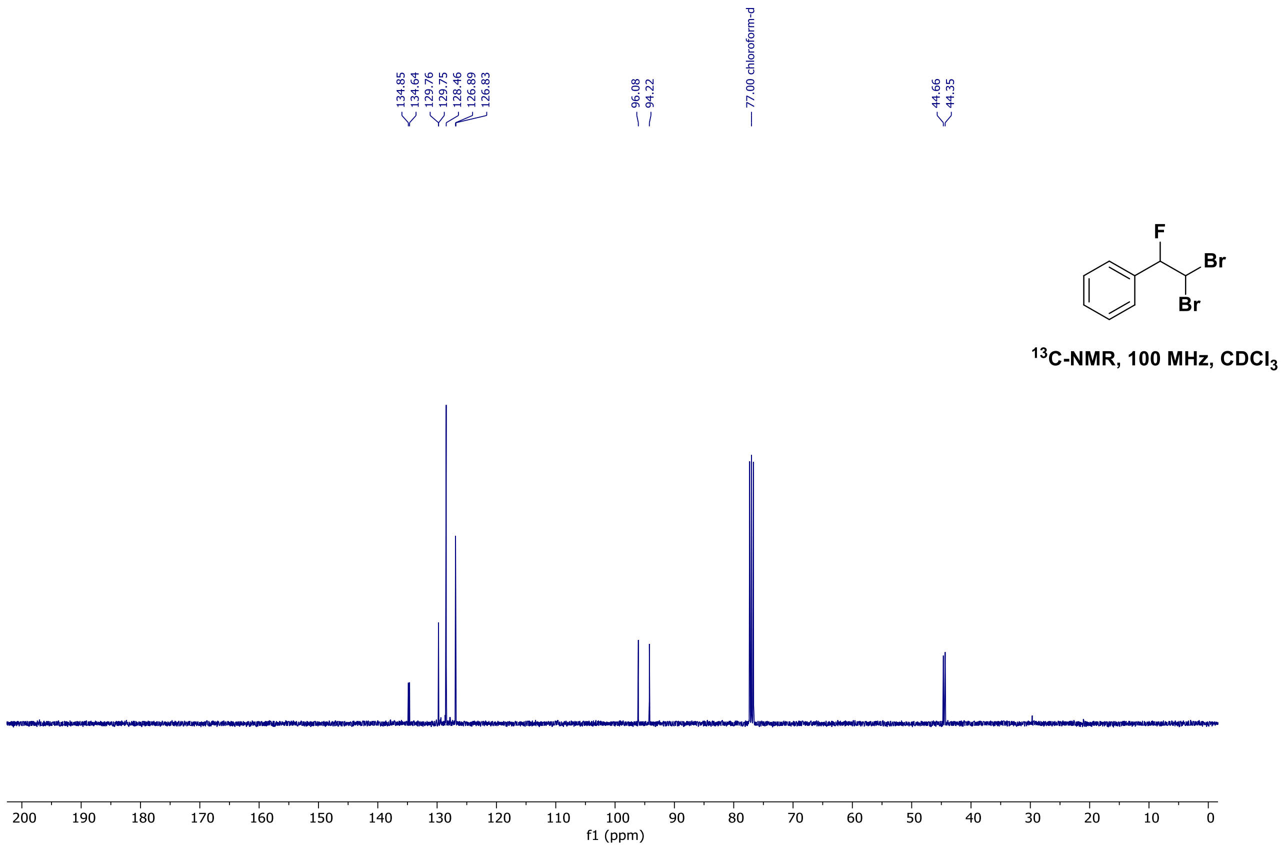
134.85
134.64
129.76
129.75
128.46
126.89
126.83
96.08
94.22
77.00 chloroform-d
44.66
44.35
F
Br
Br
^{13}C-NMR, 100 MHz, CDCl$_3$
200 190 180 170 160 150 140 130 120 110 100 90 80 70 60 50 40 30 20 10 0
f1 (ppm)

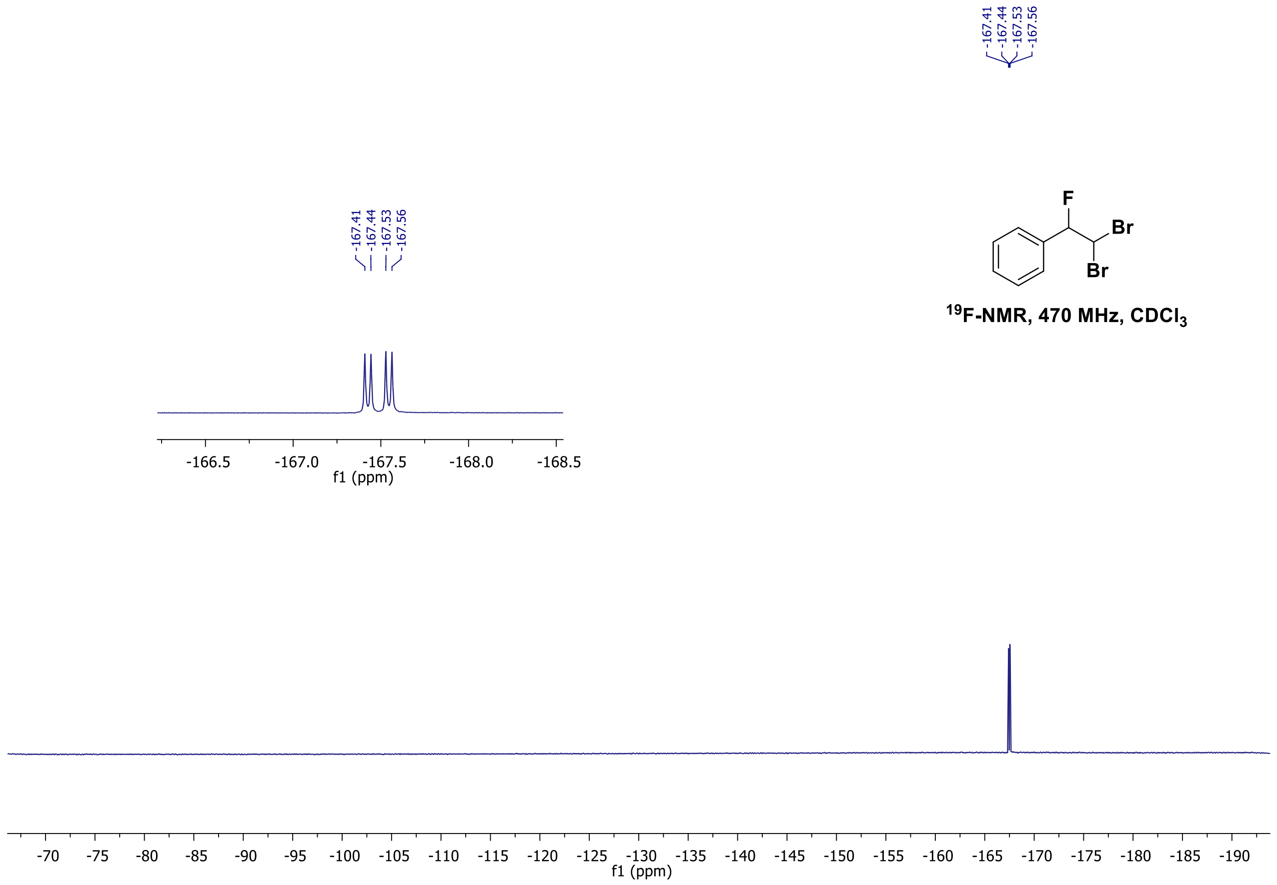
-167.41
-167.44
-167.53
-167.56
F
Br
Br
^{19}F-NMR, 470 MHz, CDCl$_3$
-166.5 -167.0 -167.5 -168.0 -168.5
f1 (ppm)
-70 -75 -80 -85 -90 -95 -100 -105 -110 -115 -120 -125 -130 -135 -140 -145 -150 -155 -160 -165 -170 -175 -180 -185 -190
f1 (ppm)

Compound 40

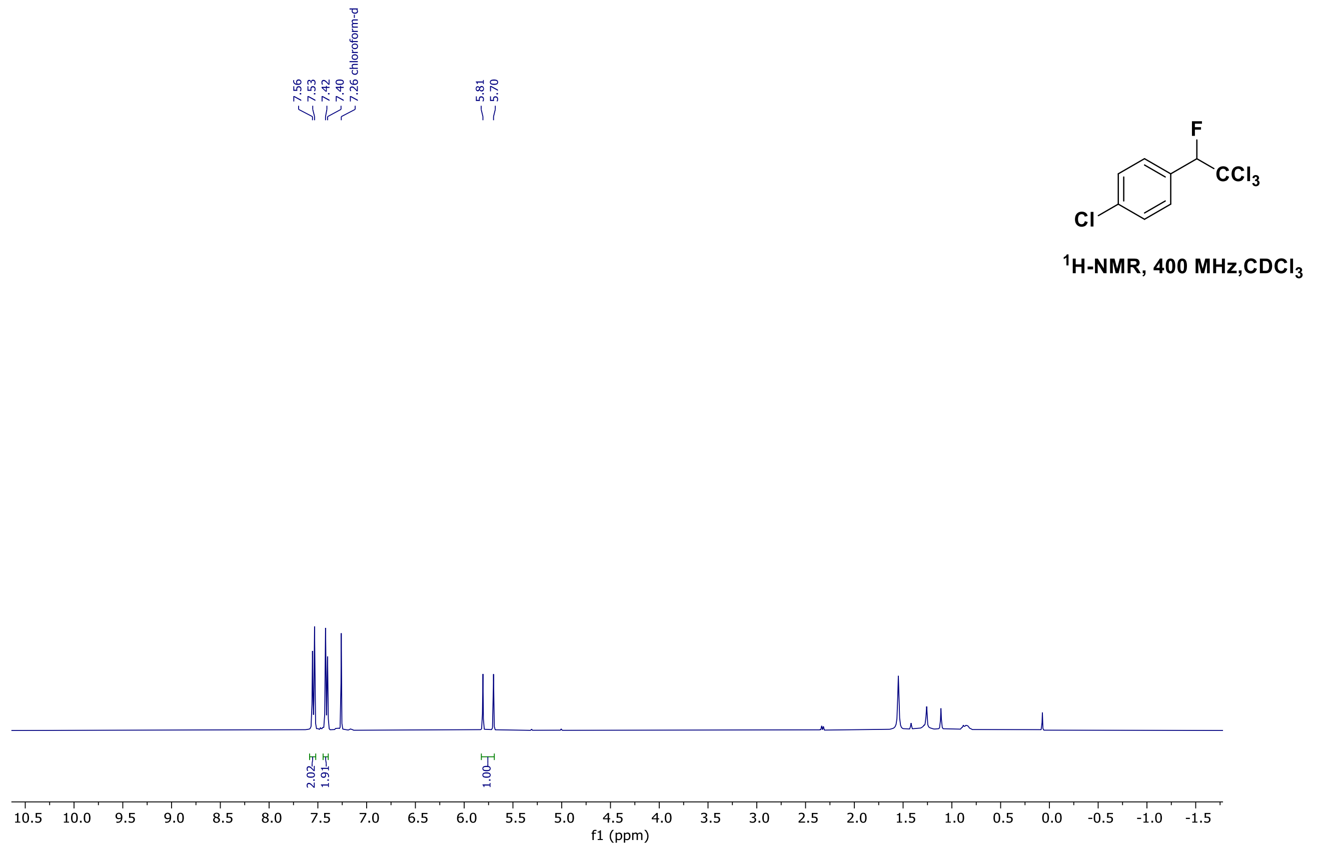

7.56
7.53
7.42
7.40
7.26 chloroform-d
5.81
5.70
F
CCl3
Cl
1H-NMR, 400 MHz,CDCl3
2.02
1.91
1.00
f1 (ppm)

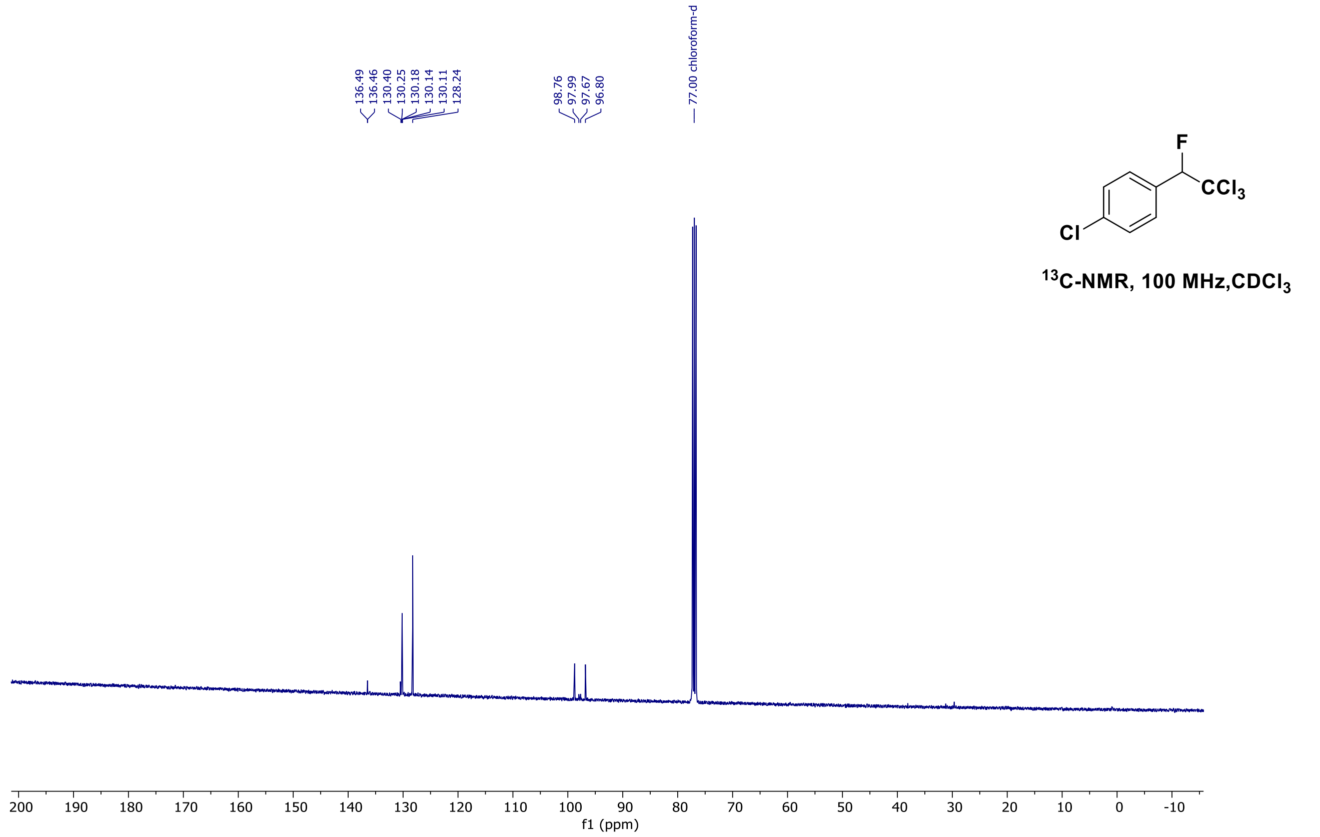

136.49
136.46
130.40
130.25
130.18
130.14
130.11
128.24
98.76
97.99
97.67
96.80
77.00 chloroform-d
F
CCl3
Cl
13C-NMR, 100 MHz,CDCl3
f1 (ppm)

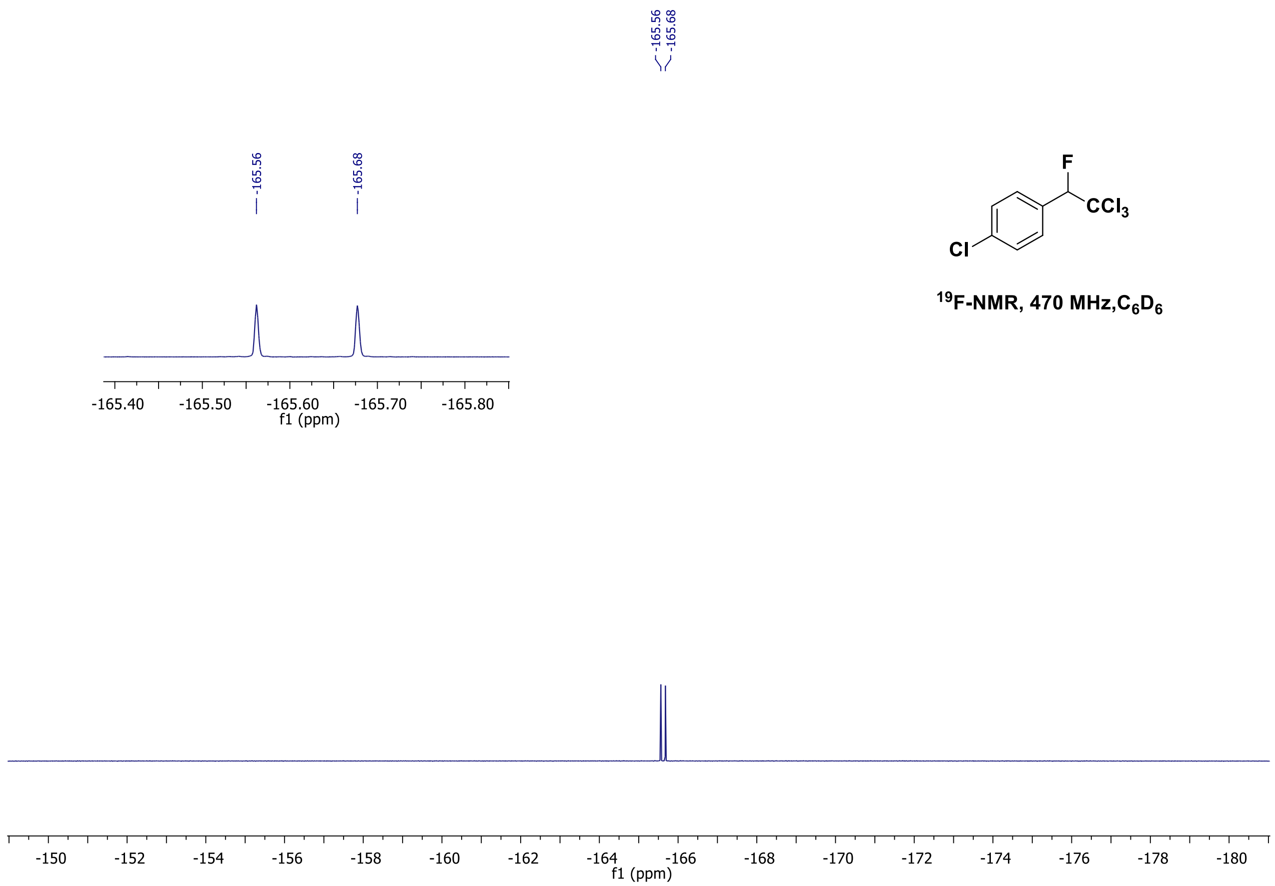

^{19}F-NMR, 470 MHz,C_6D_6

Compound 41

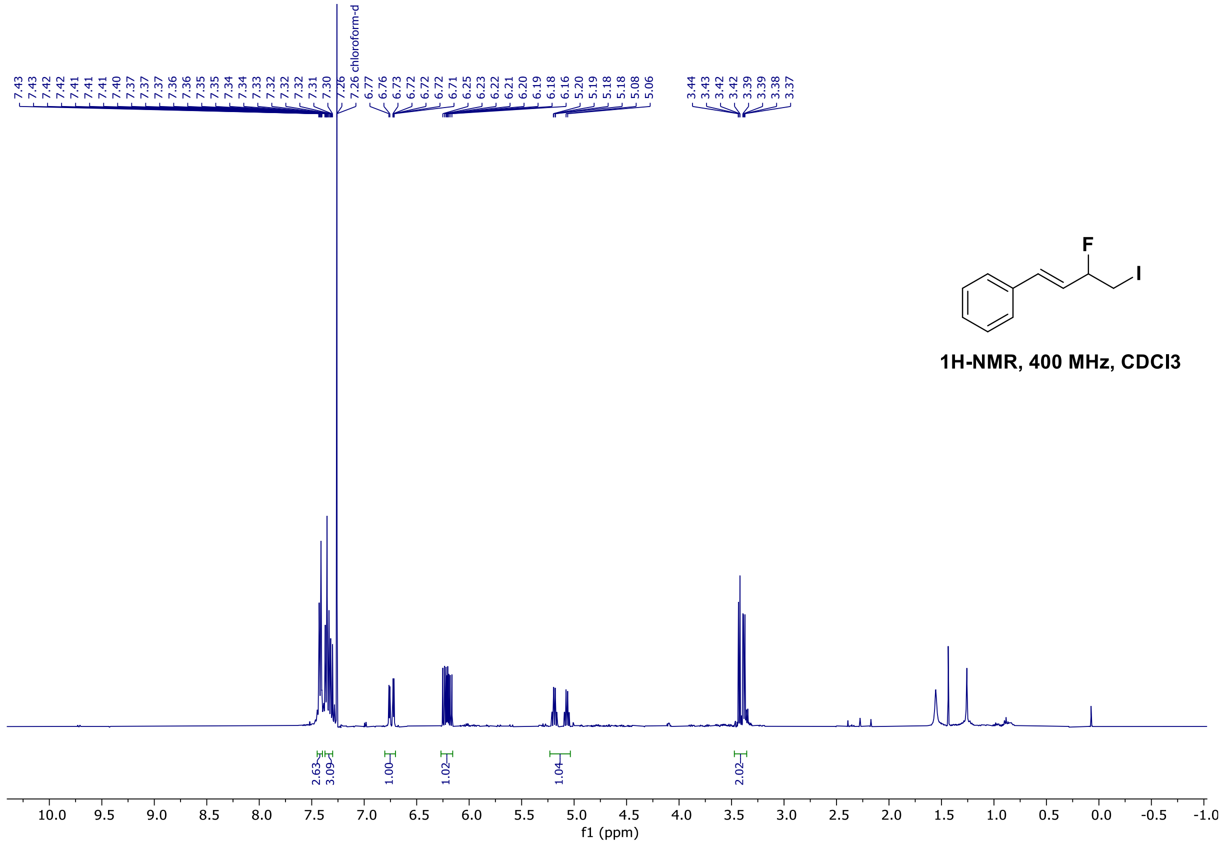

7.43
7.43
7.42
7.42
7.41
7.41
7.40
7.37
7.37
7.36
7.36
7.35
7.35
7.34
7.34
7.33
7.32
7.32
7.32
7.31
7.30
7.26
7.26 chloroform-d
6.77
6.76
6.73
6.72
6.72
6.72
6.71
6.25
6.23
6.22
6.21
6.20
6.19
6.18
6.16
5.20
5.19
5.18
5.18
5.08
5.06
3.44
3.43
3.42
3.42
3.39
3.39
3.38
3.37
F
I
1H-NMR, 400 MHz, CDCl3
2.63
3.09
1.00
1.02
1.04
2.02
10.0
9.5
9.0
8.5
8.0
7.5
7.0
6.5
6.0
5.5
5.0
4.5
4.0
3.5
3.0
2.5
2.0
1.5
1.0
0.5
0.0
-0.5
-1.0
f1 (ppm)

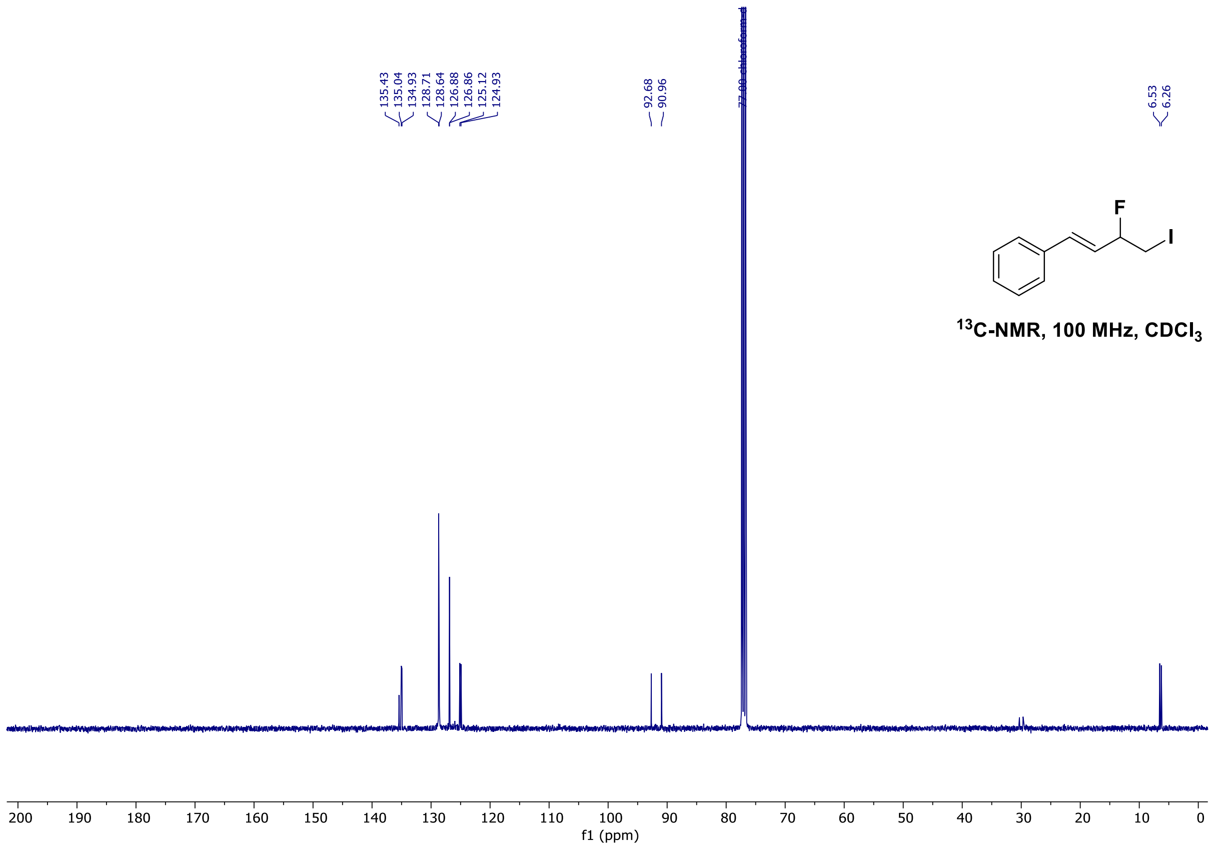
135.43
135.04
134.93
128.71
128.64
126.88
126.86
125.12
124.93
92.68
90.96
6.53
6.26
F
I
^{13}C-NMR, 100 MHz, CDCl$_3$
200 190 180 170 160 150 140 130 120 110 100 90 80 70 60 50 40 30 20 10 0
f1 (ppm)

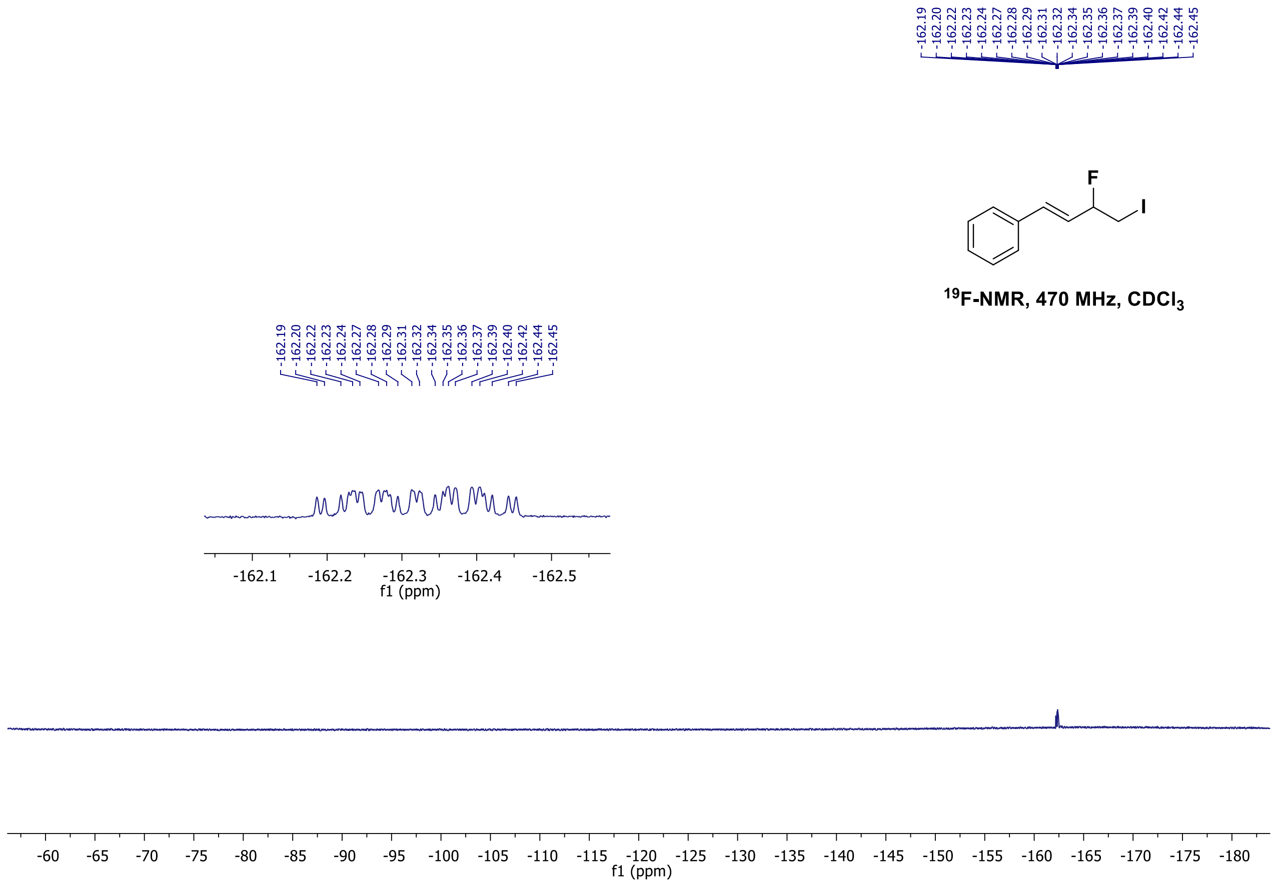
-162.19
-162.20
-162.22
-162.23
-162.24
-162.27
-162.28
-162.29
-162.31
-162.32
-162.34
-162.35
-162.36
-162.37
-162.39
-162.40
-162.42
-162.44
-162.45
F
I
^{19}F-NMR, 470 MHz, CDCl$_3$
-162.1 -162.2 -162.3 -162.4 -162.5
f1 (ppm)
-60 -65 -70 -75 -80 -85 -90 -95 -100 -105 -110 -115 -120 -125 -130 -135 -140 -145 -150 -155 -160 -165 -170 -175 -180
f1 (ppm)

Compound 42

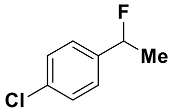

1H-NMR, 400 MHz, CDCl3

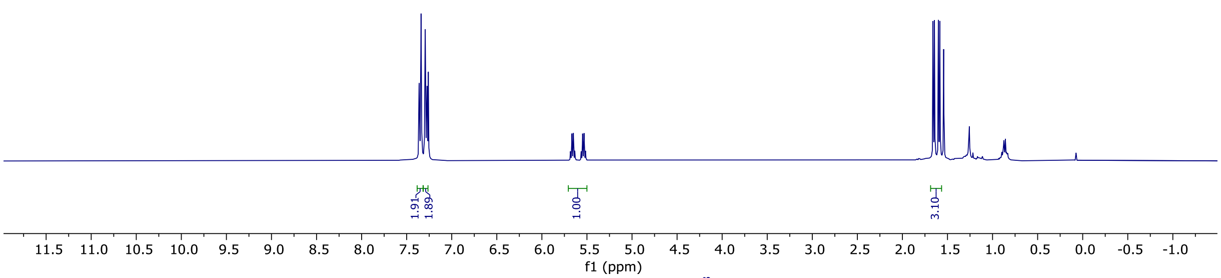

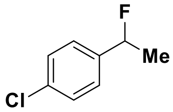

^{13}C-NMR, 100 MHz, $CDCl_3$

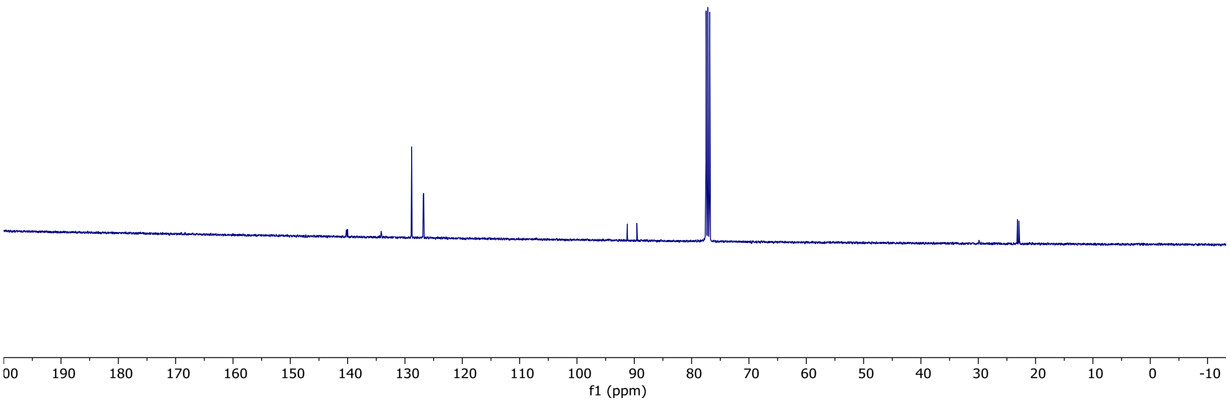

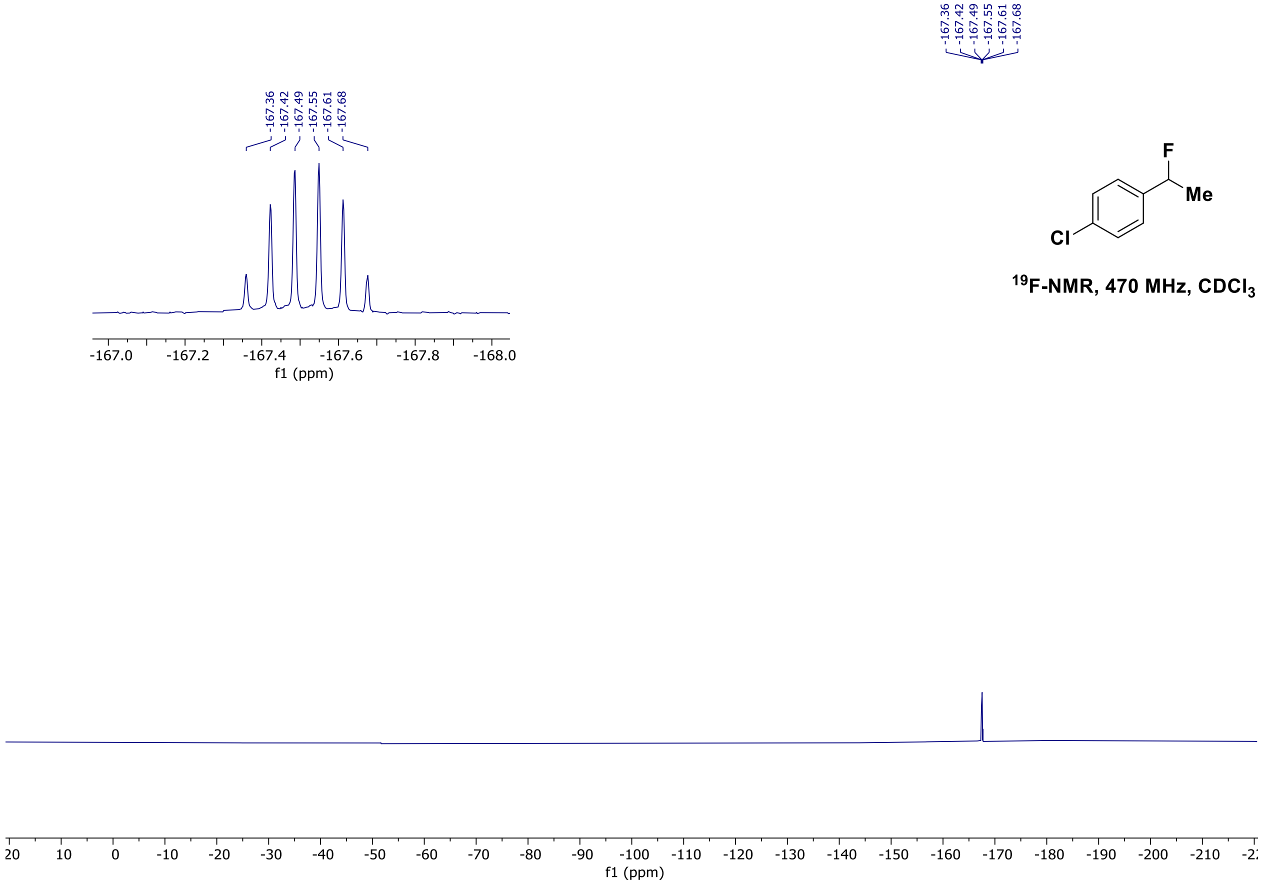
-167.36
-167.42
-167.49
-167.55
-167.61
-167.68
-167.0 -167.2 -167.4 -167.6 -167.8 -168.0
f1 (ppm)
F
Me
Cl
19F-NMR, 470 MHz, CDCl3
20 10 0 -10 -20 -30 -40 -50 -60 -70 -80 -90 -100 -110 -120 -130 -140 -150 -160 -170 -180 -190 -200 -210
f1 (ppm)

6. X-ray Analysis

The X-ray intensity data were measured on Bruker D8 Venture diffractometer, equipped with Oxford cooling system. The structures were solved by *Intrinsic Phasing.* Non-hydrogen atoms were refined with *anisotropic displacement parameters*. Hydrogen atoms were inserted at calculated positions and refined with riding model. Structure visible in Figure 1, data quality discussed in Figure 2. Measurement conditions listed in Table 1, sample and crystal data, data collection and structure refinement details listed in Table 2.
Further details on experimental data and used software are available online: http://www.ccdc.cam.ac.uk/conts/retrieving.html) .

Table 1 Experimental parameter and CCDC-Code.

Sample	Machine	Source	Temp.	Detector Distance	Time/ Frame	#Frames	Frame width	CCDC
			[K]	[mm]	[s]		[°]	
Compound **40**	Bruker D8	Mo	100	40	30	797	0.36	2410335

Compound 40

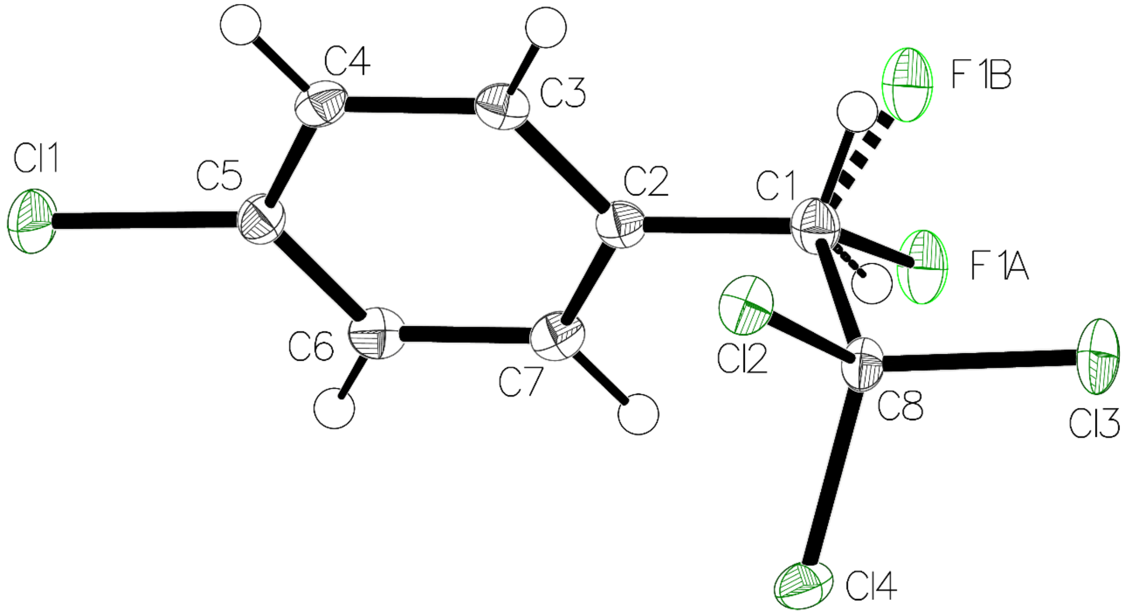

Figure 1 Asymmetric Unit of drawn with 50% displacement ellipsoid. The bond precision for C-C single bonds is 0.0017 Å. Disorder on F1 in the size of 96.9/3.1 % illustrated.

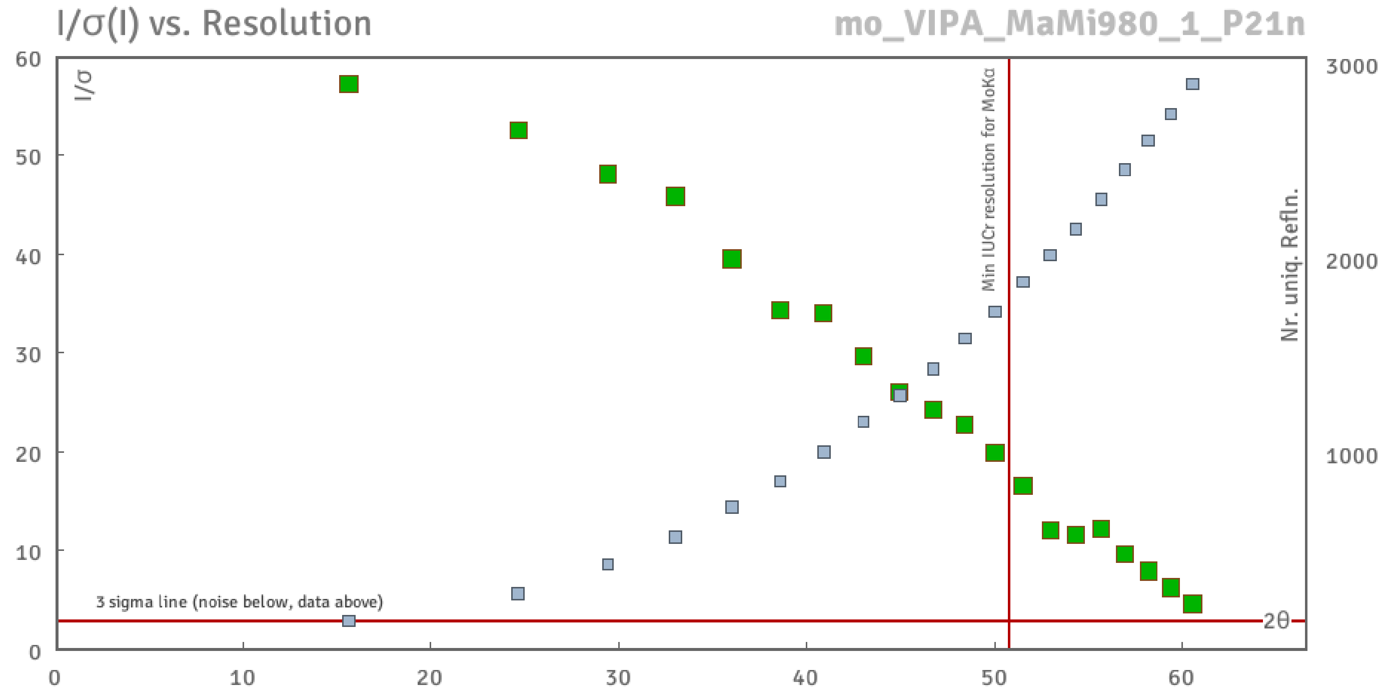

Figure 2 Data quality I 3 sigma line: All data are above the “noise level” line along the min IUCR definition.

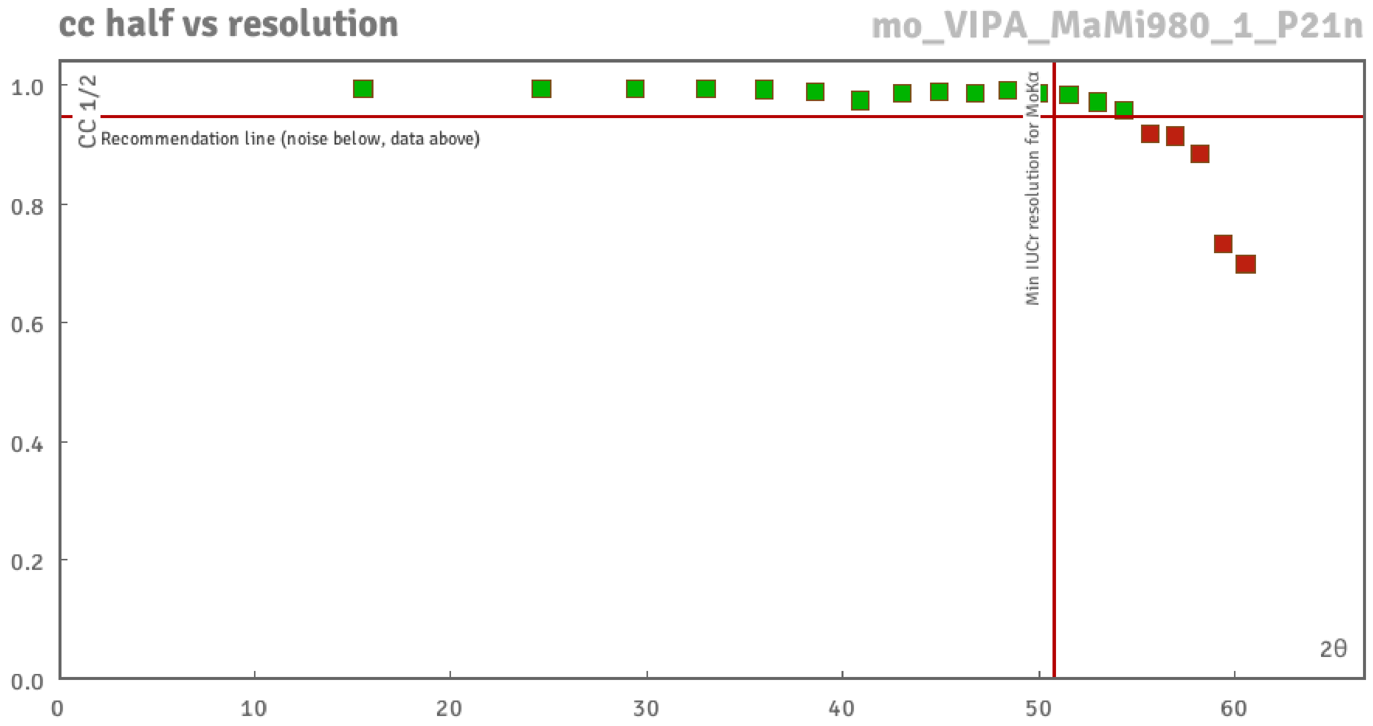

Figure 3 Data quality II CC1/2: All data are above the “noise level” line along the min IUCR definition.

Table 2 Sample and crystal data, Data collection and structure refinement.

Identification code	mo_VIPA_MaMi980_1_P21n
Empirical formula	$C_8H_5Cl_4F$
Formula weight	261.92
Temperature/K	100.0
Crystal system	monoclinic
Space group	$P2_1/n$
a/Å	9.8894(3)
b/Å	6.0908(3)
c/Å	16.5497(9)
α/°	90
β/°	96.434(2)
γ/°	90
Volume/Å^3	990.58(8)
Z	4
ρ_{calc}g/cm^3	1.756
μ/mm^{-1}	1.154
F(000)	520.0
Crystal size/mm^3	0.05 × 0.04 × 0.03
Radiation	MoKα (λ = 0.71073)
2Θ range for data collection/°	4.584 to 61.086
Index ranges	-14 ≤ h ≤ 13, -8 ≤ k ≤ 6, -21 ≤ l ≤ 23
Reflections collected	10830
Independent reflections	2927 [R_{int} = 0.0296, R_{sigma} = 0.0328]
Data/restraints/parameters	2927/1/125
Goodness-of-fit on F^2	1.113
Final R indexes [I>=2σ (I)]	R_1 = 0.0233, wR_2 = 0.0561
Final R indexes [all data]	R_1 = 0.0328, wR_2 = 0.0596
Largest diff. peak/hole / e Å^{-3}	0.48/-0.30
